# R2 and Non-Site-Specific R2-Like Retrotransposons of the German Cockroach, *Blattella germanica*

**DOI:** 10.3390/genes11101202

**Published:** 2020-10-15

**Authors:** Arina Zagoskina, Sergei Firsov, Irina Lazebnaya, Oleg Lazebny, Dmitry V. Mukha

**Affiliations:** Vavilov Institute of General Genetics Russian Academy of Sciences, 119991 Moscow, Russia; arisha-kag@mail.ru (A.Z.); firsov@vigg.ru (S.F.); Lazebnaya@mail.ru (I.L.); oelazebny@gmail.com (O.L.)

**Keywords:** R2 non-LTR retrotransposons, protein domain organization, recombination, mobile element activity, ribosomal RNA gene cluster, 5′-truncated copies, population structure

## Abstract

The structural and functional organization of the ribosomal RNA gene cluster and the full-length R2 non-LTR retrotransposon (integrated into a specific site of 28S ribosomal RNA genes) of the German cockroach, *Blattella germanica*, is described. A partial sequence of the R2 retrotransposon of the cockroach *Rhyparobia maderae* is also analyzed. The analysis of previously published next-generation sequencing data from the *B. germanica* genome reveals a new type of retrotransposon closely related to R2 retrotransposons but with a random distribution in the genome. Phylogenetic analysis reveals that these newly described retrotransposons form a separate clade. It is shown that proteins corresponding to the open reading frames of newly described retrotransposons exhibit unequal structural domains. Within these retrotransposons, a recombination event is described. New mechanism of transposition activity is discussed. The essential structural features of R2 retrotransposons are conserved in cockroaches and are typical of previously described R2 retrotransposons. However, the investigation of the number and frequency of 5′-truncated R2 retrotransposon insertion variants in eight *B. germanica* populations suggests recent mobile element activity. It is shown that the pattern of 5′-truncated R2 retrotransposon copies can be an informative molecular genetic marker for revealing genetic distances between insect populations.

## 1. Introduction

Transposable elements (TEs) are ubiquitous components of eukaryotic genomes that are important for shaping genetic material and genome evolution (for review, see [1,2,3,4,5,6,7]).

TEs can be divided into two classes: retrotransposons (Class I) and DNA transposons (Class II). All retrotransposons are transposed through an RNA intermediate. Messenger RNA from retrotransposons is expressed in host cells, and after reverse transcription by reverse transcriptases (RTs) that are encoded by TEs, new DNA copies of the elements are integrated into new sites within the host genome. In contrast, DNA transposons are transposed from one genome site to another via the movement of DNA copies through the activity of DNA transposases encoded by TEs [8,9,10].

Retrotransposons can be divided into four types: non-long terminal repeat (non-LTR) retrotransposons, LTR retrotransposons, *Penelope* retrotransposons, and *DIRS* retrotransposons. Based on structural features and RT domain phylogeny, non-LTR retrotransposons are divided into 28 clades [9]. Analyses revealed that several lineages of the R2 clade have been maintained for a long time in animals. Four supergroups of the R2 clade—R2A, R2B, R2C, and R2D—show independent lineages in the phylogenetic tree based on their reverse transcriptase sequences. These four supergroups are further classified into 11 clades belonging to the R2 superclade [11]. Notably, in addition to the R2 retrotransposons, the R2 superclade includes the R8 and R9 retrotransposons.

The vast majority of the R2 retrotransposons described to date recognize the same highly conserved target sequence within the 28S rRNA gene (5′-AAGG ↓ TAGC-3′). Only recently have two families of R2 retrotransposons that are not inserted into ribosomal RNA genes been described. One is *R2NS-1_SMed* from the Mediterranean planarian *Schmidtea mediterranea*, belonging to the R2D clade, and the other is *R2NS-1_CSi* from the liver fluke *Clonorchis sinensis*, belonging to the R2C clade [12]. R8 and R9 retrotransposons exhibit unique integration sites within 18S and 28S rRNA genes, respectively [13,14].

R2 retrotransposons are one of the most intensively investigated groups of TEs. R2 retrotransposons have a single open reading frame (ORF) flanked by two untranslated sequences of variable length. The R2 protein product shows several highly conserved regions: a nucleic acid binding domain at the N-terminus, a central reverse transcriptase (RT) domain followed by one zinc-finger motif (CCHC) and an endonuclease-like (EN) domain [11,15,16]. Some R2 retrotransposons end with simple sequence repeats due to the RT addition of non-templated nucleotides [17]. The protein’s N-terminal domain can contain one, two, or three cysteine-histidine zinc-finger motifs and conserved c-myb-like DNA binding motifs [11,15]. When three zinc fingers are present, the motifs are arranged as follows: CCHH, CCHC, and CCHH.

R2 is inserted through a target-primed reverse transcription mechanism [18,19,20,21,22]. It has been shown that the 5′-ends of R2 retrotransposons are often truncated in the course of transposition, since their reverse transcriptase sometimes incompletely reads the RNA template of the TE for unknown reasons. As a result, both full-size functional copies of these elements and 5′-truncated copies of different lengths are integrated into the target site. The truncation variants can be used to monitor R2 retrotransposon activity [23,24,25,26,27,28,29].

R2 retrotransposons were originally identified as insertion sequences in the 28S ribosomal RNA (rRNA) genes of the fruit fly *Drosophila melanogaster* [30] and the domestic silkworm, *Bombyx mori* [31]. To date, this type of TE has been reported in Echinodermata, Platyhelminthes, Nematoda, and Cnidaria as well as Arthropoda and Chordata, including vertebrates [11,12,13,32,33,34].

The German cockroach, *B. germanica* (Linnaeus), is a cosmopolitan pest species that is obligately commensal with humans (associated strictly with human habitations, farms, food stores, waste areas, and other anthropogenic habitats and not known to occupy any natural habitats). *B. germanica* represents an ideal species for studying the genetic diversity and connectivity among geographically separate populations linked solely by human-mediated dispersal. Recognized globally as a prominent household pest of medical, veterinary, and economic significance [35,36,37,38], this species exhibits the relatively unique behavior of strict human commensalism. The effect of this unique behavior on the genetic diversity and population differentiation of *B. germanica* has been addressed in a number of studies employing molecular markers. Currently, the variability of the nucleotide sequences of the non-transcribed ribosomal DNA spacers [39] and the variability of the length of variable microsatellite loci [40,41,42] are used as the most informative molecular markers that allow us to study the structure of *B. germanica* populations. Despite the rather high information content of these markers, the aim of developing new molecular genetic markers that allow the differentiation of cockroach populations remains relevant.

Recently, the 2-Gb genome of *B. germanica* was reported [43], which gives rise to the possibility of studying the features of the structural and functional organization of TEs of this insect. However, it should be noted that, based on data presented in a public database (https://www.ncbi.nlm.nih.gov/bioproject/PRJNA203136), during the assembly and scaffolding of newly defined raw sequence data, nucleotide sequences corresponding to the repeats of ribosomal DNA (rDNA) and retrotransposons integrated into rDNA repeats remain undefined.

In this study, the complete rDNA repeat sequence and a full-size copy of the R2 retrotransposon of *B. germanica* were described. A partial sequence of a R2 retrotransposon of the cockroach *R. maderae* was also analyzed. The domain organization of the proteins encoded by these TEs was described. An analysis of the patterns of 5′-truncated copies of the *B. germanica* R2 retrotransposon revealed in cockroaches collected from eight different pig farms suggested the possibility of recent transposition activity. It was shown that the pattern of the 5′-truncated copies of these retrotransposons characteristic of each individual could be considered an informative molecular genetic marker (fingerprint) that allows the analysis of the structure of *B. germanica* populations.

A BLAST search of the *B. germanica* genome database to identify TEs closely related to the R2 retrotransposon revealed a new type of retrotransposon randomly located within the genome. Phylogenetic analysis showed that these first-described R2-like retrotransposons form a separate clade closely related to the R2 superclade. It was shown that the proteins encoded by the described non-site-specific R2-like retrotransposons have a unique structural organization, and a domain that is characteristic only of the described group of TEs was revealed. Moreover, within the described R2-like retrotransposons of *B. germanica*, a recombination event was shown.

## 2. Materials and Methods

### 2.1. DNA Isolation, Library Construction, and TE Amplification/Cloning/Sequencing

Total cockroach (*B. germanica* or *R. maderae*) DNA was isolated from whole individuals by homogenization in extraction buffer followed by phenol-chloroform extraction and ethanol precipitation according to standard protocols [44].

Cosmid libraries of *B. germanica* DNA were constructed in the SuperCos I cosmid vector (Stratagene, San Diego, CA, USA). Cosmid clones containing R2 retrotransposon copies were identified by colony hybridization using previously described 5′-truncated copies of the R2 retrotransposon [45] as probes. From this library, three clones containing full-size R2 retrotransposon DNA copies were confirmed by Southern hybridization. Extensive restriction mapping showed complete consistency between the arrangement of DNA in the cosmids that were detected in the genome by Southern blotting [44].

Polymerase chain reaction (PCR) was carried out in a Primus-25 thermal cycler (MWG-Biotech, Huntsville, AL, USA). We used the following primers for PCR amplification: (1) for cockroach R2 retrotransposons: forward 28S_R2_first (gtgctgacgcaatgtgatttc) [23] and reverse 28S_R2_second (cgttaatccattcatgcgcg); (2) for the 5′ fragments of BLAG 1–BLAG 6 retrotransposons, including their upstream genomic environment: forward BLAG 1 (cgggactggtcataacaaggcat), BLAG 2 (acctaacgaaggcataatacacaggtc), BLAG 3 (agtggcatgttgtcaaatagttctct), BLAG 4 (agtggtggccatctgcattga), BLAG 5 (aggaccatacaaatggctgcga), BLAG 6 (ctgtactacttactctcagcagttgtg), and reverse BLAG 1–BLAG 6 (ctcacacgctgaagtaggcctatc); and (3) for internal fragments of the BLAG 2 and BLAG 6 retrotransposons: forward BLAG 2/BLAG 6 (gatcagtgawggtgagtgctgtg) and reverse BLAG 2 (catcaaggagcggctgggac), BLAG 6 (gaagcattggcataacgttcggt). For each of the primer groups, the following annealing temperatures and durations of DNA strand synthesis were used: (1) 53 °C/3 min, (2) 58 °C/1 min, and (3) 57 °C/1 min. The amplification of DNA fragments was carried out in a volume of 50 μL using an Encyclo Plus PCR kit (Evrogen, Russia); 0.1 μg of genomic DNA and 2 μM of each primer were added to each reaction. The PCR program was as follows: preliminary heating at 95 °C for 4 min, 30 cycles of 94 °C for 1 min, annealing at a temperature depending on the primer set (53 °C, 58 °C, or 57 °C) for 2 min, and elongation at 72 °C for 1 min or 3 min (depending on the size of the amplified fragment), and a final 7 min elongation cycle at 72 °C.

The following pairs of evolutionary conservative primers were used for the amplification of overlapping fragments of native rDNA repeat of *B. germanica*: DAMS_18 (gtccctgccctttgtacaca) and DAMS_28 (ctactagatggttcgattagtc); NTS_18 (tccaccaactaagaacggcc) and NTS_28 (aactatgactctcttaaggt). The PCR program was as follows: preliminary heating at 95 °C for 4 min, 30 cycles of 94 °C for 1 min, annealing at 53 °C for 1 min, and elongation at 72 °C for 3 min, and a final 7 min elongation cycle at 72 °C. To clone the amplified DNA fragments, the corresponding PCR products were separated in 0.7% low-melting agarose gels (Fisher Scientific, Waltham, MA, USA). After electrophoresis, the DNA fragments were eluted from the gels using a Wizard PCR Preps DNA purification system (Promega, Madison, WI, USA) according to the manufacturer’s recommendations. The purified PCR fragments were used for ligation. Cloning was carried out using the pGEM-T-Easy vector and a corresponding reagent kit (Promega, USA).

The sequencing of the cloned DNA fragments was performed according to Sanger [46]. We used an ABI PRIZM 310 sequencer and a BigDye Termination kit v.3.1 as recommended by Applied Biosystems.

### 2.2. Identification of Sequences of Retrotransposons with Maximum Similarity to the R2 Retrotransposon of B. germanica

The local BLAST 2.9.0+ tool from the BLAST 2.9.0 package for 64-bit Linux (https://www.ncbi.nlm.nih.gov) [47] was used to perform searches with the default parameters, followed by the analysis and filtering of the output table. The command line used was as follows: “tblastn -query R2_Ribo_ORF.fa -db PYGN01 -out PYGN01_R2_Ribo_ORF_blasted.csv -outfmt ‘6 sseqid sstart send qstart qend length mismatch gapopen gaps pident evalue bitscore score qseq sseq”, where R2_Ribo_ORF.fa is a FASTA file containing the amino acid sequence corresponding to the R2 retrotransposon of *B. germanica*, and “PYGN01” is a database of the *B. germanica* genome (https://www.ncbi.nlm.nih.gov/Traces/wgs/PYGN01).

### 2.3. Domain Architecture Analysis

The general domain architecture of the proteins encoded by the ORFs of the retrotransposons was analyzed using the online Simple Modular Architecture Research Tool (SMART) (http://smart.embl-heidelberg.de/smart/set_mode.cgi?GENOMIC=1) [48].

Homology, HMM-HMM comparisons, and three-dimensional (3D) protein structure predictions were analyzed using the online Protein Homology/analogY Recognition Engine V2.0 (PHYRE-2) [49] (http://www.sbg.bio.ic.ac.uk/~phyre2/html/page.cgi?id=index) and HHpred [50,51] (https://toolkit.tuebingen.mpg.de/tools/hhpred).

### 2.4. Recombination Analysis

Recombination analysis was carried out with different algorithms implemented in the Recombination Detection Program v.4.43 (RDP4) [52], including RDP, GENECONV, Chimaera, MaxChi, BootScan, 3Seq, and SiScan, with their default settings.

### 2.5. Conserved Motif Analyses

Online MEME Suite 4.11.0 (http://meme-suite.org/) was used for conserved motif analyses [53]. MEME software (http://meme-suite.org/tools/meme) was used as a powerful, comprehensive web-based tool for mining sequence motifs in proteins. The maximum motif width, minimal motif width, and maximum number of motifs were set to 300, 20, and 10, respectively. Next, FIMO (find individual motif occurrences) software (http://meme-suite.org/tools/fimo) with default settings was used for searching a set of sequences with the occurrences of known motifs, treating each motif independently [54]. For FIMO analysis, a local database consisting of the protein sequences corresponding to the ORFs of the TEs used for phylogenetic analysis was created (see list below).

### 2.6. Sequence Alignment and Phylogenetic Analysis

Most of the sequences of known TEs used for phylogenetic analysis were obtained from Repbase [55] (http://www.girinst.org/repbase). The corresponding accession numbers are as follows: *R2NS-1_CGi, R2Amar, R2Ps, R2Sm-A, R2Ci, R2A_NVi, R2_BM, R2-7_MR, R2-8_MR, R2-1_PBa, R2-2_TCas, R2-1_PPap, RaR2, R2-1_MDe, R2Nvec-A, R2Ci-B, R2-1_TCas, R2-1_MR, R2-4_MR, R2Dr, R2NS-1_SMed, R2NS-1_CSi, R2B_NVi, R2-1_BTe, R2Amel, R2-2_MR, R2-1_GFo, R2-1_ZA, R2-1_TG, R2-1_Gav, R2Ol-A, R2-1_SSa, R2-1_GA, R2B_DM, R2_DPe, R2_Dan, R2_DSi, R2C_NGi, R2-5_MR, R2_FA, R2_RU, R2_RL, R2_KF, R8Hm-A, R8Hm-B* (R2 Super-clade); *NeSL-1_TV, Utopia-1_ENe, Utopia-1B_CPB, Utopia-1_Aca, Utopia-1_AEc, Utopia-1_CFl, Utopia-1_CMy, Utopia-1_LV, R5-2_SM, LIN9_SM, R5, R5-1_SM* (NeSL clade); *HERO-1_SP, HERO-1_HR, HERO-3_BF, HERO-1_BF, HEROTn*, (HERO clade); *R4_AL, R4_Hmel, R4-5_BX, R4-4_SRa, R4-2_AS* (R4 clade); *SLACS, CRE1, CRE2* (CRE clade).

Additionally, to build a phylogenetic tree, the sequences first described in this study were used, including the BLAG retrotransposons and R2 retrotransposons of *B. germanica* and *R. maderae*. Additionally, the following sequences with GenBank Acc. ## WP_053413546 (group II intron reverse transcriptase/maturase of *Geobacillus stearothermophilus*) and GAU97528 (reverse transcriptase of *Ramazzottius varieornatus*) were used.

For the multiple alignment of the analyzed retrotransposon sequences, we used PROMALS3D (profile multiple alignment with predicted local structures and 3D constraints) software [56] (http://prodata.swmed.edu/promals3d/promals3d.php), with the group II intron reverse transcriptase sequence (PDB Acc. # 6AR1) uploaded as a structure file.

The evolutionary history was inferred by using the maximum likelihood method and the Le_Gascuel_2008 model [57]. The initial trees used for the heuristic search were obtained automatically by applying the Neighbor-Join and BioNJ algorithms to a matrix of pairwise distances estimated using a JTT model and then selecting the topology with superior log likelihood value. A discrete Gamma distribution was used to model evolutionary rate differences among sites (10 categories (+*G*, parameter = 1.3312)). The rate variation model allowed some sites to be evolutionarily invariable ([+*I*], 0.69% sites). Evolutionary analyses were conducted in MEGA X [58].

### 2.7. R2 Retrotransposon 5′-truncated Fragments Analysis

The collection of *B. germanica* was carried out on eight pig farms located in the United States (NC). On each pig farm, 50–70 individuals were collected. Only males were used for this analysis. Fresh-collected insects were fixed in 96% ethanol and stored in a freezer at −20 °C until the start of molecular genetic analysis.

Total DNA was isolated from whole individuals as described above. To amplify the 5′-truncated copies of R2 retrotransposons, the following pair of primers was used: 28S_R2_first (gtgctgacgcaatgtgatttc), located within the rDNA flanking the 5′-end of the integrated copies of the R2 retrotransposon, and R2_rev_1 (gtcaaggtagtccttcagas), located within the R2 retrotransposon. The amplification of DNA fragments was carried out in a volume of 50 μL using an Encyclo Plus PCR kit (Evrogen, Russia); 0.1 μg of genomic DNA and a 2 μM concentration of each primer were added to each reaction. The PCR program was as follows: preliminary heating (95 °C, 4 min), 30 cycles of 94 °C for 0.5 min, 60 °C for 0.5 min, and 72 °C for 0.5 min, and a final 3 min elongation cycle at 72 °C.

The amplification products were analyzed by electrophoresis in 10% polyacrylamide gels in Tris-borate electrode buffer, followed by gel staining with 0.2% ethidium bromide solution [44].

### 2.8. Bayesian Clustering and Principal Coordinate Analysis

STRUCTURE 2.3.4. software [59] was used to carry out model-based clustering analysis and to assign individuals to populations as described by Martínez et al. (2012) [60]. We tested four models differing in admixture and allele frequency parameters (admixture or no admixture, correlated or independent allele frequencies among populations). For each ancestral K value, we performed 20 independent simulations, from *K* = 1 to *K* = 8, using a burn-in of 500,000 iterations and a run length of 1,000,000 iterations. The method of Pritchard et al. (2000) [59] was used to determine the modal distribution of the estimated log probability of the data Pr(X|K) for each value of K for the eight cockroach populations.

Principal coordinate analysis of the eight cockroach populations based on Nei’s genetic distance [61] with data standardization was carried out with GenAlEx v.6.5. software [62].

### 2.9. Data Deposition

Sequences have been deposited at GenBank under the accessions ## AF005243, EF014490, MT832838.

## 3. Results and Discussion

### 3.1. Complete rDNA Repeat Sequence of B. germanica

The cluster of the ribosomal RNA genes (ribosomal DNA) of *B. germanica* has been the subject of our research for a long time. Our previous publications described the structure and features of the evolutionary variability of various structural elements of rDNA repeats, including transcribed spacers (ITS1 and ITS2), non-transcribed spacers, and rRNA gene fragments [63,64,65]. Universal primers have been proposed for the amplification of overlapping fragments of rDNA repeats and, thus, for determining the structure of native rDNA repeats. In this study, we first describe the complete rDNA repeat sequence of *B. germanica.* This sequence has been deposited in GenBank under accession number AF005243.

**Figure 1:**
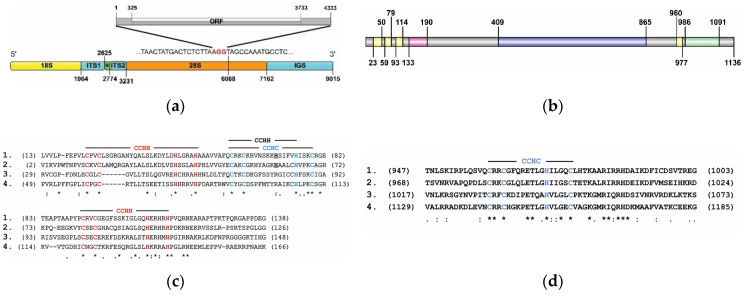
Structural organization of the R2 retrotransposon of *Blattella germanica*. (**a**) Schematic representation of the structure of the cluster of ribosomal RNA genes with the integration site of the R2 retrotransposon indicated. Corresponding structural elements are highlighted in blocks of different colors. The asterisk denotes the 5.8S RNA gene. The numbers indicate the beginning and end of the corresponding structural units. Three nucleotides were absent in the 28S sequence, suggesting that they were deleted during the integration process. These nucleotides are indicated in red. Gray blocks show the open reading frame (ORF) and 5′-, 3′-untranslated regions of the R2 retrotransposon; (**b**) Protein domain organization corresponding to the ORF of the R2 retrotransposon of *B. germanica*. The yellow blocks are zinc-finger domains, the crimson block is the c-myb domain, the blue block is the reverse transcriptase domain, and the green block is the endonuclease-like domain. The numbers indicate the positions of the beginning and the end of the corresponding domain. The alignment of the (**c**) N-terminal and (**d**) C-terminal zinc-finger motifs: **1**—*B. germanica*, **2**—*R. maderae*, **3**—*Reticulitermes* *lucifugus*, **4**—*Kalotermes flavicollis*. The CCHH type of the zinc-finger motifs is shown in red font and the CCHC type in blue. The CCHH type of the zinc-finger motif is indicated in black (explained in the text).

Figure 1a shows a schematic representation of the complete rDNA repeat sequence of *B. germanica*. The rRNA genes have the following sizes: 18S—1964 bp, 5.8S—149 bp, 28S—3931 bp. The sizes of the internal transcribed spacers (ITS1 and ITS2) are 661 bp and 457 bp, respectively. Intergenic spacer (IGS), including non-transcribed spacer (NGS) and external transcribed spacer (ETS), cover 1853 bp. The transcribed repeat units of the *B. germanica* rDNA cluster are separated by NGSs exhibiting different types of subrepeats and, accordingly, different lengths. In Figure 1a, the smallest IGS variant is shown.

The proteins encoded by R2 retrotransposons recognize and cut evolutionarily conserved sequences, providing the integration of TEs into a strictly defined rDNA region. The site for the cutting the rDNA sequence of *B. germanica* during the integration of the R2 retrotransposon is located between nucleotides 2856 and 2857 of the 28S RNA gene (Figure 1a).

### 3.2. Structural and Functional Organization of Full-Length Copies of the R2 Retrotransposon of B. germanica

During our previous studies, we described the integration features of 5′-truncated copies of the *B. germanica* R2 retrotransposon [45]. To identify full-length copies, we constructed a *B. germanica* gene library based on the SuperCos I cosmid vector (Stratagene, USA), which is able to stably maintain genomic DNA inserts of up to 40 kb in length. This library was screened by using probes corresponding to the 5′-truncated copies of the R2 retrotransposon, and full-size copies of this TE were revealed.

We determined the nucleotide sequences of three clones containing full-length R2 retrotransposons. The cloned copies of the R2 retrotransposon exhibited similar lengths and shared approximately 99.9% nucleotide sequence similarity. The consensus sequence has been deposited in GenBank under the accession numbers EF014490. It is known that in the process of R2 retrotransposon integration the top-strand cleavage occurs with a shift of several nucleotides relative to the first nick, which leads to a short deletion of the target site. In this paper, we have shown that in the case of integration of the R2 retrotransposon of *B. germanica*, three nucleotides are deleted, as indicated in Figure 1a in red font.

The full length of the R2 retrotransposon of *B. germanica* is 4333 bp, 3408 bp of which falls within a single open reading frame (ORF), while 325 bp and 600 bp falls within 5′- and 3′-untranslated areas, respectively. The sequence of the described retrotransposon with the flanking rDNA sequences is shown in Figure S1a.

The main objective of this study was to investigate the R2 retrotransposons of the German cockroach, *B. germanica*, but to study the structural and functional organization of this type of TE, we also analyzed the structure of the R2 retrotransposon of a closely related species, *R. maderae*. A long 5′-truncated copy of a R2 retrotransposon of the cockroach *R. maderae* containing the native ORF was described. This sequence has been deposited in GenBank under the accession numbers MT832838.

The total length of the protein corresponding to the ORF of the R2 retrotransposon of *B. germanica* is 1136 aa. We used the new, highly sensitive method of HMM-HMM comparison for the identification of protein similarity and structure prediction. For this purpose, we applied HHpred and PHYRE2 software, which allowed us to detect the PDB hits showing the maximum similarity to the analyzed retrotransposon protein. Using this approach, the boundaries of the c-myb motif, reverse transcriptase domain, and endonuclease-like domain were defined. Next, we used two approaches to identify Zn-finger domains: analysis with a simple modular architecture research tool (SMART) and the alignment of amino acid sequences of the proteins encoded by the R2 retrotransposons of the closely related species. The domain organization of the protein corresponding to the ORF of the R2 retrotransposon of *B. germanica* is shown in Figure 1b and Figure S1b–f.

The analysis of domain architecture using SMART software revealed three zinc-finger domains (aa 23–50; aa 59–79; aa 93–114) (Figure 1b and Figure S1c). Figure 1c shows the alignment of the amino acid sequence fragment of the *B. germanica* R2 retrotransposon containing the zinc-finger domains with the corresponding amino acid sequences of three closely related insect species: the cockroach *R. maderae* (the sequence described in this study) and the termites *Reticulitermes lucifugus* (GenBank Acc. #ADX60045) and *Kalotermes flavicollis* (GenBank Acc. #ADX60048). It was shown that the first and third zinc-finger domains of all compared R2 retrotransposons have a typical CCHH structure (the corresponding amino acids are highlighted in red), while the second zinc-finger domain of the cockroach R2 retrotransposons can be assigned to CCHC and/or CCHH structures. In Figure 1c, the amino acids that form the CCHC motif are indicated in blue font, and the amino acids that are part of the potential CCHH motif are indicated in bold and underlined. All of the retrotransposons described so far that have three zinc finger domains at the N-terminus of the polypeptide sequence are characterized by a CCHC structure in that the second of these three domains [15]. However, the determination of whether the dual structure of the second zinc finger domain of the cockroach R2 retrotransposons has functional significance will require future experimental verification.

All of the R2 retrotransposons of different living organisms described to date contain an additional zinc-finger domain located between the reverse transcriptase and endonuclease-like domains. The alignment of the polypeptide sequence fragments corresponding to the ORFs of the R2 retrotransposons of the two cockroach species described in this study and the termites studied previously [29] showed that within the protein sequence of the R2 retrotransposon of *B. germanica,* this domain is located between 960 and 977 aa (Figure 1b,d). Similar to all R2 retrotransposons described to date, this zinc-finger domain has a CCHC structure. In Figure 1d, the corresponding amino acids are indicated in blue font.

The C-myb domain of the R2 retrotransposon of *B. germanica* is located between 133 and 190 aa (Figure 1b). HMM–HMM comparison revealed the maximum similarity of the c-myb domain of this retrotransposon with one of the hypothetical mouse gene (2610100B20Rik) products; the HHpred alignment of this gene product homologous to the Myb DNA-binding domain (PDB accession number: 1UG2_A) is shown in Figure S1d.

The central portion (from 409 to 865 aa) of the protein product of the R2 retrotransposon of *B. germanica* corresponds to the reverse transcriptase domain (Figure 1b). The domain boundaries were determined according to protein homology detection by the HMM–HMM comparison method. The first six PDB hits obtained using HHsearch were for the following proteins with known structures: bacterial group II introns (PDB acc. ##: 6AR1, 5G2X, 6MEC, 5HHJ) and telomerase reverse transcriptase of *Tetrahymena thermophila* (PDB acc. # 6D6V) and *Tribolium castaneum* (PDB acc. # 5CQG). The maximum similarity was revealed between the analyzed domain of the *B. germanica* R2 retrotransposon and the Group II intron reverse transcriptase of *G. stearothermophilus* (PDB accession # 6AR1); the HHpred alignment is shown in Figure S1e. It is known that the domains of the reverse transcriptase of non-LTR retrotransposons, group II introns, and telomerase reverse transcriptase are evolutionarily closely related protein sequences [66,67].

In the study of the endonuclease-like domain of the *B. germanica* R2 retrotransposon as well as the analysis of the c-myb and reverse transcriptase domains, for similarity detection and structure prediction via HMM-HMM comparison, we applied HHpred software, which was used for the detection of HHsearch PDB hits. The maximum similarity was revealed between the analyzed domain of the *B. germanica* R2 retrotransposon and the following proteins with known structures related to Cap-snatching endonucleases and Holliday junction resolvases (Figure S1f). We previously conducted a detailed study of the structural organization of the endonuclease-like domain of R2 retrotransposons and showed the structural similarity of this domain to bacterial Holliday junction resolvases. Based on the identified similarities, a new mechanism was proposed that determines the transposition of this class of mobile elements [68]. In Figure 1b, the boundaries of the endonuclease-like domain of the *B. germanica* R2 retrotransposon are indicated according to structural similarities with the domain Holliday junction resolvase (Hjc) from *Pyrococcus furiosus* (PDB accession # 1GEF). The identified similarity of the 3D structures of the R2 retrotransposons and Cap-snatching endonucleases is unexpected from our point of view and requires additional analysis.

### 3.3. Detection and Analysis of the Non-Site-Specific R2-like Retrotransposon Distribution in the Genome of B. germanica

To identify the retrotransposons that are the most closely related to R2 retrotransposons, we performed a BLAST (TBLASTN) search of the complete *B. germanica* genome database using the protein sequence corresponding to the ORF of this TE as a query. Six mobile elements closely related to R2 retrotransposons were revealed. These newly described TEs were designated BLAG 1–BLAG 6 based on the name of the organism whose genome in which they were discovered (**Bla**ttella **g**ermanica). Next, it was shown that each of the BLAG retrotransposons is represented in the genome by several copies, including 6, 3, 4, 4, 3, and 3 copies of BLAG 1–BLAG 6, respectively. The consensus sequences of each newly described retrotransposon have been deposited in GenBank and are presented in Figure S2.

The repeated copies of the BLAG 1–BLAG 6 retrotransposons in the genomes have a structure similar to that described above for R2 retrotransposons, including one open reading frame and 5′- and 3′-untranslated regions. The variability in lengths of the corresponding structural elements of the BLAG 1–BLAG 6 retrotransposons is as follows: full length: 3215–3669 bp, ORF: 2721–3219 bp, 5′-untranslated area: −37–436 bp, 3′-untranslated area: 132–241 bp (Figure S2).

The alignment of the fragments of 5′- and 3′-untranslated areas together with extended regions of the genomic environment of all identified copies of BLAG 1–BLAG 6 retrotransposons is shown in Figure S3.

It was shown that each of the described copies of BLAG 1–BLAG 6 retrotransposons exhibits a unique genomic environment of 3′-untranslated area. This finding means that the described type of TE belongs to site-non-specific retrotransposons. Moreover, the genomic environment of the 5′-untranslated region of the studied TEs exhibits a number of interesting features.

Each copy of the BLAG 3–BLAG 5 retrotransposons presents a unique genomic environment. The following features are characteristic of BLAG 2 and BLAG 6 retrotransposons: two out of three copies of each of these TEs exhibit a similar genomic environment of the 5′-untranslated region that differs from the genomic environment of the third copy. The simplest interpretation of this observation is as follows: the third (shorter) copy is a 5′-truncated copy obtained from the full-size copies represented by two other (longer) structural variants.

The analysis of the environment of the 5′-untranslated regions of six different copies of the BLAG 1 retrotransposon provided much more information about the transposition features of this type of TE.

Before discussing the environmental features of the 5′-untranslated regions of the BLAG 1 retrotransposon, we ensured that the revealed nucleotide sequences were not a result of the incorrect assembly of the short reads during extended contig generation *in silico*.

**Figure 2:**
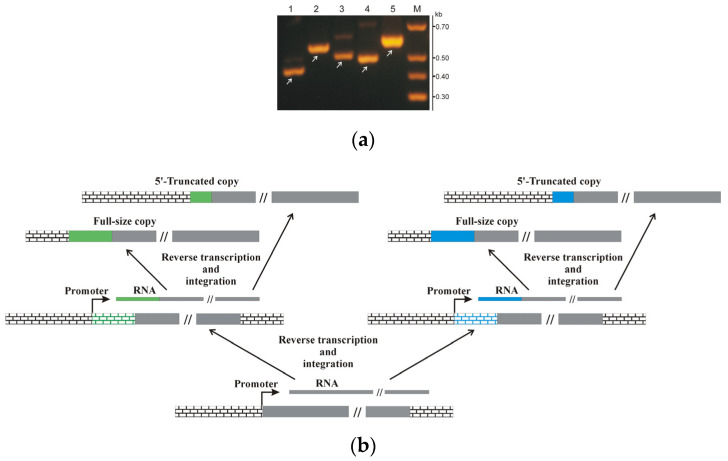
Analysis of the distribution of BLAG retrotransposons of *B. germanica* in the genome. (**a**) The result of the electrophoretic separation of the amplification products obtained using primers in a 1% agarose gel, one of which is located in the 5′-untranslated region of the BLAG 1 retrotransposon, while the second is located in the genomic environment of **1**–BLAG 1_2, **2**–BLAG 1_3, **3**–BLAG 1_4, **4**–BLAG 1_5, and **5**–BLAG 1_6. Arrows indicate the amplification products used for cloning and sequencing. (**b**) Hypothetical scheme of the transposition of BLAG retrotransposons. cDNA copies of a particular ancestral form of the BLAG retrotransposon (grey) are integrated into two different sites located next to the promoters of random genes of the host organism. The nucleotide sequences located between the promoter and randomly integrated copies of a particular ancestral form of the BLAG retrotransposon are shown by a line similar to brickwork of varying lengths in green or blue color, depending on the integration site. Solid green and blue lines correspond to newly transcribed retrotransposon variants and their genome-integrated forms.

For this purpose, seven primers were synthesized, one of which was located in the region of maximum sequence similarity among all copies of the BLAG 1 retrotransposon (inside the retrotransposon body), and six others were located within unique parts of the environment of the 5′-end of the analyzed BLAG 1 copies. The primer locations and the corresponding nucleotide sequences are presented in Figure S3 (underlined). The result of the PCR amplification of the analyzed fragments using the corresponding pairs of primers and the total DNA of *B. germanica* is shown in Figure 2a. We were not able to amplify the DNA fragment corresponding to the copy of the BLAG 1 retrotransposon designated in Figure S3 as Blag 1_01. However, the corresponding fragments of all other copies of the BLAG 1 retrotransposon (Blag 1_02–Blag 1_06) were successfully amplified.

The amplified DNA fragments were of similar size to the expected fragment: 428 bp for BLAG 1_02, 555 bp for BLAG 1_03, 501 bp for BLAG 1_04, 482 bp for BLAG 1_05, and 571 bp for BLAG 1_06 (Figure 2a). In the next step, the amplified fragments were cloned into the pGEM-T vector (Promega) and sequenced using M13 primers. The complete identity of the analyzed sequences to the sequences present in the GenBank database is shown. The absence of the amplification product in our experiments using these primers, one of which was complementary to the DNA sequence of the environment of the BLAG 1_1 integration site, can be explained by the polymorphism of the integration sites of this type of TE. The range of the integration sites of the BLAG 1 retrotransposon in the cockroach individuals used for genome sequencing and those used in our experiments can vary.

The analysis of the genomic environment of the 5′-untranslated regions of the integrated copies of the BLAG 1 retrotransposon showed that all of the described integration sites could be subdivided into two groups: the first group includes copies of BLAG 1_1–BLAG 1_3, and the second group includes copies of BLAG 1_4–BLAG 1_6. Each of the described groups contains three copies of the BLAG 1 retrotransposon, and two out of the three copies of each group exhibit a similar genomic environment of the 5′-untranslated region differing in length from the genomic environment of the third copy (Figure 2a). The simplest interpretation of this observation is as follows: the third (shorter copy) is a 5′-truncated copy obtained from full-size copies represented by two other (longer) structural variants. A similar interpretation has been applied in our analysis of the structure of the variable sites of the integration of different copies of BLAG 2 and BLAG 6 retrotransposons (see above). However, the presence of two types of environment of the BLAG 1 retrotransposon with different nucleotide compositions between the retrotransposons forming the two described groups suggests the presence of an ancestral copy of the retrotransposon and, in our opinion, sheds light on the characteristics of the transposition activity of this type of TE.

The transcription of a native copy of the retrotransposon integrated into the genome is necessary for the transpositional activity of non-LTR retrotransposons. What is the possible transcription mechanism of BLAG retrotransposons?

We believe that BLAG retrotransposons do not contain internal promoters in their own composition and that transcription occurs from gene promoters belonging to the host organism if random integration occurs near a promoter. If the retrotransposon is inserted away from a promoter, this copy remains a “dead” replica, incapable of further transposition.

An illustration of this model is shown in Figure 2b. In this example, cDNA copies of a particular ancestral form of the BLAG retrotransposon are integrated into two different sites located next to the promoters of random genes of the host organism. At the next stage, the structure of the original copy of the retrotransposon will change since the transcripts transcribed from these two different promoters will exhibit different lengths and different nucleotide compositions of the 5′ environment of the original copy of the mobile element. The integration of these new copies into the genome will lead to the formation of two subfamilies of the original TE, similar to the different subfamilies of the BLAG 1 retrotransposon that we have identified, i.e., the subfamily including the BLAG 1_2 and BLAG 1_3 retrotransposons and the subfamily consisting of the BLAG 1_5 and BLAG 1_6 retrotransposons. The ability to integrate not only full-sized copies of retrotransposons but also their 5′-truncated copies into the genome contributes to increasing the diversity of the structural variants of the integrated copies of TEs.

For the different types of non-LTR retrotransposons described to date, transcriptional activity is mediated in different ways. R2 retrotransposons whose integration sites are within ribosomal RNA genes are transcribed as a component of ribosomal RNAs from the promoter of RNA polymerase I. In the next stage, due to self-cleaving ribozyme activity, the RNA of the R2 retrotransposon is excised from the extended co-transcript and then translated via an IRES-mediated mechanism [69]. Some types of site-specific retrotransposons exhibit unique integration sites located next to the promoters of genes of the host organism. For example, the NeSL-1 retrotransposon specifically inserts into spliced leader-1 genes. NeSL-1 leader sequences do not require their own promoters because they can be co-transcribed with the SL1 gene, and their RNA is thus immediately available for translation [70]. LINE-like non-LTR retrotransposons contain a unique promoter located in their 5′-untranslated region [71,72,73]. However, it should be noted that for many types of retrotransposons described to date, the molecular mechanisms conferring transcriptional and translational activity remain unclear.

### 3.4. Structural and Functional Organization of BLAG Retrotransposons

The total lengths of the proteins corresponding to the ORFs of the BLAG 1—BLAG 6 retrotransposons of *B. germanica* are 994 aa, 1073 aa, 984 aa, 974 aa, 988 aa, and 1059 aa, respectively (see Figure S2).

In the first step of domain prediction within the proteins corresponding to the BLAG ORFs, we used an approach similar to that described above for R2 retrotransposons.

**Figure 3:**
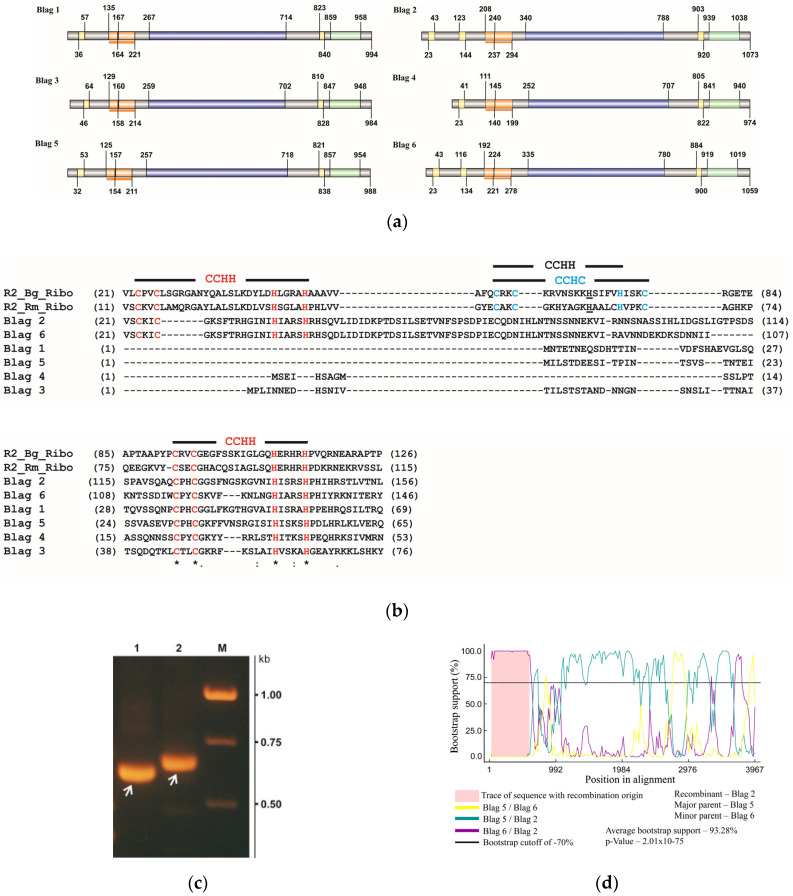
Structural organization of the BLAG retrotransposons of *B. germanica*. (**a**) Protein domain organization corresponding to the open reading frames (ORFs) of the BLAG retrotransposons of *B. germanica*. The yellow blocks are zinc-finger domains, the brown block is the BLAG domain, the blue block is the reverse transcriptase domain, and the green block is the endonuclease-like domain. The numbers indicate the positions of the beginning and the end of the corresponding domain. (**b**) The alignment of N-terminal zinc-finger motifs: R2_Bg_Ribo–R2 retrotransposon of *B. germanica*, R2_Rm_Ribo–R2 retrotransposon of *R. maderae*, Blag 1–Blag 6—the respective BLAG retrotransposons. The CCHH type of the zinc-finger motifs is shown in red font, and the CCHC type is shown in blue. The CCHH type of the zinc-finger motif is shown in black—see explanation in the text. (**c**) The result of 1% agarose gel electrophoresis to separate the amplification products obtained using a primer located in the region of maximum sequence similarity between the BLAG 2 and BLAG 6 retrotransposons and two other primers located within the unique parts of the sequences of these transposable elements (TEs). Tracks: **1**–BLAG 6, **2**–BLAG 2. Arrows indicate the amplification products used for cloning and sequencing. (**d**) BOOTSCAN plots of the recombination event detected by using RDP4 software. The bootstrap support for each pair of sequences is plotted on the *y*-axis, and their position in the alignment is plotted on the *x*-axis. The recombinant region is shown in pink. Symbols are listed in the figure.

The analysis of domain architecture using SMART software and pairwise alignment revealed one (BLAG 1, BLAG 3, BLAG 4, BLAG 5) or two (BLAG 2, BLAG 6) zinc-finger domains located in the N-terminal regions of the described proteins (Figure 3a). The alignment of the BLAG sequences with each other and with the corresponding sequences of the R2 retrotransposons of *B. germanica* and *R. maderae* shows that both zinc-finger domains of BLAG retrotransposons refer to the CCHH type and are similar to the first and third zinc-finger domains of the cockroach R2 retrotransposons described above (Figure 3b). Moreover, all BLAG retrotransposons contain an additional zinc-finger domain located close to the C-terminal regions of the corresponding proteins (Figure 3a). As for all R2 retrotransposons described to date, these zinc-finger domains exhibit a CCHC structure.

The pairwise alignment of nucleotide sequences of BLAG retrotransposons showed that the 5′-ends of the BLAG 2 and BLAG 6 retrotransposons consist of almost identical nucleotide sequences. The extended region of similarity includes a portion of the ORFs of these TEs. The corresponding alignment is presented in Figure S4. These findings may indicate a recombination event that plays a role in the evolutionary variability of this group of retrotransposons.

To prove the existence of this event, it was first necessary to verify that the revealed similarity of the nucleotide sequences was not a result of the erroneous assembly of the short reads during extended contig generation *in silico*. For this purpose, three primers were synthesized, one of which was located in the region of the maximum sequence similarity of the BLAG 2 and BLAG 6 retrotransposons, and two others were located within the unique portions of the sequences of these TEs. The primer locations are presented in Figure S4. The result of the PCR amplification of the analyzed fragments using the described pairs of primers and the total DNA of *B. germanica* is shown in Figure 3c. The amplified DNA fragments were of similar size to the expected fragments (655 b–BLAG 2, 607 b–BLAG 6). In the next step, the amplified fragments were cloned into the pGEM-T vector (Promega, USA) and sequenced using the M13 primers. The complete identity of the analyzed sequences to the sequences present in the GenBank database is shown.

Recombination analysis was carried out with different algorithms implemented in the Recombination Detection Program v.4.43 (RDP4). In Figure 3d, it can be seen that the sequences of BLAG 2 and BLAG 6 exhibit considerable similarity within the region identified as having a recombinant origin, whereas the sequence of BLAG 2 is more similar to the sequence of BLAG 5 within nonrecombinant regions. These facts suggest that BLAG 2 may be a recombinant retrotransposon that originated from a recombination event between the ancestors of BLAG 5 and BLAG 6. The average bootstrap support for this recombination event is 93.28%, *p*-value—2.01 × 10^−75^.

Since the recombination regions contain ORF fragments including zinc finger domains, it can be concluded that the variability of the number of zinc finger domains at the N-termini of the proteins corresponding to the ORFs of BLAG retrotransposons may be due to recombination between the DNA sequences of copies of these TEs integrated into the genome.

In the next step, the proteins corresponding to the ORFs of each BLAG retrotransposon (BLAG 1–BLAG 6) were analyzed with HHpred and PHYRE2 software, which allowed us to detect the PDB hits with the maximum similarity to the analyzed retrotransposon proteins. The boundaries of the reverse transcriptase and endonuclease-like domains were defined using this approach (Figure 3a).

Thus, the structural and functional organization of BLAG retrotransposons is similar to that described for R2 retrotransposons and can be characterized by the presence of the following evolutionarily conserved motifs: zinc-finger, reverse transcriptase, and endonuclease-like motifs. However, within the BLAG retrotransposons, we did not find the c-myb motifs described for all known R2 retrotransposons to date.

The c-myb motif within the protein sequence corresponding to R2 retrotransposons is located between Zn-finger and reverse transcriptase domains close to the N-terminus of the analyzed proteins. It became obvious that the main structural and, possibly, functional differences between the described R2 retrotransposons and BLAG retrotransposons were located between Zn-finger and reverse transcriptase domains. The fragments of BLAG 1—BLAG 6 retrotransposon polypeptide sequences corresponding to this region were the subject of our more detailed analysis.

Toward this end, we used Jalview Version 2, a multiple sequence alignment editor [74] (MAFFT multiple sequence alignment—L-INS-i option), for the generation of the consensus sequences corresponding to the analyzed regions of the BLAG 1–BLAG 6 retrotransposons (Figure S5). To identify the retrotransposons (proteins corresponding to the ORFs containing evolutionarily closely related sequences similar to those of BLAG retrotransposons), we conducted a PSI-BLAST (TBLASTN) search (https://blast.ncbi.nlm.nih.gov/Blast.cgi) for all nucleotide sequences present in the GenBank database using the BLAG 1–BLAG 6 consensus amino acid sequence as a query sequence.

This search allowed us to identify two extended fragments (length greater than 500 aa) of the retrotransposons, with GenBank Acc. ## CAB0007773 and GAU97528, in the genomes of *Nesidiocoris tenuis* (Insect, Hemiptera) and *R. varieornatus* (Tardigrada, water bear), respectively. We consider this an additional indication that the presence of specific evolutionarily conserved sequences may be a characteristic feature of the described new type of TEs.

**Figure 4:**
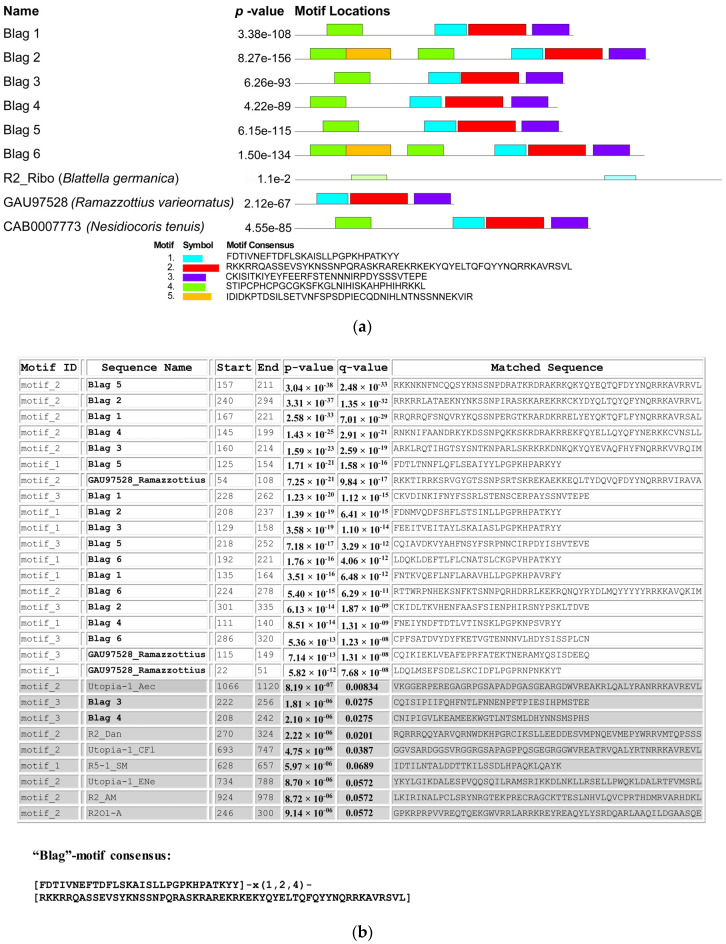
Identification and description of the “BLAG” domain. (**a**) Conserved protein motifs are identified using MEME software. Significantly represented motifs are graphically depicted by bars of different colors corresponding to their predicted position. The sequences included in the analysis are those of the BLAG 1–BLAG 6 retrotransposons, the R2 retrotransposon of *B. germanica* (R2_Ribo), and retrotransposons identified in the genomes of *N. tenuis* and *R. varieornatus* (GenBank Acc. ## CAB0007773 and GAU97528, respectively). The consensus sequence of each revealed motif is listed under the graphic. It was shown that motifs ## 1–3 are present in all analyzed sequences except for the R2 retrotransposon of *B. germanica*. (**b**) The result of FIMO software analysis. This program searches a set of sequences for occurrences of known motifs, treating each motif independently. Previously described motif ## 1–3 were analyzed using the database including the ORFs of the BLAG 1–BLAG 6 retrotransposons, the R2 retrotransposon of *B. germanica*, and the retrotransposon of *R. varieornatus* (GenBank Acc. # GAU97528) as well as TEs belonging to the R2, NeSL, HERO, and R4 clades; the list of the catalog numbers of the TEs used in this analysis is given in the Materials and Methods section. The fragment of a statistically insignificant zone is highlighted in gray (explained in the text). It was shown that only motifs #1 and #2 exhibit significant similarity to BLAG retrotransposons and may be considered a characteristic feature of this type of TE. The consensus “Blag” motif is shown as well.

Next, we applied motif distribution analysis using the MEME suite program (http://meme-suite.org/tools/meme) to identify the statistically significant motifs within the amino acid sequences corresponding to protein fragments located between the Zn-finger and reverse transcriptase domains of the BLAG 1 – BLAG 6 retrotransposons, the R2 retrotransposon of *B. germanica*, and the fragments of the retrotransposons identified in the genomes of *N. tenuis* and *R. varieornatus*. This analysis revealed a set of evolutionarily conserved motifs (Figure 4a), three of which were found in all analyzed sequences, with the exception of the sequence of the R2 retrotransposon of *B. germanica*. The described motifs #1 – #3 (Figure 4a) exhibit the following consensus sequences:

#1—FDTIVNEFTDFLSKAISLLPGPKHPATKYY,
#2—RKKRRQASSEVSYKNSSNPQRASKRAREKRKEKYQYELTQFQYYNQRRKAVRSVL,
#3—CKISITKIYEYFEERFSTENNNIRPDYSSSVTEPE.

To determine whether the presence of these three identified motifs is a characteristic feature of BLAG retrotransposons, we used FIMO (find individual motif occurrences) software. This program searches a set of sequences for occurrences of known motifs, treating each motif independently.

In the first step, a database was created, which consisted of the protein sequences corresponding to the ORFs of the TEs belonging to the following clades: R2, NeSL, HERO, and R4 (i.e., TEs that are evolutionarily closely related to each other and, as expected, to BLAG retrotransposons). The sequences of the known TEs were obtained from Repbase (http://www.girinst.org/repbase). Only TEs for which amino acid sequences corresponding to complete ORFs were determined were used in this analysis. The catalog numbers of the TEs used in this analysis are listed in the Materials and Methods section. In addition to these TEs, the created database included sequences corresponding to the ORFs of the BLAG 1–BLAG 6 retrotransposons, the R2 retrotransposon of *B. germanica*, and the above-described retrotransposon of *R. varieornatus*.

Each of the three motifs (#1, #2, and #3) presented in Figure 4a and described above was individually compared with the sequences in the created database, and the probability (*p*-value and *q*-value) that the analyzed motifs matched the TE sequences was determined. The probability that any of the analyzed motifs matched the TEs belonging to one of the R2, NeSl, HERO, and R4 clades was not considered significant. Accordingly, if the probability of any of the three analyzed motifs matching the BLAG retrotransposon of *B. germanica* or the retrotransposon described above in the *R. varieornatus* genome was less than significant, that motif could not be regarded as a characteristic feature of BLAG retrotransposons. The results of this analysis are presented in Figure 4b.

It was shown that only motifs #1 and #2 exhibit significant similarity to BLAG retrotransposons (see Figure 4b) and may be considered a characteristic feature of this type of TE. We believe that the “Blag”-motif consensus with the following structure [FDTIVNEFTDFLSKAISLLPGPKHPATKYY]-x(1,2,4)-[RKKRRQASSEVSYKNSSNPQRASKRAREKRKEKYQYELTQFQYYNQRRKAVRSVL], consisting of motifs #1 and #2, can be considered a new structural and, possibly, functional domain characteristic of the first described group of TEs (BLAG retrotransposons). In Figure 3a, the positions of these bipartite domains within protein sequences corresponding to the ORFs of the BLAG 1–BLAG 6 retrotransposons are indicated by brown boxes and underlining.

### 3.5. Retrotransposon Phylogeny

To determine the phylogenetic positions of the newly described R2 retrotransposons of *B. germanica* and *R. maderae* as well as the BLAG retrotransposons, we carried out a phylogenetic analysis of these TEs in combination with the previously described non-LTR retrotransposons, which belong to different phylogenetic clades: the CRE, R4, HERO, NeSL, and R2 superclades. To build a phylogenetic tree, a comparative analysis of fragments of the reverse transcriptase amino acid sequences was conducted.

The RT domain boundaries were determined according to protein homology detection via the HMM–HMM comparison method as described above. For all compared TEs, the maximum similarity was revealed with the Group II intron reverse transcriptase of *G. stearothermophilus* (PDB accession # 6AR1).

It was previously shown that the Group II intron–encoded RT domain is the most suitable sequence for use as an outgroup for the phylogenetic analysis of retrotransposons [70]. The recently described 3D structure of the Group II intron reverse transcriptase allows the simultaneous alignment of the amino acid sequences based on the three-dimensional folding of proteins, which can in our view significantly improve the alignment quality and consequently increase the resolution of the phylogenetic analysis.

For the multiple alignment of the 86 retrotransposon sequences, we used PROMALS3D, with 6AR1 uploaded as a structure file. The resulting alignments in FASTA format, with the designation of the predicted secondary structures, are shown in Figure S6. The length of the analyzed sequences ranged from 359 to 415 amino acids depending on the type of TE. The maximum structural similarity between the analyzed retrotransposons and the reverse transcriptase domain of the Group II intron was observed close to the N-termini. For this reason, retrotransposons for which the sequences that were too short (5′-truncated copies) were annotated could not be used in our study, since they did not contain the sequences corresponding to the N-terminus of the corresponding proteins, such as retrotransposons R2Eb and R2Pc, belonging to clade R2D3, retrotransposon R2-1_TSP, belonging to clade R2A1, and some others.

**Figure 5:**
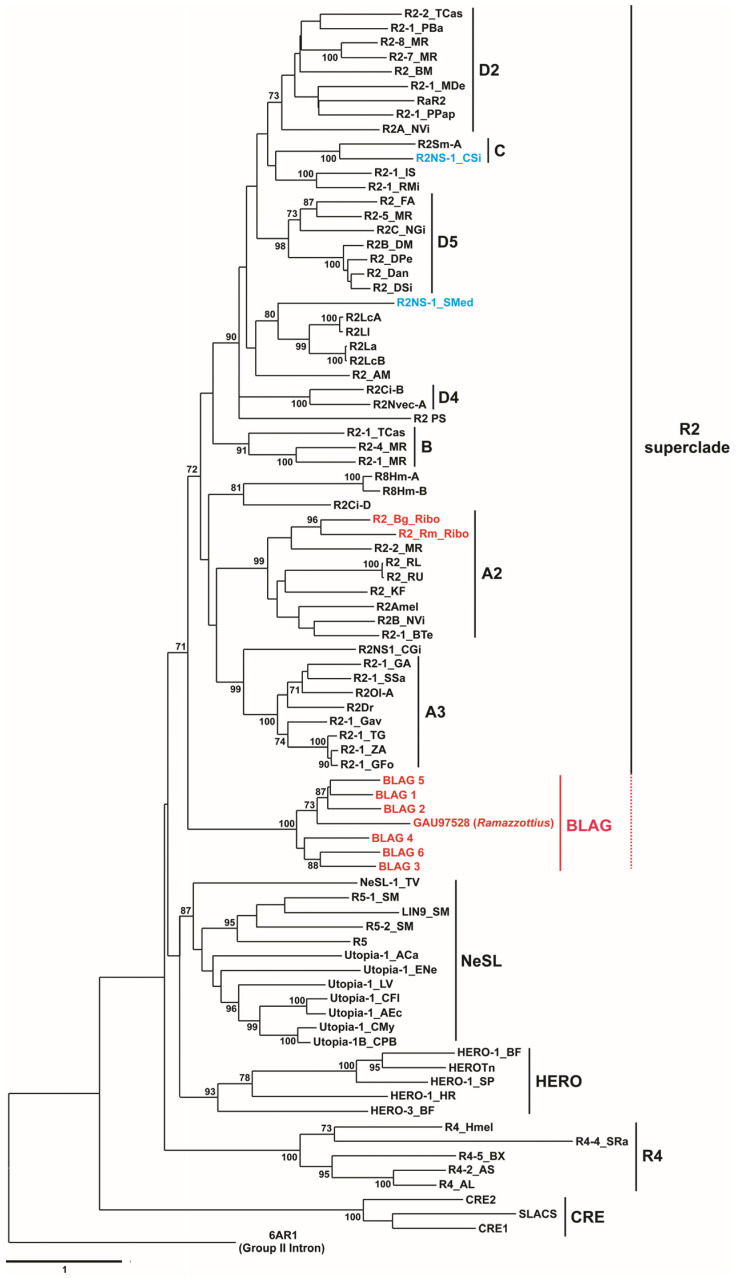
Evolutionary analysis of the described retrotransposons by the maximum likelihood method. The evolutionary history was inferred by using the maximum likelihood method and the Le_Gascuel_2008 model [57]. The tree with the highest log likelihood (−58,826.48) is shown. The percentage of trees in which the associated taxa clustered together is shown next to the branches (bootstrap values > 70% are shown). The tree is drawn to scale, with branch lengths measured as the number of substitutions per site. This analysis involved 86 amino acid sequences. The tree was rooted in the 6AR1 (Group II intron) sequence. There was a total of 518 positions in the final dataset. Evolutionary analyses were conducted in MEGA X [58]. The sequences first described in this study are highlighted in red, and the sequences of R2 non-site-specific retrotransposons described earlier [12] are highlighted in blue. A–D refer to clades/subclades following Kojima and Fujiwara (2005) [11].

The tree with the highest log likelihood is shown in Figure 5. The topology of the obtained phylogenetic tree generally corresponds to the previously described evolutionary history of the analyzed retrotransposons (for example, [11,12,70,75]). In Figure 5, the percentage of trees in which the associated taxa clustered together is shown next to the branches. The individual clades form the previously described CRE, R4, HERO, and NeSL retrotransposons with high bootstrap rates. Within the R2 superclade, the four main supergroups (A–D) identified by Kojima and Fujiwara (2005) [11] are recognizable, as are their clades.

The R2 retrotransposons of *B. germanica* and *R. maderae* belonged to the R2A2 clade with bootstrap support of 99%. This result is entirely consistent with previously published data [29] obtained during the construction of a phylogenetic tree including the R2 retrotransposon of *B. germanica* based on a small RT domain fragment comprising the C-terminal region reported by our group at the same time [45].

The BLAG 1–BLAG 6 retrotransposons together with the retrotransposon (GenBank acc. # GAU97528) found in the genome of the water bear *R. varieornatus* form a separate clade (bootstrap level–100%). This clade has been referred to as the BLAG clade based on the names of most of the TEs that form this clade (see Figure 5).

Retrotransposons belonging to the R2 superclade were separated from other described TEs with bootstrap support equal to 72% and clustered together with the BLAG clade with bootstrap support equal to 71% (Figure 5). It was evident that the BLAG retrotransposons were most closely related to the retrotransposons of the R2 superclade and, from our point of view, could be attributed to this superclade.

### 3.6. R2 Retrotransposon Dynamics and Population Structure

R2 retrotransposons are inserted through a target-primed reverse transcription mechanism, and if the synthesis of the first strand of R2 is incomplete, a 5′-truncated copy that is still able to undergo insertion is produced. These truncated variants can be used to monitor R2 activity and its role in rDNA dynamics.

We previously described the structure of several 5′-truncated copies of the R2 retrotransposon of *B. germanica*, described options for the deletion of the integration site (ribosomal DNA) and analyzed the inheritance of various structural variants of 5′-truncated copies in a number of generations [76,77,78].

**Figure 6:**
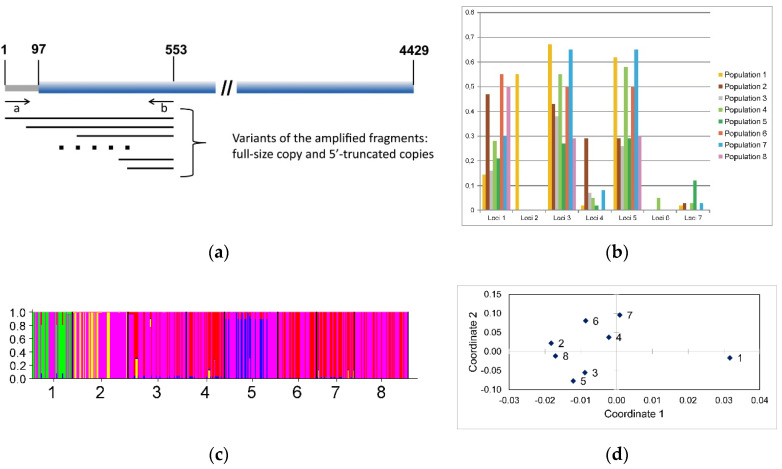
Analysis of the structural variants of R2 retrotransposon 5′-truncated copies aimed at assessing the transpositional activity of these TEs and the structure of *B. germanica* populations. (**a**) Schematic illustration of the structure of R2 retrotransposons, including the 5′-end environment. The position of the primers (**a**,**b**) used to identify the 5′-truncated copies of the R2 retrotransposon is indicated. The numbers indicate the positions of the corresponding nucleotides; (**b**) The frequency of the occurrence of the described 5′-truncated copy variants (Loci 1–7) in eight studied populations; (**c**) Bayesian genotypic cluster analysis based on the frequency of the occurrence of the described variants of 5′-truncated copies of the R2 retrotransposon of *B. germanica* in the eight populations together at *K* = 5. Each cluster is designated with a particular color (see Table 2). *x*-axis, samples collected in population ## 1–8; (**d**) Principal coordinate analysis (PCoA) of eight cockroach populations based on Nei’s genetic distances. PCoA plot in which the eight cockroach populations are plotted according to the eigenvectors corresponding to the first and second principal coordinates.

In this study, we conducted a detailed screen using a large number of individuals harboring various structural variants of 5′-truncated copies formed within a relatively short length of the R2 retrotransposon of *B. germanica*, 456 bp from the 5′-end of the complete size copy. Technically, this meant that the total DNA isolated from individuals of *B. germanica* and a pair of primers located within the rDNA upstream of the retrotransposon integration site and at a distance of 456 bp from the 5′-end of the native R2 retrotransposon were used (the position of the primers is schematically shown in Figure 6a) for the PCR amplification of the corresponding DNA fragments. Since the cluster of the ribosomal genes of *B. germanica* is located on the X chromosome and the sex of this insect is controlled by the number of X chromosomes (XX: female, XO: male) [79,80], only males were used in the study. Thus, each identified pattern of 5′-truncated copies corresponded to an individual X chromosome.

The amplification results were analyzed by ordinary acrylamide gel electrophoresis. Only fragments whose length corresponded to the range of 150–250 bp were used for statistical analysis. We identified seven different lengths of the analyzed DNA fragments. Figure S7 shows an example of the electrophoretic separation of the amplification products, in which gel fragments containing amplified fragments in the range of 150–250 bp are presented. A total of 393 individuals from eight different populations (pig farms located in NC, USA) were analyzed, including 42, 58, 61, 40, 56, 40, 40, and 56 individuals from pig farms ## 1–8, respectively.

The results of this study are summarized in Table 1. The frequency of the occurrence of different variants of the analyzed 5′-truncated copies is shown in Figure 6b. Some of the loci (1, 3, and 5) occurred at a high frequency in all populations (Table 1 and Figure 6b). It can be assumed that these variants of 5′-truncated copies were present in the genomes of the cockroaches that were the ancestors of all cockroaches in the studied populations. Additionally, loci 2 and 6 were unique markers of populations #1 and #4, respectively. It can be assumed that these variants arose from recent transpositional activity of the TEs in the period since the cockroaches populated a particular pig farm.

The frequencies of variants 1, 3, and 5 of the analyzed 5′-truncated copies, which were presumably present in the genome of the founder cockroaches of all studied populations, varied significantly in different populations. The following explanation is provided for these findings. The ribosomal RNA gene cluster within which the integration sites of the R2 retrotransposons are located is a typical example of a multigene family. Currently, three models that explain the maintenance of the structure and evolutionary variability of multigene families have been described: the concerted evolution [81], birth and death [82], and magnification and fixation [65] models. Each of these models is based on molecular genetic mechanisms that determine the isogenization of members of the multigene family based on stochastic recombination processes. From our point of view, it is precisely the random elimination of integrated copies of TEs that leads to changes in the frequency of the occurrence of the studied variants of 5′-truncated copies. In addition, one cannot exclude the possibility of a founder effect resulting from significantly different frequencies of the occurrence of the described 5′-truncated copies among the cockroaches that founded the populations on different pig farms.

Structural variants of 5′-truncated copies unique to each of these populations (loci #2 and #6) were identified in two of the eight described populations. As it was noted above, the simplest and most logical explanation for the emergence of unique variants of 5′-truncated copies is the occurrence of new acts of transposition during the period since the colonization of a new territory (in our model, pig farms) by cockroaches. However, the possibility cannot be ruled out that the emergence of these unique variants occurred before the dispersal of cockroaches to the studied pig farms, and the absence of specific variants of 5′-truncated copies is due to the founder effect resulting from the genotypes of the particular cockroaches that were the progenitors of the new populations. In addition, the elimination of damaged repeats within a multigene family consisting of a cluster of ribosomal RNA genes can occur due to stochastic processes.

Since the truncation of the synthesis of the first strand of R2 retrotransposons occurs randomly, the described pattern of the 5′-truncated copies of these TEs may be considered a unique molecular genetic marker differentiating the populations of the German cockroach, *B. germanica*. To verify this assumption, we used two approaches: Bayesian structure analysis and principal coordinates analysis.

The testing of four models via Bayesian clustering showed that the most appropriate model was the model with the absence of admixture and the correlation of allele frequencies and that the most likely number of clusters was five. The most common cluster is indicated in magenta, which is present in all populations. In most of the populations, the red cluster is quite pronounced, except in population #1, in which its representation is meager, and population #2, in which it is not found. The green cluster dominates only in population #1. In population #2, along with the magenta cluster, a yellow cluster is clearly represented. In population #5, along with the magenta cluster, a blue cluster is clearly represented. The listed distribution features of the identified clusters clearly distinguish the samples from populations #1, #2, and #5 both from each other and from other samples, which are more similar to each other (Figure 6c and Table 2).

The ordination of the samples in the space of the first two principal coordinates based on Nei’s genetic distance is presented in Figure 6d. The sample of population #1 is located the farthest from the other samples. The remaining samples form three groups combining samples from the following populations: #4, #6, and #7; #2, and #8; #3 and #5. The first group is more fragmented: the distance between the samples within it is at least two times the distance between the samples in the other two groups.

From our point of view, the obtained results convincingly indicate that the polymorphic patterns of the 5′-truncated copies of R2 retrotransposons can be considered a new molecular genetic marker that allows the differentiation of the populations of *B. germanica*. In the above analysis, only the variants of the 5′-truncated copies were used, which arose within a relatively short length of the R2 retrotransposon of *B. germanica* (456 bp) (Figure 6a). Since only amplification products with a length of 150–250 bp were analyzed, the truncation of the synthesis of the first strand of the R2 retrotransposons occurred at a distance of 206–306 bp from the 5′-end of the analyzed retrotransposons in all of the identified variants of the 5′-truncated copies. When using several different primer pairs that span the entire length of the mobile element, the number of detected variants of the 5′-truncated copies can theoretically be increased by more than ten times, which will significantly increase the resolution of the proposed method.

## 4. Conclusions

In this study, we cloned and sequenced a few full-length copies of the R2 non-LTR retrotransposon based on the screening of a gene library for the German cockroach, *B. germanica*. Compared to the analysis of the amplification products of total DNA, this approach allows the analysis of individual native copies of retrotransposons with multiple locations in the genome (copies integrated into different repeats of the 28S RNA gene in the case of R2 non-LTR retrotransposons).

Based on the analysis of the structure of 5′-truncated copies of the *B. germanica* R2 retrotransposon, we have previously shown that the position of the second single-strand break and therefore the size of the 28S rDNA deletion differs for all cloned 5′-truncated copies of the R2 retrotransposons [45]. Notably, all three full-size copies selected in this study from the gene library exhibited identical deletions of three nucleotides (AGG).

The structure of the R2 retrotransposons of a few species of termites (Blattoidea, the superfamily of cockroaches and termites, including the cockroach family Blattidae) was recently described [29]. For the comparative analysis of the structural organization of the R2 retrotransposons of cockroaches and termites, we also cloned and sequenced a long 5′-truncated copy of the R2 retrotransposon of the cockroach *R. maderae* containing the native ORF.

In general terms, the structural organization of the R2 retrotransposons described in cockroaches and termites is similar and corresponds to the characteristic structure of other species. As a characteristic feature of the cockroach R2 retrotransposons, we would like to note that the 3′-ends of all R2 retrotransposon copies revealed in *B. germanica* and *R. maderae* do not contain homopolynucleotide sequences. In addition, it remains unclear whether the dual structure of the second zinc finger domain of the cockroach R2 retrotransposons described in this study has functional significance.

Comparisons of R2 retrotransposon activity in termites (*Reticulitermes urbis*) both within and between colonies indicate very low or no transposition capacity [29]. In our experiments, we revealed significant differences in the frequency of the occurrence of various variants of 5′-truncated copies of the R2 retrotransposon between different populations of *B. germanica*.

Whatever the reason for the observed differences in the frequency of the occurrence of different variants of 5′-truncated copies of the R2 retrotransposon between different populations of *B. germanica*, it is obvious that this indicator can be considered a molecular genetic marker that theoretically makes it possible to differentiate populations of this insect species. In this study, we briefly tested this possibility.

Through the analysis of the spectrum of the 5′-truncated copies that can result from the accidental termination of reverse transcriptase activity after the generation of a relatively very short segment of RNA of the R2 retrotransposon (~100 b), we identified seven different structural variants. It is evident that the level of the transpositional activity of R2 retrotransposons in different insect species differs significantly; accordingly, the number of the detected variants of 5′-truncated copies will also depend on the object of study. However, the possibility cannot be ruled out that the proposed molecular genetic marker may be useful for studying the population dynamics of not only the German cockroach but also other insect species.

From our point of view, the new BLAG family of retrotransposons described in this study is of particular interest.

The analysis of the structural organization of these TEs showed the presence of a previously undescribed evolutionarily conserved domain in their ORFs. The functional role of this domain remains unclear. However, it has been shown that an evolutionarily conserved consensus amino acid sequence corresponding to this domain can be used as a probe to detect BLAG retrotransposons in the genomes of various organisms whose genome nucleotide sequences have been described.

BLAG retrotransposons are low-copy number site-non-specific TEs. However, although the data suggest that BLAG 1–BLAG 6 do not target a specific DNA sequence at the site of integration, it cannot be ruled out that these elements have a preference for genome-specific loci [83,84,85].

In all likelihood, this type of TE is characterized by one of the most primitive mechanisms ensuring transcription and, consequently, transpositional activity (i.e., random integration into the genome), implying random and rare integration into the region adjacent to the promoters of host organism genes.

Recombination between TEs plays an important role in both the formation of new types of TEs and increasing intraspecific genetic diversity [8,86]. The protein products of most non-LTR retrotransposons described to date contain zinc finger domains that play an important role in the interaction of these proteins with nucleic acids. Moreover, the number of zinc finger domains can differ (from one to three) between closely related retrotransposons. In this study, we described the structure of several types of BLAG retrotransposons with different numbers of zinc finger domains and showed that recombination between these TEs underlies the change in the number of domains. It can be assumed that recombination plays a decisive role in both the evolutionary variability of BLAG retrotransposons and the regulation of the number of zinc finger domains in other types of non-LTR retrotransposons.

## Figures and Tables

**Table 1:** Population analysis of the frequency of the occurrence of various variants of 5′-truncated copies of R2 retrotransposons of *B. germanica.*

Population	Number of Indiviuals *	Loci **						
		1	2	3	4	5	6	7
**1**	42/9	6/0.143	23/0.55	28/0.67	1/0.02	26/0.62	0/0	1/0.02
**2**	58/16	27/0.47	0/0	25/0.43	17/0.29	17/0.29	0/0	2/0.03
**3**	61/31	10/0.16	0/0	23/0.38	4/0.07	16/0.26	0/0	0/0
**4**	40/10	11/0.28	0/0	22/0.55	2/0.05	23/0.58	2/0.05	1/0.03
**5**	56/23	12/0.21	0/0	15/0.27	1/0.02	16/0.29	0/0	6/0.12
**6**	40/8	22/0.55	0/0	20/0.50	0/0	20/0.50	0/0	0/0
**7**	40/10	12/0.30	0/0	26/0.65	3/0.08	26/0.65	0/0	1/0.03
**8**	56/20	28/0.50	0/0	17/0.29	0/0	17/0.30	0/0	0/0

* Total number of analyzed individuals/number of individuals in which no amplification products were detected in a given length range. ** Number of individuals in which the amplification product of a certain length was detected/frequency of occurrence of that variant.

**Table 2:** Percentages of the clusters identified in the studied samples at *K* = 5.

Population	Cluster				
	1	2	3	4	5
**1**	0.014	0.600	0.006	0.000	0.379
**2**	0.001	0.000	0.000	0.293	0.706
**3**	0.246	0.001	0.009	0.013	0.731
**4**	0.548	0.001	0.012	0.007	0.432
**5**	0.023	0.001	0.257	0.000	0.719
**6**	0.497	0.000	0.003	0.000	0.500
**7**	0.631	0.000	0.009	0.010	0.350
**8**	0.303	0.000	0.000	0.000	0.697

Cluster colors: **1**, red; **2**, green; **3**, blue; **4**, yellow; **5**, magenta.

## Supplementary Materials

The following are available online at https://www.mdpi.com/2073-4425/11/10/1202/s1. Figure S1. Structural and functional characterization of the R2 retrotransposon of *B. germanica*; Figure S2. Structural of the non-site-specific R2 retrotransposons of *B. germanica*; Figure S3. Alignment of the nucleotide sequences corresponding to the 5′- and 3′-ends of all identified copies of BLAG 1–BLAG 6 retrotransposons, together with the corresponding sequences of the environments of the integration sites.; Figure S4. Comparison of BLAG 2 and BLAG 6 retrotransposon nucleotide sequences; Figure S5. Comparative analysis of the amino acid sequences of the BLAG 1–BLAG 6 retrotransposons located between the zinc finger domains and the reverse transcriptase domain; Figure S6. Alignment of amino acid sequences used to construct a phylogenetic tree; Figure S7. Population analysis of the structural variants of R2 retrotransposon 5′-truncated copies.
