# Supplementary material for: R2 and Non-Site-Specific R2-Like Retrotransposons of the German Cockroach, Blattella germanica
Source: Genes (Basel). 2020 Oct 15;11(10):1202. doi: 10.3390/genes11101202 (PMC7650587; doi:10.3390/genes11101202)
Supplement: Supplementary file 1 [file genes-11-01202-s001.zip › Supplements/Supplementary Figure 1.pdf]

A

>R2_Bg_Ribo

GTGCTGACGCAATGTGATTTCTGCCCAGTGCTCTGAATGTCAAAGTGAAGAAATTCAAGCAAGCGCGGGTAAACGGCGGGAGTAACTATGACTCTCT
TACGGGCACCCTGCCTGTAGGACACGACTGCAGTCTCCCTTCTGCAGACCTCGTCGGAAGATGAGGTGGATGTCGTGATGTCAGCTGGCTCCGGGAT
AATTTCCAGCCAGGGTTGGTAAGGGGGGGAGGAAGTCTAGTGATGCCTTGGGAAAGTTGAAGGCTCCTCTCTCTGACCAAAGCTGACACAGGGGAATG
GGTATGGGTTGACCCCCCATACTCATGAACTCTGGTCTTCGATAACGGTATCAGGCCTCGGACTTGGGGGGGGTATGGTTCCGCCTCCCCCTCGACC
TCCAGGGAACCACTTGTTGACGGACTCTGCGTGTCTATGGACGATTCTGGTTCTAACTCTGCCGTGCGCGTCTTGGTAGTTCTACCCTTCGAGCCTG
TTTTGTGCCCGGTCTGTCTCTCCGGGCGAGGGGCGAACTACCAGGCACTTAGTCTGAAGGACTACCTTGACCATCTTGGGAGAGCGCACGCGGCAGC
TGTGGTGGCATTTCAGTGCAGGAAATGCAAACGGGTGAATTCCAAGAAGCACAGTATTTTTGTGCATATTAGTAAATGTAGGGGTGAAACTGAAGCA
CCAACTGCTGCGCCATACCCTTGCAGGGTATGCGGGGAAGGCTTCAGTAGCAAGATCGGCCTCGGTCAGCACGAGAGGCACCGGCACCCTGTACAGA
GAAATGAGGCGCGAGCACCCACTCCCAAAACCCCACAGCGTGGGGCACCACCTGATGAgGGTCTCGCTCGATTGGCAATGCTCGAGGTGGAACTCGA
GTCCCGGGGGGGAGTTAAGTTCATCAATAAGGAGCTGGCTTGTAGGTTTGGGAGTCTTACGCCAAAGCAGATTAGCGACCGACGGAGGTCGGCCGCT
TACCGTGACCTTCTTTTGAAGGCTCGTGGTGGAGCGGTCGAAACCCCCCCTCCTGGGGGGCAAGGGGATGTTCTGCCTACATCCCAGGACGCGTCAC
GCGAGGAGCAGTGTGGCGTGTACCCAGATGCTGCAGTCCGTGACTCCAAAGTGGAGGAGACTACCGCTTCGATGGCGCCGCAGACCTGTGAATCCGA
TTCGGATGAGGTTGAGTGGCGCGAGGAAGCTCTCAATGCCTTTGTGGGAGCTCTGCCCACGGGACTTCCTCCCGAAGCGGAGGCCCTGGTACAAGCT
TGTCGAGAGGCTGCTGTGAATCAGGGGTCAAACCCTGACAAACAGGGCGTTATTGACGGCTTGTATGACTTAGTCATCCGGTTCTTGGCTCAAGCGT
CCCCCGCATGTGCTGGACCAGGTCCTCAGCGTCAACCTCGCCGAAAGGGCAAGGGCAAGAAAGCTCTTAAAAGGTTCAAGTTCGGAAGAGTCCAGGA
GCTCTTCAAACGTGATCCGCGTGTATTGGCAAAGCGCGTGTTGCAGGGACTTGAACCGTCTGGACTGAGGGACGGGGAACGAGATCTCCTCGAGCCC
TGTGATGTTCGAAGGTGCTACCAGGATCTCTGGGGAGAAAGACACCTTCAGACAAATCTTGACTGCACAGGCATGCCGAAGGCGGATGAGATCCCTA
TTGATCGAATCTGCTGCCGAATTGCCTGCGACGAGGTTGGGCGTCGCGTGCGCAAGATTAAGAGCACGACGGCTCCAGGGCCAGACGGAATTAACAT
CCAGGCAGTCCGTTCTGCGCCCGCCCAGGCTGTATTATCCACCTTTTACAGCCTTATCCTTCTCCTGGGACGGCCCCCGGCAGCCTGGCGGGAAAAC
CGATCTGTGTTAATACCGAAAGAGGGGTACGATGTTCGATTAATTTCGAATTATCGCCCTCTGACCATCGGATCGATTCTCAGTCGGATCTTTTGGG
GGATCATCGATCGACGGCTGAGGGAACTGTTAAAGATCCACCGGTATCAGAAAGGTTTTACTACGGAGCCAGGCTGCTATCAAAATGTGCGCATCTT
CTCGGAGGTACTGAGGCAGGCTAAGTCTGCACGCGGGCTCGTAGCGGTGCAGCTTGACATTTCAAAGGCCTTTGACACTGTACCACATGCTGCGATT
GCCACGGTTCTGCGCCAAAAAGGTCTTCCGCCATATTTTGCCGATATGGTGCGCCGGTCCTACGAGGGTGCGTACACGTCTATTAGGGTTAGGGACC
ACCGCATTCAACTGGAACTCAAGCGAGGGGTGAAGCAGGGGGACCCGATCTCACCTCTGCTTTTCAACATGATAATGGAACCGCTCCTCAACAAGCT
TGAGTCTCTGGGCGGTGTTACTTTGGCTGACGGCTCCCAGCTTGCTTGCCTTGCTTTTGCTGATGACTTGATACTCCTCGCCGACAATCGAGAGAGA
GCACGGGAGTTGCTTGAGACCGTGGAGGAGTTCCTGGATGGTTTGGGCATGAAGATCTGGGCGCCCAAAAGCGTCGCATTCGAGATCGTGCCAACCA
AGGACTCCTGGTGTGTCGTGGATCCGGGTCTTCGCCTGCGGCAGGGCGGCGCGATCACCTATGCAGGCCCGGATCACTCATTGAAGTACTTGGGTGT
TAAGGTGTCCCCGTGGGTCGGGGTTGATGTGTCCGACTTGCGCACCCATTTTTCCAAAACTTTGGAGGACACCCGCAAGCTACCATTAAAGCCCCAC
CAGAAGCTACATCTTTTGAAGACGTATCTCATCCCTCACTTCACGCACCGGCTGGTCTCTAGTCTGCCATCGATCGCAGAGCTGAGGGCTCTCGATC
TGGAGATCAGAGGATGCGTGAAGCAAATCCTTCATTTGCATCCGTCGACCGCGGATGGCATGCTTTATTCAAAGCCGAAGGATGGAGGGTTGGGAAT
ACCGTGTCTTGAGGTGGGTATAGTGTGCTCTGCACTCAAGATGGCCATAAAGACACGGCAGAGCGATGACCCAACTCTCCAAGCACTAGACCCCAAC
CTGATGGAACGAATGGAGAGGTACATCCGGAGCATACGCATTAGTGAGCTGACTGTGAAAGCAGTCAACTCCTATGCGCGAAGGGTGTACTCTGCTA
GGGTGGGTCAATGGTCTAACCTCCCGGTACAGGGTCGCGCGGTTAAGTCTCTGACCGGAGATAGAATTGGGAATTCTATTTTCTACGACCCGAGCCT
ATTAAAGCCATGTAGATTTATAACGGCTTTAAGGCTCCGTTCGAACACCGGGGGGAATAGGACAAATTTGTCAAAGATTAGACCACTCCAATCCGTG
CAATGTCGCCGCTGTGGTTTCCAGAGAGAGACACTGGGACACATACTTGGGCAATGTCTGCACACCAAGGCTGCACGGATACGGAGACACGACGCTA
TTAAAGACTTCATCTGCGATAGCGTCACTCGCGAAGGAGACAAAACGGCGACAGTTACTAGGGAGCCTGAGCTTCTCTTGAGTGGCGACTCGGGTAA
ACTGAAACCGGACCTGGTGATAACCCGCCAGGGAAGGGTCTTTGTGGTCGACGTAACAGTCCGCCATGAGGATTCAGATTACCTCGAGCGCGCTCGA
CGAGAGAAGATAGACAAGTACCAGGCGCTGTTGCCGTCGCTCATCGCCCAGTTTGGGGCAACTGGGGGTGAGGTGTTGCCCATTGTTGTGGGCACAC
GTGGAGCGATGCCCAAGCTTACCATGGAAGCTTTAGCTCAGTTGGGGATTACTGAGCGTGGAAGGTTGAAGACCATTAGCCTTATGAGTCTAAGAAG
TTCGATTGAGATCTACCATGGTTTCTTAGACTACGATGCATCGCTCCTATAATTTTGTTGTGGAAGGAACTACCGGCAAGCAGATTGTGAACCAGGA
GATTGATTGTCCTTACTCTATAGGCCCTTTTTAAGTAGGTATCCCATCTACAGGTACCTACCCTTCTTTTGACCGGGGATTAAAGTAAATTTAAGGC
TTCAGATTCGAGGGCAGCTGAGGCCTACTGGTTGCCTATTGCAACGGGCGCAATCTGCGAGTCTTCAGCTGAGTATCAAGCTTTTGATAGTAATTGC
AGCTGGGGGCGGCGAGTCTGCATTGCAGAGTGGACACCTACGCGAGGTTTTCCCTGTTAAGGTTTTCTTGCACCATAATGGTGGGGGACCGAACTAA
GTTCCATCCTCCTTCGGGGGGCTCGCTTGCCTGATTCTCCAAGCCCTTTTTGGCGCAAGAAAAGACCGTCTAAATTAATATAAAGGAACCTTCCCGG
CAGTGGTTGGGCCGGCGCTAGACTCGTTAATCTGTTTGAGAGGGGCAGATAATGGAGGGTCCTCGGTGGCCCTGCTCTCTGCACTTCTGCTTCCCAG
CTAAGGGTATGAGGTTACTATGCCTTCCAAAAAGATAGTAAGTTGCACTCGGTGGGGTGTTAGTAAGTAGCCAAATGCCTCGTCATCTAATTAGTGA
CGCGCATGAATGGATTAACG

B

>ORF_R2_Bg_Ribo

MDDSGSNSAVRVLVVLPFEPVLCPVCLSGRGANYQALSLKDYLDHLGRAHAAAVVAFQCRKCKRVNSKKHSIFVHISKCRGETEAP
TAAPYPCRVCGEGFSSKIGLGQHERHRHPVQRNEARAPTPKTPQRGAPPDEGLARLAMLEVELESRGGVKFINKELACRFGSLTPK
QISDRRRSAAYRDLLLKARGGAVETPPPGGQGDVLPTSQDASREEQCGVYPDAAVRDSKVEETTASMAPQTCESDSDEVEWREEAL
NAFVGALPTGLPPEAEALVQACREAAVNQGSNPDKQGVIDGLYDLVIRFLAQASPACAGPGPQRQPRRKGKGKKALKRFKFGRVQE
LFKRDPRVLAKRVLQGLEPSGLRDGERDLLEPCDVRRCYQDLWGERHLQTNLDCTGMPKADEIPIDRICCRIACDEVGRRVRKIKS
TTAPGPDGINIQAVRSAPAQAVLSTFYSLILLLGRPPAAWRENRSVLIPKEGYDVRLISNYRPLTIGSILSRIFWGIIDRRLRELL
KIHRYQKGFTTEPGCYQNVRIFSEVLRQAKSARGLVAVQLDISKAFDTVPHAAIATVLRQKGLPPYFADMVRRSYEGAYTSIRVRD
HRIQLELKRGVKQGDPISPLLFNMIMEPLLNKLESLGGVTLADGSQLACLAFADDLILLADNRERARELLETVEEFLDGLGMKIWA
PKSVAFEIVPTKDSWCVVDPGLRLRQGGAITYAGPDHSLKYLGVKVSPWVGVDVSDLRTHFSKTLEDTRKLPLKPHQKLHLLKTYL
IPHFTHRLVSSLPSIAELRALDLEIRGCVKQILHLHPSTADGMLYSKPKDGGLGIPCLEVGIVCSALKMAIKTRQSDDPTLQALDP
NLMERMERYIRSIRISELTVKAVNSYARRVYSARVGQWSNLPVQGRAVKSLTGDRIGNSIFYDPSLLKPCRFITALRLRSNTGGNR
TNLSKIRPLQSVQCRRCGFQRETLGHILGQCLHTKAARIRRHDAIKDFICDSVTREGDKTATVTREPELLLSGDSGKLKPDLVITR
QGRVFVVDVTVRHEDSDYLERARREKIDKYQALLPSLIAQFGATGGEVLPIVVGTRGAMPKLTMEALAQLGITERGRLKTISLMSL
RSSIEIYHGFLDYDASLL

C

Zn finger domains:

#1 YPCRVCGEGFSSKIGLGQHERHRH
#2 FQCRKCKRVNSKKHSIFVH
#3 VLCPVCLSGRGANYQALSLKDYLDHLGRAH

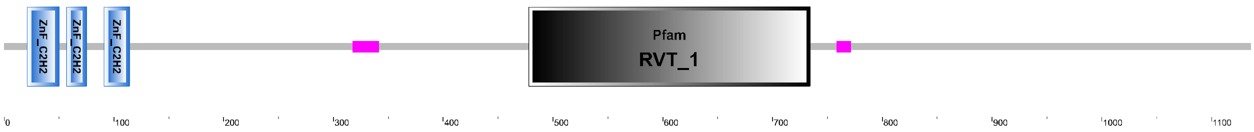

D

C-myb motif:

```
Q ss_pred            CCCHHHHHHHHHHHHHHhHhccCchHHHHHHHHHHcCCCCHHHHHhhhhcHHHHHHHHHH
Q Q_R2_ORF_Bg        APPDEGLARLAMLEVELESRGGVKFINKELACRFGSLTPKQISDRRRSAAYRDLLLKA
Q Consensus       29 rWs~eE~~~La~lE~~l~~~~~~~~~~in~~L~~~~~p~Rt~eaIk~~Rr~~~yk~~v~~~
                     .||.+|..+|..+-..+   ......-..|+..||+||..+|+.+      |..++..+
T Consensus       35 ~WT~eED~~Ll~~v~~~~---G~~~~~W~~Ia~~lpgRT~~q~k~R-----~~~Ll~~~   84 (95)
T 1UG2_A          35 LWTREADRVILTMCQEQ---GAQPHTFSVISQQLGNKTPVEVSHR-----FRELMQLF   84 (95)
T ss_dssp           SSCHHHHHHHHHHCCCC---CSCTTTHHHHHHHHSSCCHHHHHHH-----HHHHHHHH
T ss_pred            CCCHHHHHHHHHHHHHHh---CCCcccHHHHHHHhCCCCHHHHHHH-----HHHHHHHH
```

HHpred alignment with mouse hypothetical gene (2610100B20Rik) product homologous to Myb DNA-binding domain (PDB accession number - 1UG2_A).

Probability: 92.4%, E-value: 0.78, Score: 36.1, Aligned cols: 50, Identities: 20%.

E

Reverse transcriptase domain:

```
Q ss_pred             HHHccCCCCHHHHHHHHHhcCCCC-CCCCCCCCHHHHHcCcHHHHHHHHHHHHHHHHCCCChHHhcceEEEEeCCCCCcch
Q Q_R2_ORF_Bg         IDRICCRIACDEVGRRVRKIKSTT-APGPDGINIQAVRSAPAQAVLSTFYSLILLLGRPPAAWRENRSVLIPKEGYDVRL
Q Consensus           ~~~l~~~it~~Ev~~ai~~~~~~~~-ApGpDgi~~~~lk~~~~~~~~~l~~l~n~~l~~~~~~P~~~k~~~~vlipK~~~~~~~~
                      .+.|..+++.+||.++++.++.++ ||||||++.+.++..  ....+..+++.++..+..|..|+. ++|||++
T Consensus        1    ~~~l~~~~~~~~ei~~a~~~~~~~~~~~~apG~dg~~~~~~~~~~~-~~~~~~~~~~~~~~~~~~~~~~~~~~~~---~~i~k~~~-----   71 (428)
T 6AR1_D           1    MALLERILARDNLITALKRVEANQGAPGIDGVSTDQLRDY-IRAHWSTIHAQLLAGTYRPAPVRR---VEIPKPG-----   71 (428)
T ss_dssp              -CHHHHHSCHHHHHHHHHHHHHHHHTCCCCTTCCCGGGHHHH-HHHHHHHHHHHHHHHTCCCCCCBEE---EEEECTT-----
T ss_pred              CcHHHHHcCHHHHHHHHHHHHHHHcCCCCCCCCCCHHHHHHH-HHHhHHHHHHHHHHcCCCccCCCceE---EEEeCCC-----

Q ss_pred             hhhcCchhHHHHHHHHHHHHHHHHHHHHhc--ccCCCCCCCCCchHHHHHHHHHHHHHHHHHHcCCEEEEEEeehhccCCC
Q Q_R2_ORF_Bg         ISNYRPLTIGSILSRIFWGIIDRRLRELLK--IHRYQKGFTTEPGCYQNVRIFSEVLRQAKSARGLVAVQLDISKAFDTV
Q Consensus           ~~n~RPItl~s~~~Ki~~kil~~Rl~~~~~~~--~~~~~~Q~gf~~~~g~~~~~~~~~~l~~~~~~~~~~~~~~~~~~~~~~~~~lD~~kAFDsV
                      ++||||++.+..+|++.+++..+|...+.   ..+.|.||++++++.+++..+...+.    .....++.+|+++||||+|
T Consensus        72   -~~~R~i~~~~~~~~k~~~~~~l~~~l~~~~~~~~~~~~~~~~~~q~~~~~~~~~~~~~~~~~~~~~~~i~~~~~~~~~~-----~~~~~~~~~~~~~~~~~D~~~afd~i   146 (428)
T 6AR1_D           72   -GGTRQLGIPTVVDRLIQQAILQELTPIFDPDFSSSSFGFRPGRNAHDAVRQAQGYIQ----EGYRYVVDMDLEKFFDRV   146 (428)
T ss_dssp              -SSEEEEEEECCHHHHHHHHHHHHHHHHHHHHHGGGSCTTBCSSCTTCCHHHHHHHHHHHHHH----HTCCEEEEECBTTTTTTC
T ss_pred              -CCeecCccCCHHHHHHHHHHHHHHHHHHHHhCcccCcCCCCCCCCCCCCHHHHHHHHHHHHH----cCCcEEEEechHHhccCC

Q ss_pred             CHHHHHHHHHHCCCCHHHHHHHHHHHHhCCcEEEEECCeeeEEEeCcccCCCCCCChHHHHHHHHHHHHHHHHHHHCCCccccCC
Q Q_R2_ORF_Bg         PHAAIATVLRQKGLPPYFADMVRRSYEGAYTSIRVRDHRIQLELKRGVKQGDPISPLLFNMIMEPLLNKLESLGGVTLAD
Q Consensus           ~h~~l~~~L~~~g~~~~~~~~~~~~i~~~~~~~~~~~~~~i~~~~~~~~~~~~~~~~~~~~~~~Gv~QGdpLSPlLF~l~~~~~l~~~l~~~~~~~~~~~~~~~
                      +|+.+...|...++++.+..++..++..........     +...+|++||+|+||+||+++++.+...+..
T Consensus        147  ~~~~l~~~l~~~~~~~~~~~~~~~~~~~~~~i~~~~~~~~~~~~~~~~~~~~~~~~~~--------~~~~~~Gl~qG~~lsp~l~~~~~~~~~~~~~~~~~l~~--------   213 (428)
T 6AR1_D           147  NHDILMSRVARKVKDKRVLKLIRAYLQAGVMIEGVK-----VQTEEGTPQGGPLSPLLANILLDDLDKELEK--------   213 (428)
T ss_dssp              BHHHHHHHHHHTTCCCHHHHHHHHHHHHHHCCEESSSCE-----ECCSBCCCTTCTTHHHHHHHHHHHHHHHHHHHHH--------
T ss_pred              CHHHHHHHHHhHcCCHHHHHHHHHHHHHHcCcEeCCee-----eecCCCcCCCCCCcHHHHHHHHhhHHHHHHHHH--------

Q ss_pred             CCeEEEEEEccCeEEecCCHHHHHHHHHHHHHHHHHHH-ccccCCCCCeEEEEEeCCCCCceEEeCCCCceEeeCcccceecCCC
Q Q_R2_ORF_Bg         GSQLACLAFADDLILLADNRERARELLETVEEFLDG-LGMKIWAPKSVAFEIVPTKDSWCVVDPGLRLRQGGAITYAGPD
```

```
Q Consensus          ~~~i~~l~fADD~~l~~~s~~~l~~~l~~l~~~~~~-~GL~iN~~K~~~~~~~~~~~~~~~~~~~~~~~~~~i~~~~~~
                      ..+.++.||||+++++.+.+.++..+..+.+++.. .|+.+|.+||..+ ..
T Consensus      214 -~~~~~~~~yaDD~~l~~~~~~~~~~~~~~~~~~~~~~~~~gl~in~~K~~~~---------------------------~~   264 (428)
T 6AR1_D         214 -RGLKFCRYADDCNIYVKSLRAGQRVKQSIQRFLEKTLKLKVNEEKSAVD---------------------------RP   264 (428)
T ss_dssp           -HTCEEEEETTEEEEEESSHHHHHHHHHHHHHHHHHHTSCCCBCTTTCBCC---------------------------CG
T ss_pred           -cCCccceecCceEEEECCHHHHHHHHHHHHHHHHHHhhCCCcChhhcccC---------------------------Cc

Q ss_pred           CceEEcceeeccc---cCCCHHHHHHHHHHHHHHHHH--cCCCCHHHHHHHHHHhHHHHHHHHHHcCCCCHHHHHHHHHHH
Q Q_R2_ORF_Bg      HSLKYLGVKVSPW---VGVDVSDLRTHFSKTLEDTR--KLPLKPHQKLHLLKTYLIPHFTHRLVSSLPSIAELRALDLEI
Q Consensus          ~~~kYLGv~~~~~~---~~~~~~~~~~~~~~~~~l~~i~--~~~L~p~qkl~~l~~~~iP~l~y~l~~~~~~~~~~~~l~~lD~~i
                     ..++|||+++...    ...........+...+..+. ...++..+++..++..+.+.+.|.....  ....++++|..+
T Consensus      265 ~~~~~LG~~~~~~~~~~~~~~~~~~~~~~~~~~~~l~~~~~~~~~~~~~~~~~~~~i~~~~~~~i~~~~~~y~~~~~~--~~~~~~~~l~~~~   342 (428)
T 6AR1_D         265 WKRAFLGFSFTPERKARIRLAPRSIQRLKQRIRQLTNPNWSISMPERIHRVNQYVMGWIGYFRLVE--TPSVLQTIEGWI   342 (428)
T ss_dssp           GGSCBTTEEECSSSSCCEEECHHHHHHHHHHHHHHSCCC--CCHHHHHHHHHHHHHHHHHHHHTTCS--CHHHHHHHHHH
T ss_pred           cccccccEEEEecccccccCHHHHHHHHHHHHHHhCCCCCCCHHHHHHHHHHHHHHHHHhhhhcC--CHHHHHHHHHH

Q ss_pred           HHHHHHHHcCCccCCCccceecCHhhCCCCCCCHHHHHHHHHHHHHHHHhcCCCHHHHHHcHHHHHH
Q Q_R2_ORF_Bg      RGCVKQILHLHPSTADGMLYSKPKDGGLGIPCLEVGIVCSALKMAIKTRQSDDPTLQALDPNLMER
Q Consensus          r~~vk~~l~lp~~~~~~~~ly~~~~~GGLGl~~l~~~~~~~~~~~k~~~~l~~s~d~~~~~~~~~~~~~~~
                     +++++.+.........   +.+...||||+++++........+..+++..+.+ ....+.......
T Consensus      343 ~~~l~~~~~~~~w~~~~~---~~~~~~~gglG~~~~~~~~~~~a~~~k~~w~~~~~~~~-~~~~~~~~~~~~~~   404 (428)
T 6AR1_D         343 RRRLRLCQWLQWKRVR---TRIRELRALGLKETAVMEIANTRKGAWRTTKTPQ-LHQALGKTYWTA   404 (428)
T ss_dssp           HHHHHHHHHHHCCSHH---HHHHHHHHHTCCHHHHHHHHHTCCSCHHHHTTSHH-HHHHTSHHHHHH
T ss_pred           HHHHHHHHHHhccChH---HHHHHHHHCCCCHHHHHHHHhcCCChhHhcCCHH-HHHHHCHHHHHH
```

HHpred alignment with Group II Intron Reverse Transcriptase (PDB accession number - 6AR1).
Probability: 100%, E-value: 1.4e-31, Score: 319.99, Aligned cols: 395, Identities: 20%.

F

Endonuclease-like domains:

Cap snatching endonuclease

```
Q ss_pred             hHHHHHHHHHHHHHHHHHHHHhcCCCceEEEeCCceecCCCCCccccеEEEEE-CCEEEEEEEEccCCC-hhHHHHHHHHH
Q Q_R2_ORF_Bg         KAARIRRHDAIKDFICDSVTREGDKTATVTREPELLLSGDSGKLKPDLVITR-QGRVFVVDVTVRHED-SDYLERARREK
Q Consensus           ~~~ri~RHn~V~~~l~~~l~~~~~~~~~~~~v~~ep~~~~~~~~~~~~~~rPDlv~~~~-~~~~~ViDVtV~~e~-~~~~l~~a~~~K
                      ...+.-+||-++..|...-...-...|-+..       ++.+.+-||.|+.. ++..+||+++=.--+ ...+..+++.|
T Consensus        73   ~~~~~f~HDftf~~L~~~TD~~l~~~F~~~~-------d~~d~lTPD~I~~~~~g~~~VvEftT~r~~~~~~~l~~~~~~~K   145 (226)
T 6NTV_A          73   SEAYKINHDFTFSGLSKTTDRRLSEVFPITH-------DGSDGMTPDVIHTRLDGTIVVVEFSTTRSHNIGGLEAAYRTK   145 (226)
T ss_dssp              HHHTTHHHHHHTTTTCCCCCCCGGGTSCCCC-------SSCTTCCCSEEEECTTSCEEEEEEEECCCCSHHHHHHHHHHH
T ss_pred              HHHhhcCceehhhhhccccCCcHHHhCCCCC-------CCCCCcCCcEEEEeCCCeEEEEEEEeCCCCCChHhHHHHHHHHH

Q ss_pred             HHHHHHHHHHHHHHHHCCCccEEEEEEEccCCCCC
Q Q_R2_ORF_Bg         IDKYQALLPSLIAQFGATGGEVLPIVVGTRGAMP
Q Consensus           ~~KY~~l~~~~~~~~~~~~~~~~v~v~~ivvgarG~~~
                      +.||...........+..++.+..|+||..|.|.
T Consensus        146  ~~KY~~~l~~r~~~~~~~~~~~i~l~vIvV~~~~~V~s   179 (226)
T 6NTV_A          146  IEKYRDPISRRVDIMENPRVFFGVIVVSSGGVLS   179 (226)
T ss_dssp              HHHHHHHHHHHHHHTSSSCEEEEEEEEECSSEEEE
T ss_pred              HHHcHHHHHHHHHHHhcCCCcEEEEEEEEeCCCcEEe
```

HHpred alignment with Cap snatching endonuclease, Severe fever with thrombocytopenia syndrome virus (SFTSV). PDB accession number 6NTV_A.
Probability: 95.69%, E-value: 0.031, Score: 60.56, Aligned cols: 105, Identities: 24%.

Holliday junction resolvase

```
                               α1              β1                   β2        β3
Q ss_pred                 HHHHHHHHHHHHHHHHCCCCCEEEEECCCCCCCCCCCCCCCCEEEEECCEEEEEEEEEEC
Q R2_ORF_Bg               RRHDAIKDFICDSVTREGDKTATVTREPELLLSGDSGKLKPDLVITRQGRVFVVDVTVRH
Q Consensus               ~RHn~I~~~i~~~l~~~~~k~w~v~~Ep~i~~~~~~~~~kPDLV~~~~~~~~ViDVtV~~
                          +|-..--+.+...|.+   .||-|.     +.|.++.   ||||+.+++..++++|-...
T Consensus             3 rkG~~~ERel~~~L~~---~Gf~vv-----R~~gSg~---pDiiA~~~~~~~aiEvKst~   51 (120)
T 1gef                  3 RKGAQAERELIKLLEK---HGFAVV-----RSAGSKK---VDLVAGNGKKYLCIEVKVTK   51 (120)
T ss_dssp                  HHHHHHHHHHHHHHHH---TTCEEE-----ECGGGSS---CSEEEECSSCEEEEEEEEES
T ss_pred                  CCHHHHHHHHHHHHHH---CCCEEE-----EECCCCC---CCEEEEECCEEEEEEEEECC


                                      α2                  β4
Q ss_pred                 CCCHHHHHHHHHHHHHHHHHHHHHHHHCCCCCEEEEEEEECCCCCC
Q R2_ORF_Bg               EDSDYLERARREKIDKYQALLPSLIAQFGATGGEVLPIVVGTRGAM
Q Consensus               e~~~~L~~a~~~Kv~KY~~l~~~i~~~~~~~~V~~~~vvigaRG~w
                          ..+-.++..--+|..       +..+.++++.  ++++=+.-+| |
T Consensus            52 ~~~iyi~~eqvekL~-------~f~~~fg~~p--~IAvkf~g~~-W   87 (120)
T 1gef                 52 KDHLYVGKRDMGRLI-------EFSRRFGGIP--VLAVKFLNVG-W   87 (120)
T ss_dssp                  SSCEEECHHHHHHHH-------HHHHHHTCEE--EEEEEETTTE-E
T ss_pred                  CCEEEECHHHHHHHH-------HHHHHCCCCE--EEEEEECCCC-E
```

HHpred alignment with the archaeal Holliday junction resolvase (Hjc) from *Pyrococcus furiosus* (PDB accession number - 1GEF).

Probability: 90.96%, E-value: 2.1, Score: 23.84, Aligned cols: 85, Identities: 20%/

Supplementary Figure 1

Structural and functional characterization of the site-specific R2 retrotransposon of *Blattella germanica*.

A – Nucleotide sequence of the site-specific R2 retrotransposon of *Blattella germanica* with the environment of the integration site. The sequences of the 28S RNA gene are highlighted in gray, the sequence of the open reading frame (ORF) is highlighted in yellow, and the sequences without background highlighting correspond to 5'- and 3'- untranslated regions. The sequences of the primers used to amplify full-length copies of this mobile element are underlined.

B – Domain organization of the protein corresponding to the ORF of the R2 retrotransposon. Zinc finger domains are highlighted in yellow, the c-myb domain in crimson, and the reverse transcriptase domain in blue. The endonuclease-like domain shows significant similarities to Cap-snatching endonucleases (underlined) and Holliday junction resolvases (highlighted in green).

C – Domain organization predicted by SMART (Simple Modular Architecture Research Tool). Blue boxes indicate zinc finger domains, and gray boxes indicate reverse transcriptase domains. The amino acid sequences corresponding to the three zinc finger domains located at the N-terminus of the ORF are shown.

In this study, for the identification of similarity and prediction of the structure of the domains of the R2 retrotransposon, we applied HHpred software, which was used for the detection of HHsearch PDB hits. The maximum similarity was revealed between the analyzed domain of the R2 retrotransposon of *B. germanica* and the following proteins with known structures related to the **D** – c-myb motif, **E** – reverse transcriptase domain, **F** – endonuclease-like domain (Cap-snatching endonucleases and Holliday junction resolvases).
