# Supplementary material for: R2 and Non-Site-Specific R2-Like Retrotransposons of the German Cockroach, Blattella germanica
Source: Genes (Basel). 2020 Oct 15;11(10):1202. doi: 10.3390/genes11101202 (PMC7650587; doi:10.3390/genes11101202)
Supplement: Supplementary file 1 [file genes-11-01202-s001.zip › Supplements/Supplementary Figure 2.pdf]

>Blag_1

GGCCTATGCCTATATCTGACCAAGTTTCATTAAACGTATGAATACCGAAACAAACGAGCAATCCGACCATACTACTATCAACGTCGATTTCTCACAC
GCTGAAGTAGGCCTATCACAAACGCAAGTCAGTAGCCAAAACCCTTGTCCCCATTGTGGGGGCTTGTTTAAAGGTACTCATGGTGTAGCCATCCACA
TCAGTCGTGCACACCCCCCCGAACACAGACAATCCATTCTGACTAGGCAATCAAGAACAAAACAGTCCACATCTAGTGAGCCACAGCTTCGAAATCC
AACTGATAATGATCAATCTATCGTCGATAGACAAGTTGATGTAACTGAAATTAATGATTGTTCAGTCCTAAATGATGACCTCGCAACTTACAAAAAT
GAAATGCAAAAATGGAAACTTGAATTCTCAAAAAATAACACAAACGACAGTTTCAATACCAAAGTCCAGGAATTCCTTAATTTCCTCGCTCGAGCTG
TACATTTACTTCCTGGTCCTAAACACCCAGCAGTACGCTTTTACGAAGCACGACGTCAGCGCCGACAGTTCAGCAATCAGGTTCGTTATAAACAATC
CAGCAATCCTGAACGAGGAACCAAAAGGGCTCGAGACAAGAGGCGCGAATTGTATGAATACCAGAAAACGCAATTCCTTTTTTACAACCAGAGGAGA
AAAGCTGTTAGATCAGCACTGAACGATAGGCACACGGCTTGTAAAGTTGATATTAACAAAATCTTCAACTATTTTTCGTCTCGACTATCTACTGAGA
ATTCTTGCGAACGTCCAGCATACTCTTCCAATGTCACCGAACCGGAGCGAGCTGTTTTAGACAGTACAGAGGATTCTGTTATCACTAAACAAGAGGT
GGCAGTAGCTGTAAGCAAGATAGCTGCTGACACTGCTCCAGGTCCTGACCACGTGCTTGTCAGAGCATTAAAAGATGCTACCTGCTATGAAATTATA
GCCAAGATAGCTACCACCATGCTACAACATTCTTTTGTACCAGAATGCCTACAGAAAGCACGAACTGTGTTAATTTATAAAGGTGGAGATGATAAGG
ACCTTGGTAACTGGAGACCTATAACAATCTGTTCTGTGGTGAGACGGATAATTGAGCGAATACTCGATGCACGTCTACGAGCATTAGTGAACCTATG
CGAAAATCAAAGAGGATTTACTAACTCTCCTGGTACCTTAATAAACACATCTATTTTAGAATCCATTTTGGTTGCCGCGAAGTCGAAGAAAAAAGAT
TGTACAATAGTTTTCTTGGATATAAGTAAAGCCTTCGACAATGTGGGTCATAACCACTTACAGAACAGCTTGTCTGCACTTAGAATACCTTTCAAAT
TATCATCTTTGATATTACAGCTGCAGAGGGGTAATAAAACCCAAATAGAAACAGGAAGATGTAGAACTAAACCAATAGACATAAAGAGAGGAATCAT
GCAGGGTTCACCACTCTCTCCCATTCTTTATAATATATCTACTGACCATATCCTCGATGAACTTTCCAATCAAGAGTTGCAGGATGAATATGGATTC
TCAGTATCACCAGGCCTATCTAACATAACAGCATTAGGCTTTGCTGATGACACGTTGATTGCTGCTAAAAGTATGAATTCAGCACGAATTCTTGTAG
AACTTGCCATGAGTAGATTTAAAGAGATTGGATTAGACATAAATACAGACAAATGTAGATGCATCTCCATAATCAAGGGATGTGTCAGATATAACCC
TCTTAATTTAACTTCTGGTCAAACAATTGCATCAATTACTCCTGATGAAAACATAAAATACCTTGGTGTCAGTTATAATGCTACTATCCTTGATGCA
GCCAACTCTATAGAAGAATTAAAAAATAAACTGGATAGACTTGCTTCAACACCTTTATTACAACCCCACCAGAAATACACTGTTTTATGTAGCTTTA
TCTGTCCTACTCTGATCTACAAATTTCAAACAACACCACTTAAGAAGCTACCTGCTAAATTTCTTTCCGATGCAGACGTTCTCATTAAAACCACATT
GAAGGAGGTACTTCAACTTCCAACAGATACACCTGACAGTATGCTGTATACCGATAGAAAATACAAAGGATTGGGACTCTTTAATTCCAGCTGGGAA
GCTTTTTTGCAGCATATTAATGCTTGTCAGAGATTACAACAATCCCAAAATATACATGTTAATACCTGCCGCAACTTGTCCCACGAAATTAAGACTT
GCATAGAAAATCTGGGGCTTGAGACCACCGAATCCGAAACATACAGTGTTAAAAAAATTAGGAAAGAATTACGAGAGCGAGCTTTCCAATCCTGGTG
CTCTTTAAGCCAGAAAGGTAAAGGTGTCATATTATACAAAGAGCACACTCCGGCCAACAAATGGATTAGGCAACCAGATCGCCTTAGTAGTAGTGAT
TGGAGACTAGCTCTCAAAATGACTGCCAACGTCTGCCCCCTACGAGCTGTTCCTGGAAGGTCCCGAGACGGAAACCACTGTCGCCGTTGCAGCAGTG
AGATGGAAACACTTGCACATGTCTTGGGTGCCTGTCCTTTCAGCGAGTCTCTGCGAAATTCACGACACCACCATATAAGATCTGTGATCGCCGATGC
TCTAAGAAAAAAGGGACTTGTTGTGCACGAGGAAGTTCACGGAATCTCTACGAATGGATCTTCTCGCCGCATTGATATGCTTGCCATTCCTCCAGGC
TCTACATCCGCCTATATCATCGACCCGACAGTCCGATTTGAGACAGACGAACAACAGCCCGCGGAAGTAAATGCTGAAAAATGCAGAATCTACAACC
CCACAGTTCCATTCTATCTGGAAAAATACCAACTCACCTCCATAGAAGTTATTGGACTATTGGTTGGGGCTCGGGGCACAATACCTGGCTTCTTTGT
GAAATTCTGCAGGAAATTCCAGCTCAATAATAATCTAATTAGAGATGTTTCCCACGCTGCCATCAAAGGCTCATTGGCCATTTTAAGGAATCATCTA
TACTCTCTTTGTTAAAAAATACTTATAATTTTATGGCAGACTATTATTTTCTATACTGGACTTATTGTATTTATTTTATGTATTTCTAAAATTATTT
TGTGTTTCCGTCCCAATCATTTAACAACTTTCAGTTTGCCATTATATTATGCTTTGTCCACTGTGGCAGTCCCTTAGTTGGGAAAGCCGAAAGGTTC
ACTTTCTAAATAAA

>ORF_Blag_1

MNTETNEQSDHTTINVDFSHAEVGLSQTQVSSQNPCPHCGGLFKGTHGVAIHISRAHPPEHRQSILTRQSRTKQSTSSEPQLRNPTDNDQSIVDRQV
DVTEINDCSVLNDDLATYKNEMQKWKLEFSKNNTNDSFNTKVQEFLNFLARAVHLLPGPKHPAVRFYEARRQRRQFSNQVRYKQSSNPERGTKRARD
KRRELYEYQKTQFLFYNQRRKAVRSALNDRHTACKVDINKIFNYFSSRLSTENSCERPAYSSNVTEPERAVLDSTEDSVITKQEVAVAVSKIAADTA
PGPDHVLVRALKDATCYEIIAKIATTMLQHSFVPECLQKARTVLIYKGGDDKDLGNWRPITICSVVRRIIERILDARLRALVNLCENQRGFTNSPGT
LINTSILESILVAAKSKKKDCTIVFLDISKAFDNVGHNHLQNSLSALRIPFKLSSLILQLQRGNKTQIETGRCRTKPIDIKRGIMQGSPLSPILYNI
STDHILDELSNQELQDEYGFSVSPGLSNITALGFADDTLIAAKSMNSARILVELAMSRFKEIGLDINTDKCRCISIIKGCVRYNPLNLTSGQTIASI
TPDENIKYLGVSYNATILDAANSIEELKNKLDRLASTPLLQPHQKYTVLCSFICPTLIYKFQTTPLKKLPAKFLSDADVLIKTTLKEVLQLPTDTPD
SMLYTDRKYKGLGLFNSSWEAFLQHINACQRLQQSQNIHVNTCRNLSHEIKTCIENLGLETTESETYSVKKIRKELRERAFQSWCSLSQKGKGVILY
KEHTPANKWIRQPDRLSSSDWRLALKMTANVCPLRAVPGRSRDGNHCRRCSSEMETLAHVLGACPFSESLRNSRHHHIRSVIADALRKKGLVVHEEV
HGISTNGSSRRIDMLAIPPGSTSAYIIDPTVRFETDEQQPAEVNAEKCRIYNPTVPFYLEKYQLTSIEVIGLLVGARGTIPGFFVKFCRKFQLNNNL
IRDVSHAAIKGSLAILRNHLYSLC

>Blag_2

GATCAGTGATGGTGAGTGCTGTGTAAAAGATGACGTCGAATGCGACCCCGCTGTTACTTAAACATCATTATACTTGTTGATATCTTCAAACTACTAC
AATTTATGTTGGATTTTCTCCGTGAAGACTGACTTCTCTCCTTAGACATTGTTTTACTCATGATATCAGATTCTGGAGGACCTTCGTTACCATCTAA
TGACGGTAAGACTATTGCTTTAGTCTCTTGCAAGATATGTGGAAAAAGTTTTACTAGACATGGAATTAATATCCATATTGCTAGAAGCCATAGACAT
TCCCAAGTTTTGATAGACATTGATAAGCCTACTGATTCAATTTTATCAGAGACTGTAAACTTCTCACCTTCTGACCCTATTGAATGCCAGGATAATA
TCCATCTTAATACTAATTCATCTAACAATGAGAAAGTCATTCGTAACAATTCCAATGCATCAAGTATACATCTAATAGATGGTTCATTAATTGGTAC
TCCCTCTGACTCTTCTCCAGCTGTTTCGCAAGCTCAATGTCCCCATTGTGGAGGTTCATTTAACGGATCTAAGGGTGTCAATATTCATATTAGTCGA
TCGCATCCCCACATTCATAGATCGACATTAGTTACAAATTTAACTAAAAATGCGTCCCAGCCGCTCCTTGATGAGCAAGCTGATAGGGGTAGTTCTA
GTAATTCAGAATCCAGTCAACCTGACTCAGGTTTTAACATAGCCCTGAATGAGTGGAAAACAAAATTTGTTCGCACTGCAACTGCAAATGACTCTGA
TTTCGATAACATGGTCCAGGATTTTTCACACTTCTTAAGTACTAGCATCAATCTTTTACCCGGACCTCGACATCCCGCAACCAAGTACTATGAAGCC
CGCAGGAAACGACGTCTCGCAACAGCAGAAAAGAACTACAATAAGTCCTCCAACCCCATACGTGCCAGCAAAAAAGCCCGTGAGAAGAGGAAATGCA
AATACGATTATCAACTGACGCAATACCAATTCTATAATCAGAGACGTAAGGCCGTCCGTCGAGTTCTCGATTCTACAGCACCTTCTTGTAAAATCGA
TCTCACTAAAGTTCATGAAAATTTTGCTGCCAGTTTTTCCATTGAAAATCCACACATCAGGTCAAATTACCCTTCCAAACTAACTGACGTGGAACGT
ATAGAACTAGATGAGACATACGATCCTACTATCTCCAAAGAAGACATCGCTATTGCAGCCCACAAAATTGCAGTGGACACAGCCCCCGGCCCTGATC
ACGTTCTTGTTCGAGTCATCCGCAATGAGATTGTGTATGAAATCTTAGCAATAATTGCTACAAGAATGCTTTCCTCTGGAATCGTTCCTCGTTGCTT
CAAAACAGCTCGGACTGTCTTAATACATAAAGCTGCAGATGTTAACATCCTCTCGAACTGGAGACCGATCACCATTTGTTCTGTACTTAGGAGAGTT
ATTGAACGAGTTCTGGACAAACATTTACGTACTTTTGTTAGCTTCAATGAACATCAGAGAGGCTTTACTAATAATTCAGGCACTCTTATCAACACGT
CTATCCTAAGAAAAGTTTTAAAAACCACAAAGGACAACAAAGAAGATGTTACTGTAGTATTTTTAGATATATCGAAGGCTTTCGATAATATTGGGCA
CAAGCATTTAGATTTAACATTATCAGCAGAACCTATCCCTGCTAAACTTAAAGATCTTATCATAAATCTACAAATCAACAATACCACGAATATCCAA
ACATCTCTAGGTCAAACAAAAGCTATTAACCTACGACGTGGTGTTATGCAAGGCTCCCCTTTATCGCCAACATTATATAATCTTGCTACAGATTTCA
TCTTCGATGAACTCACTGAATTGAGTCTTTTGCAACAATACGGAAAATCCATCTCTCCTCAGTTATCTTCTCTTAGCATCATGGGCTTCGCTGATGA
CACTCTCCTCATGGGGAAAAATTCAGAAACTACTCTCGAATTATTACATATTGCACTACAGCGTTTTCAAGAAATAGGTCTACAAGTAAATCCTCAA
AAATGCACGGGAATATCTATAAAAAGAGGAAAACTGGTTGAAGAGGTTCTTCATATCAATGAAAAATTCACCATTAAGACCATAAATAATCAAGAAG
AAGTTCGTTACCTTGGGGCCCCCTTTCAAGATAACGCTCTCTTTGATAAAGACTCTACTATAAAGCAGCTGCATAAGAAATTAGAACTATTAGCTTC
ATCCCCTTTACTAAAATCTGATCAAAAATTCCTAGTCCTAAATACCGCTATTTGTCCAACCCTTATTTATCGCTTTCAAACCACAGATCTACAAACC
ATTCCTTTGCAGTTTTTACATACTGCCGATAAATTAGTCAGAAGCACATTGAAAGATATTTTACAATTGCCTACCGATATCCCTGATAGTACTCTAT
ATTCGCCTTGTCAAAACAAAGGTCTTGGTCTTTTTAAGGCAGCTTGGGAAATCTACCTCCAACATATCAATGCTTGTAACACTCTCCTCAAATCACA
GAACTCCCATGTCCTTCACTTGTGTACCTTGCAAGCGAATATCTCTACCTGTCTCCAACTCCTCAACATTACAAATACCGATCTACATATTAACAAC

CACACAGGCCTCATTGACACAAGGAAAATCCGAAACGAACTTCGACTTCGAGAAACCGAGAATTGGAGTCATCTACCTCACAAAGGAGTCGGTATTG
ATCTTTATAAGGAATATACTCCTGCCAACAGCTGGATAAGGCATCATAGAGGACTTTCTTGCAGTGAATGGCGCGAGGCAATCAAAATGCAAGCTAA
TGTATCCGCAGTGAGAACCGTTCCGGGCAGATCTCTAGACGGCAACCTCTGTCGCAGATGCAACAGAGAGAAAGAAACACTTGGCCACGTCTTGGGA
TCCTGTCCCTATGGTGAAATACTGAGGAATGCCCGCCATCATAAATTGCGATCCTTCATAGCTGAACAACTTCGCAGGAAGGGATATACCGTTTACG
AAGAAATCCATGGAATAGCTACTCAAGGCAGTACACGTCGAATTGACATTTTAGCCTTTCAACCAAACAGTACGAAAGGATATATAATTGACCCAAC
TATCAGGTTCGAACACCACACTAACCAACCTGCTGAGGTGAATGCCGAAAAACAGAACATTTATAATCCTACCATTCCATACTACAAAAGTCAATAC
CATCTACAAGACATCACAGTTATAGGGCTTATGGTAGGAGCCCGTGGCACTATTCCATCCCTGTTCGCCAAATTTTGGAGTGAATTCCACCTGCCTA
AGAAGAAAATCTATGATGTTGTCACCACTACCTTACGTGGATCTGTCCAAATTCTACGATGTCATCTCTTTGGTCCTTAAATATTCATCAACAGAAG
AATCACCAAAATTGCTACTTGATATTACCTTACAACACCTTGTACAAAATTGTAATTTTTATTTATATTTATGTATTTACAGCTATGTTATTTGTAA
CTCTAGTATATAATGCATACGTTATACTTTGTCCATAGTGGCAACTCGCAACTGCGAGAAGTTGTTGATTTCGTTCAATAAATATATATAATATATA
TTTAT

>ORF_Blag_2

MISDSGGPSLPSNDGKTIALVSCKICGKSFTRHGINIHIARSHRHSQVLIDIDKPTDSILSETVNFSPSDPIECQDNIHLNTNSSNNEKVIRNNSNA
SSIHLIDGSLIGTPSDSSPAVSQAQCPHCGGSFNGSKGVNIHISRSHPHIHRSTLVTNLTKNASQPLLDEQADRGSSSNSESSQPDSGFNIALNEWK
TKFVRTATANDSDFDNMVQDFSHFLSTSINLLPGPRHPATKYYEARRKRRLATAEKNYNKSSNPIRASKKAREKRKCKYDYQLTQYQFYNQRRKAVR
RVLDSTAPSCKIDLTKVHENFAASFSIENPHIRSNYPSKLTDVERIELDETYDPTISKEDIAIAAHKIAVDTAPGPDHVLVRVIRNEIVYEILAIIA
TRMLSSGIVPRCFKTARTVLIHKAADVNILSNWRPITICSVLRRVIERVLDKHLRTFVSFNEHQRGFTNNSGTLINTSILRKVLKTTKDNKEDVTVV
FLDISKAFDNIGHKHLDLTLSAEPIPAKLKDLIINLQINNTTNIQTSLGQTKAINLRRGVMQGSPLSPTLYNLATDFIFDELTELSLLQQYGKSISP
QLSSLSIMGFADDTLLMGKNSETTLELLHIALQRFQEIGLQVNPQKCTGISIKRGKLVEEVLHINEKFTIKTINNQEEVRYLGAPFQDNALFDKDST
IKQLHKKLELLASSPLLKSDQKFLVLNTAICPTLIYRFQTTDLQTIPLQFLHTADKLVRSTLKDILQLPTDIPDSTLYSPCQNKGLGLFKAAWEIYL
QHINACNTLLKSQNSHVLHLCTLQANISTCLQLLNITNTDLHINNHTGLIDTRKIRNELRLRETENWSHLPHKGVGIDLYKEYTPANSWIRHHRGLS
CSEWREAIKMQANVSAVRTVPGRSLDGNLCRRCNREKETLGHVLGSCPYGEILRNARHHKLRSFIAEQLRRKGYTVYEEIHGIATQGSTRRIDILAF
QPNSTKGYIIDPTIRFEHHTNQPAEVNAEKQNIYNPTIPYYKSQYHLQDITVIGLMVGARGTIPSLFAKFWSEFHLPKKKIYDVVTTTLRGSVQILR
CHLFGP

>Blag_3

TTGAGAAGGCTGTGGAATTATAAGTTTTTGATATTGCTATACTGGGACCTATTAATCTTTAATTATCAATATCTACTACATCTTTTGGAAATTATGT
ATATGGATTATATATTATATACTTAATAGCTGATGTGAAAGTATTAGTGTGGTTCCTTAACCAATATTATGTGAGAAATATTTTACTTCTTTTCAAG
GTATGAATTCGTTACTTTTTGTTTACATAGTAACGCCATTATATGCTTATTGTAATTAATTATTTTTGTAAAATTATTATATAAAGACAAAGT**ATG**C
CTCTTATTAATAATGAGGATCATTCTAATATTGTCACCATACTTTCTACAAGTACAGCTAATGATAATAATGGAAATTCAAATAGTTTGATTACCAC
AAATGCCATTACAAGCCAAGATCAAACTAAATTATGTACATTATGTGGTAAACGATTTAAATCTCTAGCCATTCATGTTAGTAAGGCCCATGGTGAA
GCTTACAGGAAAAAATTATCTCACAAGTATGAATGTCCGTTAGAAAATACTAAAGACACTGACACGGATTCTCCAAGCTCCATTAATCCTATAACAG
TTGCTGATGTTTCTTCTCCTGCAACTCAACAAATTTTGCAATTTGAAAAAGCACTTGAAGAATGTATATTATCATCTGATCTTAACAGGTTTGAAGA
AATTACTGTAGAAATAACAGCGTATTTATCTAAGGCTATAGCATCCCTCCCTGGACCAAAACATCCGGCTACACGTTACTATAATGCAAGAAAACTC
CGCCAAACAATTCATGGGACTTCATACTCTAATACAAAAAATCCTGCACGTTTATCCAAGCGAAAAAGGAAGGATAATAAACAAAAGTACCAGTATG
AAGTAGCACAATTCCATTATTTTAATCAGAGGCGTAAAGTAGTGAGACAAATTATGAAAAATCATAATAATGTACAATGCCAAATTTCTATCCCTAT
TATATTTCAACACTTTAACACATTGTTTAACAATGAAAATCCTTTTACGCCTATAGAGTCGATTCATCCAATGTCCACTGAAGAAGATATTGAAATC
ACTGAAGATGAAATCAAAGCTGCAATGGCAAAAATCAAAATTGACACTTCTGCTGGTATAGATCATGTCTTAGTTAGGACTATTAAAGAACTAAAAA
CATCTAAATTATTATGTTTGTTAACAAATGCTATGCTTAAATTTGAAATATGCCCTGGAATATTAAAAACAGCCAGAACTGTTCTGCTTTTTAAAGG
AGGCGATGAAACAAAACTCTCTAATTGGAGACCAATCTCCATTTTTTCTGTTATACGCCGCATTATTGAAAAAGTAATTGACAGACTACTAAAATCA
TATATAGAACTATCACCTCACCAGCGTGGCTTTATTAATGTCCCTGGTACTCACATAAATACATCCATCATTAACGGTTGCCTCACAAGAGCTAAAA
AGGAAAAGAGGAACTGTTGCATTGCCTTCCTCGATATTTCCAAAGCCTATGATAACGTTGGGCATGAACATGTGAAACGATGTTTACTTAGAACAAA
TATGCCAAATAATTTAAGAGACTTAGTGCTTAATCTTTTACAGGACAACAAAATTCAGATCGAATCTGGGTTCCAGAAAACTAAACCCATACAAGTT
CAGAAGGGAGTAGCACAAGGTTCTCCATTGTCCCCTGGTTTGTTTAACTTGGCTCTTGATGACACACTTAAAGAATTGGCTGATCCCAATGTTAGAG
AAGTCTTTGGGTTTTCCTTACATCCTGATCTTGAAAAGGTGTCTGTCTTAGCTTTTGCTGATGATATAGCCTTAATAAGTAAAGATGAGGCTTCTTT
GCAGACATTAATCAATATGACTGTTTTCAATTTAGATGCCATTGGTCTGAAAGTTAACCCTAAAAAATGCAAGTTAATTAATATTAAGAATGGCTTT
CTAGAACAAGGACAAATATTGGTTGGAGGTCAAACATTAGAATTTATAAAACCAGGAGAAATTATCAAATATTTGGGTGTTACTTTTGAAGATGAAA
TTGTGTTGGACAAATCAACTTTAATCAAGAATTTTGAGAATGATCTAAATTCACTAACGGTTTCCAATTTACTTACTCCAGATCAAAAATTAAATAT
TGTCAATCAATATATCTGGTCAAGGCTCATATATCCTTTGCAGTGTACTCCATTAGATAAAATTCCTCCTTCCTTTTTGCAAGATATTGATAAATTA
CTAAGGTCTTCTATTAAGGAAATCATTGGCTTACCAAGTGATTGCCCTACTGATATGCTGTATGCAGCGAGAAATGTCCGTGGATTGGGTCTAATTA
AAGCAGAATGGGAAGCTTCAATACAGCACTTCAACATTGCACGGACATTGTTAAACACGAATGATGACCATCTTCACTGCTCTAGAAATCTTGTAAA
AGAACAACAACAATCACTCGTCCAGCTAGACATTCCTTGCAATGAACAGGTCCTACAGAAGTCCAGTAGATGGCTAAGAAATCAGCTACGTACAAGA
TCCTATGAGGCTTGGTGTAAACTCCCACACAAAGGCAAAGGCGTTGAAGTGTTTAAACAAGTTCCAGATTCAAATTCATGGCTGGTTAATAGAATAG
GCCTTTCCACATCTGAATGGACTAACGCCATTAAGATGTCCTGCAATACAACAGCAGTAAGAACAATTCCAGGAAGATCTAAACACACCACAAAATG
TCGTCACCCTGGGTGTGAACATCAGGAAACTCTTGGCCATGTCCTTGGAATGTGTGCGAAAGGTGAATTATTAAGGAACACTCGTCATCATAAAGTG
CGTTCCTACATTGCCACTCACCTCCGTAAAGCCAATTGGGAGGTTTACGAGGAAGTACACTGTACATCGGAAGATGGAAGTCATAGAAGAGCAGACA
TTTTGGCAATTGATAGGCAAGCTGGACGTGGACTCATTCTCGACCCGACCATCAGAATGGAACAAGATGGTGAACAGGCCATTCGTGTTAACACAGA
AAAGGAGACGATTTATCGTCCATGCATACCTCACTTCAGTGAGAGATATAACTTACCTGTAAATGCATGGGCGGTGAAGGGTCTCCTCTTCGGTTCT
CGTGGCACAGCTTATCAATGGACAGCAGACATATTAAAGGGATTAGGTATGTCTGTTATAAATTTGAAACTATGCTGTTCAATGATTTTGAAAGATT
CTATTCAAATTCTCCAGAATCACTTATATAATCAAACT**TAG**ACAAAAAAATGTTTTTTTTAAATTATTTTCAACATTTTTAAATTCATTTTATTATCA
AAATTCATCTTGTTTTCTGGATCCAAGAGGTCTCCCGATAATTCGGGCAGATGTTCTTTTTGAACTTAGTAGT

>ORF_Blag_3

MPLINNEDHSNIVTILSTSTANDNNGNSNSLITTNAITSQDQTKLCTLCGKRFKSLAIHVSKAHGEAYRKKLSHKYECPLENTKDTDTDSPSSINPI
TVADVSSPATQQILQFEKALEECILSSDLNRFEEITVEITAYLSKAIASLPGPKHPATRYYNARKLRQTIHGTSYSNTKNPARLSKRKRKDNKQKYQ
YEVAQFHYFNQRRKVVRQIMKNHNNVQCQISIPIIFQHFNTLFNNENPFTPIESIHPMSTEEDIEITEDEIKAAMAKIKIDTSAGIDHVLVRTIKEL
KTSKLLCLLTNAMLKFEICPGILKTARTVLLFKGGDETKLSNWRPISIFSVIRRIIEKVIDRLLKSYIELSPHQRGFINVPGTHINTSIINGCLTRA
KKEKRNCCIAFLDISKAYDNVGHEHVKRCLLRTNMPNNLRDLVLNLLQDNKIQIESGFQKTKPIQVQKGVAQGSPLSPGLFNLALDDTLKELADPNV
REVFGFSLHPDLEKVSVLAFADDIALISKDEASLQTLINMTVFNLDAIGLKVNPKKCKLINIKNGFLEQGQILVGGQTLEFIKPGEIIKYLGVTFED
EIVLDKSTLIKNFENDLNSLTVSNLLTPDQKLNIVNQYIWSRLIYPLQCTPLDKIPPSFLQDIDKLLRSSIKEIIGLPSDCPTDMLYAARNVRGLGL
IKAEWEASIQHFNIARTLLNTNDDHLHCSRNLVKEQQQSLVQLDIPCNEQVLQKSSRWLRNQLRTRSYEAWCKLPHKGKGVEVFKQVPDSNSWLVNR
IGLSTSEWTNAIKMSCNTTAVRTIPGRSKHTTKCRHPGCEHQETLGHVLGMCAKGELLRNTRHHKVRSYIATHLRKANWEVYEEVHCTSEDGSHRRA
DILAIDRQAGRGLILDPTIRMEQDGEQAIRVNTEKETIYRPCIPHFSERYNLPVNAWAVKGLLFGSRGTAYQWTADILKGLGMSVINLKLCCSMILK
DSIQILQNHLYNQT

>Blag_4

AGGAGGCAGTGAAGTTGAATTGGCTTCTGTTGATGGGCTCTGTACATTTTCTATCATTTTTCATGTTTTCATGCGACTTGCTGCTTCGGAAGTGTTT
TCAATTCAAGTTTACATGTATATCTATTCAGAAACGTATTAGTGGTGGTAACTATTAATTATTTTGTGAAAATTTGTTTCATATATATTGAATACTT
GAAGTTGTTTATTTTACCTAGTAACACATTAGTTACGTGCTTATATGGACATTTATTGAAACTGAATAGGAAGAATTTTTATTTTTAATTATTTTTA
ATTATTTTTTTTTATTTATTTATTTATTTTTATTATTCATATACCCGTAAAGATCTAATTAGGTACTTCATTATTATTCATATTTTATTTATTGACT
TAATTAATTAACCTGTAAATCGTAACATAACCATTATATACCTGTAACATGTCAGAAATCCATAGTGCTGGAATGAGTTCTTTGCCTACCGCTTCTA
GTCAGAATAATTCTTCATGTCCATATTGTGGAAAATATTATCGTCGGTTAAGCACCCATATTACAAAATCACATCCTGAACAGCATCGAAAAAGTAT
CGTTATGAGAAATACAAGTTCTGTTTCTCCTGTCGTACAAACTGCTTCCAAGGATGATAATGTAAGTAATTCTTCTGCTAATACTAATTCAGATGTT
GGACATTTACATGAAATTAGATCTGAAGTGATGTCCTTCCGAGAAAAATTTTCATCTTTGCTACTTTCAGATCCTTGTCCTTCTAAATTTAATGAAA
TATACAATGACTTTACTGATACACTAGTTACCATTAATAGCAAACTACCGGGACCAAAAAACCCTTCTGTACGTTATTATGAATTACGACAACGGAA
CAAAAATATTTTTGCAGCCAATGATAGGAAATATAAAGATAGTAGCAACCCTCAGAAAAAATCCAAGAGGGATAGAGCTAAGCGACGTGAAAAATTT
CAGTATGAGCTTCTTCAATATCAATTTTATAATGAACGAAAAAAATGTGTTAATTCTCTATTAAATGATAATAAACGTAATGAACCTTGCAATATTC
CTATTGGAGTTCTAAAGGAAGCAATGGAGGAAAAATGGGGGACACTTAATACTTCTATGTTAGATCATTATAATAATTCTATGTCACCGCACTCACA
GCAGAATGATTTAGATCAGCTCTTTACAATTCCAGTGGAAGTTATTCAACAATCACTCAATAAAATGCGTACTGATTCAGCACCTGGACCTGATAGA
ATTTTAGTGCGAACGTTAAAATTAACCGAATGCTCTCCAGTTATTGCTGTAATTCTGAAAATCATGCTTCAATGGGGTATGGTTCCAGACAGAATGC
GGGGGGCAAGAACTATTCTAATTTATAAGGGTAAAGGCGAACGTTGTAGTGTATCTAGTTGGAGGCCTATTTCTGTGTGCTCGGTAATTCGCAGGTT
AATAGAGCGAGCGCTTGATTCAGTTCTTAGACAGTATCTTACTCTCAATAATAACCAAAGAGGATTCGTTTCTCAACTTGGCACATCTATTAACACA
TCTCTAGTTGATGGTTGTTTAAAGAAAGCAAAATTGGAACAACGAAATGTTTCTATCGTTATGCTTGACGTGGCACAAGCTTTTGATTCTGTTGGAT
TAGACCATTTAGAAAAATGTTTAGATTCCCAATTTCTTCCAACATCACTTTGTACTTTAATAAAAACGTTGACTAGAGGTAACTTTACACGGATCCA
TAATGGTAAACAAATTTCCGAACCCATTGAGCTTAAAAGGGGTGTTTTTCAGGGATCGCCCCTATCCCCAATTTTATTTAACCTCTCAATAGACTTT
ATTATTAAAGAGTTGTCTGAGAAACAAATTACTGATAAATATGGTTTTAAAATTTCTAATAATTTAGATCACTTGTCGGTGGCTGCATTTGCAGACG
ATTTGGTTGTTATAGGGCAAAATATGCATGCTGCCCGAGAACTAGTCATGATGACAAAATATTTACTTCAGCAAATAGGTCTTCAGCTGAACATTGC
AAAGTCCGTATCTATAAATATCGAACATGGAAAGTTACAGGACAAGGAATTACTTCTTTATAGTGGTTATGCTATTAGAGGCATTACAAAAGAGGAA
AAAATTAAATACTTAGGAGTTTCTTTTAATGATGAAATAATTTTTGACCGTGAGTCATTTATAACTAGTCTAAGAAATGATCTCCAAATTTTAGTAT
CAACTCCAATGCTTCGTCCAGACCAGAAGTTAAATATAGTCAATCAATATATATGGCCCAAATTTATATATGCACTCCAAATGGTTCCTGTACATAA
ACTGCCTCTAACATTTTTGGAAGATGTAGACAAATTAATACGTAGCGCCGTAAAGGAAATGCTGCTTTTACCATCAGATGTACCAAATAGCATGTTA
TATTCGGCAAAACAATTTCGGGGTCTAGGACTAGTACGAGCTTCTTGGGAAGCATTTCTCCAAAATTGTAATACTGTTATAAATTTATTACTGGCTG
ACAATGCATATGTGAATGCTGTGAGACCTTTATTAACCGAACTCCAGTCATCCTGCAAACACCTTGATATGGTATTTCCATCAGAGCTTCGAAAATC
AATAACACGATCATTTCGAGAAAAACTTCGTCGTCGGGAATTCGAACTTTGGAGTAATTTACCAGGGAAAGGAAGAGGTGTTGTTTTGTACAGTGAA
GTTCCAAAAGCCAATAAATGGATTGCCAATAAATCAGGACTATCTACTTCAGAATGGGTCTCTTCTTTGAAGATGAATGTCAATGTTGCGGCCGTAC
GGGCAATTCCCGGCAGGTCTTTGGACGGATCCCGGTGCAGATATGGCTGCCCGGAGACCGAAACTCTTGCCCACGTCCAAGGCCAATGCGATCACGG
GTTGCTACTGCGTAATGCTCGTCACCACCAAGTACGGTCTCTCATTGCTTCTGCTTTAAGAAAGAAAGGTTGGGAGGTAGAAGAAGAAATTAGTTGT
ATAGCAACAAATGGCTCCACTAGAAGAGTTGATATATTGGCGTACTCCAAGACCACGAGAATTGGTTATATTATAGATCCTACCATCCGATATGAGA
AGAATGCCGATCAATCTGAAGAAGTTGATCAGGAGAAAAAAATAATATACGAACCAACAATACCATATTTTAAAACCAAATATGATCTCAACAGCTT
GGAGGTCATTGGACTTCTAATAGGTGCCCGAGGTGTTATTCCAAATTTTTTTAGTAATTTCCTAAAACAATTTGATTTACCAAAAACTTTATTAGAA
GACATTGTAGTAATAGTTTTGAAAAGATCCTGCCAAATTTTAAGTAATCATTTACGTTTTTAACATTTATAAAATTTAGTTCTATTAGTCTTATGA
TTATTGTTTTATTGTTCTTATTGTTTTATATATACTGTATTTATGTTTTGTATACTGAGGCAAAAGTGTCTACCTCTGTTGGGAGGCAGTATTGAAT
AAATAAATA

>ORF_Blag_4

MSEIHSAGMSSLPTASSQNNSSCPYCGKYYRRLSTHITKSHPEQHRKSIVMRNTSSVSPVVQTASKDDNVSNSSANTNSDVGHLHEIRSEVMSFREK
FSSLLLSDPCPSKFNEIYNDFTDTLVTINSKLPGPKNPSVRYYELRQRNKNIFAANDRKYKDSSNPQKKSKRDRAKRREKFQYELLQYQFYNERKKC
VNSLLNDNKRNEPCNIPIGVLKEAMEEKWGTLNTSMLDHYNNSMSPHSQQNDLDQLFTIPVEVIQQSLNKMRTDSAPGPDRILVRTLKLTECSPVIA
VILKIMLQWGMVPDRMRGARTILIYKGKGERCSVSSWRPISVCSVIRRLIERALDSVLRQYLTLNNNQRGFVSQLGTSINTSLVDGCLKKAKLEQRN
VSIVMLDVAQAFDSVGLDHLEKCLDSQFLPTSLCTLIKTLTRGNFTRIHNGKQISEPIELKRGVFQGSPLSPILFNLSIDFIIKELSEKQITDKYGF
KISNNLDHLSVAAFADDLVVIGQNMHAARELVMMTKYLLQQIGLQLNIAKSVSINIEHGKLQDKELLLYSGYAIRGITKEEKIKYLGVSFNDEIIFD
RESFITSLRNDLQILVSTPMLRPDQKLNIVNQYIWPKFIYALQMVPVHKLPLTFLEDVDKLIRSAVKEMLLLPSDVPNSMLYSAKQFRGLGLVRASW
EAFLQNCNTVINLLLADNAYVNAVRPLLTELQSSCKHLDMVFPSELRKSITRSFREKLRRREFELWSNLPGKGRGVVLYSEVPKANKWIANKSGLST
SEWVSSLKMNVNVAAVRAIPGRSLDGSRCRYGCPETETLAHVQGQCDHGLLLRNARHHQVRSLIASALRKKGWEVEEEISCIATNGSTRRVDILAYS
KTTRIGYIIDPTIRYEKNADQSEEVDQEKKIIYEPTIPYFKTKYDLNSLEVIGLLIGARGVIPNFFSNFLKQFDLPKTLLEDIVVIVLKRSCQILSN
HLRF

>Blag_5

TAAGAAGGCCATGACTTGAGTTTGCTACTATTCTATGTGCATCGCAACTTTTTTACAATTTTACCTGCTTATCTACTATTGAATCGTTGAACTTTAT
TACCAGAGTTTTTGTGATATATTGACTGTTTGCTACAAATATTAGGATGATTTTATTTTGAGTATCTTGGATATAAGTATTGAAAATTCATTTATAC
TGACTGTTTACCTTAGCAACCTGCTTCCCAGGTTTGAATCAATTTCATAAATTTTAAGTTATAAAC**ATG**ATACTATCAACTGATGAAGAATCAATTA
CGCCTATAAATACCTCTGTGTCTACCAATACCGAAATATCCAGCGTAGCCAGTGAAGTACCATGTCCTCATTGTGGAAAATTCTTTGTGAACTCAAG
AGGTATTTCGATCCATATAAGTAAGTCTCATCCTGATTTACATAGACTTAAGCTTGTTGAACGACAAACTACAAGTTCCTATACTATTTGGAATTCT
GGTGAATCATCGTCAACTTGTAAGACGAGTATTAATGACACTCCTTCTAAAAGCCATCCAGTGTCTGTGAATGATCCATTATCCAATTACAAACAAC
AGTTACTTACATGGACAACCAAATTTGATTGTGACTTAACCGACATTGATTTTGATACTTTAACGAACAATTTTTTACAATTTCTTTCTGAAGCCAT
ATATTATTTACCTGGGCCTAAACACCCAGCTAGAAAATATTATGCTCTGAGGAAGAAAAACAAAAATTTCAATTGTCAACAATCTTACAAGAATTCT
TCTAATCCGGACAGGGCCACAAAAAGGGATAGAGCAAAACGAAAACAAAAATATCAATATGAACAAACACAGTTTGACTACTATAACCAGCGTCGGA
AAGCGGTCAGAAGAGTTCTCACTTCAGACAAGCCATCATGCCAAATAGCAGTAGATAAAGTGTACGCACATTTCAATTCCTACTTTAGTCGTCCGAA
CAACTGTATACGACCAGATTACATCTCCCATGTTACTGAAGTCGAACGGGTTGAACTAAATGACTCCTTCCCGACTCATATTACAAAAGAAGAAATT
AAATTTGCAATATCAAGGATAGCTGTTGATACCGCTCCAGGTCCGGACCACGTCCTAATGCGGGTGTTAAAAGATGACGCTGCTACTGGGGTCCTCT
CACTTCTTGCTACAAGAATGCTACGAACAGGACATGTTCCTCCCTGCCTCTTAATTGCCCGAAGTATCCTTATCCACAAATCTGGCGATGTCAATGA
CATTACTAACTATCGACCTATTACCATCTGTTCAGTGGTGCGCAGAGTTATAGAAAGAACCCTGGATCGACGTCTTAAGGAATATGTTTCTTTCAAC
CGCCATCAAAGAGGGTTTACTTCATCCCCTGGGACGCTCATGAACACCTCTCTTCTCAGATCCATCCTCACCTCAGCAAAGACCAAGAAATGTAATG
CTACTCTAGTATTTCTTGACATTCGAAAGGCTTTCGATAATGTAGGTCATTTACATCTCCAAAAAACCTTAGAAAGCCTACCTATTCCTAGTCTGCT
CTCTAATTTAATACTCAACTTACAAGAAAACAACCACACTAGATTTGAAATTAATCACCATAAAACCAATCTTGTACCTCTAGCGAGAGGTGTTATG
CAAGGATCACCCCTCTCACCTACGTTGTATAATCTTTCGACCGATCACATTTTGAATGAACTTTCCGAAGACGGTGTCGCCAAAGCATATGGTTATC
AACTTGTCCCGGGGTTAAAACCCGTAACCGTTCTCGGATTTGCAGATGATACGGTAATAGTCGCTAATAGTACAACATCGGCCCTTGAATTAACTAA
AATGGCGATAGAACGTTTTCGAAGTATCGGTCTAGAAATTAATTTTTCAAAATCCGTGGCTATTAATATATCTTGTGGCAAACTTAACCCAAATTAT
TCCTTATTAGTTGCAGAGAATGCCACTATACCTTGTCTAGGTCCAAATGAGTTCGTTCGTTATTTAGGTGTGAATTTTGCTGACGCCTGTTTGTTTG
ATTCCCAAGTGACTCTCAACGATTTGAAAAATAAATTAGACCTCTTGATTTCATCACCATTGTTAAATCCCGACCAAAAGTTCTGTATATTAAATTC
ATCAATTTGTCCTTCCTTAATATACACTTTTCAAACAACGCCCTTAAACAAAATCCCCAATAAATTTATCGAGGCGGCAGATAAAATAATTAAAAGC
GGATTGAAAGAAATTTTACAACTTCCAGCTGATATACCGGACTCCATGATTTATACCCACCGAAAATTTAAAGGCCTCGGACTGTTTAGAGTAAAAT

GGGAGTCCGCTTTGCAACATATTAATAGCCTCAGAATCCTCCATCGGGCAAATGATGCTTACATATCATCCACACGAAATTTATATGATGAAAGCAA
ACACTGCTTAACTGAGCTAAAACTAAAATTTAGTGACTCACTTCTGAATAAACATGATAACTTTCTCGATTCTCGTAAAATACGTGAAACTTTACGT
GACCGTGAGTTCAATACTTGGTGTAGTTTACAAAGCAAAGGTAGAGGGGTGATTCTTTTTAAAGAGTTCCCTCCATGTAATCAGTGGATAACCAATC
ATGAGGGTCTGTCAAGTTCCGAATGGAAAGACGCTATAAAAATGGTCGGTGGTGTTGCTCCTGTTCGAGCCGTGCCGGGTAGAAGTCGAGACACTAA
CCGTTGTAGGCGTTGCCTAAACGAGATTGAGACACTTGCTCATGTTCTTGGCTCCTGTCCCCATGGTGAGAATTTGAGAAACATACGCCACCATACA
ATTCGTTCCATTTTGGCTGCCGCTTTACGGGATGTTGGTTTTATTGTTCATGAGGAGGTTCAGGGTTTGGCCACTCAGGGTAGCATTAGGCGTATTG
ATATCATAGCCATCCGTAACAATACAGCTTACATTCTCGACCCCACTATCCGCACTGAGACTCATGCAGAACAACCACGTGAGGTAGACATGGAAAA
GAGGGCGATCTATGAGCCTACAATACCTTATTATAAAGAAAAATACCATCTATCAACAATCGAGGTTGTAGGTTTAATGGTGGGAGCTCGTGGCACA
ATCCCATCCCATTTCGCGGAAATCTGTAAAAGGTTGGGTCTTTCTTCCTTTATCATTAAATCAATAGCGGTCTCAGCTCTCAAGGGTTCTATTTTGC
TCTTGAGACATCATTTATATGGC**TGA**ATAATTGTGCCTTTTTTTTAGAAAAATTTTAAGTACAGGTATTTTCTAGTTGGGTCAGTATACATTCAAAT
TTTATACAATTTTATATAGTGCATTGTTTAGATGGGTATTGCATTTTTTACATATTTTAAATTTATAAATGTTTGGTACGCTTTGTCTTATTTGGCA
CCCTTAAATTTAAGGAAGCTATTAAATTTAA

>ORF_Blag_5

MILSTDEESITPINTSVSTNTEISSVASEVPCPHCGKFFVNSRGISIHISKSHPDLHRLKLVERQTTSSYTIWNSGESSSTCKTSINDTPSKSHPVS
VNDPLSNYKQQLLTWTTKFDCDLTDIDFDTLTNNFLQFLSEAIYYLPGPKHPARKYYALRKKNKNFNCQQSYKNSSNPDRATKRDRAKRKQKYQYEQ
TQFDYYNQRRKAVRRVLTSDKPSCQIAVDKVYAHFNSYFSRPNNCIRPDYISHVTEVERVELNDSFPTHITKEEIKFAISRIAVDTAPGPDHVLMRV
LKDDAATGVLSLLATRMLRTGHVPPCLLIARSILIHKSGDVNDITNYRPITICSVVRRVIERTLDRRLKEYVSFNRHQRGFTSSPGTLMNTSLLRSI
LTSAKTKKCNATLVFLDIRKAFDNVGHLHLQKTLESLPIPSLLSNLILNLQENNHTRFEINHHKTNLVPLARGVMQGSPLSPTLYNLSTDHILNELS
EDGVAKAYGYQLVPGLKPVTVLGFADDTVIVANSTTSALELTKMAIERFRSIGLEINFSKSVAINISCGKLNPNYSLLVAENATIPCLGPNEFVRYL
GVNFADACLFDSQVTLNDLKNKLDLLISSPLLNPDQKFCILNSSICPSLIYTFQTTPLNKIPNKFIEAADKIIKSGLKEILQLPADIPDSMIYTHRK
FKGLGLFRVKWESALQHINSLRILHRANDAYISSTRNLYDESKHCLTELKLKFSDSLLNKHDNFLDSRKIRETLRDREFNTWCSLQSKGRGVILFKE
FPPCNQWITNHEGLSSSEWKDAIKMVGGVAPVRAVPGRSRDTNRCRRCLNEIETLAHVLGSCPHGENLRNIRHHTIRSILAAALRDVGFIVHEEVQG
LATQGSIRRIDIIAIRNNTAYILDPTIRTETHAEQPREVDMEKRAIYEPTIPYYKEKYHLSTIEVVGLMVGARGTIPSHFAEICKRLGLSSFIIKSI
AVSALKGSILLLRHHLYG

>Blag_6

ATTACGTTCACAAGAGCTGTGCTAAGCTTCAAAAAATGTGAAATTCATCGATAAGAATTTAATTCAGTGCTGTGAAAATACCGTTGGTAATTCGATC
AGTGAAGGTGAGTGCTGTGTAAAAGATGACGTCGCGAATGCGACCCCGCTGTTACTTAAACATCATTATACTTGTTGATATCTTCAAACTACTACAA
TTTATATTGGATTTTCTCCGTGAAGACTGACTTCTCTTCTTAGACATTGTGTTACTC**ATG**ATATCAGATTCTGGAGGACCTTCGTTACCATCTAATG
ACGGTAAGACTATTGCTTTAGTCTCTTGCAAGATATGTGGAAAAAGTTTTACTAGACATGGAATTAATATCCATATTGCTAGAAGCCATAGACATTC
CCAAGATTTGATAGACATTGATAAGCCTACTGATTCAATTTTATCAGAGACTGTAAACTTCTCACCTTCTGACCCTATTGAATGCCAGGATAATATC
CATCTTAATACTAATTCATCTAACAATGAGAAAGTCATTCGTGCAGTCAATAATGATGAGAAAGATAAAAGTGATAACAATATTATTAAAAATACAT
CTTCAGATATCTGGTGTCCTTACTGTAGTAAAGTATTCAAAAATTTAAATGGCCATATAGCACGATCACATCCCCATATTTATAGAAAAAATATTAC
CGAACGTTATGCCAATGCTTCTCCTTGTCTGCAAAAAACAGAAATTAATAATAATAGCGGTCAACTTAAAATAAATGAAAATAAGTTAGAGCCTTGG
GAAAAAACATTCTCTGAAATTCTTAATAGCGAAAAAGATGAAGTGAAACTAGACCAGAAGTTAGATGAATTTACCTTATTTCTATGCAATGCCACCT
CTCTGTGCAAAGGTCCGGTACATCCTGCTACAAAGTATTATAAAGCTAGAACTACATGGAGACCAAATCATGAGAAGTCTAATTTTAAAACATCCAA
TAATCCTCAAAGACATGATAGGCGTTTGAAGGAAAAGAGACAAAATCAATACCGATATGATCTTATGCAATACTATTATTATTACCGAAGAAAAAAG
GCAGTGCAAAAAATCATGCATCTTTCAGATTACAAACGATGTCCATTCTCTGCAACAGATGTTTATGACTACTTCAAAGAAACAGTGGGAACAGAAA
ATAATAATGTCTTGCATGATTATTCAATCTCATCTCCTTTGTGCAATTCTATAGATGAGGAAACAATAGACTATGATTGTCAGTATTGTACTGTAAC
AGAAAAAGACATTGATCAAGCAATGAAAGGAATTAATCTTGATACTGCCCCTGGCCCCGATAGGGTTCTTTTCAAGGTCGTCAAAGAACTTAAATGC
ATGAAGATCATCAGCATAATTGCTAACATCATGTTAAGCTGGAACTATGTGCCCAACTCTTTTCAATCGGGTCGCACTATCTTGATCTTTAAATCCG
GTGATCCAAATTCATTGAAAAACTGGAGACCAATAACAATTTTCTCAATTCTAAGAAGAATTATAGAAAGAGCATTAGAGAGAAAAATCAGACCATT
TGTGGAATTATCAACTTGTCAGCGCGGTTTTATATCAACACCAGGGACTTTTATAAATTCCAGCTTAATAAATAGTTGTCTTCAGAAATCTAAGAAA
GAAAAGGGTGACTGTTGTATTACTTTTCTTGATGTATCTAAAGCGTTTGACTCTATTGGTCACAAGCATATTGAAATGAGTTTGAACTCTACATCAA
TTCCAAGCCAATTAAAATCCCTAGTTCTTTCTCTAGTAAAAAATAACTCAGTTGGGATTGAGTTAGATGCAACAACGCGCACTAAGCCAATAAATAT
AGAAAAAGGAGTAGCCCAAGGTTCCCCATTATCTCCTCTTCTCTTCAACTTGTCCATTGATTTTATTTATCGCAACTTGAATGATTTTCAGAATGCA
TCTCAATATGGTTATCGCCTTACCGAAGAGGTAGACAACTTAATTGCTCTTGGTTTCGCAGATGATATTGCTTTAATATCTAATAATGAAACATCTG
CTGTTATTATGATTGAAAGAGTAAACCAATGCCTAACAGAAATTGGGCTCAATTTAAACGCTAGTAAATCTAAAATTATTAATATCAAGAAAGGGAA
ATTAATGAATACCACAGTTACAACATTGAATGACATTAAGATCAATTCTATACAAGCGGAGGAGACCATGATATATTTGGGAGTTACATATAATGAT
GAAATTATATTAGATAAATGTTCTATCATAACTAACCTAAATCGTAGCATATCAAAACTTATCACCTCTTCGCTTCTGAAACCAGACCAAAAACTCA
ACATCTTAAATCAATATATATGGCCAACTCTAATTTATCCACTTCAATGTGCTCCTCTCTGCAAACTTCAACTTGGGTTTTTACAAGATATTGATAA
AATTGTAAAAAATGCAGTTAAGGAAATTGTAGGACTTCCTTGTGACACTCCTGATTCGATGTTATATACCTCTCGAAAATATCGAGGGTTAGGTGTG
ATGAAAGCGACATGGGAAGCTTTCCTCCAACATATTAACATCTGTAATAAACTGCAAGTGTCTTCCGATCCTGTTATTGGATTCACAAGAAATTTTA
ATAATGAGATACAGGAAAGTTTAGATCATCTTAACATAAGCAGAGAAGAACATCCCAATGTCCGCTCTCTTCGGAAAAAACTCAGAGAAAAGGAATA
CGAAAACTGGAAGAGTTTGCCTCATCGTGGCCGAGGAGTAGCCCACTTTTCAGAATGGACTCATTCTAACACCTGGATTTCTTCAAAGAAAGGTTTG
TCCTCCTCTCAGTGGACAAATGCAATTAAAATGGATTGTAATGTCATTCCTGTCCGTAGTCTCCCAGGCAGAAGCGAAGAAACCCGTTGCCGTCATT
GCAACGAGTCAGAAACCCTTCCCCATATTCTTGGTTTTTGCAAAAAGGGAGAATTACTGCGTAACAACAGGCATCATAAGGTCAGGAGTGCCATTGC
TAAACTTCTTCTAGAGACAGGCCATTTTGAAATATTTGAAGAAATACATTGCATATCCACTGACGGTTCTACGAAGCGTGCTGACATCATCGCAATT
GATAGAAAACGTGATTTAGGATATATACTGGATCCCACGGTCAGATTTGAGAAAGACCTATCTCAAGCTCATCTAGTTAACGAAGAGAAAAAGAAAC
ACTACCAACCGTGCTGCAAATACCTTTCTGAGAAATATAAAATCAACACCTGGGAAGTAATAGGTTTATTATTTGGCTCACGTGGTACAATAACCAA
ATTTTGCGTGAATATATTACTACATTTCGGCATAAAGAAAAAAAAGTCTACAGGAAATTCTACTTGCCATACTGAGAGATTCAGTACACATCGTACA
TTTTCATCTGTATGGTCACATGAATATCTATAC**TGA**ATAAATAAATTATCCAGTTATAAGAAAGCTGCCTTTTTCACATTTTAATATAAACAAAATT
CATAACAGTTATTTATTTTTTTTTTCTTTATTTATTAATTTTTTCAGCAAAAGAAAATTAATATCCCTAATCTAAGTACCTATTATAAAATTATTGT
ATTTATTATTGATTGGCAGGCTGTACCTTTTGGTAACCCTTAATAAAGGGCAGCTATCCTATTTTTGGATTTTCTCTCTC

>ORF_Blag_6

MISDSGGPSLPSNDGKTIALVSCKICGKSFTRHGINIHIARSHRHSQDLIDIDKPTDSILSETVNFSPSDPIECQDNIHLNTNSSNNEKVIRAVNND
EKDKSDNNIIKNTSSDIWCPYCSKVFKNLNGHIARSHPHIYRKNITERYANASPCLQKTEINNNSGQLKINENKLEPWEKTFSEILNSEKDEVKLDQ
KLDEFTLFLCNATSLCKGPVHPATKYYKARTTWRPNHEKSNFKTSNNPQRHDRRLKEKRQNQYRYDLMQYYYYYRRKKAVQKIMHLSDYKRCPFSAT
DVYDYFKETVGTENNNVLHDYSISSPLCNSIDEETIDYDCQYCTVTEKDIDQAMKGINLDTAPGPDRVLFKVVKELKCMKIISIIANIMLSWNYVPN
SFQSGRTILIFKSGDPNSLKNWRPITIFSILRRIIERALERKIRPFVELSTCQRGFISTPGTFINSSLINSCLQKSKKEKGDCCITFLDVSKAFDSI
GHKHIEMSLNSTSIPSQLKSLVLSLVKNNSVGIELDATTRTKPINIEKGVAQGSPLSPLLFNLSIDFIYRNLNDFQNASQYGYRLTEEVDNLIALGF
ADDIALISNNETSAVIMIERVNQCLTEIGLNLNASKSKIINIKKGKLMNTTVTTLNDIKINSIQAEETMIYLGVTYNDEIILDKCSIITNLNRSISK
LITSSLLKPDQKLNILNQYIWPTLIYPLQCAPLCKLQLGFLQDIDKIVKNAVKEIVGLPCDTPDSMLYTSRKYRGLGVMKATWEAFLQHINICNKLQ
VSSDPVIGFTRNFNNEIQESLDHLNISREEHPNVRSLRKKLREKEYENWKSLPHRGRGVAHFSEWTHSNTWISSKKGLSSSQWTNAIKMDCNVIPVR
SLPGRSEETRCRHCNESETLPHILGFCKKGELLRNNRHHKVRSAIAKLLLETGHFEIFEEIHCISTDGSTKRADIIAIDRKRDLGYILDPTVRFEKD
LSQAHLVNEEKKKHYQPCCKYLSEKYKINTWEVIGLLFGSRGTITKFCVNILLHFGIKKKKSTGNSTCHTERFSTHRTFSSVWSHEYLY

Supplementary Figure 2

Structural of the non-site-specific R2 retrotransposons of *Blattella germanica*.

The open reading frames are highlighted in yellow, and the nucleotide sequences without background highlighting correspond to 5'- and 3'- untranslated regions.
