# Supplementary material for: R2 and Non-Site-Specific R2-Like Retrotransposons of the German Cockroach, Blattella germanica
Source: Genes (Basel). 2020 Oct 15;11(10):1202. doi: 10.3390/genes11101202 (PMC7650587; doi:10.3390/genes11101202)
Supplement: Supplementary file 1 [file genes-11-01202-s001.zip › Supplements/Supplementary Figure 3.pdf]

Blag 1

5’

```
Blag 1_01  ttaaatggcaataattgactcaactgcagatgctaaactagtacaattcaatcaatcaat
Blag 1_02  -----------------------------------------ataaatttaaatttaaaa
Blag 1_03  --------------------------------------------tgtctttatttttct
Blag 1_04  ---------------------------------gagcttatcctattcatttattgttt
Blag 1_05  ------------------------------accccatatgtggggattgccaaatgtca
Blag 1_06  ------------------------------gagaaacaaatcagatttctgtactactt
                                                      *.      *

Blag 1_01  gttctttatttgcctttgagtataaatatacaattggcatcgtccatagaatta------
Blag 1_02  aacttgaatctgtatttagatttcaagattaaagatctagtttcattggaatca------
Blag 1_03  gttatgaagatctatctaagtgtgaagtttcaaggtacttatatagtggcat--------
Blag 1_04  gtttattatttgttatttattatcacttgagtactaaccttgtttacaga-----agatg
Blag 1_05  caga-ataataaatattagattttccttcattaaatgaaataaaattaaaagaaaaatt-
Blag 1_06  actc-tcagcagttgtgaaatatcctgtgaatattaggaatatcatttgatatatactta
                  *       .  . * *         *             . .

Blag 1_01  -----------------------caatatattttataaatctatttcacattaaaaaaat
Blag 1_02  -----------------------atttcaaaactgggtaactaatattcatgaaaacact
Blag 1_03  ----------------------------gttgtcaaatagttctcttgtaagtcaccaac
Blag 1_04  ttgaaagtggtggccatctgcattgatgcattcttgacatctctttagtt--ttaaaatt
Blag 1_05  ---aaatcaatttaaacattggtaggaccatacaaatggctgcgaaaaatcatgaccgag
Blag 1_06  ttgaattttattctgaaataattagtgctatattggacattgaaattgtttattggtaac
                                           .     . .             .  .

Blag 1_01  tcgtccatgttg-----tgaaaaggattttcatcaaccaaccagagatcttggttttga
Blag 1_02  atggacataatatggtttgatgtggaattaaatttcaaattttgaacttatttgaaatgg
Blag 1_03  atttttatatttacactaactagggtttccacctttacaattctcaattctcgaacataa
Blag 1_04  ttagtagccgta-----tgaactaaggactttgaaacacttacttcttgtgcaacacgtc
Blag 1_05  ta---tgttttc-----agtgcaaaattacatctacaatttcttaatttcctcatccttg
Blag 1_06  taagtcgttttc-----agtgcaaaattacatctacaattccttaatttcctcatccttg
                 ..  *       .    ..                 .        . .

Blag 1_01  aatagggttg-ccaggtatttggaatttcaatacgggactggtcataacaaggcatttc
Blag 1_02  atagaattgtcttacctaacgaag--------gcataatacac---aggtcaatattta
Blag 1_03  aaaaaagacattttaagaattgaatgatttctgtagaactgct---aaattcaaattgta
Blag 1_04  taagaggcttatttggagagcgagtgaatgatgcatgaatttc-------aacgagtttt
Blag 1_05  catatggctatctttcgaggcaaaattacaaaggaggaatt----------gtgatttg
Blag 1_06  catatggctatctttcaaggcaaaattacaaaggaggaatt----------gtgatttg
              . .     ..    .   ...        . . .*                * * *

Blag 1_01  tcctttgatagtgtc---------------------atggaatgtctgacaacaactatg
Blag 1_02  ttacttgtcccattc----------------tgtatacgtgatatctgactgtgagttct
Blag 1_03  tccgttgtaaagttga----------aggccgtgatacgtgatatctgactgtgagttct
Blag 1_04  tcctcatttaggaccctt---------ctctttggcttcggacatttagcgtttagggat
Blag 1_05  atggaagtgaaaatcactgaagagaaatttatgaatatttattatttattagcaagtggt
Blag 1_06  atggaagtgaaaatcactgaagagaaatttatgaa-------tatttattagcaagtggt
            .           .                            ..*.*.  .  *

Blag 1_01  --atcaaaattaagtgtgacttt--atcagagttaatgaacatctttaccttccatcgtt
Blag 1_02  aaagttaaagtgattgtgaatgcaaatacaatctgtttgacaatgtcagaaaactgtttg
Blag 1_03  aaagttaaagtgattgtgaatccaaatacaatctgtttgacaatgtcagaaaactgtttg
Blag 1_04  --cccgttgttcttaatt--ttt--taataagacgcctgacagtctttaca---tttacg
Blag 1_05  --accggtactacactttattc--ttgtatgcagttggacatccgtttt----------
Blag 1_06  --accggtactacactttaattc--ttgtatgcagttggacatccgtttt----------
               .   . *     *   * .      .    . . .*** .  .

Blag 1_01  ccatttgtttgaattggaaaaaaaaatactttcaacgtgtatggtatagttaagaaaata
Blag 1_02  ttaattattggcttcagtgaaatatcttttaatagtgtgtatggtatagttaagaaaata
Blag 1_03  ttaattattggcttcagtgaaatatcttttaatagtgtgtatggtatagttaagaaa-ta
Blag 1_04  gcatttacttggtaaccaaacgaagaccttatcaaaaactgaaattt---------aaga
Blag 1_05  -catttacttggtaaccaaacgaagaccttatcaaaacctgaaattt---------aaga
Blag 1_06  -catttacttggtaaccaaacgaagaccttatcaaaaactgaaattt---------aaga
            .* **..* *       .* . *  . .*  .*. .  *. ..* *         *  *

Blag 1_01  atgtgct-tattcagatataaactatgcttaactaaggtaaatgttttatcaatattgctg
Blag 1_02  atgtgct-tattcagatataaactatgcttaactaaggtaaatgctttatcaatattgctg
Blag 1_03  atgtgctttattcagatataaactatgcttaactaaggtaaatgctttatcaatattgctg
Blag 1_04  ttttccgtattcatattgaatttctttt------------gtcctctaccgacat------
Blag 1_05  ttttccgtattcatattgaatttctttt------------gtcctctaccgacat------
Blag 1_06  ttttccgtattcatattgaatttctttt------------gtcctctaccgacat------
                . ****** **  ** .* * .*            .* .*.**.*.*.**

Blag 1_01  tgaagtgagaaattgccataattgactgattttgaactgaattaacacattttaggccta
Blag 1_02  tgaagtgagaaattgccataattgactgattttgaactgaattaacacattttaggccta
Blag 1_03  tgaagtgagaaattgccataattgactgattttgaactgaattaacacattttaggccta
```

```
Blag 1_04  ------------------------gctactgttggtaaatattaagtattgtatggccta
Blag 1_05  ------------------------gctactgttggtaaatattaagtattgtacggccta
Blag 1_06  ------------------------gctactgttggtaaatattagatattgtacggccta
                                   .**. * ***.   . ****.    * *  ******

Blag 1_01  tgcctatatctgaccaagtttcattaaacgtatgaataccgaaacaaacgagcaatccga
Blag 1_02  tgcctatatctgaccaagtttcattaaacgtatgaataccgaaacaaacgagcaatccga
Blag 1_03  tgcctatatctgaccaagtttcattaaacgtatgaataccgaaacaaacgagcaatccga
Blag 1_04  tgcctatatctgaccaagtttcattaaacgtatgaataccgaaacaaacgagcaatccga
Blag 1_05  tgcctatatctgaccaagtttcattaaacgtatgaataccgaaacaaacgagcaatccga
Blag 1_06  tgcctatatctgaccaagtttcattaaacgtatgaataccgaaacaaacgagcaatccga
           ************************************************************

Blag 1_01  ccatactactatcaacgtcgatttctcacacgctgaagtaggcctatc
Blag 1_02  ccatactactatcaacgtcgatttctcacacgctgaagtaggcctatc
Blag 1_03  ccatactactatcaacgtcgatttctcacacgctgaagtaggcctatc
Blag 1_04  ccatactactatcaacgtcgatttctcacacgctgaagtaggcctatc
Blag 1_05  ccatactactatcaacgtcgatttctcacacgctgaagtaggcctatc
Blag 1_06  ccatactactatcaacgtcgatttctcacacgctgaagtaggcctatc
           ************************************************
```

3’

```
Blag 1_01  tttgtgtttccgtcccaatca-tttaacaactttcagtttgccattatattatgctttgt
Blag 1_02  tttgtgtttccgtcccaatca-tttaacaactttcagtttgccattatattatgctttgt
Blag 1_03  tttgtgtttccgtcccaatcatttaacaactttcagtttgccattatattatgctttgt
Blag 1_04  tttgtgtttccgtcccaatca-tttaacaactttcagtttgccattatattatgctttgt
Blag 1_05  tttgtgtttccgtcccaatcatttaacaacttccagtttgccattatattatgctttgt
Blag 1_06  tttgtgtttccgtcccaatcatttaacaactttcagtttgccattatattatgctttgt
           ********************* ***********.**************************

Blag 1_01  ccactgtggcagtcccttagttgggaaagccgaaaggttcactttctaaataaaaaaaaa
Blag 1_02  ccactgtggcagtcccttagttgggaaagccgaaaggttcactttctaaataaatatgaa
Blag 1_03  ccactgtggcagtcccttagttgggaaagccgaaaggttcactttctaaataaataaaaa
Blag 1_04  ccactgtggcagtcccttagttgggaaagccgaaaggttcactttctaaataaaataaat
Blag 1_05  ccactgtggcagtcccttagttgggaaagccgaaagattcactttctaaataaaaatagt
Blag 1_06  ccactgtggcagtcccttagttgggaaagccgaaaggttcactttctaaataaaaatatt
           ************************************.*****************   .

Blag 1_01  aataaactaggc-------------agaaatgtttgcgctatgtgaagaaaatgaagtga
Blag 1_02  aataatatgaat-------------acataaatttctcctcccatcttcttaaataatta
Blag 1_03  atacagggttaa------------tttattattattatttaacaaaatcacagaaattaa
Blag 1_04  a------------------------tcgtatctttcactgcccattgttgaatttaaata
Blag 1_05  caataatatgga------------ttcttgaagatttt------------------atga
Blag 1_06  tggaactgaaacagtagggaatttgtcttgaaattgcctacacacttctctgcatgactc
                                             *   .

Blag 1_01  aatgcatatgcagaaatacgttgttaaa----ataagggaaaagggcgtcccgtatgttg
Blag 1_02  aat---------------------ctaaccctcaaaagaaacaaacctccaccggaacct
Blag 1_03  ggaaaacatagtggtcctaacctcttaacccgccattacggaaaagttttggcgaacccc
Blag 1_04  cat------------------------------------tggctacttttatttactttt
Blag 1_05  agcttatatttaactctcaagttctgaa----ataatttaggaatggtgcctctgacacc
Blag 1_06  tgtttta----------------cataagtgtcatacatgaacactctttgttcaagcgt
            .                                     ..  .     .    .
```

Blag 2

5’

```
Blag 2_01  atccaatatctggttcatagaattaattaaaacaaaagatatagaaacaataaaccaaca
Blag 2_02  aatccatatcttttgtgtaaggaaaaatggagggcaaaatttaagtgttacgg------g
Blag 2_03  aatgcccagct---------ggaaaatgaattcattgagtatatagggttcaaaccaact
           * .   .* **         ..  **  ..   .  ...* ** . .   ...

Blag 2_01  tattacaaggaaatacggtaatagaatgttagaatgaaaacatttcaattagtttgatac
Blag 2_02  tgtgacaaaatttttctacaggcattgcgcattcatgcagttaatttagtaactctgttag
Blag 2_03  atttatcaag-------------ggacatggacataaccttaaaataatgtcaaggttac
             * *. *..               ....    **.    .*   .*.*      * **

Blag 2_01  ttacaaaattgtacttggagataaatattttcataatccactgttggtagttggtactcg
Blag 2_02  ttactacattctgcaataatgggacatttttt----------ttctggtagttggtactcg
Blag 2_03  ttatcgtaaac-aaagaattaactcacttttt----------gcttcataatcttcatttc
           ***. . *    .    .  .      ****            .* .**.*.  .*.*.
```

```
Blag 2_01  aggtaagcggacgtgtttcggatcgctcccgagttgaggttatggtgccgggtggagtca
Blag 2_02  aggtaagcggacgtgtttcggatcgctcccgagttgaggttatggtgccgggtggagtca
Blag 2_03  ctgtttat-------ttccattgtactcccacactgacacatcgcatataagtaaattta
             **  ..       **.*.   ..*****. ..*** ..  .*    ...**..* *.*

Blag 2_01  acaatgtcaacatgagcctttcaaattcaaattgtatccgttgttaagtgaatgttgcta
Blag 2_02  acaatgtcaacatgagcctttcaaattcaaattgtatccgttgttaagtgaatgttgcta
Blag 2_03  --------tattttaataatgtaaaacaacatttatttcatataaacatagccataatc
                    *. * *..  * .*** . * *** ***..  *.* **   *..  *. *

Blag 2_01  atttaaccaacggttttaaatatgtacaatg----tgaaaattacgttcataagagctgt
Blag 2_02  atttaaccaacggttttaaatatgtacaatg----tgaaaattacgttcataagagctgt
Blag 2_03  aataaa--aacaattacgaaaacagaaaatgacattgatcagggtacccaacagagcaaa
           * * **  ***..** ..** *.. * ****    ***  *  .....**  ***** .

Blag 2_01  gctaagcttctaaaaatgtgaaattcatcgataagaatttaattcagtgctgtgaaaata
Blag 2_02  gctaagcttctaaaaatgtgaaattcatcgataagaatttaattcagtgctgtgaaaata
Blag 2_03  ttagcctacatctaaatgcgaaatcgcattatcaattacattggtagaactgcaatagta
            . .  . . *  *****.*****.   . ** *.   .     .** .***..* *.**

Blag 2_01  ccgttggtaatt--cgatcagtgatggtgagtgctgtgtaaaagatgacgtcgaatgcga
Blag 2_02  ccgttggtaatt--cgatcagtgatggtgagtgctgtgtaaaagatgacgtcgaatgcga
Blag 2_03  acatgtgtcatctatgatcagtgatggtgagtgctgtgtaaaagatgacgtcgaatgcga
            *.*  ** **.  .*********************************************

Blag 2_01  ccccgctgttacttaaacatcattatacttgttgatatcttcaaactactacaatttatg
Blag 2_02  ccccgctgttacttaaacatcattatacttgttgatatcttcaaactactacaatttatg
Blag 2_03  ccccgctgttacttaaacatcattatacttgttgatatcttcaaactactacaatttatg
           ************************************************************
```

3’

```
Blag 2_01  tataatgcatacgttatactttgtccatagtggcaactcgcaactgcgagaagttgttga
Blag 2_02  tataatgcatacgttatactttgtccatagtggcaactcgcaactgcgagaagttgttga
Blag 2_03  tataatgcatacgttatactttgtccatagtggcaactcgcaactgcgagaagttgttga
           ************************************************************

Blag 2_01  tttcgttcaataaatatatataatatatatttatccataattcaattgaatttcaaactc
Blag 2_02  tttcgttcaataaatatatagctttttttaggacaaggttactttttcgaaattaagattt
Blag 2_03  tttcgttcaataaatatatacaata---gtaaattcatatcaagaggaagaatttaaata
           ********************   *    .       .*  .      .*.  *  .* *

Blag 2_01  gtaaagatctaaatcaagtctagaattatgaataatgatccacttaagaatcgagcaata
Blag 2_02  gtttttatccaagggcaca-------------------------------------gaga
Blag 2_03  ataatgtttataagatacgagaaaattgtatttatttattcattctagga------agtg
           .*     *.  *.   *                                       .. .

Blag 2_01  ataaatacatcgcaaaagaactgaataagctaagggataattattattagactgaa-ttt
Blag 2_02  attattaaagcatgtttcaaaaatttgcgagcatgatattttaaatattttgttattat
Blag 2_03  aaaattacactagcctataaccatttccgcataaacataattaaaacatacaaaga-aat
           *  * ** * ..      **  .  *       .  *** **   *.        *   *
```

Blag 3

5’

```
Blag 3_01  gctcatatccaggacttattgacaatacattctccattttgttttgtagtattttctcgg
Blag 3_02  cttcataacatggaagtaatatatgt-tagaataggcattattaaatggagtagtttcga
Blag 3_03  agtgacgtgagaagcaaccttcagatagacacacaataacataaaagacgttg-------
Blag 3_04  aataatatcgaaccactcttctgggtgttttacttgttttgtcaggtactttatttgaga
             * *..    .       *    .*         ..  ..*   . .   *

Blag 3_01  tattactggaaacagaatttaaactgaaagttgtatctttaaaaacttaagtgcatcatt
Blag 3_02  taaggcgtactatagaatttaaat-----------tcttatctaaagtaaatattttatg
Blag 3_03  --------tatacaatactccaatggcggacattgtcactgcttgtacaactatcactcc
Blag 3_04  a-------cattctatcattttatgtgtaagttcgtttcattatgtagtaagctccatcc
                       . .   *.  *.           *. .     .    *    .    .

Blag 3_01  taaggttagacgtattt-----gattgagaaggctgtggaattataagtttttgatattg
Blag 3_02  tcgaaagaatatttgaa-----tattgagaaggctgtggaattataagtttttgatattg
```

```
Blag 3_03  agaggtgtttccaaaca-----cattgagaaggctgtggaattataagtttttgatattg
Blag 3_04  ggatatatatatgacaaattggatttgagaaggctgtggaattataagtttttgatattg
             . .                   ************************************

Blag 3_01  ctatactgggacctattaatctttaattatcaatatttactacatcttttggaaattatg
Blag 3_02  ttattctgggacctattaatctttaattatcaatatctactacatcttttggaaattatg
Blag 3_03  ctatactgggacctattaatctttaattatcaatatctactacatcttttggaaataatg
Blag 3_04  ctatactgggacctattaatctttaattatcaatatctactacatcttttggaaattatg
           .*** *******************************.******************* ***
```

3’

```
Blag 3_01  ttttaaattattttcaacatttttaaattcattttattatcaaaattcatcttgttttct
Blag 3_02  ttttaaattattttcaacatttttaaattcattttattatcaaaattcatcttgttttct
Blag 3_03  tttttaattattttcaacatttttaaattcattttattatcaaaattcatcttgttttct
Blag 3_04  ttttaaattattttcaacatttttaaattcattttattatcaaaattcatcttgttttct
           **** *******************************************************

Blag 3_01  ggatccaagaggtctcccgataattagggcagatgttctttttgaacttagtagtagtag
Blag 3_02  ggatccaagaggtctcccgataattcgggcagatgttcttttcgaacttagtagtagcta
Blag 3_03  ggatccaagaggtctcccgataattcgggcagatgttctttttgaacttagtagtgtata
Blag 3_04  ggatccaagaggtctcccgataattcgggcagatgttctttttgaacttagtagtaagaa
           ************************* ****************.************.   .

Blag 3_01  ct-----------ttaaatagtccaagaaaatttg-------------------------
Blag 3_02  tgggcactgtagggttagtaggggtgatacgtcaggtatgaggacgtggagagcagctcg
Blag 3_03  ta-------------gaatatcgcctggacatctgctttggaagcacttttgtcaattat
Blag 3_04  ac-------------aaataacatttatagtggtgatgttgataccgtctgcttgcttta
                           *.**      . *     *

Blag 3_01  ---caggcagggaacaag--attagcttccaattcgctattacacaaattt---------
Blag 3_02  cagaaccatgtcaacgagtggtggactactaaagcatt----------------------
Blag 3_03  tgttaaattattggtaaattttgtttcggtaagccatt----------------------
Blag 3_04  ttacaaattaaaagcaagcagacggtatcaacatcatcactatactaatcttgctttggt
               *    .  ....*.       .    *   *...

Blag 3_01  -------ttgggctgtaagaatggtgtactgtcggcccaaaaaaaaaaaaagg--------
Blag 3_02  --------------cgggcaatcattagacattctaatataaattatcaagaaaatttt
Blag 3_03  --------------------atagtaaagaattaaaaagtaaagtaaaatgaagattaca
Blag 3_04  atcaaaatgaagataaaataacatttaattattgctaaagaaaatgacaaggatttccct
                               *.  *  .  .*.     . ***  .  * *.
```

Blag 4

5’

```
Blag 4_01  tc-aattatgctgaatgtttactacttaaatagggcataa-cataactgctac-tccagt
Blag 4_02  ta-tagcaattcgtataataatg-------taggccatgatcgtacggataat-ttcatt
Blag 4_03  aagtcataaaatgagcaatcaat-------taaaatagtaacttacaaaacatattaata
Blag 4_04  tt-taaaggtttatttatatatt-------ta---------tttatttaccatattcatt
                  .   ..  ..   *         **         . **   .  *. *. *

Blag 4_01  tattggatatcgttgagtaatattttcataatcatactcttttaaagataca---cgagc
Blag 4_02  cagagcatat--------------------------tttaagttaagaatgacttatagg
Blag 4_03  tat---tttt--------------------------tttacaaaattctgta---tattt
Blag 4_04  tatttattat--------------------------ttcatccaaaaaaaca---aaaaa
           .*     * *                          .*.     *      *

Blag 4_01  atactggaggaggcagtgaagttgaattggcttctgttgatgggctctgtacatttcta
Blag 4_02  aggatacaggaggcagtgaagttgaattggcttctgttgatgggctctgtacatttcta
Blag 4_03  ttcagtcaggaggcagtgaagttgaattggcttctgttgatgggctctgtacatttcta
Blag 4_04  aaacaacaggaggcagtgaagttgaattggcttctgttgatgggctctgtacatttcta
                  *****************************************************

Blag 4_01  tca-tttttcatgttttcatgcgacttgctgcttcggaagtgttttcaattcaagtttac
Blag 4_02  tca-tttttcatgttttcatgcgacttgctgcttcggaagtgttttcaattcaagtttac
Blag 4_03  tca-tttttcatgttttcatgcgacttgctgcttcggaagtgttttcaattcaagtttac
Blag 4_04  tcatttttttcatgttttcatgcgacttgctgcttcggaagtgttttcaattcaagtttac
           *** ********************************************************
```

3’

```
Blag 4_01  tattgttttatatatactgtatttatgttttgtatactgaggcaaaagtgtctacctctg
Blag 4_02  tattgttttatatatactgtatttatgttttgtatactgaggcaaaagtgtctacctctg
```

```
Blag 4_03  tattgttttatatatactgtatttatgttttgtatactgaggcaaaagtgtctacctctg
Blag 4_04  tattgttttatatatactgtatttatgttttgtatactgaggcaaaagtgtctacctctg
           ************************************************************

Blag 4_01  ttgggaggcagtattgaataaataaata--------------------------ttttta
Blag 4_02  ttgggaggcagtattgaataataataat----------tattcaaataatatttatatt
Blag 4_03  ttgggaggcagtattgaataaataaataaa-------taatgttgatactggatgattct
Blag 4_04  ttgggaggcagtattgaataaataaaataaaataggtcgatcaagacaatactgtagaca
           *********************  * *                                . 

Blag 4_01  aatatgcctttatagcttacaataatttaaa-----------------------------
Blag 4_02  agtctaatcgtcattccttaattattttaaa-tagctattt-----atcacatttcaaag
Blag 4_03  aatttttattattttttttatttatttatt-tttttattt-----attttttttttttc
Blag 4_04  aaaacggaacagcatattgcgttgatttgttatgatcatgttgccagttaaaattatggg
           *.  .           .*  . *  ***.

Blag 4_01  ------tcctctaccagtcatggaggtgcatatacagaattaactgcatcctagattctc
Blag 4_02  ttgataccaatcttatgtactatttgtgtttctat---agcaatt--------cgtataa
Blag 4_03  catgtactatatgttagtgttcatagtgtcagtgt---aaaaa---------------aa
Blag 4_04  tgggtatcgattacgcgcgataaaggtaaaaattc---atcaactacgcgcggtggtcag
                 ..   . .  *.  *    **.    * .   *  **
```

Blag 5

5’

```
Blag 5_01  aaaataagacctatgtttatatatgaatttttaatcacattgataccagcaaccagatc
Blag 5_02  aacgaaatttatagtttaaag----------acaaaaaaatccctcttctaaaggcatct
Blag 5_03  aattcaagttttatcttgatg----------ataattgcattttgtttgttaactttgta
           **   **  . **  ** * .           .**  . **.    ..   **     .

Blag 5_01  ctgcaatagtgtccaaacttcgtgaatcaccctgtacagatacgagaagctgcttataac
Blag 5_02  aatttaa----ttgtattttgtccctcat-------------------------aaag
Blag 5_03  catgtaagtttttttaattttataccgtatttaaataataagccaatgacaaatgaaac
                *     *.  * .**..*    .*.                           **

Blag 5_01  ----tgaaattacgagtataaaaaaaaaaaaaaaaaaaaaaaga--taagaaggccatgac
Blag 5_02  aatgtgaaaatgtaagcaattatcaataataataagttaataagttaagaaggccatgac
Blag 5_03  gaggtggaatttcaaaactccagcaatcgtaataacgtagtga--taagaaggccatgac
               **.** * ..*.     *  **  . ** **   *. .*  ***************

Blag 5_01  ttgagtttgctactattctatgtgcatcgcaactttttttacaattttacctgcttatcta
Blag 5_02  ttgagtttgctactattctatgtgcatcgtaactttttttacaattttacctgcttatcta
Blag 5_03  ttgagtttgctactattctatgtgcatcgcaactttttttacaattttacctgcttatcta
           *****************************.******************************
```

3’

```
Blag 5_01  tttttacatattttaaatttataaatgtttggtacgctttgtcttatttggcaccctta
Blag 5_02  tttttacatattttaaatttataaatatttggtacgctttgtcttatttggcaccctta
Blag 5_03  tttttacatattttaaatttataaatgtttggtacgctttgtcttatttggcaccctta
           ***************************.********************************

Blag 5_01  aatttaaggaagctattaaatttaatttacctt----tttgtcagattttaacaatagac
Blag 5_02  aatttaaggaagctttt-------------------------------------------
Blag 5_03  aatttaaggaagcttttcattgtattctataataaaatatttaaaatagtaacattttaa
           ************** **

Blag 5_01  ttaata-aaatgttaatttaagattagctatagtttcgaccacttccaacagaat-tgca
Blag 5_02  -------aaatttaaaaaataccgtaactggtttctaacaaataccttac----------
Blag 5_03  aaaatagaaatcgcaaaaacaggtcaactggattattgtttgttctttctacagtatgta
                  ****   **    *   .*.**.   * * .   .. ...  .

Blag 5_01  acgttgtgcacgcatgtatacgagtgttctggtacagtggtggtagcaagaatcgaaaat
Blag 5_02  -----gttttgtgataattatactttattactataaacagtttgtcggacaaagccaat
Blag 5_03  agttagtctaataaaatttacatttttcttctattggtactttatagttgtccaaata
                ** .    * .  **..  * * .*  **. .  .   *  ...  .     *
```

Blag 6

5’

```
Blag 6_01  tgtgaataaacaatttacca----taaaatcaactttaacatagtaacgtatacaaactt
Blag 6_02  aatatttgctctacttttcatctatttaatgaacattattattg-aaagactgtgaaatt
Blag 6_03  aaagagtgaataaaattttacaactataatagttatttatgcaacaagggataaaataaa
            . .  *.  . *  * ..*    *  *** . . **  ... . ** *  *. .*

Blag 6_01  cagggaatcttgttagctgttctaaaaattggctataaaacg------------tagtac
Blag 6_02  --------tttattagtttgagcatattcaagtcatagccta------------cttgat
Blag 6_03  ta------ttttacggttgagtaaaatctaacatatgctttggatgaactgatcctcgat
                   .**  ..*.*     * *  . .  .**.   ..            .   *.


Blag 6_01  actatgtacaattattttttaagtattacgttcacaagagctgtgctaagcttctaaaaa
Blag 6_02  atttcatgtcatgacaatgtgaaaattacgttcacaagagctgtgctaagcttctaaaaa
Blag 6_03  catggccaacattagaatgtgaaaattacgttcacaagagctgtgctaagcttcaaaaaa
             *   ..  ** *   * *.*. ****************************** *****

Blag 6_01  tgtgaaattcatcgataagaatttaattcagtgctgtgaaaataccgttggtaattcgat
Blag 6_02  tgtgaaattcatcgataagaatttaattcagtgctgtgaaaataccgttggtaattcgat
Blag 6_03  tgtgaaattcatcgataagaatttaattcagtgctgtgaaaataccgttggtaattcgat
           ************************************************************
```

3’

```
Blag 6_01  agcaaaagaaaattaatatccctaatctaagtacctattataaaattattgtatttatta
Blag 6_02  agcaaaagaaaattaatatccctaatctaagtacctattataaaattattgtatttatta
Blag 6_02  agcaaaagaaaattaatatccctaatctaagtacctattataaaattattgtattcatta
           *******************************************************.****

Blag 6_01  ttgattggcaggctgtaccttttggtaacccttaataaagggcagctatcctattttagg
Blag 6_02  ttgattggcaggctgtaccttttggtaacccttaataaagggcagctatcctatttttgg
Blag 6_03  ttgattggcaggctgtaccttttggtaacccttaataaagggcagctatcctatttttgg
           ********************************************************* **

Blag 1_01  atttctctctcat---tgatttcacatatcaatgtctcgggtattttaaagccaattgt
Blag 1_02  atttctctctcccataagaatagctgtagaaacacaagtagtagcctacaaatactggt
Blag 1_03  atttctctctctaaatattcttccctggcaacaacaaaaatcaataagcattgttggca
           ************         *   .  .  *  ..    . .* .  . *

Blag 6_01  agaagttttacaagttgaggaccaaacaagacacctgaa---------------------
Blag 6_02  attaattttgaaatttgataaatagctatttcaaataagt----------------aagc
Blag 6_03  aaaaatctcaataaattacaattcgctttgagcaatgatttttttattttatcgtcttatc
           *  *.*.*..  *  * * .* . . .        *.*

Blag 6_01  aaaaacataaaatattgagagtgaaaataataataatgaacaaagaatattaatatgtta
Blag 6_02  aaagacaattgatataaaatgtg-gtttgatgaatgtatattgagcgtatttacttgct-
Blag 6_03  aaaggaatccagtg------------------------actgaacccccttacagtcca
           ***.. *   ..*.                         *. .*.  . .* *.   ..

Blag 6_01  at-ttaa
Blag 6_02  -------
Blag 6_03  ctatctt
            * *
```

Supplementary Figure 3

Alignment of the nucleotide sequences corresponding to the 5'- and 3'-ends of all identified copies of BLAG 1 – BLAG 6 retrotransposons, together with the corresponding sequences of the environments of the integration sites. Sequences of retrotransposon fragments are highlighted in gray; no highlighting indicates unique environment sequences; blue, green, and yellow indicate similar sequences surrounding the various copies of the BLAG retrotransposons. The sequences of primers used to amplify the corresponding DNA fragments are underlined.
