# Supplementary material for: R2 and Non-Site-Specific R2-Like Retrotransposons of the German Cockroach, Blattella germanica
Source: Genes (Basel). 2020 Oct 15;11(10):1202. doi: 10.3390/genes11101202 (PMC7650587; doi:10.3390/genes11101202)
Supplement: Supplementary file 1 [file genes-11-01202-s001.zip › Supplements/Supplementary Figure 4.pdf]

A

>Blag 2

GATCAGTGATGGTGAGTGCTGTGTAAAAGATGACGTCGAATGCGACCCCGCTGTTACTTAAACATCATTATACTTGTTGATATCTTCAAACTACTAC
AATTTATGTTGGATTTTCTCCGTGAAGACTGACTTCTCTCCTTAGACATTGTTTTACTCATGATATCAGATTCTGGAGGACCTTCGTTACCATCTAA
TGACGGTAAGACTATTGCTTTAGTCTCTTGCAAGATATGTGGAAAAAGTTTTACTAGACATGGAATTAATATCCATATTGCTAGAAGCCATAGACAT
TCCCAAGTTTTGATAGACATTGATAAGCCTACTGATTCAATTTTATCAGAGACTGTAAACTTCTCACCTTCTGACCCTATTGAATGCCAGGATAATA
TCCATCTTAATACTAATTCATCTAACAATGAGAAAGTCATTCGTAACAATTCCAATGCATCAAGTATACATCTAATAGATGGTTCATTAATTGGTAC
TCCCTCTGACTCTTCTCCAGCTGTTTCGCAAGCTCAATGTCCCCATTGTGGAGGTTCATTTAACGGATCTAAGGGTGTCAATATTCATATTAGTCGA
TCGCATCCCCACATTCATAGATCGACATTAGTTACAAATTTAACTAAAAATGCGTCCCAGCCGCTCCTTGATGAGCAAGCTGATAGGGGTAGTTCTA
GTAATTCAGAATCCAGTCAACCTGACTCAGGTTTTAACATAGCCCTGAATGAGTGGAAAACAAAATTTGTTCGCACTGCAACTGCAAATGACTCTGA
TTTCGATAACATGGTCCAGGATTTTTCACACTTCTTAAGTACTAGCATCAATCTTTTACCCGGACCTCGACATCCCGCAACCAAGTACTATGAAGCC
CGCAGGAAACGACGTCTCGCAACAGCAGAAAAGAACTACAATAAGTCCT

>Blag 6

ATTACGTTCACAAGAGCTGTGCTAAGCTTCAAAAAATGTGAAATTCATCGATAAGAATTTAATTCAGTGCTGTGAAAATACCGTTGGTAATTCGATC
AGTGAAGGTGAGTGCTGTGTAAAAGATGACGTCGCGAATGCGACCCCGCTGTTACTTAAACATCATTATACTTGTTGATATCTTCAAACTACTACAA
TTTATATTGGATTTTCTCCGTGAAGACTGACTTCTCTTCTTAGACATTGTGTTACTCATGATATCAGATTCTGGAGGACCTTCGTTACCATCTAATG
ACGGTAAGACTATTGCTTTAGTCTCTTGCAAGATATGTGGAAAAAGTTTTACTAGACATGGAATTAATATCCATATTGCTAGAAGCCATAGACATTC
CCAAGATTTGATAGACATTGATAAGCCTACTGATTCAATTTTATCAGAGACTGTAAACTTCTCACCTTCTGACCCTATTGAATGCCAGGATAATATC
CATCTTAATACTAATTCATCTAACAATGAGAAAGTCATTCGTGCAGTCAATAATGATGAGAAAGATAAAAGTGATAACAATATTATTAAAAATACAT
CTTCAGATATCTGGTGTCCTTACTGTAGTAAAGTATTCAAAAATTTAAATGGCCATATAGCACGATCACATCCCCATATTTATAGAAAAAATATTAC
CGAACGTTATGCCAATGCTTCTCCTTGTCTGCAAAAAACAGAAATTAATAATAATAGCGGTCAACTTAAAATAAATGAAAATAAGTTAGAGCCTTGG
GAAAAAACATTCTCTGAAATTCTTAATAGCGAAAAAGATGAAGTGAAACTAGACCAGAAGTTAGATGAATTTACCTTATTTCTATGCAATGCCACCT
CTCTGTGCAAAGGTCCGGTACATCCTGCTACAAAGTATTATAAAGCTAGAACTACATGGAGACCAAATCATGAGAAGTCTAATTTTAAAACATCCA

B

```
CLUSTAL format alignment by MAFFT (v7.461)


Blag_2          ------------------------------------------------------------
Blag_6          attacgttcacaagagctgtgctaagcttcaaaaaatgtgaaattcatcgataagaattt


Blag_2          ---------------------------------gatcagtgatggtgagtgctgtgtaaa
Blag_6          aattcagtgctgtgaaaataccgttggtaattcgatcagtgaaggtgagtgctgtgtaaa
                                                 ********* *****************

Blag_2          agatgacgt--cgaatgcgaccccgctgttacttaaacatcattatacttgttgatatct
Blag_6          agatgacgtcgcgaatgcgaccccgctgttacttaaacatcattatacttgttgatatct
                *********  *************************************************

Blag_2          tcaaactactacaatttatgttggattttctccgtgaagactgacttctctccttagaca
Blag_6          tcaaactactacaatttatattggattttctccgtgaagactgacttctcttcttagaca
                *******************.*******************************.********

Blag_2          ttgttttactcatgatatcagattctggaggaccttcgttaccatctaatgacggtaaga
Blag_6          ttgtgttactcatgatatcagattctggaggaccttcgttaccatctaatgacggtaaga
                **** *******************************************************

Blag_2          ctattgctttagtctcttgcaagatatgtggaaaaagttttactagacatggaattaata
Blag_6          ctattgctttagtctcttgcaagatatgtggaaaaagttttactagacatggaattaata
                ************************************************************

Blag_2          tccatattgctagaagccatagacattcccaagttttgatagacattgataagcctactg
Blag_6          tccatattgctagaagccatagacattcccaagatttgatagacattgataagcctactg
                ********************************* **************************

Blag_2          attcaattttatcagagactgtaaacttctcaccttctgaccctattgaatgccaggata
Blag_6          attcaattttatcagagactgtaaacttctcaccttctgaccctattgaatgccaggata
                ************************************************************
```

```
Blag_2          atatccatcttaatactaattcatctaacaatgagaaagtcattcgtaacaattccaatg
Blag_6          atatccatcttaatactaattcatctaacaatgagaaagtcattcg------------tg
                **********************************************            **

Blag_2          catcaagtatacatctaatagatggttcattaattggtactccctctgactcttctccag
Blag_6          cagtcaataatgatgagaaagataaaagtgataacaatattattaaaaatacatcttcag
                ** . *.**   **  .* ****..       * ...**.* ..   .*. * ***.***

Blag_2          ctgtttcgcaagctcaatgtccccattgtggaggttcatttaacggatctaagggtgtca
Blag_6          atatct---------ggtgtccttactgtag---------taaagtattcaaaaatttaa
                 *.*.*         ..*****..*.***.*         *** * **..**...* * *

Blag_2          atattcatattagtcgatcgcatccccacattcatagatcgacattagttacaaatttaa
Blag_6          atggccatatagcacgatcacatccccatatttataga------------aaaaatatta
                **. .***** .  *****.********.***.*****            * **** * *

Blag_2          ctaaaaatgcgtcccagccgctccttgatgagcaagctgataggggtagttctagtaatt
Blag_6          ccgaacgttatgccaatgcttctccttgtctgcaaaaaacagaaattaataataatag--
                *..** .*    ** *  * ...*.* .*  ****.  .  .... **.*  **.**.

Blag_2          cagaatccagtcaacctgactcaggttttaacatagccctgaatgagtggaaaacaaaat
Blag_6          -------cggtcaacttaaaataaatgaaaataagttagagccttgggaaaaaacattct
                       *.******.*.*  .*..*   **.* . .   *  * .* ..******   *

Blag_2          ttg---ttcgcactgcaactgcaaatgactctgatttcgataacatggtccaggatttt t
Blag_6          ctgaaattcttaatagcgaaaaagatgaagtgaaactagaccagaagttagatgaattta
                .**   *** .* *.  .  . *.****  . .* .* **. * * * *  * ** ***

Blag_2          cacacttcttaagtactagcatcaatcttttacccggacctcgacatcccgcaaccaagt
Blag_6          ccttatttctatgcaatgccacctctctgtgcaaaggtccggtacatcctgctacaaagt
                * .  **..** *.* *. **.*  *** *     ** **   ******.** ** ****

Blag_2          actatgaagcccgcaggaaacgacgtctcgcaacagcagaaaagaactacaataagtcct
Blag_6          attataaagctagaactacatggagaccaaatcatgagaagtctaattttaaaacatcca
                *.***.****. * *  * *.*. * *. .     * ..*.   **.* .** * .***
```

Supplementary Figure 4

Comparison of BLAG 2 and BLAG 6 retrotransposon nucleotide sequences.
A – Nucleotide sequences of 5'-end fragments of the BLAG 2 and BLAG 6 retrotransposons. Sequences corresponding to ORFs are highlighted in yellow, and 5'-untranslated regions are highlighted in gray. **B –** Alignment of the corresponding DNA fragments.
The primers used for PCR amplification are underlined.
