# Supplementary material for: R2 and Non-Site-Specific R2-Like Retrotransposons of the German Cockroach, Blattella germanica
Source: Genes (Basel). 2020 Oct 15;11(10):1202. doi: 10.3390/genes11101202 (PMC7650587; doi:10.3390/genes11101202)
Supplement: Supplementary file 1 [file genes-11-01202-s001.zip › Supplements/Supplementary Figure 5.pdf]

A

>**BLAG 1**
PPEHRQSILTRQSRTKQSTSSEPQLRNPTDNDQSIVDRQVDVTEINDCSVLNDDLATYKNEMQK
WKLEFSKNNTNDSFNTKVQEFLNFLARAVHLLPGPKHPAVRFYEARRQRRQFSNQVRYKQSSNP
ERGTKRARDKRRELYEYQKTQFLFYNQRRKAVRSALNDRHTACKVDINKIFNYFSSRLSTENSC
ERPAYSSNVTEPERAVLDSTEDSVITKQEVAVAVSKIAADTAPGPDHVLVRALKDATCYEIIAK
IATTMLQHSFVPECLQKARTVL

>**BLAG 2**
PHIHRSTLVTNLTKNASQPLLDEQADRGSSSNSESSQPDSGFNIALNEWKTKFVRTATANDSDF
DNMVQDFSHFLSTSINLLPGPRHPATKYYEARRKRRLATAEKNYNKSSNPIRASKKAREKRKCK
YDYQLTQYQFYNQRRKAVRRVLDSTAPSCKIDLTKVHENFAASFSIENPHIRSNYPSKLTDVER
IELDETYDPTISKEDIAIAAHKIAVDTAPGPDHVLVRVIRNEIVYEILAIIATRMLSSGIVPRC
FKTARTVL

>**BLAG 3**
GEAYRKKLSHKYECPLENTKDTDTDSPSSNNPITVADVSSPATQQILQFEKALEECILSSDLNR
FEEITVEITAYLSKAIASLPGPKHPATHYYNARKLRQTIHGTSYSNTKNPARLSKRKRKDNKQK
YQYEVAQFHYFNQRRKVVRQIMKNHNNVQCQISIPIIFQHFNTLFNNENPFTPIESIHPVSTEE
DIEITEDEIKAAMAKIKIDTSAGIDHVLVRTIKELKTSKLLCLLTNAMLKFKICPGILKTARTV
LL

>**BLAG 4**
PEQHRKSIVMRNTSSVSPVVQTASKDDNVSNSSANTNSDVGHLHEIRSEVMSFREKFSSLLLSD
PCPSKFNEIYNDFTDTLVTINSKLPGPKNPSVRYYELRQRNKNIFAANDRKYKDSSNPQKKSKR
DRAKRREKFQYELLQYQFYNERKKCVNSLLNDNKRNEPCNIPIGVLKEAMEEKWGTLNTSMLDH
YNNSMSPHSQQNDLDQLFTIPVEVIQQSLNKMRTDSAPGPDRILVRTLKLTECSPVIAVILKIM
LQWGMVPDRMRGARTIL

>**BLAG 5**
PDLHRLKLVERQTTSSYTIWNSGESSSTCKTSINDTPSKSHPVSVNDPLSNYKQQLLTWTTKFD
CDLTDIDFDTLTNNFLQFLSEAIYYLPGPKHPARKYYALRKKNKNFNCQQSYKNSSNPDRATKR
DRAKRKQKYQYEQTQFDYYNQRRKAVRRVLTSDKPSCQIAVDKVYAHFNSYFSRPNNCIRPDYI
SHVTEVERVELNDSFPTHITKEEIKFAISRIAVDTAPGPDHVLMRVLKDDAATGVLSLLATRML
RTGHVPPCLLIARSIL

>**BLAG 6**
PHIYRKNITERYANASPCLQKTEINNNSGQLKINENKLEPWEKTFSEILNSEKDEVKLDQKLDE
FTLFLCNATSLCKGPVHPATKYYKARTTWRPNHEKSNFKTSNNPQRHDRRLKEKRQNQYRYDLM
QYYYYYRRKKAVQKIMHLSDYKRCPFSATDVYDYFKETVGTENNNVLHDYSISSPLCNSIDEET
IDYDCQYCTVTEKDIDQAMKGINLDTAPGPDRVLFKVVKELKCMKIISIIANIMLSWNYVPNSF
QSGRTIL

B

MAFFT-L-INS-i Result

```
CLUSTAL format alignment by MAFFT (v7.468)


Blag_1          PPEHRQSILTRQSRTKQSTSSEPQLRNPTDNDQSIVDRQVDVTEINDCSVLNDDLATYKN
Blag_2          PHIHRSTLVTNLTK-----NASQPLLDEQADRGSSSNSESSQPDSG-----------FNI
Blag_5          PDLHRLKLVERQT------TSSYTIWNSGESSSTCKTSINDTPSKSHPVSVNDPLSNYKQ
Blag_3          GEAYRKKLSHKYE------CPLENTKDTDTDSPSSNNPIT----------VADVSSPATQ
Blag_6          PHIYRKNITERYA------NASPCLQKTEINNNSGQLKIN-----------------EN
Blag_4          PEQHRKSIVMRNTS-----SVSPVVQTASKDDNVSNSSANTNSDVGH-------LHEIRS
                   :* .:  .                   .

Blag_1          EMQKWKLEFSKNNT----NDSFNTKVQEFLNFLARAVHLLPGPKHPAVRFYEARRQRRQF
Blag_2          ALNEWKTKFVRTAT--ANDSDFDNMVQDFSHFLSTSINLLPGPRHPATKYYEARRKRRLA
Blag_5          QLLTWTTKFDCDLT----DIDFDTLTNNFLQFLSEAIYYLPGPKHPARKYYALRKKNKNF
Blag_3          QILQFEKALEECILS-SDLNRFEEITVEITAYLSKAIASLPGPKHPATHYYNARKLRQTI
Blag_6          KLEPWEKTFSEILNSEKDEVKLDQKLDEFTLFLCNATSLCKGPVHPATKYYKARTTWRPN
Blag_4          EVMSFREKFSSLLLSDPCPSKFNEIYNDFTDTLVTINSKLPGPKNPSVRYYELRQRNKNI
                 :  :   :            ::    ::   *        ** :*: ::*  *   :

Blag_1          --SNQVRYKQSSNPERGTKRARDKRRELYEYQKTQFLFYNQRRKAVRSAL-NDRHTA--C
Blag_2          --TAEKNYNKSSNPIRASKKAREKRKCKYDYQLTQYQFYNQRRKAVRRVL-DSTAPS--C
Blag_5          --NCQQSYKNSSNPDRATKRDRAKRKQKYQYEQTQFDYYNQRRKAVRRVL-TSDKPS--C
Blag_3          ---HGTSYSNTKNPARLSKRKRKDNKQKYQYEVAQFHYFNQRRKVVRQIMKNHNNVQ--C
Blag_6          --HEKSNFKTSNNPQRHDRRLKEKRQNQYRYDLMQYYYYYRRKKAVQKIMHLSDYKR--C
Blag_4          FAANDRKYKDSSNPQKKSKRDRAKRREKFQYELLQYQFYNERKKCVNSLL-NDNKRNEPC
                       :. :.** :  :: : ..:  : *:  *: :: .*:* *.  :         *

Blag_1          KVDINKIFNYFSSRLSTENSCERPAYS-----SNVTEPERAVLDSTEDSVITKQEVAVAV
Blag_2          KIDLTKVHENFAASFSIENPHIRSNYP-----SKLTDVERIELDETYDPTISKEDIAIAA
Blag_5          QIAVDKVYAHFNSYFSRPNNCIRPDYI-----SHVTEVERVELNDSFPTHITKEEIKFAI
Blag_3          QISIPIIFQHFNTLFNNENPFT--------P-IESIHPVSTEED----IEITEDEIKAAM
Blag_6          PFSATDVYDYFKETVGTENNNVLHDYSISSPLCNSIDEETIDYDCQY-CTVTEKDIDQAM
Blag_4          NIPIGVLKEAMEEKWGTLNTSMLDHYN------NSMSPHSQQNDLDQLFTIPVEVIQQSL
                 .    :   :    .  *              .         :      :. . :  :

Blag_1          SKIAADTAPGPDHVLVRALKDATCYEIIAKIATTMLQHSFVPECLQKARTVL-
Blag_2          HKIAVDTAPGPDHVLVRVIRNEIVYEILAIIATRMLSSGIVPRCFKTARTVL-
Blag_5          SRIAVDTAPGPDHVLMRVLKDDAATGVLSLLATRMLRTGHVPPCLLIARSIL-
Blag_3          AKIKIDTSAGIDHVLVRTIKELKTSKLLCLLTNAMLKFKICPGILKTARTVLL
Blag_6          KGINLDTAPGPDRVLFKVVKELKCMKIISIIANIMLSWNYVPNSFQSGRTIL-
Blag_4          NKMRTDSAPGPDRILVRTLKLTECSPVIAVILKIMLQWGMVPDRMRGARTIL-
                  :  *::.* *::*.:.::      ::. : . **     *  :  .*::*
```

C

>**Consensus/1-292 Percentage Identity Consensus**
PEIHRKKIVERQTKTKQSTNASPCLQDTDADDNSSNNSINDNPDIGHCSSVNDDLSEYKNELLE
WEKKFSECLTSDKDDSDFDEITNEFTAFLSTAISLLPGPKHPATKYYEARKKRRNFFAANQKNY
KNSSNPQRASKRARAKRKEKYQYELTQFQFYNQRRKAVRRILNDNSDNPSCKIDITKVFEHFNE
KFSTENNCIRPDYSIFSPLSNSTDPERIELDCTYDCTITKEEIAQAMSKIAVDTAPGPDHVLVR
VLKDLKCSEIIAIIATIMLQWGIVPDCLKTARTILL

Supplementary Figure 5

Comparative analysis of the amino acid sequences of the BLAG 1 - BLAG 6 retrotransposons located between the zinc finger domains and the reverse transcriptase domain.
A – corresponding amino acid sequences, **B** – MAFFT-L-INS-i alignment, **C** – consensus sequence.
