# Supplementary material for: R2 and Non-Site-Specific R2-Like Retrotransposons of the German Cockroach, Blattella germanica
Source: Genes (Basel). 2020 Oct 15;11(10):1202. doi: 10.3390/genes11101202 (PMC7650587; doi:10.3390/genes11101202)
Supplement: Supplementary file 1 [file genes-11-01202-s001.zip › Supplements/Supplementary Figure 6.pdf]

A

```
>6AR1 (Group II Intron)
NQGAPGIDG-VSTDQLRDYIR---------AHWSTIHAQLLAG---TYRPA----PVRRVEIPKPG--------GGTRQLGIPTVVDRLI
QQAILQELTPIFDP-----DFSSSSFGFRPGRNAHDAVRQA---QGYIQ------------------EGYRYVVDMDLEKFFDRVNHDIL
MSRVARKVKDKRVLKLIRAYLQA-------------GVM---------------IEGVKVQTEEGTPQGGPLSPLLANILLDDLDKELEK
RG------------------LKFCRYADDCNIYVKSLRAGQRVKQSIQRFLEKTLKLKVNEE---------KSAVDRPW-----------
---------------------------------KRAFLGFSFTPERKARI---RLAPRSIQRL----KQRIRQLTNPN-ISMPERIHRVN
QYVMGWIGYFRL---------VETPSVLQT-------------IEGWIRRR----------LRLCQWL
>SLACS
RGAAPGLDG-WTRELLYPLTLD----PALKMEIAAVVKDIINADVSMEVGRRLQ-ATSLTVLRKPN--------GKYRPIGAESVWAKLA
SHIAISRVMKTAEKK----FSGIQ-FGVGG--------HIE---EAIAK----IRKDF---------ATKGSLAMLDGRNAYNAISRRAI
LEAVYGDSTWSPLWRLVSLLLGT------------TGEVGFYENG---------KLCHTWESTRGVRQGMVLGPLLFSIGTLATLRRLQQ
TFP----------------EAQFTAYLDDVTVAAP-PEELKNVCAATAEAM-EALGIVNNAD---------KTEVLELT---GDTGF---
------------------------GTAV---KRVREFLERTWP--DPMS----EEIREGVEKKAMETDRLFKAIVELP-LYNRTRWRILA
MSAMPRITFLLRNH-----DMQHTHRVASW-------------FDERTTQV----------MEHILGQ
>CRE1
RGTAPGLDG-WTRELLLPLAEDPALLHEITSVVSDIMQGKVA----EVVARRLR-SSAVTPIPKDE------AGTKIRPIVPESAWLKLA
SLVAMAEIPSSFKET----FKGWQ-YGVWG--------DVA---KAVAK----IRRDS---------EEHEYLVALDGVNAYNTMSRAHI
LQAVYAEQRLKPIWGVVKVALGG------------PGFLGVYRDG---------CLKGNLWSTKGIRQGMVLGPLLYATGMAAAIGPVRQ
RIP----------------GVPVTAYIDDITLAASGAEGA-RAAEAYADAL-ETVGVVTNAR---------KSMVVGPE---GTRVG---
-----------------IGG---VDLPV---VAEARILGAHFRARGTPEARTIEWLQAAVEKW----RPIHQKLRQDI-IPKNIAMMMTR
ISLGSKMTFLLQTH-----SPQELETAAKT-------------ADDEVEQT----------LQHLMGQ
>CRE2
RAAAPGLDG-WTRELLVPITED----KGLLTELTALVQDMLVG---NVHPSFAT-RIRACILHPFR---KEAGSAKVRPITPESALMKLA
AHIALDSVEKSFRST----FKGWQ-YGVWG------DSTEA---VKRIR----EAYAE---------ASSDTLVALDATNAYNRMSRRHI
LEAAYAQPELRFAFGVVNLSLGA------------AGELALYENG---------AKIHALKSTEGVRQGMVLSPLLFANAMSGIIRPLME
MHP----------------RVKVVAYLDDVTLIGP-HAAVQDFLAEAGPQL-SRVGFDINPA---------KSHHLAKL---EVPEA---
------------LSV--SGR---TIPIA---QGVVRILGAGFRGDTASVE---EWVWEKTKTH----DHYFEKLQSEW-LPRLARLQLLR
GSTVPRLNHLLRTH-----KPEELNRSTTW-------------FDERVMET----------ALNIANI
>GAU97528__Ramazzottius_
VDSAAGPDR-VLVRVIKQEAVME---VLAVIAAVLLEMNATKE---HPLPSVLQ-LALTVLIHKGG---DVTDGNNYRPISICSARRRVL
ERVVDRHLRRYGS------VSRFR-QGFTSAPGTHVNASIL---GSVSK----KAQAE---------QSTVVVVLLDIKQAFEGILHVHL
KKTLRASPLPTLVRNLILSLQGS-------------NRTQLQVGG---------QRSSAIPVKKGVFQGVPLSPMLYNMSTDYVTRNMTE
APIAEEYGFPL---CEGEQPVTFLGFADDSTVVGKSREEAIQLTEMAIGLF-KEIGLEVSPT---------KSKAIVVE---NG------
------VMSEVPLYL--SSG---AVIEATKKGEKVRYLGATVTDQLDF------DQGEVIKQL----TDQVDRLVHFAHLHADQKLNLLN
QWLLPSIIYPLQTA----PTNTILKVFLQT-------------VDKIVKST----------VREILQL
>BLAG 1
ADTAPGPDH-VLVRALKDATC--------YEIIAKIATTMLQH---SFVPECLQ-KARTVLIYKGG---DDKDLGNWRPITICSVVRRII
ERILDARLRALVN------LCENQ-RGFTNSPGTLINTSIL---ESILV----AAKSK---------KKDCTIVFLDISKAFDNVGHNHL
QNSLSALRIPFKLSSLILQLQRG-------------NKTQIETGR---------CRTKPIDIKRGIMQGSPLSPILYNISTDHILDELSN
QELQDEYGFSV---SPGLSNITALGFADDTLIAAKSMNSARILVELAMSRF-KEIGLDINTD---------KCRCISII---KG------
------CVRYNPLNL--TSG---QTIASITPDENIKYLGVSYNATIL-------DAANSIEEL----KNKLDRLASTPLLQPHQKYTVLC
SFICPTLIYKFQTT----PLKKLPAKFLSD-------------ADVLIKTT----------LKEVLQL
>BLAG 5
VDTAPGPDH-VLMRVLKDDAA--------TGVLSLLATRMLRT---GHVPPCLL-IARSILIHKSG---DVNDITNYRPITICSVVRRVI
ERTLDRRLKEYVS------FNRHQ-RGFTSSPGTLMNTSLL---RSILT----SAKTK---------KCNATLVFLDIRKAFDNVGHLHL
QKTLESLPIPSLLSNLILNLQEN-------------NHTRFEINH---------HKTNLVPLARGVMQGSPLSPTLYNLSTDHILNELSE
DGVAKAYGYQL---VPGLKPVTVLGFADDTVIVANSTTSALELTKMAIERF-RSIGLEINFS---------KSVAINIS---CGK-----
------LNPNYSLLV--AEN---ATIPCLGPNEFVRYLGVNFADACLF------DSQVTLNDL----KNKLDLLISSPLLNPDQKFCILN
SSICPSLIYTFQTT----PLNKIPNKFIEA-------------ADKIIKSG----------LKEILQL
>BLAG 2
VDTAPGPDH-VLVRVIRNEIV--------YEILAIIATRMLSS---GIVPRCFK-TARTVLIHKAA---DVNILSNWRPITICSVLRRVI
ERVLDKHLRTFVS------FNEHQ-RGFTNNSGTLINTSIL---RKVLK----TTKDN---------KEDVTVVFLDISKAFDNIGHKHL
DLTLSAEPIPAKLKDLIINLQIN-------------NTTNIQTSL---------GQTKAINLRRGVMQGSPLSPTLYNLATDFIFDELTE
LSLLQQYGKSI---SPQLSSLSIMGFADDTLLMGKNSETTLELLHIALQRF-QEIGLQVNPQ---------KCTGISIK---RG------
------KLVEEVLHI--NEK---FTIKTINNQEEVRYLGAPFQDNALF------DKDSTIKQL----HKKLELLASSPLLKSDQKFLVLN
TAICPTLIYRFQTT----DLQTIPLQFLHT-------------ADKLVRST----------LKDILQL
>BLAG 4
TDSAPGPDR-ILVRTLKLTEC--------SPVIAVILKIMLQW---GMVPDRMR-GARTILIYKGKG--ERCSVSSWRPISVCSVIRRLI
ERALDSVLRQYLT------LNNNQ-RGFVSQLGTSINTSLV---DGCLK----KAKLE---------QRNVSIVMLDVAQAFDSVGLDHL
EKCLDSQFLPTSLCTLIKTLTRG-------------NFTRIHNGK---------QISEPIELKRGVFQGSPLSPILFNLSIDFIIKELSE
KQITDKYGFKI---SNNLDHLSVAAFADDLVVIGQNMHAARELVMMTKYLL-QQIGLQLNIA---------KSVSINIE---HG------
------KLQDKELLL--YSG---YAIRGITKEEKIKYLGVSFNDEIIF------DRESFITSL----RNDLQILVSTPMLRPDQKLNIVN
QYIWPKFIYALQMV----PVHKLPLTFLED-------------VDKLIRSA----------VKEMLLL
>BLAG 3
IDTSAGIDH-VLVRTIKELKT--------SKLLCLLTNAMLKF---KICPGILK-TARTVLLFKGG---DETKLSNWRPISIFSVICRII
EKVINRRLKSYIE------LSPHQ-RGFINVPGTHINTSII---NGCLT----RAKKE---------KRNCCIAFLDISKAYDNVGHEHV
KRCLLRTNMPNNLRDLVLNLLQG-------------NKIQIESGF---------QKTKPIQVQKGVAQGSPLSPGLFNLALDDTLKELAD
PNVREVFGFSL---HPDLEKVSGLAFADNIALISKDEASLQTLINMTVFNL-DAIGLKVNPK---------KCMLINIK-----------
-----NGFLEQGQIL--VGG---QTLEFIKPGEIIKYLGVTFEDEIVL------DKSTLIKNF----ENDLNSLTVSNLLTPDQKLNIVN
QYIWSMLIYPLQCT----PLDKIPSSFLQD-------------IDKLLRSS----------IKEIIGL
>BLAG 6
LDTAPGPDR-VLFKVVKELKC--------MKIISIIANIMLSW---NYVPNSFQ-SGRTILIFKSG---DPNSLKNWRPITIFSILRRII
ERALERKIRPFVE------LSTCQ-RGFISTPGTFINSSLI---NSCLQ----KSKKE---------KGDCCITFLDVSKAFDSIGHKHI
EMSLNSTSIPSQLKSLVLSLVKN-------------NSVGIELDA--------TTRTKPINIEKGVAQGSPLSPLLFNLSIDFIYRNLND
FQNASQYGYRL---TEEVDNLIALSFADDIALISNNETSAVFMIERVNQCL-TEIGLNLNAS---------KSKIINIK---KG------
------KLMNTTVTT--LND---IKINSIQAEETIKYLGVTYNDEIILD-----KCSIITNLN----RSISKLITSSL-LKPDQKLNILN
QYIWPTLIYPLQCA----PLCKLQLGFLQD-------------IDKIVKNA----------VKEIVGL
>R2_PS
SKTAKGVDG-VGLEELKRVPR---------RTLFNILNGLW-----THVPESLY-KGRITLIPKKS---LPELAGDFRPICVLPVVVRLL
HRILAKRLAIVQ-------HTEFQ-AGFQSGRSTSENIFLL---RTILE----SLPAG---------KESMYIALLDFRKAFDSVNHTVL
CGLLRDLGLPERLTGYVESIYRS-------------VHLTLGDD--------------WFVQGRGVLQGDPISPFLFNLMIDYILSGTQA
GVGVG----------VGERLVSSLAYADDLALLASSRRGLNANLESVLARA-RSVNLALGIN---------KCATIGKR---WLGREKK-
------MILDREPFL--LEG---VAIPVYRWNNIYKYLGIETSPGAA--------ARWSVTGL----RNRLSKLESAC-LKPQQRMHLLR
CYLLPGLYYGLIH-------QGISVGLLRS-------------ADKQVRAA----------ARKILHL
```

>RaR2
NNTAAGPDG-IQPKSWNRISL---------KYKTLIYNLLLYY---EKVPHKLK-VSRTVFIPKKKD--GSSDPGEFRPLTICSVVLRGF
NKILVQRLVSLYK------YDERQ-TAYLPIDGVGTNIHVL---AAILN----DSNTK---------LSELHVALLDITKAFNRLHHTSI
IKSLVGKGFPYGFITFIRRMYTG------------LQTMMQFEG----------HCKMTQVNRGVYQGDPLSGPIFLLAIEKGLQALDK
EVGYD----------IGDVRVNAGAYADDTDLVAGTRLGLQDNINRFSSTI-KQVGLEVNPR---------KSMTLSLV---PSGKEKK-
-----MKVETGKPFR--AND---VPLKELSINDFWRYLGISYTNEG---------PERLSLTI----EQDLERLTKAP-LKPQQRIHMLN
AYVIPKYQDKLVL-------SKTTAKGLKR------------TDRQIRQY---------VRRWLKL
>R8Hm-A
KDSASGPDG-LLLQDVRRLGP---------LLLCNIFNMWYL----HGIPVEEN-RCRTILLYKSG---DRHLASNYRPVTIGNMLNRLY
AKIWDKRIRKNVR------LHVRQ-KAFIPVDGCFENVKTI---QCVLQ----SYRKR---------KLEHNVVFIDLAKAFDTVLHDSI
RKALWRKGVPSGVVKVVDSLYAG------------AVTSISVGK---------TKTRSICINSGVKQGCPLSPLLFNLILDELAERIEA
TGCGLD---------LDGHVLSSMAFADDYVLLAKDSVEMNELIRVCSTFF-KEKGLSVNPG---------KCQSLRVL---PVKEKKRS
M-KVLVRPHRWWRIK--DQD---VDIPSMTYDSLGKYLGVSIDPTGK--------IALPIEEW----KNWMTKLKECK-LKPEQKVKILK
EVVCSRVNYVLRM-------SECGISELRS------------WTRFVRNW---------AKNIIHL
>R8Hm-B
KDASGGPDG-LLLQDVKKASP---------RQLCIIFNMWYL----HGIPVVEN-RCRTILLHKGG---EKHLTSNYRPVTIGNMLNRVY
AKIWDRRIRKNLQ------LHVRQ-KAFVPLDGCFENVKTI---QCILQ----SYRRS---------RREHNVVFVDLAKAFDTILHDSI
EKALLRKGIPRSVIKVVDSLYAG------------AVTSITVGK---------TKTRPICINSGVKQGCPLSPLLFNLVIDELAERLEA
TGCGLD---------LEGHVISSMAFADDYVLLAKDSVEMNVLMNVCNTFF-EEKGLAVNPA---------KCQSLRVL---PVK-GKRS
M-KVLTRTHRWWKIN--NQD---VEIPSMTYESVGKYLGVMIDPAGK--------IALPIEEW----KLWLTRLRECK-LKPDQKVKVLK
EVVCARANYVLRM-------SGCGICELRK------------WSRFVRGW---------VKSIIHF
>R24_MR
KQSASGPDK-ITRDDLRKANL---------ADVVGLLNIVFGL---CRTPTLLR-HNRTVLIPKKG---DLSLTSNWRPITVSSIFSRLL
HKILASRLSNNIK------LHHAQ-RGFTPCDGIMSSSTIL---DAIVR----EHRTN---------GKPLYVLSIDLTKAFDKIHPIAI
ENSLINKGVDQHTVKYIMSTYKD------------VDTTIECHG---------ERSSPIRMCRGVRQGDTDSPMLFNIAIDDLVTSINT
TDGVQ----------LGNSKIGCLLFADDIIFVSNTVHGINEHLRRLRAFL-EGTHMEVNPS---------KCRALQLA---RVPGTKR-
-----VVVDTKPKFS--IDG---ASVPTLRVLEQLKYLGHGYSQSGM--------LAPSPSNL----ESMLERLRRAA-LRPWQKLYILN
RYLIPRLIHCCQS-------SSITAGRLEY------------MDRLIRKF---------IKKTLHL
>R2-1_MR
KESAPGIDG-ITREHLRSMSR---------PDLYALLNIIWGM---KCLPPVLR-SNRTSLIPKSG---NTRELKQWRPIMVSSRVLRLL
NKVIVSRLENEIK------LNYSQ-RGFTKIDGVMANNTIL---QTAIR----TMRNS---------SKPFIILSIDLAKAFDRVSIVSI
IDALWRRGVDEHTICYIEANYRN------------ITTTLECHG---------KRSRPINVDRGVKQGDPISGFLFNVVLDGLLQKLHD
QTGIQ----------INGTNIAALAFADDIVLMAPTPMIMRSHIRIVESYF-RRHGFEVNVE---------KCATFQYI---PVPGTKR-
-----LVVETKPLFK--ING---TDVPTLNVSSQLKYLGLQYGFRGA--------TCPTPSKT----EEMLSRLGKSP-LKPWQKLNVLQ
RYLIPRLHHGMQM-------MDVNKKKLTY------------LDGCIVKF---------VKETLHL
>R2Sm-A
HGTAGGIDR-LTSYDLMRFGK---------NGLAGYLNMLLAL---AYLPTNLS-TARVTFVPKSS---SPVSPEDFRPISVAPVATRCL
HKILAKRWMPLFP------QERLQ-FAFLNRDGCFEAVNLL---HSVIR----HVHTR---------HAGASFALLDISRAFDTVSHDSI
IRAAKRYGAPELLCRYLNNYYRR------------STSCVN--------------RTELHPTCGVKQGDPLSPLLFIMVLDELLEGLDP
MTHLT----------VDGESLNYIAYADDLVVFAPNAELLQRKLDRISLLL-HEAGWSINPE---------KSRTLDLI---SGGHSK--
-----ITALSQTEFT--IAG---MRIPPLSAADTFDYLGIKSNFKGR--------CPVAHIDLL----NNYLTEISCAP-LKPQQRMKILK
DNLLPRLLYPLTL-------GIVHLKTLKS------------MDRNIHTA---------IRKWLRL
>R2NS-1_CSi
GNSSPGLDK-LTPRMLRRFNA---------NVLAGYFNLLLLS---GGCPPHLC-RARITLVPKVP---NPTSPDQLRPISVSSILVRCF
HKVLADRWSRRLQ------LPSLQ-FAFLHRDGCLEATSLL---HALLR----HSSAT---------ASNLSLAFVDISKAFDSVSHDTI
VRSAEAFGAPSPLVRYIAQSYEN------------AVAVFP--------------SSEVHCHRGVRQGDPLSPLLFIMAMDEVLGLSMP
QLGYQ----------FHDTLVDGFAFADDWVVCAESQARLKEKLEAAAVEL-GRAGMKINAR---------KTKAMVIC---GDRKHR--
-----ATAVSVEPFC--FAE---ELITPLGPTDTVTYLGIPFTFKGK--------GVFNHRQHL----LKLLDEVTRAP-LKPHQRMEITR
NYLIPRLTYSLVL-------GQVHRNTLKR------------LDNYIRQS---------IRGWLRL
>R2Ci-B
TPSAPGPDK-LTLDGVKRIPI---------AELVSHYNLWLYA---GYQPEGLR-EGITTLIPKIK---GTRDPAKLRPITVSSFICRIF
HRCLAQRMETSLP------LGERQ-KAFRKVDGICHNIWSL---RSLIH----NSKDN---------LKELNITFLDVRKAFDSISHKSL
GIAAARLGLPPPLITYISNLYPN------------CSTKLKVNG---------KISKPIEVRRGVRQGDPLSPLLFNAVMDWALSELDP
RVGVQ----------IGEQRINHLAFADDIILVSSTKIGMVSSINTLSRHL-AKSGLEISAG---------KEGKSASMAIVVDGKKKM-
-----WTVDPLPRFK--VNS---QKIPALSITQQYKYLGINIDAQGA--------RNDAARIL----TEGLAELSRAP-LKPQQRLYLLR
VHLLPKLQHGLVL-------SSCAKRALTY------------LDKSVRSA---------IRRWLTL
>R2Nvec-A
KKSASGPDG-VPCAALQTMGA---------ASLAAHFNLWLLA---GTQPKRLT-ECRTIFVPKEV---NTHLPLHHRPITIGSVVVRLF
HQILGKPMEAVLP------LGSGQ-RGFRKGDGICQNIWLL---HTLIR----RSTDL---------LRPLKLVFLDVKKAFDSVSHESL
LIAAKRLGVPGPLLTYINELYSR------------SETVFEVGG---------ESSGSVKVSQGVKQGDPLSSTLFNCVIDWAVSDLDP
HIGVL----------LGESRVSFLAYADDLVLLSETEAALTSQLNSIEKSL-AHCGLKLSTG---------DSGKSASLNIVIDGKAKR-
-----WVVNPTPFLR--ASG---GEIRSLVANETYKYLGINIGAQGV--------KAAEYNAF----KEALDNLSRAP-LKPQQRLFLLK
TYLLPQLHHSLVL-------SRTTGKLLNS------------LDALVRKA---------VRGWLKL
>R2-1_MDe
ADSAAGPDG-ITVVNWIGMDI---------RLRLLFLNMIMKR---GRLDDDLK-SARTVLIPKGK---GNIRPENTRPLSITSVVLRHL
HKILAKRLKSLQT------FSDSQ-KAFIDCDGTQENLAIL---NTLLT----DARKN---------LKSIHIATLDLRKAFDSVSHKTI
IDSITGMGCPKMFIDYVTDLYLN------------ARTVFQYNG----------SSTFLDIKRGVLQGDPLSPLLFNLVMDRAIVKLSA
NIGYT----------YNGAIINCIAYTDDIILIAETKCGLQSLIDALTSEL-ASFGLQTNIG---------KSSTISLV---PSGRQKK-
-----IKVITEPSFL--VSG---QFLSAIGVMDVWRYLGVDFRCNV---------LNGSEYGI----DANLRKLDRAP-LKPQQRLKMLC
VAIIPRYLHSLVL-------GRVNKTKLAG------------FDNLIRKY---------VRKWLYL
>R2A_NVi
KRTAAGPDG-MTTTAWNSIDE----------CIKSLFNMIMYH---GQCPRRYL-DSRTVLIPKEP---GTMDPACFRPLSIASVALRHF
HRILANRIGEHGL------LDTRQ-RAFIVADGVAENTSLL---SAMIK----EARMK---------IKGLYIAILDVKKAFDSVEHRSI
LDALRRKKLPLEMRNYIMWVYRN------------SKTRLEVVK---------TKGRWIRPARGVRQGDPLSPLLFNCVMDAVLRRLPE
NTGFL----------MGAEKIGALVFADDLVLLAETREGLQASLSRIEAGL-QEQGLEMMPR---------KCHTLALV---PSGKEKK-
-----IKVETHKPFT--VGN---QEITQLGHADQWKYLGVVYNSYG---------PIQVKINI----AGDLQRVTAAP-LKPQQRMAILG
MFLIPRFIHKLVL-------GRTSNADVRK------------GDKIIRKT---------VRGWLRL
>R2-1_TCas
QISAPGSDQ-IPVAAVKTMSE---------LELAILFNIILFR---NVQPSAWG-VLRTTLVPKDG---DLRNPANWRPITISSALQRLL
HRVLAARLSKLVS------LSSSQ-RGFTEIDGTLANALIL---HEYLQ----YRRQT---------GRTYXVVSLDVRKAFDTVSHCSV
SRALGRFGIPSVIREYILATFGA-------------QTTIKCGS---------VTTRPIRMLRGVRQGDPLSPVLFNLVMDELLEKVNE
KYEGGSL--------QSGERCAIMAFADDLILIADRDQDVPAMFDDVSTFL-ERRGMSVNPA---------KCRALIAG---AVSGR---
-----SVVRTGSSYK--IHN---TPIPNVDALDAFKYLGLEFGHKGV--------ERPTIHNL----SVWLNNLRRAP-LKPDQKCLFIR
QYVIPRLLYGMQN-------PQVTSRVLRE------------ADRLIRRH---------LKTYYHL
>R2LcA
SSTAPGVDG-FSAKQLRSMSP---------RVLNKILNLLLLS---ENLPNSFK-MHKTVLIPKID---DPKSPGDFRPITISPVLARLL
NKILAARLSKLVP------ISQRQ-KAFLPVDGCGENIFLL---DYILR----SSKKS---------SKSVAMAVLDVKKAFDSVSHHSI
LRALNEAKCPINFINFVRNSYDG------------CTTKLTCGG--------TSFPDSVRMNRGVKQGDPLSPVLFNLIIDSAIRKLPD
SIGYVI---------RDGLKINCLAYADDLILVASSRAGLKTLLNIVAEHL-SLRGLDLNAA---------KCHGLSII---ASGKAKT-
-----TYVSAADSLD--LDG---QPIKNLGVLDTWTYLGIPFSHLGR--------AEKVSPDL----TNLLNKLQKAP-LKLQQKLYAVR
NFVIPRALHGLIL-------SKTNLKELNT------------LDRAIRVF---------LRTLLYL

```
>R2Ll
PSTAPGVDG-FSAKQLRSMTP---------RVLNKILNLLLLC---ENLPNSFK-MHKTVLIPKIV---DPKSPGDFRPITISPVLARHL
NKILAARLSKLVP------ISQRQ-KAFLPVXGCGENIFLL---DYILX----NSKKK---------SKSVVMAVLDVKXAFDSVSHHSI
LRALNEAKCPVNLXNFVRNSYDG------------CTTKLTCGG--------TSSPDSVRMNRGVKQXXPLSPVLFNLIIDSAIRKLPD
SIXYLI---------RDGLKINCLAYADDLILVASTRAGLKTLLNIVAEHL-SLRGLDLNAA---------KCHGLSII---ASGKAKT-
-----TYVXALESLD--LDG---QPIKNLRVLDTWTYLGIPFSHLGR--------AXKFSPDL----SNLLNKLQKAP-LKIQQKLYAVR
NFIIPRALHGLIL-------SRTNLKELNT------------LDRAIRVF---------IRTLLHL
>R2La
SSTSPGPDG-VTARMLRSIPA---------RVLNKLLNLLLFI---EDLPAVFK-CHRTVLIPKID---NPTSPGEFRPITISSIVVRQL
NKIIAARVSEGVP------INPRQ-KAFRQIDGCAENVFLL---DFILR----DAKTK---------IKSLSLATVDIKKAFDSVSHHSI
FRAIRGARCPENLVNYIQNSYSG------------CTTQISVGG--------SISASKIPMNRGVKQGDPLSPVLFNLVINEIIRKLPA
SIGYPI---------NSELSINCIAYADDLILVTNTREGLKLLLGLLNEEL-PKRGLELNAS---------KCFGLSLT---ALGKLKK-
-----THLCTSDQLD--LHG---TLIKNLTAEESWVYLGVPFSHIGR--------SKSFSPDL----EALLNKLQKSP-LKLQQKLFALR
VYLIPRLLHGLVL-------SRVAIGELKI------------MDKLILKH---------LRVWLRL
>R2Lc-B
TSTSPGPDG-VTARMLRSIPA---------RVLNKLLNLLLFI---EDLPAVFK-CHRTVLIPKVD---NPALPGEFRPITISSIIVRQL
NKIIAARVSEGVP------INPRQ-KAFRQIDGCAENVFLL---DFILR----DAKTK---------IKSLSLATVDIKKAFDSVSHHSI
FRAIRGARCPENLVNYIQNSYSG------------CTTQISVGG--------SISTTKILMNRGVKQGDPLSPVLFNLVINEIIRKLPA
SIGYPI---------NSELSINCIAYADDLILVANTREGLKLLLNLLNEEL-PKRGLELNAS---------KCFGLSLT---ALGKLKK-
-----THLCTSDQLD--LHG---TLIKNLTAEESWVYLGVPFSHIGR--------SKSFSPDL----EALLNKLQKSP-LKLQQKLFALR
VYLIPRLLHGLVL-------SRVAIGELKI------------MDKLILKH---------LRVWLRL
>R2NS-1_SMed
LKTSSGPDN-ISTEVLREIPV---------LVLVKLFNLIILL---SHLPSEFK-SSRTTLIPKVE---TPKDPGDYRPISVSSVFVRAL
NKILASRVSNGNK------GAHAQ-KGFKALDGCLENTVLL---NCILG----DAKIN---------NKSIALSFIDMKKAFDSVNHDSI
LRAATIAGYPKLLLGYIKESYNG------------ADTEVEK-------------IKATFKRGVKQGDPLSPVLFNNIVELAIKKAYE
SGIGYR---------MNGTVFPIMAYADDIILLSESKEGLQDLLDIVLTEL-RHNGLEANAD---------KCASIIAK---KDGKQK--
-----KWVISSDSVL--AAG---KTMPCMNANSMYKYLGIYFGPLGI-------TTERLTKIY----SDQLYNIGIAP-LKPQQRLEMIN
DFLLPGILHEAVL-------GNTNVGELKD------------LDKMTRVA---------IRKWLHL
>R2-1_PPap
PGSAAGIDR-ISVQQFQRCPV---------HVRVLLFNVLLLV---GHLPGRMS-CARTVFLPKVE---GSSDPKDYRPISITSVITRQF
HKILAARLTSMHA------WDERQ-AGFLPVDGCGENLAIL---NELIR----FSRVN---------RRELHLASLDISKAFDMVPRQAI
INSVAQLGAPQNLVEYLKGLYAN------------NQTTLEYGG----------SELYCRVKRGVRQGDPLSPLLFNLVMESALVRLDK
KLSFS----------LYGVSVNGLAYADDVILVASTSGGLQKNTESFLGAL-REIGLDLNLA---------KCKSLSLK---PSGRDKR-
-----CKVLSESQLS--IGG---TSVPQVDLVGFWRYLGIWFSGPRV--------VSPEQLSM----GVYLERISKAP-LKPQQRIRILV
DYLLPKYTHGSVL-------GRYTRKTYKA------------MDAQIRSY---------VRKWLHL
>R2-2_TCas
NGAAPGPDG-IAVSVWNKLPP---------EAAALLFNVLLLG---RCLPAELT-RTRTVFIPKTD---APRTPADYRPISIASVVARHF
HRVLSARVQRIPDLF----TKYQR-GFLSGVDGIADNLSVL---DTMLT----MSRRC---------CKHLHLAALDVSKAFDTVSHFAI
VRACRSIFG--------------------------SAETVLEEG---------GRRHFVQVRXGVRQXDPLSPLLFNLVLDRALKRLST
DVGFRL---------TDATKVTALAFADDVVLCATTARGLQTNLDVLEAEL-RLAGLLLNPN---------KCQALSLV---ASGRDHK-
-----VKLVTKPTFK--VGQ---NTIHQVDASSIWKYLGIQFRGSGM--------CGCGSEGV----AAGLKRITCAP-LKPQQRMHLLR
VFFLPKFYHAWTF-------GRLNAGVLRR------------LDVVVRTS---------VRTWLRL
>R2-1_PBa
NGTAPGLDG-ITPRAWNAVPV---------GLRALVFNLLLLA---ETAPLSIT-TTRTVFLEKGGM-SDRPGPSEYRPLSIGSVIIRHL
HKILAKRLAALDI------FDARQ-RGFRPVDGVCENITVL---SSVLG----DARRR---------CKSLHVACVDLSKAFDTVSHAAI
HRTLEELGLPREFRDYVRAIYAE------------ARTVLQPTG--------RQRMSPIHVGRGVRQGDPLSPLLFNLVVDRALGILSE
DVGYR----------LESRLINALGYADDIVLLSSTKIGLQENLTRLHAAF-LQNGLTINAN---------KTGVLSMV---ASGRDKK-
-----VKIDMTPYFT--VGG---ALIPQRSPVDVWTYLGGMYQGAR---------EYASVPPL----ANSIRHITKAP-LKPQQRLRLLR
DCLLPRYYHRWII-------GTITSKILKE------------MDVLLRAA---------VRRWLRL
>R28_MR
PQSAPGPDG-IGVRQWRAVPP---------SVRALLYNIVLAT---GGFSPALL-TSRTVFVPKKSG--DPDSPGDYRPISVTSVVVRQL
HKILANRLRASGL--------VDL-RQRCFDDGCGENIAAL---AAVIH----DSRSH---------LRELHVASLDIAKAFDSVSHQAI
ARVLTTLGVPCELVSYVTTMYAR------------SSTMFEVRG---------QRSELFPVGRGVRQGDPLSPILFCLVVDSILRTVPP
DVGYD----------LGGSRLNALAYADDFLLFAASVWGMQKTLGLVEQRA-GEYGLTFNAQ---------KCSVLSLV---PSGKDKK-
-----MKVITTPLFH-LGRQ---AFIPQITTTAEWRYLGVDFRSTG---------PKKSRIDL----TTYLDRIGKAP-LKPQQRLKLLR
CFLLPRLYHVLVL-------GRTTLGALRA------------LDVLTRAA---------VRKWLRL
>R2-7_MR
LDTAVGPDG-VTARQWRAVLP---------AVRALLYNIILKR---GSFPASML-ESRTVFLPKKQ---HSVNPADFRPISIASVVVRQL
HKILAMRLRRTNL--------VDE-RQRCMDDGCAENITVL---ASLLD----DARHG---------LKELHLVSLDCAKAFDSVSHHAI
DATLKECGLPAGFVQYISRTYSD------------SSTRLEVGR---------NRSEPIKTNRGVRQGDPLSTLIFCLCFDRVARTLSP
HIGYD----------LNNTRISTLLYADDAFLVSTTAPGMNILLRSVEESA-GEVGLSFNTS---------KCSALSLI---PSGKEKK-
-----MKVGTTPTFK--TSQ---GFITQITPSQEWRYLGVDFQYSG---------PKKASRSL----KIELERISKAP-LKPQQRLLILR
VYLLPRYYHHLVL-------SRTTLGHLRG------------LDLQVRAA---------VRRWLSL
>R2-1_IS
VNSAAGPDG-FSARLLKSVPA---------LLLRVMVNLLLLV---RRVPAALR-DARTTFIPKVP---DAVDPSQFRPITVASVLQRLL
HRILAKRALEAIP------LNFRQ-RAFQPVDGCAENIWLL---STALN----EARTR---------RRPLHMASVDLTKAFDRVTTDAI
LRGARRAGLSGEFIGYLKELYTT------------SRTLLQFQG----------ESLLVEPTTGVRQGDPLSPILFNLVLDEYLSSLDP
DISFV----------SGDLRLDAMAFADDLIVFASTPAGLQDRLDALVEFF-DPRGLRVNVK---------KSFTLSLQ---PGRDKK--
-----VKVVCDQIFT--IGG---TPLPASKVATPWRYLGMTFTPQGS-------INKGTSEQL----DLLLTRTSKAP-LKPQQRLVVLR
NYLLPRLYHRLVL-------GPWSAALLLK------------MDTTIRGA---------IRRWMDL
>R2-1_RMi
SSSAAGPDG-FAARELRKVPP---------VILQVLLNLLLLL---KRLPLFLT-QARTIFIPKVP---GASTASQHRPISISHVLLRLL
HKIYFRRLLADLD------LDLQQ-RAFLPVDGCAENILLL---ATIID----EARKS---------LRPLSLASLDIAKAFDRVVLPAI
LRALRRKGISEDFISYIEDFYQN------------AVTVLTFGG----------KSLVVHPTVGVRQGDPLSPLLFNLVIDEFLAELDP
QLAFT----------SEGMKVSAMAFADDIILTTATHWGLKQQIDRLNSFL-GARGLKINAA---------KSTTLVIE---PSGWQKR-
-----SKIRTDIDFF--VNG---ERLATTNCTSTWRYLGVHFGVKGL-------EKGLVRRQL----AILLERVSKAP-LKPQQRLVVLR
FYLLPRLYHRLVL-------GPISAKTLLT------------IDRVVRSA---------VRRWLAL
>R2_FA
MTTSPGPDG-ITVRQLYLVPE---------QLLVRILNLLMAC---GKMPDSFL-ESKTTLIPKKP---NSTEPGDFRPITVQSVLVRQL
NKILAARVAQHIP------LDERQ-RGFRPVDGVAHNIFEL---DMILR----CHRSE---------FRDLRLASLDIAKAFDSITHNTI
EDTMEVRGFPKPMINYIMACYRR------------SKTRFTFNG---------WISDTVKPTCGVKQGDPLSPILFNLVMDRMIRKLPK
EVGVN----------VGSKHYNGLTFADDLLLFATTPEGLQSSIDIVHLFL-LECGLLINKQ---------KSFVLTVK---AYPKLKK-
-----TAVIVTEKYM--LDR---HILPAIDREKLFHYLGVPFTAEGR-------CRDDTIAHL----KRKIDVLTKAP-LKPQQRLFALR
VVILPSCYHILTL-------GGSNLSLLKK------------IDLMVRAA---------GRKWCCL
>R2-5_MR
MTTAPGPDG-LTARQIRAVPL---------EVLVRIFNIFLVC---GRLPKHLL-ESRTILIPKKD---NASEPGDFRPITISSVMTRTY
HKVIARRFAGSIE------LDKRQ-KAFXPVDGCAENIFDL---DMVLR----YHRES---------FKSIFMASTDVAKAFDSVSHRAM
YDTLRSKGVPAPLVEYVRDVYER------------STTRLTGSD----------WQSDLIRCGVKQGDPLPPIIFNMIIDRLLKLIPP
EIGVD----------VGESHFNALAFADDLLFMASTTKGLQTCLDLASEYL-LKCGLRVNAX---------KSFTVALR---NVPHVKK-
-----SXVDGKVRFR--CLG---HEMPSLTREDQWRYLGVPFTPEGR-------SSARADEQL----KQTLGKLTSAP-LKPQQRLFALR
VVVLXGLHHLLSL-------GNVTLSLLRK------------VDKLVRAA---------VRKWLDL
```

```
>R2_Dan
LSSSPGPDG-ITPKTARSVPE---------GIMLRIMNLILWC---GNLPYSIR-LARTIFIPKKA---TANQPQDYRPISVPSVIVRQL
NAILASRLSAAIN------WDTRQ-RGFLPTDGCADNTTIV---DLVLR----EHHKR---------FKSCYIGTLDVSKAFDAVAHEAV
YNTLASYGAPKGFINYLRKAYEG-------------GGTMLAGNG---------WVSEAFIPARGVKQGDPLSPILFNLVIDRLLRSLPS
EIGAK----------VGNAMTNAAAFADDIVLFAETPMGLQKLLDTTVCFL-SSVGLTLNTD---------KCFTVSIK---GQAKQK--
-----CTVVERRSFL--IGG---RECPSLKRTDEWKYLGIKFTAEGR-------ARYDPAEDL----GPKLLRLTRAP-LKPQQKLFALR
TVLIPQLYHKLTL-------GSVTIGVLKK-------------FDKLVRYT----------ARKWLGL
>R2_DSi
LSSSPGPDG-ITPKSAREVPS---------GIMLRIMNLILWC---GNLPHSIR-LARTVFIPKTV---TAKRPQDFRPISVPSVLVRQL
NAILATRLNSSIN------WDPRQ-RGFLPTDGCADNATIV---DLVLR----HSHKH---------FRSCYIANLDVSKAFDSLSHASI
YDTLRAYGAPKGFVDYVQNTYEG-------------GGTSLNGDG---------WSSEEFVPARGVKQGDPLSPILFNLVMDRLLRNLPS
EIGAK----------VGNAITNAAAFADDLVLFAETRMGLQVLLDKTLDFL-SLVGLKLNAD---------KCFTVGIK---GQPKQK--
-----CTVLEAQSFY--VGS---REIPSLKRTDEWKYLGINFTATGR-------VRCNPAEDI----GPKLQRLTKAP-LKPQQRMFALR
TVLIPQLYHKLAL-------GSVAIGVLRK-------------TDKLIRYY----------VRRWLNL
>R2B_DM
LTSSPGPDG-ITSQTARSIPI---------GIMLRIVNLILWC---GDLPVPFR-MARTIFIPKTV---RANRPQDFRPISVPSIVVRQL
NAILASRLTAAVS------WDPRQ-RGFLPTDGCADNATIV---DLVLR----DHHKR---------YASCYIATLDVSKAFDSVAHDAV
FNTVTAYGAPKSFVDYVRRWYSG-------------GGTYFNGGD---------WRSEEFVPARGVKQGDPLSPVLFNLIIDRLLRSLPK
DIGVH----------VGNAKVNACAFADDLMLFASTPKGLQELLNTTVKFL-SSVGLTLNAD---------KCFTISIK---GQPKQK--
-----VTVVEQRTFC--IG----RARVQLKRSEEWKYLGIHFTADGR-------ARYNPSEDI----GPKLERLMQSP-LKPQQKLFALR
TVLVPQLYHKLTL-------GSVALGVLRK-------------CDKLVRSF----------ARKLLGL
>R2_DPe
LTKSPGPDG-ITPRTVRSIPS---------GVMLRIMNLILWC---GKLPVSIR-QARTIFIPKVG---NASRPQDFRPITVQSVMVRIL
NAILASRLTSSVD------WDPRQ-RGFLPTDGCADNTTIV---DLILR----DHHKR---------CKSLYIATLDISKAFDSVSHAAV
SATLTAYGAPKEFVDYVQNSYEV-------------CGTTLNGDG---------WRSEEFIPARGVRQGDPLSPIIFNLIIDQLLRSYPN
EIGAT----------IGDHTTNAAAFADDIVLFAETRLGLQTMLDTTVDFL-SSVGLTLNSD---------KCFTVGIK---GQPKQK--
-----CTVVIPETFR--IGS---RSCPALKRTDEWKYLGITFTAQGR-------TRYSPADDL----GPKLLRLTRSP-LKPQQKLFALR
TVLIPQLYHKLTL-------GSVMIGVLRK-------------CDILVRST----------VRKWLGL
>R2_BM
WRTSPGPDG-IRSGQWRAVPV---------HLKAEMFNAWMAR---GEIPEILR-QCRTVFVPKVE---RPGGPGEYRPISIASIPLRHF
HSILARRLLACCP------PDARQ-RGFICADGTLENSAVL---DAVLG----DSRKK---------LRECHVAVLDFAKAFDTVSHEAL
VELLRLRGMPEQFCGYIAHLYDT-------------ASTTLAVNN---------EMSSPVKVGRGVRQGDPLSPILFNVVMDLILASLPE
RVGYR----------LEMELVSALAYADDLVLLAGSKVGMQESISAVDCVG-RQMGLRLNCR---------KSAVLSMI---PDGHRKK-
-----HHYLTERTFN--IGG---KPLRQVSCVERWRYLGVDFEASG---------CVTLEHSI----SSALNNISRAP-LKPQQRLEILR
AHLIPRFQHGFVL-------GNISDDRLRM-------------LDVQIRKA----------VGQWLRL
>R2C_NGi
QNTAAGIDG-LTVGDLKGVSR---------EMLARIFNLFMWC---GKLPEHLC-ASRTILLPKKP---GAKVPGEFRPITVTSVLIRTF
HKVLAERLKVVP-------LDPRQ-RGFRESDGCAENVMLL---DMTIR----YHHER---------RRKMFLALLDMAKAFDSVSFESM
REVLTTKGIPTPFIEYFMTHLED-------------SFTVLQHGN---------WQSGKIHPTCGVKQGDPLSPPIFNFIMDEMLKRLPK
EIGVN----------LDGLFVNAMAFADDLSLVANTEQGLQILIDEATSFL-GLCGLRANPN---------KCVTLAIK---TIPKEKK-
-----TAIDPSSHFR--IGN---AVIPSLKRTDEWVYLGIKFNSNGR-------LISDAKPKL----IKDLELLTKAP-LKPQQRLWALK
VIVIPGILYRGTL-------GSSTAGYLRS-------------LDCVIRAY----------VRRWLRL
>R2_AM
SSGATGPDG-FSVRSLKCTPS---------RVLAKVFNLFLLE---EKLPAFLM-TSRTVLVPKVK---EPKAPTDYRPISVSSTLVRLF
HKILARRLTLASG------LDSRQ-RGFVPVDGCAENLVVL---ESAIR----SAKNY---------KRSLFVASMDIKNAFGSVAHEAI
FEALSKSGAPDSFVTYVRNCYDG-------------FASVVKLG----------RDTAQTTVRQGVLQGDPLSPILFNLVIDQIIRSLPE
TVGVQL---------DANTKLNSMAFADDLILLSSSEAGMRRMLGVLAGVS-SKFGLIFHPG---------KCKYLAMI---WAGKQKK-
-----MKIATDLSFE--IGG---GFMTPVGVTETWKYLGAYLGQIG---------IQPARLSL----QTFLERIAKSP-LKPQQKLYLIR
VHLLPKLIYPLVM-------APIRASMLNK-------------LDRMVRVA----------LTGKDGI
>R2Ci-D
VASASGPDG-VSVSGMTKIGA---------RVLCMLFNVWQFT---GRTPRWTK-ENRTTLIAKSAS--AAEDVGDWRPITIGSHVVRLY
AKLLSRRLGSCVQ------LNLCQ-RAFRDVQGCAENVMLL---SGMIR----DAKAR---------SRDLNLVFLDLAKAFDTVSHSSI
VKALRRQQVPEKFVRIVQDLYDG-------------AVTTVSVGG---------RATPQLVVKRGVKQGCPLSPLLFNLVLDELIDELSS
SFGYTL---------DDGSKLSVMAFADDLVLSSGSLAGLRKLIETTQQFL-LRCGMGLNVQ---------NCTSLGWK---SMVGGKQ-
-----VRLRTEPFVR--VGD---SELACLGPNSTCRYLGIHFGMVGS--------CRIRRREI----RESLLRLASSA-LKPEQKVFMLR
SFLIPRWRYSVTL-------TRVTVSFLSW-------------IDRVVRMR----------VRAILHA
>R2-1_BTe
KKAAAGPDG-LQRDHLTIPGL--------PIIMAKIYNILVYC---SYFPSAWK-ENRTTLIPKINK--PCSLVENWRPITISPILGRIF
SSIIDGRIRRGTV------LNMRQ-KGFTSENGCKINIELL---NSALN----YSKR----------NSGGIFTIVDISKVFDTVPHAAL
KPCLAKKGVPALIVDLIDEMYKN-------------VKTTIKTKD----------GGVEIMIRRGVKQGDPLSPLLFNLCLEPLLEEIEE
QASGINV--------SEHRKVSVLAFADDIVLLGADAREAQHQINVLTDYL-QSLMMNLSIE---------KCQTFEVV---AKKD----
-----TWFIKEPGLK--IGN---QIMPTVDPDEAFKYLGAKIGPWKGV------HCGVIVPEL----LSVVKRVRKLS-LKPGQKVELLT
KYIFPRYIYHLLV-------SPPSDTVLKL-------------LDSEVRQE----------VKTILHL
>R2-2_MR
PKSAAGPDG-LMKRDVIKSGH--------LYILYPMFNLIVAA---GRQPDAWR-KNITKLLNKEGK--DPSLPGSYRPITISPLFSRIF
WGLIDQQLRLRIN------ITPRQ-KGFVPEMGCFLNVNLL---NEIIR----IAKR----------KQGIVITQVDISRAFDTVPFSAI
LNALNAKGLPKVVVQLIMDSYSN-------------ISTQIQYQS----------STFDISIKRGVKQGDPLSPLIFNIVMESLILKLES
MPGFKI---------NDRTNLSSLAFADDLMFLADNVEDARAQLNLLCQFL-DSVDMDISID---------KCAAFQII---TSKD----
-----SWYIIDPHLT--VAQ---GSIAFLGPDTPLSYLGAKISPWVGV------KTDYSIQGL----KACVGRVQSLA-LKPHQKVELMA
RHIIPHYLYGLIL-------SIPSISVIRQ-------------LDQCVRVA----------VKEYLHL
>R2_KF
KDYAPGPDG-VTKLKVQSMGA-------YPSLLAKVYNLVMLT---GYFSSCWK-EHKTSLIPKDRG--SPMDVSNWRPITIGSLLSRIY
TGLIERRLRTVSD------IHQRQ-VGFMPVNGCAANLFIF---DECIR----QAKK----------EGTIVGSLIDVAKAFDTVPHEAI
LRALSSQGVDEHTMAHIRDMYSG-------------IRTRINGKG------------SDIPLVRGVKQGDPLSPMLFNMVMDPLIRDLQR
KGFR-----------IGGHEIGALAFADDIVLLADSIDGAQDHVDQVGRYM-NKLGMTLNPR---------KSSSFLIT---AMRK----
-----TWICRDPGLS--IGE---TKVPGARPSSALKYLGVNYTLSEGL------ESGALIDKL----MQAVNRARGLA-LKPLQKVNLIL
ERIIPKFLYGIIL-------GGPSLTRLHA-------------ADKCVRMA----------VKEILHL
>R2B_NVi
NDSAAGPDG-VTKDDLRGRGV--------SIALSKLFNSILLA---GYYPKAWR-ENRTTLLPKPEK--DPADVKNWRPITISSMVSRVY
SGLLDQRVRAVIK------QCDRQ-KGFTEENGCFSNIQLL---DDAVS----NAK-----------KAGGVITILDVSKAFDTVPHAVI
QGCLEKKGIPETVAAYISSMYRD-------------CSTAIRTRS----------GDVKIGMKRGVKQGDPLSPLIFNLVLEPLLERLQE
TSGVE----------IEGMNLSCAAFADDIVCFANTAPEAGRQLRMVADYL-GRLDMSLSVS---------KCIAVEYV---PHRK----
-----TWYTKNPGLE--VNG---NAVPSISPSETFKYLGAKVSPWKGL------LEGFESDAF----REVISRVQRLP-LKPMQKVDLLQ
MYIFPRYTYGLIT-------SPPAKAVLKT-------------IDRIIRTR----------IKEILHL
>R2Amel
SDTAAGADQ-IKKFHLRKKGA--------LHVFAKLCNLLMLH---RIYPAQWK-TNRTTLIPKPGK--SAEEVENWRPITIGSLLGRIY
SAMIDRKLRSKIK------QHIRQ-KGFTQEDGCKNNIAIL---SSALT----KMKE----------DSGGIITIIDISKAFDTVPHGEI
SQSLMNKGVPSPICEYIQKMYIG-------------CKTIIYCRD---------KKTLPVDILRGVKQGDPLSPLLFNLIIDPIIGTLDE
TTEGIK---------LENENISVLAFADDLVLLAKDKETADKQNRLINEYL-DDLKMKVSAE---------KCTTFEIK---RQNK----
-----TWFLGDPQLT--LGQ---QRIPYADPEAAIKYLGTNFNPWRG-------LCKTSIKEI----IDAARTVKQLK-LKPHQKINLIR
TYLLPRYIHKLVA-------NPPPLGTLDL-------------IDKELKTI----------IKEILHL
```

>R2_Rm_Ribo
NASAPGPDG-VKKCHVATRAK--------QAVLSALFNTVLLT---GILPAEWS-TNRTTMIPKEGK--DASLLANWRPLTISSLVSRIF
WGCVDTRMRRVVR------LTPRQ-KGFVAEAGCFNNVVLL---DSVLR----HAKC----------GGGVVAVQLDISKAFDTIPHCTI
ATALLNKGLPPFLANLVSRAYVG------------QRTHLNVRG---------EKVEVRNPLVIVKQGDPLSPMLFNLIMEPMINELES
KPGYRI---------DEETQISSLVFADDLILLASDAPKAQVLLSTVHRYL-CNLGMSLAAS---------KSFAFQVT---RTQDSWCL
VDPGLKLSKGDEERC--HNL---EGIPFAGPVDRLRYLGVSVSPWFGI------DIRETRANL----TEVLHKLCRLA-LKPWQKHHLLI
THLVPHYLPELVA-------AAPALTTLRE------------LDQELRVV---------VKKIFHL
>R2_Bg_Ribo
STTAPGPDG-INIQAVRSAPA--------QAVLSTFYSLILLL---GRPPAAWR-ENRSVLIPKEGY--DVRLISNYRPLTIGSILSRIF
WGIIDRRLRELLK------IHRYQ-KGFTTEPGCYQNVRIF---SEVLR----QAKS----------ARGLVAVQLDISKAFDTVPHAAI
ATVLRQKGLPPYFADMVRRSYEG------------AYTSIRVRD----------HRIQLELKRGVKQGDPISPLLFNMIMEPLLNKLES
LGGVTL---------ADGSQLACLAFADDLILLADNRERARELLETVEEFL-DGLGMKIWAP---------KSVAFEIV---PTKDS---
-----WCVVDPGLRL--RQG---GAITYAGPDHSLKYLGVKVSPWVGV------DVSDLRTHF----SKTLEDTRKLP-LKPHQKLHLLK
TYLIPHFTHRLVS-------SLPSIAELRA------------LDLEIRGC---------VKQILHL
>R2_RU
PSSAPGLDG-VGKIHLVGKGI--------TLVLAKLYNLLFLT---GGYPECWK-RNRTVFIPKIGK--DLSEVGGWRPLTIGSLLARMY
SAFLERRIRRVTS------LSSSQ-RGFTNIQGCHVNLTIL---KEGIR----QAKV----------KNGGVIVSVDIEKAFDTIPHSVI
FSRLASQGVPPLLRKIISNMYKD------------VYTVIEGQ-------------CIPIKRGVKQGDPLFPLLFNIAIDPVLRSLEE
FQGGLP---------LGNSAIKILAFDDDIILGASSAGQAQQMVDMLGIGL-TSCGLGVSHR---------KCFGFQIV---NKNK----
-----TWAIVDPMIT--LNG---SSLPFSGPEDRLPYLGVDTNPWDRK------SRYDAGQRL----ISAAKRGSQLS-LKPQQKINLIT
TFLLPKFLYILIE-------DPPSPAYLKS------------IDHDLRQI---------YKNILHL
>R2_RL
PSSAPGLDG-VRKIHLVGKGI--------TLVLVKLYNLLFLT---GGYPECWK-RNRTVFIPKIGK--DLSEVGGWRPLTIGSLLARMY
SAFLERRIRRVTS------LSLSQ-RGFTNIQGCHVNLTIL---KEGIR----QAKV----------KNGGVIVSVDIEKAFDTIPHSVI
FSRLASQGVPPLLRKIISNMYKD------------VYTVIEGQ-------------CIPIKRGVKQGDPLSPLLFNIAIDPVLRSLEE
FQGGLP---------LGNSAIKILAFADDIILGASSAGQAQQMVDMLGIGL-TSCGLGVSHR---------KCFGFQIV---NKNK----
-----TWTIVDPMIT--LNG---SSLPFSGPEDRLPYLGVDINPWDRK------SRYDAGQRL----ISAAKRGSQLS-LKPQQKINLIT
AFLLPKFLYILIE-------DPPSPAYLKS------------IDHDLRQI---------YKNILHL
>R2Dr
NASTPGPDG-VGKRDISNWDP-------ECETLTQLFNMWWFT---GVIPSRLK-KSRTVLLPKSSDPGAEMEIGNWRPITIGSMVLRLF
TRVINTRLTEACP------LHPRQ-RGFRRSPGCSENLEVL---ECLLR----HSKEK---------RSQLAVVFVDFAQAFDTVSHEHM
LSVLEQMNVDPHMVNLIREIYTN------------SCTSVELGR---------KEGPDIPVRVGVKQGDPLSPLLFNLALDPLIQSLER
TGKGCE---------AEGHKVTALAFADDLALVAGSWEGMAHNLALVDEFC-LTTGLTVQPK---------KCHSFMVR---PCRGA---
-----FTVNDCPPWV--LGG---KALQLTNIENSIKYLGVKVNPWAGI------EKPDLTVAL----DRWCKRIGKSL-LKPSQKVYILN
QFAIPRLFYLADH-------GGAGDVMLQN------------LDGTIRKA---------VKKWLHL
>R2-1_GA
NRSSPGPDG-ITKVALSKWDP-------EGIKLAHMYSTWLVS---AGIPKVFK-KCRTTLIPKTGDVSLHGDVGQWRPITIASLVLRLY
SRILTERMTVACP------SHPRQ-RGFIASPGCSENLMLL---EGCMS----LSKAG---------NGSLAVVFVDFAKAFDTVSHEHL
LSVLVQKGLDQHMVELIKDSYEN------------SVTKVHCQE---------GCSTDIAMKVGVKQGDSMSPLLFNLALDPLIQQLER
EGRGFP---------VNGKSITAMAFADDLAIVSDSWEGMRANLDILVDFC-ELTGMRTQPS---------KCHGFLIE---KSGSRS--
-----YKVNRCEPWL--LND---TALHMVGPKESIKYLGVQVNPWTGI------FAEDTVAKL----RQWVVAISKTP-LRPLDKVSLLC
QFAVPRVIFVADH-------CMLSAKALTE------------MDRSIRQA---------VKRWLHL
>R2-1_SSa
NNTAAGPDG-ITKPALLEWDP-------TGAKLAAIFSIWLTS---GTLPGPFK-KCRTTLIPKTDDPILLTQVAGWRPLTIGSVVLRLY
SRILTHRLERACP------INPRQ-RGFISSPGCSENLMIL---GGLIK----RSWAK---------GERLAVVLVDFARAFDSVSHSHI
LEILRQRGLDEHIIGIVGDSYTD------------VTTTITVSG---------EQSPPIDMRVGVKQGDPMSPLLFNLALDPMIDTLER
YGLGYR---------MGEQQITALAFADDLVLVSDSWEGMACNIRILEEFC-RLTGLRIQPR---------KCHGFLIQ---KIQRA---
-----RSVNLCKPWI--VCG---EELHMVGPEESVSYLGMKVSPWHGI------MEPDPVERL----CNWISSIGRSP-LKPSQKVRMLN
VYAAPRMTYQADH-------GGLGPIVLNV------------LDGMIRKA---------VKVWLHL
>R2Ol-A
NGTAPGPDR-ISKKALLDWDP-------RGEQLARLYTTWLIG---GVIPRVFK-ECRTKLLPKSSDPVELQDIGGWRPVTIGSMVTRLF
SRILTMRLTRACP------INPRQRGFLASSSGCAENLLIF---DEIVR----RSRRD---------GGPLAVVFVDFARAFDSISHEHI
LCVLEEGGLDRHVIGLIRNSYVD------------CVTRVGCVE---------GMTPPIQMKVGVKQGDPMSPLLFNLAMDPLIHKLET
AGTGLK---------WGDLSIATLAFADDLVLVSDSEEGMGRSLGILEKFC-QLTGLRVQPR---------KCHGFFMD---KG------
------VVNGCGTWE--ICG---SPIHMIPPGESVRYLGVQVGPGRGV------MEPDLIPTV----HTWIERITEAP-LKPSQRMRVLN
SFALPRIIYQADL-------RKVTVTKLAQ------------IDGIVRKA---------VKKWLHL
>R2-1_Gav
SKSAPGPDK-LTLRDLRRADP-------EGDALAELFSLWLIT---GTVPDGLK-ECRSVLIPKTVDREKLGQLGNWRPITIGSIVLRLF
SRVLTARLAAACP------INPRQ-RGFIAAPGCAENLKVL---ELLLR----KRKRD---------RQPLGVVFVDLARAFDSVSHDHI
SWVLKAKGVDEHIVNLIEDSYQK------------VTTRVQVFN---------GVTPPISIKTGVKQGDPMSPLLFNIAMDPLIAKLET
DGQGVK---------VGSASLTTLAFADDLVLLSDSWEGMLKNISILEDFC-NLTGLRVQPK---------KCQGFFLN---PTCDS---
-----FTVNNCEAWK--IAG---REITMLGPGESTRYLGLNVGPWVGI------DKPDLGTQL----SSWLERIGTAP-LKPMQKLSLLV
QYAIPRLNYQADY-------AGIGRVALEA------------LDSMNRRK---------VKEWFHL
>R2-1_ZA
KGSAPGPDG-IALGDIKKMDP-------GYSRTAELFNLWLTA---GDIPDMVR-GCRTVLIPKSTTPERLKDINNWRPITIGSILLRLF
SRIITARMTKACP------LNPRQ-RGFISAPGCSENLKLL---QSIIR----TAKNE---------HKPLGVIFVDIAKAFDTVSHQHI
IHVLQQRRVDPHIVGLVNNMYKD------------ISTYVTTKK--------NTHTDKIQIRVGVKQGDPLSPLLFNLAMDPLLCKLEE
SGKGFH---------RGQSSITAMAFADDLVLLSDSWENMKENIKILETFC-NLTGLKTQGQ---------KCHGFYIK---PTKDS---
-----YTINNCPAWT--ING---TPLNMINPGESEKYLGLQIDPWTGV------AKYDLSTKL----KIWLESIDRAP-LKPLQKLDILK
TYTIPRLTYLADH-------SEMKAGALEA------------LDQQIRTA---------VKDWLHL
>R2-1_TG
KGSAPGPDG-ITLGDVVKMDP-------EFSRTMEIFNLWLTT---GKIPDMVR-GCRTVLIPKSSKPDRLKDINNWRPITIGSILLRLF
SRIVTARLSKACP------LNPRQ-RGFIRAAGCSENLKLL---QTIIW----SAKRE---------HRPLGVVFVDIAKAFDTVSHQHI
IHALQQREVDPHIVGLVSNMYEN------------ISTYITTKR--------NTHTDKIQIRVGVKQGDPMSPLLFNLAMDPLLCKLEE
SGKGYH---------RGQSSITAMAFADDLVLLSDSWENMNTNISILETFC-NLTGLKTQGQ---------KCHGFYIK---PTKDS---
-----YTINDCAAWT--ING---TPLNMIDPGESEKYLGLQFDPWIGI------ARSGLSTKL----DFWLQRIDQAP-LKPLQKTDILK
TYTIPRLIYIADH-------SEVKTALLET------------LDQKIRTA---------VKEWLHL
>R2-1_GFo
KGSAPGPDG-IALGDIRKMDP-------EYTRTAELFNLWLTS---GEIPDMVR-GCRTVLIPKSSKPERLKDINNWRPITIGSILLRLF
SRIITARLTKACP------LNPRQ-RSFISAAGCSENLKLL---QTIIR----TAKNE---------HRPLGVVFVDIAKAFDTVSHQHI
IHVLQRRRVDPHIIGLVKNMYKD------------ISTVITTKK--------NTYTDKIQIQVGVKQGDPLSPLLFNLAMDPLLCKLEE
HGKGFH---------RGQSKITAMAFADDLVLLSDSWEDMNANIKILETFC-DLTGLKTQGQ---------KCHGFYIK---PTKDS---
-----YTVNNCAAWT--ING---TPLNMINPGESEKYLGLQFDPWVGI------AKTSLPEKL----DFWLERIDRAP-LKPFQKLDILK
TYTIPRLTYVADH-------SEMKAGALEA------------LDRTIRSA---------VKDWLHL
>R2NS1_CGi
KDSAAGPDE-IELKTVMAKDP-------KGXILVNLFNSFLYR---KKVPEIFK-GNRSILLPKGND--GLEDVNNWRPLTISSSVLRLY
TSLLARRVLDSCA------LNPRQ-RGLIAASGCSENSFLL---SEMIN----HAKKE---------RRQLCVTFLDLAKAFDTVSHKHF
IAGLRRFGSPEHFIDIVADLYNG------------ASTVFQTDA---------GQTGEIPMTRGVKQGDPLSPLLFNIAMDPLLEVIGR
QNNGY-KFGP-----EESDRIESLCYADDNALMTESPDEMNENLALVEKFC-CETGMRLNIK---------KSATFCIT---PCGSRSY-
-----TVNTTKTKVA--IKG---ERVPVIPPDGCMKYLGSKMSPWVTK------IRKDIVAQL----EGMVESIGGAH-LKPRQKLVLLN
HYALVRLTYALTQ-------DAYPXXVLGK------------LDLIVRAA---------IRRWAKL

```
>NeSL-1_TV
HLSAHGLDG-IPNSVYMLFPVS------AAKFLSILFRSIIIS---GHIPDCWK-LSKTVMLFKKD---DPSLAKNWRPIGITSCTYRIF
MTLVNKALQMIPM------FHAMQ-KGFVRGATLSEHIAVA---NEVLC----QSTRT---------QSEMFQTAIDFTNAFGTVPHQLI
FDSLEAKKVPDSIINLLKDLYKG------------ARTAIYTRH---------AHSEIVPVRRGVIQGCPLSPILFNCCLDPLLYAVQR
RHFEDGYRFQD---KAGQYSIAIQAYADDVLVISPTHEGMQRILNTVDEFQ-KIAKLKVAPQ---------KCVTLAKT---STA-----
----------IQPFR--IGP---DEIPIKTSMDNITYLGIPISGTKTSRF---AAATGILEKV----KAQIRVVFASH-LALSQKIIALR
VFILPQLDFYMFH-------NVFRVNDLKA------------TDQMIRGL---------IDKEAPT
>Utopia-1_ACa
NGSAPGIDG-IPYEAYKRTKL--------DATLAHVFEVVRLN---ARLPARWD-VARTVLLYKKG---DPNDTGNWRPISLQVTIYKIF
TAALSKRLISWAGKHNT--FSASQ-KGFLPAEGCHEHAFVL---RSVLD----DARRH---------KQNVYLAWYDLRNAFGSVSHDLI
AWCAAMLGLPRYLRDAIGAIYRH------------SALFVQVGD--------QETTGVIPMRCGVKQGCPLSPLLFNLCVEPALRCLRR
TTGYKFY--------GTSITVEGQAYADDLLTAAPSAYHAARQVATIEEWA-NWAGVSFVVQALSLDAPAGKCAALAIN---FEGG----
-----LMHSIDPALK--VQG---AAIPAMSRNNVYRYLGVHVGLTDALG-----QANELLEKA----SRDARTICASG-LEPWQKVVAIK
TFILSRLPFFFHN-------GKIQRGRCQQ------------FDRELREN---------LRAALRL
>Utopia-1_LV
TNSAPGKDR-LEYRHIRRADG-------SFSISEAIFNKCLAE---GRIPAPWK-TASTILLHKAG---PTDDPANFRPIALQSCLYKLF
MAVLADRLTKWACENQY--LSPEQ-KSARPCEGCFEHSFLL---SAALK----DCRRN---------QKTICIGWLDLRNAFGSIPHPVI
KIVLSSLGVPDSLVTLLMDAYNG------------ASTSFTLTG---------GQTDTVPIRSGVKQGCPMSPILFNLAIELIIRAVKK
NASDNHLGVT-----VQGKNLSILAYADDLVLLSRDTEGLQSLLQVAGSSA-STLQMQFKPQ---------KCATLTLD---CKR-----
-----GTNVRQSAHH--IQG---AAIPSLTEEERYRYLGVPIGLPRLT------SLQESSRKL----SSDIETISSSL-LAPWQKLDAIK
TFVIPILQYTLRA-------TEYLKSDLKP------------LRAAIIKH---------VKKICHL
>Utopia-1_CFl
TNSAPGPDG-ITYRDLRGADP-------GARLLAALFNACYRL---EAVPASWK-TSNTVLVYKKG---DRDSLENWRPLALGDTAPKLF
AALIADRMTEWAVTNNK--LCPAQ-KGFLRDEGCFEHNFVL---QEILT----DARRT---------RRQAVVAWLDLSNAFGSVPHAAI
RRALVRSGVPGGLVNVWNSMYDG------------CSTRVRTAE---------GYTTPVLIRSGVRQGCPLSPIVFDLAIDSVLRAVTA
VDAGFD---------LLGSRHSVVAYADDIALVADSPEGMRRLLAVAEGAA-SSVGLRFNPA---------KCATLHLA---AGS-----
-----GGRVLPTTFV--IQG---QPVHHLSSGEPYRHLGVPTGSSVDQ------TPFATIGGL----LEDLRAVDQSL-LAPWQKLEVVA
TNILPRLDFLLRG-------ATVGKKPLKA------------ADLQVRRM---------AKSWLNL
>Utopia-1_AEc
NNSAPGPDG-VSYRDLRGADR-------GARLLTALYNICLRL---EAVPASWK-TSNTVLIHKKG---DRGMLENWRPLALGDTVPKLF
AALLADRLTDWAVTRGK--LCSAQ-KGFLRDEGCYEHNFVL---QEVLT----HAKRS---------KRQAVVAWLDLSNAFGSIPHATI
RRALIRSAVPRGLIAIWDSMYDG------------CTTRVRTAE---------GHTAPIPIRSGVRQGCPLSPIIFNLAIDSVVRVAAE
XNDGYS---------LHGNTWSALAYADDIALLAQTPEGMERMLASVEAEA-ASVGLRFNPA---------KCATLHVG---AGN-----
-----GGRVLPTSFQ--IQG---ETINPLAQGESYTHLGVPTGFSVDQ------TPYAAVGDI----VSDLRAVDRSL-LAPWQKIEMLG
TFILSRLDFLLRG-------ARVFKGPLTA------------VDLNIRRH---------VKSWLNL
>Utopia-1_CMy
KNTAPGKDG-IPYSLLKKRDP-------GCLVLATLFNQCKRF---CRTPSSWK-KAMTVLVYKKG---ERDDPSNWRPISLCSTMYKLY
ASCLASRITEWSVSGGA--ISSIQ-KGFMSCEGCYEHNFVL---QTTIE----TARRA---------RRQCAVAWLDLANAFGSMPHHHI
FATLQEFGMPENFLRVIREVYEG------------CSTTIRSVE---------GETAEIPIRSGVKQGCPLSPIIFNLAMEPLLRAISN
GTDGFN---------LHGERVSVLAYADDLVLTADDPESLQGMLDATSRAA-DWMGLRFNAK---------KCATLHID---GSK-----
-----RDSVQTTGFQ--IQG---EPVIPLAEGQAYQHLGTPTGFRVRQ------TPEDTIQEI----LQDAAKIDASL-LAPWQKINALN
TFLIPRISFVLRG-------SAVAKVPLNK------------ADKIVRQL---------VKKWLFL
>Utopia1B_CPB
KNTAPGKDG-IRYHLLKKRDP-------GCLVLAAIFTKCKQF---HRVPRSWK-KSMTVLIHKKG---ERDDPGNWRPISLCSTIYKLY
ASCLAARITDWSVCGGA--VSSVQ-KGFMSCEGCYEHNFLL---QTAIQ----EARRS---------KRQCAVAWLDLTNAFGSIPHHHI
FATLGEFGMPETFIQILRDLYKD------------CTTTIRATD---------GETDAIPIRRGVKQGCPLSPIIFNLAMEPLIRAISS
GPTGFD---------LHGKKISILAYADDLALVADSSESLQQMLDVTSQAA-EWMGLRFNPK---------KCASLHVD---GGA-----
-----RALVRPSRFL--IQG---EPMASLEEGEVYQHLGTPTGVRVRQ------TPEDTIAEI----LRDAAQIDSSL-LAPWQKINALN
TFLIPRISFVLRG-------SAVAKVPLNK------------ADSTIRQL---------VKKWLYL
>Utopia-1_ENe
LRSAAGPDG-IRFSVYKQFADV------LIPAMSAIYNACSKH---RQVPRKWK-ESVTVLIPKGG---DPKSVKNWRPINLQDCIYKLY
AALWAQKIAAWAIKSGV--ASKSQ-KGFMPVNGCHEHLFLA---QSILN----STRRS---------KKPLYMTYYDLQNAFGSISHQLI
HVVLQAQRLPKHAQEVIMDLYQG------------ASFCILTKE---------GCTGRIVNQRGVKQGCPLSPILFNLAIEPLLQRLAA
CNEGLELRTTD---GKPQVKVSHMAYADDLKTVAATRVGISKLHQVVEDFL-QWTGLKANPS---------KCATLGLK---VQKA----
-----KQVPDPVKLT--LHY---EVLPVVKLGEAYKYLGIKDALESPVQQ---SQILRAMSRI----KKDLNKLLRSE-LLPWQKLDALR
TFVMSRLDYYLRH-------CYPYKQQLVA------------FDVYVRAA---------LKAAFKL
>R5-2_SM
DNTSPGIDG-IQYKDLALLDP-------EGILLSFLFNKIISS---KIIPTSWK-TFKTILIPKPDKTDNYDKVSSWRPIALLSVIYKVF
ASILTTRLTSWVICNDI--LHIGQ-KGGSVHEGCVEHNSIL---SSALE----HSKYS--------KNSPLAIAWLDIKDAFGSVPHDYL
WSVLKTIGVSEEFITIVKLLYTD------------TQSFYSCGP---------IVTPNLSIKKGVKQGCPLSMILFSIAINPVLEAISR
SCIEPFM--------IGDSPVQVLAYADDIALVANNVENLQKIVDVAVEAA-TEIGFEYRPE---------KCGYMQLP---RVN-----
---------INGEIL--INE---KEIKKLLSKEFYQYLGVPVGEDNDQ------SPYAILDKV----VSDTKKIADSG-LFGWQKLKAYK
IFIHSRLTFAFRT-------REIKTMALSA------------SQGNTNSCGNNSSKLRGHLRRILNL
>R5
SKSAAGLDW-ITYEDIKLGDP-------SGEILQPIFEYIVQN---NICPSEGK-ASRTIMIPKPGK-SDYSDPSSWRPITITSAVYRLL
MKYLTWELYNWILLNQM--LSRSQ-KSLGKFEGCHDHNAML---NMLIQ----DVRRQTNPSNPINKNKRLYIVFLDFTNAFGSVPLDTL
MYVPQRFGLGTSALTLIKNLYLD------------NYTNVTCGE---------SKIENVKLNKGVKQGCPLSMLLFNIFINIIIRAIEA
MPDVHGYP-------LGDMDIRILAYADDIALISDSHKDLQEMVYKAEYIG-RILGLLFNPS---------KCALMDIP---HDK-----
--------KRTPPIL--VNG---EMIKCVGKADPYKYLGTFRSWFRKL------DIKELLQMM----MDETKLITESN-LHPHQKIHAYE
TFIHSQLPFHLRH-------SRIPFSDFITNRKTNKTTNNSNDSEKSIQKAYDPE--SGQLFLNTFAL
>R5-1_SM
THSAPGSDK-ITFSDWRRLDP-------DFEFLAALFNNILRT---GKSPNKWK-TFRTKLIIKPGKEHSPHEVSSWRPLAILDTAYRFF
ATILNNRLLSWIAKRNL--LCSNQ-KAVGIPDGCAEHNTVI---SLAKE----WAVIN---------GKDINIVWLDLADAFGSVPHDLI
WHSLSRLKLKNKTINLIKDMYND------------CYTVYECEG---------KHTKTIKVNNGVKQGCPMSMTLFSLTINFILQNILK
EYPLI----------IHNHNISIMAYADDLVLIADTREKMRKMIKDITKYT-DSATLKFRPS---------KCGYFQLK---RNH-----
---------NDPPIT--LYD---EQIPIIDENHLYKYLGVDFGQKGKH------NIDTILNTA----LDDTTKILSSD-LHPAQKLQAYK
TFIHSRLIFPFRN-------CNINHMTLDT------------NRNRTVQH---------REKQLGF
>LIN9_SM
TDSAPGEDN-IPLRQWIIWNN--------DGVLFDMFNYIKRT---HDIPDMWK-NYTTTLLIKPGKSQESNIPANWRPISILPTSYRIF
MKVLNKRVLEWANRGEL--ISKWQ-KAVDKANGCDEHSYVIQALIEKAN----RSYYK---------NEQCHLAFLDLADAFGSIPFQVI
WHTLKNMGMDEETINLLKEIYKD------------CSTKYKCGK---------NESEKIKITKGVRQGCPLSMTLFSLCIQYLIQGIAE
KKKGAT---------IAGQEVCILAYADDLVIVANTAKDMQMLLTTIENLA-KQADLIFKPA---------KCGYYRDP---RDK-----
----------KSMMK--IYG---KEISIVDEKNVYTYLGVRIGDTKKK------DLNVRFEEV----KKKTTAIFKSK-LRSDQKLEAYN
IFCQSKFVYILQG-------EDIAKTKIET------------YDEEIKKM---------IKEDILK
>R4_Hmel
NWKAPGTDN-IHNYWYKKLTCT------HSLLLKHINQFIQSP---CTLPLFIT-NGITYMLPKGL---DPTNPANYRPITCLQTIYKII
TACITDIIYKHIDQNNI--LAEQQ-KGCRKNSQGCKEQLTI----DAIV----MKQAH---------NKNXNTMYIDYRKAFDSVPHSWL
LYILKKYKIHPILITFLSSVMLS------------WKTRLKLINNN-----ETLITDWIKIQRGIFQGDALSPLWFCLALNPLSELLNN
TNTGFKLKHN-----NTYHIISHLMYMDDIKLYASNNKELKILADLTQSFS-TDIRMEFGIE---------KCKVHSIK---RGKS----
--------QQNTYIL--NTG---EQIESMDENSTYKYLGFQQAKQIQQK----QTKIELTNKF----KFRLNQILRSQ-LNSRNIIKAIN
TYAIPILTYSFAI-------INWSQTDLSN------------LQRIINTH---------MTTHRKH
```

```
>R4-2_AS
PFKAAGPDG-IHAYWWKHLPS-------AGKLLGNIIVEWLVT---GKVSCRWLMRGRTVLLPKKG---DLRLPQNYRPITCLNTCYKLL
TATIAKALLRHLDKGEA--IPQQQ-RALRKKEWGCTHAILL---DRAVA----TDATTQ-------KGRPLSIAWLDFKKAFDSLSHSYI
RWILESVNVPKTITATFFKLMAG-------------WETRYELKYKTKRGCMSIQRSQPVKIVNGIFQGDALSPLLFILSVSPISFALEN
KAKPYRSSVGQ--LSGKGFSLGHQFYVDDLKIYAPNRRELVNALVVAKNIS-TAIGLDLNVE---------KCAQAHYT---PNVQ----
--------QLEVETLADEEP--LMELPVLGLRQSYRYLGIDQKFLATKN-----ALQRFEESL----FNRASAIFRSD-LTWRQMSNAFQ
SIALGGLRYGFLN-------TNGAGQKLSE-------------ALKHARDI----------DVRIRSL
>R4_AL
PWKAPGPDG-LHAHWWKALPS-------AKRLLGELVVDWLTT---GKVTTGWMCRGRTILIPKKG---DRGDPSNYRPITCLNTCYKVL
TSVMNSVILSHLSRGEA--LPMNQ-RAMRKREWGCTHAMVL---DRAMV----MDAMAQ-------KKHSLSVAWLDYRKAYDSVSHEYI
RWAINSVNIPRSVQLTLKRLMSD-------------WETRFESTQCR-----PKLRSDKMKVLNGIFQGDSLSPTLFVLCIAPISYALNK
GVGQCQSSSGW--SAGYGFEIGHQFYMDDLKLYARTPAMLDSQIQVVSEVS-EAMGLHLNLS---------KCAKAHYA---PHGA----
--------GGAQEAVEGAEGSRKGEIPILGLRSTYKYLGVEQRLLPMEV-----ALKEFEDKF----MDRAETIFASE-LTWGQMATAYN
TIAIAGLRYVYSN-------TNGASPKLLE-------------ALKRAATL----------DTRIRDL
>R4-5_BX
PNKAAGPDG-LPGCVWKCLPNA------SNWLCTWLIKKLACS--RIDTPRWLS-EGRVVLLMKKG---DPNDPANYRPIACLNTCYKLM
TSVIERAIREQVTACPT-LIPSEQ-VANRKGVWGCTHASIV---DRLVTGTAAMSKGQ---------NADLRVLFYDCKKAFDSVSRDHM
FRVLDVAGVDRKIYYLLKSFTMN-------------WTVRYELRRSG-----RVERSAPLRVKRGLLQGDSLSPTWFCLCIAPISHALRL
RNPGPCIRQVAGHSDGGQVHVSHLFYMDDLKVYCTGEAAQVRAERLVPELF-SQIGLCVNES---------KSAAAAGP---SR------
-----------------TLR---SSLPLLGVKDQYKYLGIESGFVANEA----SAYSRMQTTI----LAKVQSILDVEGHTVGQRRIAIR
TVAFPGAAFILGNIILGDKQPNSAKADMLK-------------LDMVIREK----------IRRKGLL
>HERO-1_BF
AGSAPGPNG-VSYQIYKKCPK-------VVRLLWKLFRVAWKK---QCIPSAWR-RAGGVLIPKEK---VSTDIKQFRNISLLNVEGKLF
FSILTKRMITYLLANNYIDTEVQK-AGVPGFPGCIEHASTI---WQQIQ----MARRQ---------KEDLHVVWLDLTNAYGAVPHSVI
HYALNFFWVPETIRTMIQNYFQD-------------FRVSVTTPQ---------FTTGWQQLEKGIAMGCTISPLLFVLGFEIFLIGARQ
VAGGIKLP-------SGQRLPPLRAIMDDVTSILRSAPCTRRVLQRLEELT-DWARMRFKPS---------KSRSLSLR---KGK-----
--------SSNRVFS--INN---QDIPTIQ-QEPVKSLGRLYTSDIADT----KRGQELVKQA----VEGLRAIDKCE-LPGKLKVWCLQ
SVLIPRLKWPMKM-------YDIPLSTADQ-------------VEGKANSF----------VRKWLGV
>HEROTn
NNSAPGPNG-VPYLVYKRCPK-------LLARLWKILRVIWRR---GKVAHQWR-WAEGVWVPKEE---KSTLIEQFRTISLLNVEGKIF
FSILSHRLSDFLLKNQYIDSSVQK-GGIPGVPGCLEHCGVV---TQLIR----EAREG---------RGSLAVLWLDLANAYGAIPHKLV
EMALARHHVPCSIKTLIMDYYDS-------------FHLRFVTSG--------SVTSEWHRLEKGIITGCTISVIIFALAMNMLAKSAEP
ECRGPITK-------SGIRQPPIRAFMDDLTVTTTSVPGCRWILQGLERLM-TWARMRFKPG---------KSRSLVLK---AGK-----
-------VTDRFRFY--LGG---TQIPSVS-EKPVKSLGKMFDGSLKYA----FSIRETNDQL----GHWLTLVDKSG-LPGKFKAWVYQ
HGILPRILWPLLV-------YEFPISTVEG-------------LERRVSSC----------LRRWLGL
>HERO-1_SP
NASSPGQNG-IPYKMYKKCPR-------LLKIIWKLMLDVWRT---KYIPVQWR-VAEGVYIPKSAT-PNEKTITDFRPIALLNVEAKMF
FAIPAKRLETYMRKNGYLDTTVQK-GGVGGHPGVWEHVATL---WEVIK----DAKTS---------QKNLVAIWLDLANAFGSVPHAAI
AFALKWYHLPESLVSLMNNYYVG-------------LYARFSVKD---------WNSEWQKFSVGIFMGCTLSPILFVITFNLLNDFLKS
TPAKQYHLKK-----RDFNVPVMKEYMDDITIIITASVESAGSILSKVNDFM-DWSKMRIKPV---------KSRSLVVH---GGK-----
-------VKHIEPFA--VDG---QTIPGVH-NKPVKFLGRLIDGNLKDN----VARQDMMVSL----GRWLHTIDKCV-LTGVMKCWIYQ
YLIVPRMQWKLMI-------YDVPVSQVEK-------------METLVSKK----------LRRWLGA
>HERO-1_HR
AKATPCPLDQISVIALKRCPI-------LRTYLHKLIVKCWNA---GTIPLCWR-KALTILIHKKG---DANDPANFRPITLQPVLYKIF
STLIKNRLHNFLLQNNFISTNIQK-GFYPTLDGLAEHTQML---SHLMK----DAKRY---------QRSIIIALLDLRNAFGEVNHQLI
NSILLYHHVPQLFRNLILSIYSS-------------YAISVSIND---------QLTPLLPVRKGVLQGDPCSPLLFNLCFNPLMKILEG
DEFKQCGYLWG--PTSDVRARSWLQFADDTAIIAHNSESAQKLIDLNLAWC-RWSDMQMRVD---------KCCVFGMM---KKDG----
-----SYDQFLPRLT--IDN---IPIPPVELGSGFKYLGKIYDAELKDT----KAKEEMVSKL----TRLLKRTTDLK-VSTQMKLQVLR
RFIPSQLKFELRS------YNLSLTWIKNN-------------LDNMVTEA----------VRDWLTM
>HERO3_BF
ATSAPGPDG-IMYGHLKHLPA-------CHLFLSTLFSKLLES---GDPPTSWS-SGNVSLIHKDG---SPEAAENFRMICLTSCVSKIF
HQILSERWAKYMTCNDL-IDPETQKAFLTGINGCVEHVQVM---REILA----HAKKN---------RRTVHITWFDLADAFGSVEHELI
YYQMERNGFPPIITTYIKNLYSR-------------LKGKVKGPG---------WESDPFPFGRGVFQGDNLSPIIFLTVFQPILQHLKG
VEQQHGYN-------LNDKHYVTLPFADDFCLITTNKRQHQKLITQISSNT-KSMNLKLKPR---------KCKSMSIV---SGK-----
--------PSDISFT--IDG---DPVKTT-KDAPEKFLGGYITFLSKTK----ETYDILAKTI----ETTVENINKSA-IRNEYKLRVYM
EYAFPSWRYMLMV-------HDLTDTQLQK-------------LDSIHTKA----------IKTWLRM
>R4-4_SRa
NWKCPGENG-IPIFLIKKLPS-------AKTFVKKWMKGVVEN--RIPITEYDI-KGNAYAIFKKG---DKNIESNYRFINALNSHYKLL
MKYLNKCVREWCSNL----ISDNQ-FSGKQGYDGALDIGIT---NRILQ----EM-------------GQFAQGWYDMQKAYDSVDHSRL
KMIINSVKEIPKPIRNLLCKLPKMYKFSVVNCSPNRDENGKIIKE---------KKKSWITMKRGIYQGCSLAPWLFCLCLAPLQWKMKQ
IIDPIDL--------YGQKKIDILLFMDDIKGFGKNKNELDKITNLIINYG-KEIGLTINKD---------KTEYS-------------
--------------------------IPDEEKIIQYKYMGVYENNKTACDE---INAEQLLMKL----DEKMDKIFNSK-LNVKNIIKAIN
GCVIPTCTYLFSH-------EFS-DEERIQ-------------LARNVDIR----------IRSYMNT
```

B

```
Conservation:                                         14649373 411 132  21           12  322 22      1 1
6ar1_chainD_p001          1   ALLERILARDNLITALKRVEANQGAPGIDG-VSTDQLRDYIR---------AHWSTIHAQLLAG---TYR   57
SLACS                     1   ---------------------RGAAPGLDG-WTRELLYPLTLD----PALKMEIAAVVKDIINADVSMEV   44
CRE1                      1   ---------------------RGTAPGLDG-WTRELLLPLAEDPALLHEITSVVSDIMQGKVA----EVV   44
CRE2                      1   ---------------------RAAAPGLDG-WTRELLVPITED----KGLLTELTALVQDMLVG---NVH   41
GAU97528__Ramazzottius_   1   ---------------------VDSAAGPDR-VLVRVIKQEAVME---VLAVIAAVLLEMNATKE---HPL   42
Blag_1                    1   ---------------------ADTAPGPDH-VLVRALKDATC--------YEIIAKIATTMLQH---SFV   37
Blag_5                    1   ---------------------VDTAPGPDH-VLMRVLKDDAA--------TGVLSLLATRMLRT---GHV   37
Blag_2                    1   ---------------------VDTAPGPDH-VLVRVIRNEIV--------YEILAIIATRMLSS---GIV   37
Blag_4                    1   ---------------------TDSAPGPDR-ILVRTLKLTEC--------SPVIAVILKIMLQW---GMV   37
Blag_3                    1   ---------------------IDTSAGIDH-VLVRTIKELKT--------SKLLCLLTNAMLKF---KIC   37
Blag_6                    1   ---------------------LDTAPGPDR-VLFKVVKELKC--------MKIISIIANIMLSW---NYV   37
R2_PS                     1   ---------------------SKTAKGVDG-VGLEELKRVPR---------RTLFNILNGLW-----THV   34
RaR2                      1   ---------------------NNTAAGPDG-IQPKSWNRISL---------KYKTLIYNLLLYY---EKV   36
R8HmA                     1   ---------------------KDSASGPDG-LLLQDVRRLGP---------LLLCNIFNMWYL----HGI   35
R8HmB                     1   ---------------------KDASGGPDG-LLLQDVKKASP---------RQLCIIFNMWYL----HGI   35
R24_MR                    1   ---------------------KQSASGPDK-ITRDDLRKANL---------ADVVGLLNIVFGL---CRT   36
R21_MR                    1   ---------------------KESAPGIDG-ITREHLRSMSR---------PDLYALLNIIWGM---KCL   36
R2SmA                     1   ---------------------HGTAGGIDR-LTSYDLMRFGK---------NGLAGYLNMLLAL---AYL   36
R2NS1_CSi                 1   ---------------------GNSSPGLDK-LTPRMLRRFNA---------NVLAGYFNLLLLS---GGC   36
R2CiB                     1   ---------------------TPSAPGPDK-LTLDGVKRIPI---------AELVSHYNLWLYA---GYQ   36
R2NvecA                   1   ---------------------KKSASGPDG-VPCAALQTMGA---------ASLAAHFNLWLLA---GTQ   36
```

```
R21_MDe                      1  ---------------------ADSAAGPDG-ITVVNWIGMDI---------RLRLLFLNMIMKR---GRL   36
R2A_NVi                      1  ---------------------KRTAAGPDG-MTTTAWNSIDE----------CIKSLFNMIMYH---GQC   35
R21_TCas                     1  ---------------------QISAPGSDQ-IPVAAVKTMSE---------LELAILFNIILFR---NVQ   36
R2LcA                        1  ---------------------SSTAPGVDG-FSAKQLRSMSP---------RVLNKILNLLLLS---ENL   36
R2Ll                         1  ---------------------PSTAPGVDG-FSAKQLRSMTP---------RVLNKILNLLLLC---ENL   36
R2La                         1  ---------------------SSTSPGPDG-VTARMLRSIPA---------RVLNKLLNLLLFI---EDL   36
R2LcB                        1  ---------------------TSTSPGPDG-VTARMLRSIPA---------RVLNKLLNLLLFI---EDL   36
R2NS1_SMed                   1  ---------------------LKTSSGPDN-ISTEVLREIPV---------LVLVKLFNLIILL---SHL   36
R21_PPap                     1  ---------------------PGSAAGIDR-ISVQQFQRCPV---------HVRVLLFNVLLLV---GHL   36
R22_TCas                     1  ---------------------NGAAPGPDG-IAVSVWNKLPP---------EAAALLFNVLLLG---RCL   36
R21_PBa                      1  ---------------------NGTAPGLDG-ITPRAWNAVPV---------GLRALVFNLLLLA---ETA   36
R28_MR                       1  ---------------------PQSAPGPDG-IGVRQWRAVPP---------SVRALLYNIVLAT---GGF   36
R27_MR                       1  ---------------------LDTAVGPDG-VTARQWRAVLP---------AVRALLYNIILKR---GSF   36
R21_IS                       1  ---------------------VNSAAGPDG-FSARLLKSVPA---------LLLRVMVNLLLLV---RRV   36
R21_RMi                      1  ---------------------SSSAAGPDG-FAARELRKVPP---------VILQVLLNLLLLL---KRL   36
R2_FA                        1  ---------------------MTTSPGPDG-ITVRQLYLVPE---------QLLVRILNLLMAC---GKM   36
R25_MR                       1  ---------------------MTTAPGPDG-LTARQIRAVPL---------EVLVRIFNIFLVC---GNL   36
R2_Dan                       1  ---------------------LSSSPGPDG-ITPKTARSVPE---------GIMLRIMNLILWC---GNL   36
R2_DSi                       1  ---------------------LSSSPGPDG-ITPKSAREVPS---------GIMLRIMNLILWC---GNL   36
R2B_DM                       1  ---------------------LTSSPGPDG-ITSQTARSIPI---------GIMLRIVNLILWC---GDL   36
R2_DPe                       1  ---------------------LTKSPGPDG-ITPRTVRSIPS---------GVMLRIMNLILWC---GKL   36
R2_BM                        1  ---------------------WRTSPGPDG-IRSGQWRAVPV---------HLKAEMFNAWMAR---GEI   36
R2C_NGi                      1  ---------------------QNTAAGIDG-LTVGDLKGVSR---------EMLARIFNLFMWC---GKL   36
R2_AM                        1  ---------------------SSGATGPDG-FSVRSLKCTPS---------RVLAKVFNLFLLE---EKL   36
R2CiD                        1  ---------------------VASASGPDG-VSVSGMTKIGA---------RVLCMLFNVWQFT---GRT   36
R21_BTe                      1  ---------------------KKAAAGPDG-LQRDHLTIPGL--------PIIMAKIYNILVYC---SYF   37
R22_MR                       1  ---------------------PKSAAGPDG-LMKRDVIKSGH--------LYILYPMFNLIVAA---GRQ   37
R2_KF                        1  ---------------------KDYAPGPDG-VTKLKVQSMGA-------YPSLLAKVYNLVMLT---GYF   38
R2B_NVi                      1  ---------------------NDSAAGPDG-VTKDDLRGRGV--------SIALSKLFNSILLA---GYY   37
R2Amel                       1  ---------------------SDTAAGADQ-IKKFHLRKKGA--------LHVFAKLCNLLMLH---RIY   37
R2_Rm_ribo                   1  ---------------------NASAPGPDG-VKKCHVATRAK--------QAVLSALFNTVLLT---GIL   37
R2_Bg_ribo                   1  ---------------------STTAPGPDG-INIQAVRSAPA--------QAVLSTFYSLILLL---GRP   37
R2_RU                        1  ---------------------PSSAPGLDG-VGKIHLVGKGI--------TLVLAKLYNLLFLT---GGY   37
R2_RL                        1  ---------------------PSSAPGLDG-VRKIHLVGKGI--------TLVLVKLYNLLFLT---GGY   37
R2Dr                         1  ---------------------NASTPGPDG-VGKRDISNWDP-------ECETLTQLFNMWWFT---GVI   38
R21_GA                       1  ---------------------NRSSPGPDG-ITKVALSKWDP-------EGIKLAHMYSTWLVS---AGI   38
R21_SSa                      1  ---------------------NNTAAGPDG-ITKPALLEWDP-------TGAKLAAIFSIWLTS---GTL   38
R2OlA                        1  ---------------------NGTAPGPDR-ISKKALLDWDP-------RGEQLARLYTTWLIG---GVI   38
R21_Gav                      1  ---------------------SKSAPGPDK-LTLRDLRRADP-------EGDALAELFSLWLIT---GTV   38
R21_ZA                       1  ---------------------KGSAPGPDG-IALGDIKKMDP-------GYSRTAELFNLWLTA---GDI   38
R21_TG                       1  ---------------------KGSAPGPDG-ITLGDVVKMDP-------EFSRTMEIFNLWLTT---GKI   38
R21_GFo                      1  ---------------------KGSAPGPDG-IALGDIRKMDP-------EYTRTAELFNLWLTS---GEI   38
R2NS1_CGi                    1  ---------------------KDSAAGPDE-IELKTVMAKDP-------KGXILVNLFNSFLYR---KKV   38
NeSL1_TV                     1  ---------------------HLSAHGLDG-IPNSVYMLFPVS------AAKFLSILFRSIIIS---GHI   39
Utopia1_ACa                  1  ---------------------NGSAPGIDG-IPYEAYKRTKL--------DATLAHVFEVVRLN---ARL   37
Utopia1_LV                   1  ---------------------TNSAPGKDR-LEYRHIRRADG-------SFSISEAIFNKCLAE---GRI   38
Utopia1_CFl                  1  ---------------------TNSAPGPDG-ITYRDLRGADP-------GARLLAALFNACYRL---EAV   38
Utopia1_AEc                  1  ---------------------NNSAPGPDG-VSYRDLRGADR-------GARLLTALYNICLRL---EAV   38
Utopia1_CMy                  1  ---------------------KNTAPGKDG-IPYSLLKKRDP-------GCLVLATLFNQCKRF---CRT   38
Utopia1B_CPB                 1  ---------------------KNTAPGKDG-IRYHLLKKRDP-------GCLVLAAIFTKCKQF---HRV   38
Utopia1_ENe                  1  ---------------------LRSAAGPDG-IRFSVYKQFADV------LIPAMSAIYNACSKH---RQV   39
R52_SM                       1  ---------------------DNTSPGIDG-IQYKDLALLDP-------EGILLSFLFNKIISS---KII   38
R5                           1  ---------------------SKSAAGLDW-ITYEDIKLGDP-------SGEILQPIFEYIVQN---NIC   38
R51_SM                       1  ---------------------THSAPGSDK-ITFSDWRRLDP-------DFEFLAALFNNILRT---GKS   38
LIN9_SM                      1  ---------------------TDSAPGEDN-IPLRQWIIWNN--------DGVLFDMFNYIKRT---HDI   37
R4_Hmel                      1  ---------------------NWKAPGTDN-IHNYWYKKLTCT------HSLLLKHINQFIQSP---CTL   39
R42_AS                       1  ---------------------PFKAAGPDG-IHAYWWKHLPS-------AGKLLGNIIVEWLVT---GKV   38
R4_AL                        1  ---------------------PWKAPGPDG-LHAHWWKALPS-------AKRLLGELVVDWLTT---GKV   38
R45_BX                       1  ---------------------PNKAAGPDG-LPGCVWKCLPNA------SNWLCTWLIKKLACS--RIDT   40
HERO1_BF                     1  ---------------------AGSAPGPNG-VSYQIYKKCPK-------VVRLLWKLFRVAWKK---QCI   38
HEROTn                       1  ---------------------NNSAPGPNG-VPYLVYKRCPK-------LLARLWKILRVIWRR---GKV   38
HERO1_SP                     1  ---------------------NASSPGQNG-IPYKMYKKCPR-------LLKIIWKLMLDVWRT---KYI   38
HERO1_HR                     1  ---------------------AKATPCPLDQISVIALKRCPI-------LRTYLHKLIVKCWNA---GTI   39
HERO3_BF                     1  ---------------------ATSAPGPDG-IMYGHLKHLPA-------CHLFLSTLFSKLLES---GDP   38
R44_SRa                      1  ---------------------NWKCPGENG-IPIFLIKKLPS-------AKTFVKKWMKGVVEN--RIPI   39
Consensus_aa:                   .......................sAsG.D..l....h..hs...........hh..lhp.hh.......h
Consensus_ss:                                                hhhhhhhhh         hhhhhhhhhhhhh


Conservation:                   5  22  2244363911   11 1323396734242117242234213411         11  5 3531
6ar1_chainD_p001            58  PA----PVRRVEIPKPG--------GGTRQLGIPTVVDRLIQQAILQELTPIFDP-----DFSSSSFGFR  110
SLACS                       45  GRRLQ-ATSLTVLRKPN--------GKYRPIGAESVWAKLASHIAISRVMKTAEKK----FSGIQ-FGVG  100
CRE1                        45  ARRLR-SSAVTPIPKDE------AGTKIRPIVPESAWLKLASLVAMAEIPSSFKET----FKGWQ-YGVW  102
CRE2                        42  PSFAT-RIRACILHPFR---KEAGSAKVRPITPESALMKLAAHIALDSVEKSFRST----FKGWQ-YGVW  102
GAU97528__Ramazzottius_     43  PSVLQ-LALTVLIHKGG---DVTDGNNYRPISICSARRRVLERVVDRHLRRYGS------VSRFR-QGFT  101
Blag_1                      38  PECLQ-KARTVLIYKGG---DDKDLGNWRPITICSVVRRIIERILDARLRALVN------LCENQ-RGFT   96
Blag_5                      38  PPCLL-IARSILIHKSG---DVNDITNYRPITICSVVRRVIERTLDRRLKEYVS------FNRHQ-RGFT   96
Blag_2                      38  PRCFK-TARTVLIHKAA---DVNILSNWRPITICSVLRRVIERVLDKHLRTFVS------FNEHQ-RGFT   96
Blag_4                      38  PDRMR-GARTILIYKGKG--ERCSVSSWRPISVCSVIRRLIERALDSVLRQYLT------LNNNQ-RGFV   97
Blag_3                      38  PGILK-TARTVLLFKGG---DETKLSNWRPISIFSVICRIIEKVINRRLKSYIE------LSPHQ-RGFI   96
Blag_6                      38  PNSFQ-SGRTILIFKSG---DPNSLKNWRPITIFSILRRIIERALERKIRPFVE------LSTCQ-RGFI   96
R2_PS                       35  PESLY-KGRITLIPKKS---LPELAGDFRPICVLPVVVRLLHRILAKRLAIVQ-------HTEFQ-AGFQ   92
RaR2                        37  PHKLK-VSRTVFIPKKKD--GSSDPGEFRPLTICSVVLRGFNKILVQRLVSLYK------YDERQ-TAYL   96
R8HmA                       36  PVEEN-RCRTILLYKSG---DRHLASNYRPVTIGNMLNRLYAKIWDKRIRKNVR------LHVRQ-KAFI   94
R8HmB                       36  PVVEN-RCRTILLHKGG---EKHLTSNYRPVTIGNMLNRVYAKIWDRRIRKNLQ------LHVRQ-KAFV   94
R24_MR                      37  PTLLR-HNRTVLIPKKG---DLSLTSNWRPITVSSIFSRLLHKILASRLSNNIK------LHHAQ-RGFT   95
R21_MR                      37  PPVLR-SNRTSLIPKSG---NTRELKQWRPIMVSSRVLRLLNKVIVSRLENEIK------LNYSQ-RGFT   95
R2SmA                       37  PTNLS-TARVTFVPKSS---SPVSPEDFRPISVAPVATRCLHKILAKRWMPLFP------QERLQ-FAFL   95
R2NS1_CSi                   37  PPHLC-RARITLVPKVP---NPTSPDQLRPISVSSILVRCFHKVLADRWSRRLQ------LPSLQ-FAFL   95
R2CiB                       37  PEGLR-EGITTLIPKIK---GTRDPAKLRPITVSSFICRIFHRCLAQRMETSLP------LGERQ-KAFR   95
R2NvecA                     37  PKRLT-ECRTIFVPKEV---NTHLPLHHRPITIGSVVVRLFHQILGKPMEAVLP------LGSGQ-RGFR   95
```

```
R21_MDe                     37  DDDLK-SARTVLIPKGK---GNIRPENTRPLSITSVVLRHLHKILAKRLKSLQT------FSDSQ-KAFI   95
R2A_NVi                     36  PRRYL-DSRTVLIPKEP---GTMDPACFRPLSIASVALRHFHRILANRIGEHGL------LDTRQ-RAFI   94
R21_TCas                    37  PSAWG-VLRTTLVPKDG---DLRNPANWRPITISSALQRLLHRVLAARLSKLVS------LSSSQ-RGFT   95
R2LcA                       37  PNSFK-MHKTVLIPKID---DPKSPGDFRPITISPVLARLLNKILAARLSKLVP------ISQRQ-KAFL   95
R2Ll                        37  PNSFK-MHKTVLIPKIV---DPKSPGDFRPITISPVLARHLNKILAARLSKLVP------ISQRQ-KAFL   95
R2La                        37  PAVFK-CHRTVLIPKID---NPTSPGEFRPITISSIVVRQLNKIIAARVSEGVP------INPRQ-KAFR   95
R2LcB                       37  PAVFK-CHRTVLIPKVD---NPALPGEFRPITISSIIVRQLNKIIAARVSEGVP------INPRQ-KAFR   95
R2NS1_SMed                  37  PSEFK-SSRTTLIPKVE---TPKDPGDYRPISVSSVFVRALNKILASRVSNGNK------GAHAQ-KGFK   95
R21_PPap                    37  PGRMS-CARTVFLPKVE---GSSDPKDYRPISITSVITRQFHKILAARLTSMHA------WDERQ-AGFL   95
R22_TCas                    37  PAELT-RTRTVFIPKTD---APRTPADYRPISIASVVARHFHRVLSARVQRIPDLF----TKYQR-GFLS   97
R21_PBa                     37  PLSIT-TTRTVFLEKGGM-SDRPGPSEYRPLSIGSVIIRHLHKILAKRLAALDI------FDARQ-RGFR   97
R28_MR                      37  SPALL-TSRTVFVPKKSG--DPDSPGDYRPISVTSVVVRQLHKILANRLRASGL--------VDL-RQRC   94
R27_MR                      37  PASML-ESRTVFLPKKQ---HSVNPADFRPISIASVVVRQLHKILAMRLRRTNL--------VDE-RQRC   93
R21_IS                      37  PAALR-DARTTFIPKVP---DAVDPSQFRPITVASVLQRLLHRILAKRALEAIP------LNFRQ-RAFQ   95
R21_RMi                     37  PLFLT-QARTIFIPKVP---GASTASQHRPISISHVLLRLLHKIYFRRLLADLD------LDLQQ-RAFL   95
R2_FA                       37  PDSFL-ESKTTLIPKKP---NSTEPGDFRPITVQSVLVRQLNKILAARVAQHIP------LDERQ-RGFR   95
R25_MR                      37  PKHLL-ESRTILIPKKD---NASEPGDFRPITISSVMTRTYHKVIARRFAGSIE------LDKRQ-KAFX   95
R2_Dan                      37  PYSIR-LARTIFIPKKA---TANQPQDYRPISVPSVIVRQLNAILASRLSAAIN------WDTRQ-RGFL   95
R2_DSi                      37  PHSIR-LARTVFIPKTV---TAKRPQDFRPISVPSVLVRQLNAILATRLNSSIN------WDPRQ-RGFL   95
R2B_DM                      37  PVPFR-MARTIFIPKTV---RANRPQDFRPISVPSIVVRQLNAILASRLTAAVS------WDPRQ-RGFL   95
R2_DPe                      37  PVSIR-QARTIFIPKKV---NASRPQDFRPITVQSVMVRILNAILASRLTSSVD------WDPRQ-RGFL   95
R2_BM                       37  PEILR-QCRTVFVPKVE---RPGGPGEYRPISIASIPLRHFHSILARRLLACCP------PDARQ-RGFI   95
R2C_NGi                     37  PEHLC-ASRTILLPKKP---GAKVPGEFRPITVTSVLIRTFHKVLAERLKVVP-------LDPRQ-RGFR   94
R2_AM                       37  PAFLM-TSRTVLVPKVK---EPKAPTDYRPISVSSTLVRLFHKILARRLTLASG------LDSRQ-RGFV   95
R2CiD                       37  PRWTK-ENRTTLIAKSAS--AAEDVGDWRPITIGSHVVRLYAKLLSRRLGSCVQ------LNLCQ-RAFR   96
R21_BTe                     38  PSAWK-ENRTTLIPKINK--PCSLVENWRPITISPILGRIFSSIIDGRIRRGTV------LNMRQ-KGFT   97
R22_MR                      38  PDAWR-KNITKLLNKEGK--DPSLPGSYRPITISPLFSRIFWGLIDQQLRLRIN------ITPRQ-KGFV   97
R2_KF                       39  SSCWK-EHKTSLIPKDRG--SPMDVSNWRPITIGSLLSRIYTGLIERRLRTVSD------IHQRQ-VGFM   98
R2B_NVi                     38  PKAWR-ENRTTLLPKPEK--DPADVKNWRPITISSMVSRVYSGLLDQRVRAVIK------QCDRQ-KGFT   97
R2Amel                      38  PAQWK-TNRTTLIPKPGK--SAEEVENWRPITIGSLLGRIYSAMIDRKLRSKIK------QHIRQ-KGFT   97
R2_Rm_ribo                  38  PAEWS-TNRTTMIPKEGK--DASLLANWRPLTISSLVSRIFWGCVDTRMRRVVR------LTPRQ-KGFV   97
R2_Bg_ribo                  38  PAAWR-ENRSVLIPKEGY--DVRLISNYRPLTIGSILSRIFWGIIDRRLRELLK------IHRYQ-KGFT   97
R2_RU                       38  PECWK-RNRTVFIPKIGK--DLSEVGGWRPLTIGSLLARMYSAFLERRIRRVTS------LSSSQ-RGFT   97
R2_RL                       38  PECWK-RNRTVFIPKIGK--DLSEVGGWRPLTIGSLLARMYSAFLERRIRRVTS------LSLSQ-RGFT   97
R2Dr                        39  PSRLK-KSRTVLLPKSSDPGAEMEIGNWRPITIGSMVLRLFTRVINTRLTEACP------LHPRQ-RGFR  100
R21_GA                      39  PKVFK-KCRTTLIPKTGDVSLHGDVGQWRPITIASLVLRLYSRILTERMTVACP------SHPRQ-RGFI  100
R21_SSa                     39  PGPFK-KCRTTLIPKTDDPILLTQVAGWRPLTIGSVVLRLYSRILTHRLERACP------INPRQ-RGFI  100
R2OlA                       39  PRVFK-ECRTKLLPKSSDPVELQDIGGWRPVTIGSMVTRLFSRILTMRLTRACP------INPRQRGFLA  101
R21_Gav                     39  PDGLK-ECRSVLIPKTVDREKLGQLGNWRPITIGSIVLRLFSRVLTARLAAACP------INPRQ-RGFI  100
R21_ZA                      39  PDMVR-GCRTVLIPKSTTPERLKDINNWRPITIGSILLRLFSRIITARMTKACP------LNPRQ-RGFI  100
R21_TG                      39  PDMVR-GCRTVLIPKSSKPDRLKDINNWRPITIGSILLRLFSRIVTARLSKACP------LNPRQ-RGFI  100
R21_GFo                     39  PDMVR-GCRTVLIPKSSKPERLKDINNWRPITIGSILLRLFSRIITARLTKACP------LNPRQ-RSFI  100
R2NS1_CGi                   39  PEIFK-GNRSILLPKGND--GLEDVNNWRPLTISSSVLRLYTSLLARRVLDSCA------LNPRQ-RGLI   98
NeSL1_TV                    40  PDCWK-LSKTVMLFKKD---DPSLAKNWRPIGITSCTYRIFMTLVNKALQMIPM------FHAMQ-KGFV   98
Utopia1_ACa                 38  PARWD-VARTVLLYKKG---DPNDTGNWRPISLQVTIYKIFTAALSKRLISWAGKHNT--FSASQ-KGFL  100
Utopia1_LV                  39  PAPWK-TASTILLHKAG---PTDDPANFRPIALQSCLYKLFMAVLADRLTKWACENQY--LSPEQ-KSAR  101
Utopia1_CFl                 39  PASWK-TSNTVLVYKKG---DRDSLENWRPLALGDTAPKLFAALIADRMTEWAVTNNK--LCPAQ-KGFL  101
Utopia1_AEc                 39  PASWK-TSNTVLIHKKG---DRGMLENWRPLALGDTVPKLFAALLADRLTDWAVTRGK--LCSAQ-KGFL  101
Utopia1_CMy                 39  PSSWK-KAMTVLVYKKG---ERDDPSNWRPISLCSTMYKLYASCLASRITEWSVSGGA--ISSIQ-KGFM  101
Utopia1B_CPB                39  PRSWK-KSMTVLIHKKG---ERDDPGNWRPISLCSTIYKLYASCLAARITDWSVCGGA--VSSVQ-KGFM  101
Utopia1_ENe                 40  PRKWK-ESVTVLIPKGG---DPKSVKNWRPINLQDCIYKLYAALWAQKIAAWAIKSGV--ASKSQ-KGFM  102
R52_SM                      39  PTSWK-TFKTILIPKPDKTDNYDKVSSWRPIALLSVIYKVFASILTTRLTSWVICNDI--LHIGQ-KGGS  104
R5                          39  PSEGK-ASRTIMIPKPGK-SDYSDPSSWRPITITSAVYRLLMKYLTWELYNWILLNQM--LSRSQ-KSLG  103
R51_SM                      39  PNKWK-TFRTKLIIKPGKEHSPHEVSSWRPLAILDTAYRFFATILNNRLLSWIAKRNL--LCSNQ-KAVG  104
LIN9_SM                     38  PDMWK-NYTTTLLIKPGKSQESNIPANWRPISILPTSYRIFMKVLNKRVLEWANRGEL--ISKWQ-KAVD  103
R4_Hmel                     40  PLFIT-NGITYMLPKGL---DPTNPANYRPITCLQTIYKIITACITDIIYKHIDQNNI--LAEQQ-KGCR  102
R42_AS                      39  SCRWLMRGRTVLLPKKG---DLRLPQNYRPITCLNTCYKLLTATIAKALLRHLDKGEA--IPQQQ-RALR  102
R4_AL                       39  TTGWMCRGRTILIPKKG---DRGDPSNYRPITCLNTCYKVLTSVMNSVILSHLSRGEA--LPMNQ-RAMR  102
R45_BX                      41  PRWLS-EGRVVLLMKKG---DPNDPANYRPIACLNTCYKLMTSVIERAIREQVTACPT-LIPSEQ-VANR  104
HERO1_BF                    39  PSAWR-RAGGVLIPKEK---VSTDIKQFRNISLLNVEGKLFFSILTKRMITYLLANNYIDTEVQK-AGVP  103
HEROTn                      39  AHQWR-WAEGVWVPKEE---KSTLIEQFRTISLLNVEGKIFFSILSHRLSDFLLKNQYIDSSVQK-GGIP  103
HERO1_SP                    39  PVQWR-VAEGVYIPKSAT-PNEKTITDFRPIALLNVEAKMFFAIPAKRLETYMRKNGYLDTTVQK-GGVG  105
HERO1_HR                    40  PLCWR-KALTILIHKKG---DANDPANFRPITLQPVLYKIFSTLIKNRLHNFLLQNNFISTNIQK-GFYP  104
HERO3_BF                    39  PTSWS-SGNVSLIHKDG---SPEAAENFRMICLTSCVSKIFHQILSERWAKYMTCNDL-IDPETQKAFLT  103
R44_SRa                     40  TEYDI-KGNAYAIFKKG---DKNIESNYRFINALNSHYKLLMKYLNKCVREWCSNL----ISDNQ-FSGK  100
Consensus_aa:                   P..hp..sphhhl.K.....p.p...p@RPIsl.shh.+hh..hlspcl...h.......h...Q..th.
Consensus_ss:                    hhh   eeeeee                 hhhhhhhhhhhhhhhhhhhh

Conservation:                     2541252 34   11241      22 1         121213331493358763524 25   24   21
6ar1_chainD_p001           111  PGRNAHDAVRQA---QGYIQ-----------------EGYRYVVDMDLEKFFDRVNHDILMSRVARKVK  159
SLACS                      101  G--------HIE---EAIAK----IRKDF---------ATKGSLAMLDGRNAYNAISRRAILEAVYGDST  146
CRE1                       103  G--------DVA---KAVAK----IRRDS---------EEHEYLVALDGVNAYNTMSRAHILQAVYAEQR  148
CRE2                       103  G------DSTEA---VKRIR----EAYAE---------ASSDTLVALDATNAYNRMSRRHILEAAYAQPE  150
GAU97528__Ramazzottius_    102  SAPGTHVNASIL---GSVSK----KAQAE---------QSTVVVVLLDIKQAFEGILHVHLKKTLRASPL  155
Blag_1                      97  NSPGTLINTSIL---ESILV----AAKSK---------KKDCTIVFLDISKAFDNVGHNHLQNSLSALRI  150
Blag_5                      97  SSPGTLMNTSLL---RSILT----SAKTK---------KCNATLVFLDIRKAFDNVGHLHLQKTLESLPI  150
Blag_2                      97  NNSGTLINTSIL---RKVLK----TTKDN---------KEDVTVVFLDISKAFDNIGHKHLDLTLSAEPI  150
Blag_4                      98  SQLGTSINTSLV---DGCLK----KAKLE---------QRNVSIVMLDVAQAFDSVGLDHLEKCLDSQFL  151
Blag_3                      97  NVPGTHINTSII---NGCLT----RAKKE---------KRNCCIAFLDISKAYDNVGHEHVKRCLLRTNM  150
Blag_6                      97  STPGTFINSSLI---NSCLQ----KSKKE---------KGDCCITFLDVSKAFDSIGHKHIEMSLNSTSI  150
R2_PS                       93  SGRSTSENIFLL---RTILE----SLPAG---------KESMYIALLDFRKAFDSVNHTVLCGLLRDLGL  146
RaR2                        97  PIDGVGTNIHVL---AAILN----DSNTK---------LSELHVALLDITKAFNRLHHTSIIKSLVGKGF  150
R8HmA                       95  PVDGCFENVKTI---QCVLQ----SYRKR---------KLEHNVVFIDLAKAFDTVLHDSIRKALWRKGV  148
R8HmB                       95  PLDGCFENVKTI---QCILQ----SYRRS---------RREHNVVFVDLAKAFDTILHDSIEKALLRKGI  148
R24_MR                      96  PCDGIMSSSTIL---DAIVR----EHRTN---------GKPLYVLSIDLTKAFDKIHPIAIENSLINKGV  149
R21_MR                      96  KIDGVMANNTIL---QTAIR----TMRNS---------SKPFIILSIDLAKAFDRVSIVSIIDALWRRGV  149
R2SmA                       96  NRDGCFEAVNLL---HSVIR----HVHTR---------HAGASFALLDISRAFDTVSHDSIIRAAKRYGA  149
R2NS1_CSi                   96  HRDGCLEATSLL---HALLR----HSSAT---------ASNLSLAFVDISKAFDSVSHDTIVRSAEAFGA  149
R2CiB                       96  KVDGICHNIWSL---RSLIH----NSKDN---------LKELNITFLDVRKAFDSISHKSLGIAAARLGL  149
R2NvecA                     96  KGDGICQNIWLL---HTLIR----RSTDL---------LRPLKLVFLDVKKAFDSVSHESLLIAAKRLGV  149
```

```
R21_MDe                        96  DCDGTQENLAIL---NTLLT----DARKN---------LKSIHIATLDLRKAFDSVSHKTIIDSITGMGC  149
R2A_NVi                        95  VADGVAENTSLL---SAMIK----EARMK---------IKGLYIAILDVKKAFDSVEHRSILDALRRKKL  148
R21_TCas                       96  EIDGTLANALIL---HEYLQ----YRRQT---------GRTYXVVSLDVRKAFDTVSHCSVSRALGRFGI  149
R2LcA                          96  PVDGCGENIFLL---DYILR----SSKKS---------SKSVAMAVLDVKKAFDSVSHHSILRALNEAKC  149
R2Ll                           96  PVXGCGENIFLL---DYILX----NSKKK---------SKSVVMAVLDVKXAFDSVSHHSILRALNEAKC  149
R2La                           96  QIDGCAENVFLL---DFILR----DAKTK---------IKSLSLATVDIKKAFDSVSHHSIFRAIRGARC  149
R2LcB                          96  QIDGCAENVFLL---DFILR----DAKTK---------IKSLSLATVDIKKAFDSVSHHSIFRAIRGARC  149
R2NS1_SMed                     96  ALDGCLENTVLL---NCILG----DAKIN---------NKSIALSFIDMKKAFDSVNHDSILRAATIAGY  149
R21_PPap                       96  PVDGCGENLAIL---NELIR----FSRVN---------RRELHLASLDISKAFDMVPRQAIINSVAQLGA  149
R22_TCas                       98  GVDGIADNLSVL---DTMLT----MSRRC---------CKHLHLAALDVSKAFDTVSHFAIVRACRSIFG  151
R21_PBa                        98  PVDGVCENITVL---SSVLG----DARRR---------CKSLHVACVDLSKAFDTVSHAAIHRTLEELGL  151
R28_MR                         95  FDDGCGENIAAL---AAVIH----DSRSH---------LRELHVASLDIAKAFDSVSHQAIARVLTTLGV  148
R27_MR                         94  MDDGCAENITVL---ASLLD----DARHG---------LKELHLVSLDCAKAFDSVSHHAIDATLKECGL  147
R21_IS                         96  PVDGCAENIWLL---STALN----EARTR---------RRPLHMASVDLTKAFDRVTTDAILRGARRAGL  149
R21_RMi                        96  PVDGCAENILLL---ATIID----EARKS---------LRPLSLASLDIAKAFDRVVLPAILRALRRKGI  149
R2_FA                          96  PVDGVAHNIFEL---DMILR----CHRSE---------FRDLRLASLDIAKAFDSITHNTIEDTMEVRGF  149
R25_MR                         96  PVDGCAENIFDL---DMVLR----YHRES---------FKSIFMASTDVAKAFDSVSHRAMYDTLRSKGV  149
R2_Dan                         96  PTDGCADNTTIV---DLVLR----EHHKR---------FKSCYIGTLDVSKAFDAVAHEAVYNTLASYGA  149
R2_DSi                         96  PTDGCADNATIV---DLVLR----HSHKH---------FRSCYIANLDVSKAFDSLSHASIYDTLRAYGA  149
R2B_DM                         96  PTDGCADNATIV---DLVLR----DHHKR---------YASCYIATLDVSKAFDSVAHDAVFNTVTAYGA  149
R2_DPe                         96  PTDGCADNTTIV---DLILR----DHHKR---------CKSLYIATLDISKAFDSVSHAAVSATLTAYGA  149
R2_BM                          96  CADGTLENSAVL---DAVLG----DSRKK---------LRECHVAVLDFAKAFDTVSHEALVELLRLRGM  149
R2C_NGi                        95  ESDGCAENVMLL---DMTIR----YHHER---------RRKMFLALLDMAKAFDSVSFESMREVLTTKGI  148
R2_AM                          96  PVDGCAENLVVL---ESAIR----SAKNY---------KRSLFVASMDIKNAFGSVAHEAIFEALSKSGA  149
R2CiD                          97  DVQGCAENVMLL---SGMIR----DAKAR---------SRDLNLVFLDLAKAFDTVSHSSIVKALRRQQV  150
R21_BTe                        98  SENGCKINIELL---NSALN----YSKR----------NSGGIFTIVDISKVFDTVPHAALKPCLAKKGV  150
R22_MR                         98  PEMGCFLNVNLL---NEIIR----IAKR----------KQGIVITQVDISRAFDTVPFSAILNALNAKGL  150
R2_KF                          99  PVNGCAANLFIF---DECIR----QAKK----------EGTIVGSLIDVAKAFDTVPHEAILRALSSQGV  151
R2B_NVi                        98  EENGCFSNIQLL---DDAVS----NAK-----------KAGGVITILDVSKAFDTVPHAVIQGCLEKKGI  149
R2Amel                         98  QEDGCKNNIAIL---SSALT----KMKE----------DSGGIITIIDISKAFDTVPHGEISQSLMNKGV  150
R2_Rm_ribo                     98  AEAGCFNNVVLL---DSVLR----HAKC----------GGGVVAVQLDISKAFDTIPHCTIATALLNKGL  150
R2_Bg_ribo                     98  TEPGCYQNVRIF---SEVLR----QAKS----------ARGLVAVQLDISKAFDTVPHAAIATVLRQKGL  150
R2_RU                          98  NIQGCHVNLTIL---KEGIR----QAKV----------KNGGVIVSVDIEKAFDTIPHSVIFSRLASQGV  150
R2_RL                          98  NIQGCHVNLTIL---KEGIR----QAKV----------KNGGVIVSVDIEKAFDTIPHSVIFSRLASQGV  150
R2Dr                          101  RSPGCSENLEVL---ECLLR----HSKEK---------RSQLAVVFVDFAQAFDTVSHEHMLSVLEQMNV  154
R21_GA                        101  ASPGCSENLMLL---EGCMS----LSKAG---------NGSLAVVFVDFAKAFDTVSHEHLLSVLVQKGL  154
R21_SSa                       101  SSPGCSENLMIL---GGLIK----RSWAK---------GERLAVVLVDFARAFDSVSHSHILEILRQRGL  154
R2OlA                         102  SSSGCAENLLIF---DEIVR----RSRRD---------GGPLAVVFVDFARAFDSISHEHILCVLEEGGL  155
R21_Gav                       101  AAPGCAENLKVL---ELLLR----KRKRD---------RQPLGVVFVDLARAFDSVSHDHISWVLKAKGV  154
R21_ZA                        101  SAPGCSENLKLL---QSIIR----TAKNE---------HKPLGVIFVDIAKAFDTVSHQHIIHVLQQRRV  154
R21_TG                        101  RAAGCSENLKLL---QTIIW----SAKRE---------HRPLGVVFVDIAKAFDTVSHQHIIHALQQREV  154
R21_GFo                       101  SAAGCSENLKLL---QTIIR----TAKNE---------HRPLGVVFVDIAKAFDTVSHQHIIHVLQRRRV  154
R2NS1_CGi                      99  AASGCSENSFLL---SEMIN----HAKKE---------RRQLCVTFLDLAKAFDTVSHKHFIAGLRRFGS  152
NeSL1_TV                       99  RGATLSEHIAVA---NEVLC----QSTRT---------QSEMFQTAIDFTNAFGTVPHQLIFDSLEAKKV  152
Utopia1_ACa                   101  PAEGCHEHAFVL---RSVLD----DARRH---------KQNVYLAWYDLRNAFGSVSHDLIAWCAAMLGL  154
Utopia1_LV                    102  PCEGCFEHSFLL---SAALK----DCRRN---------QKTICIGWLDLRNAFGSIPHPVIKIVLSSLGV  155
Utopia1_CFl                   102  RDEGCFEHNFVL---QEILT----DARRT---------RRQAVVAWLDLSNAFGSVPHAAIRRALVRSGV  155
Utopia1_AEc                   102  RDEGCYEHNFVL---QEVLT----HAKRS---------KRQAVVAWLDLSNAFGSIPHATIRRALIRSAV  155
Utopia1_CMy                   102  SCEGCYEHNFVL---QTTIE----TARRA---------RRQCAVAWLDLANAFGSMPHHHIFATLQEFGM  155
Utopia1B_CPB                  102  SCEGCYEHNFLL---QTAIQ----EARRS---------KRQCAVAWLDLTNAFGSIPHHHIFATLGEFGM  155
Utopia1_ENe                   103  PVNGCHEHLFLA---QSILN----STRRS---------KKPLYMTYYDLQNAFGSISHQLIHVVLQAQRL  156
R52_SM                        105  VHEGCVEHNSIL---SSALE----HSKYS--------KNSPLAIAWLDIKDAFGSVPHDYLWSVLKTIGV  159
R5                            104  KFEGCHDHNAML---NMLIQ----DVRRQTNPSNPINKNKRLYIVFLDFTNAFGSVPLDTLMYVPQRFGL  166
R51_SM                        105  IPDGCAEHNTVI---SLAKE----WAVIN---------GKDINIVWLDLADAFGSVPHDLIWHSLSRLKL  158
LIN9_SM                       104  KANGCDEHSYVIQALIEKAN----RSYYK---------NEQCHLAFLDLADAFGSIPFQVIWHTLKNMGM  160
R4_Hmel                       103  KNSQGCKEQLTI----DAIV----MKQAH---------NKNXNTMYIDYRKAFDSVPHSWLLYILKKYKI  155
R42_AS                        103  KKEWGCTHAILL---DRAVA----TDATTQ--------KGRPLSIAWLDFKKAFDSLSHSYIRWILESVNV  158
R4_AL                         103  KREWGCTHAMVL---DRAMV----MDAMAQ--------KKHSLSVAWLDYRKAYDSVSHEYIRWAINSVNI  158
R45_BX                        105  KGVWGCTHASIV---DRLVTGTAAMSKGQ---------NADLRVLFYDCKKAFDSVSRDHMFRVLDVAGV  162
HERO1_BF                      104  GFPGCIEHASTI---WQQIQ----MARRQ---------KEDLHVVWLDLTNAYGAVPHSVIHYALNFFWV  157
HEROTn                        104  GVPGCLEHCGVV---TQLIR----EAREG---------RGSLAVLWLDLANAYGAIPHKLVEMALARHHV  157
HERO1_SP                      106  GHPGVWEHVATL---WEVIK----DAKTS---------QKNLVAIWLDLANAFGSVPHAAIAFALKWYHL  159
HERO1_HR                      105  TLDGLAEHTQML---SHLMK----DAKRY---------QRSIIIALLDLRNAFGEVNHQLINSILLYHHV  158
HERO3_BF                      104  GINGCVEHVQVM---REILA----HAKKN---------RRTVHITWFDLADAFGSVEHELIYYQMERNGF  157
R44_SRa                       101  QGYDGALDIGIT---NRILQ----EM------------GQFAQGWYDMQKAYDSVDHSRLKMIINSVKE  150
Consensus_aa:                      ...Ghhpp..ll...p.hlp......+.p..........p.h.lhhlDh.pAFsslsH.hl..hl....h
Consensus_ss:                          hhhhhhhh   hhhhh    hhhh              eeeeee   hh    hhhhhhhhhh

Conservation:                      4  311341114 2             113 31 11           2  2 213963895567636733
6ar1_chainD_p001              160  DKRVLKLIRAYLQA-------------GVM---------------IEGVKVQTEEGTPQGGPLSPLLANI  201
SLACS                         147  WSPLWRLVSLLLGT------------TGEVGFYENG---------KLCHTWESTRGVRQGMVLGPLLFSI  195
CRE1                          149  LKPIWGVVKVALGG------------PGFLGVYRDG---------CLKGNLWSTKGIRQGMVLGPLLYAT  197
CRE2                          151  LRFAFGVVNLSLGA------------AGELALYENG---------AKIHALKSTEGVRQGMVLSPLLFAN  199
GAU97528__Ramazzottius_       156  PTLVRNLILSLQGS-------------NRTQLQVGG---------QRSSAIPVKKGVFQGVPLSPMLYNM  203
Blag_1                        151  PFKLSSLILQLQRG-------------NKTQIETGR---------CRTKPIDIKRGIMQGSPLSPILYNI  198
Blag_5                        151  PSLLSNLILNLQEN-------------NHTRFEINH---------HKTNLVPLARGVMQGSPLSPTLYNL  198
Blag_2                        151  PAKLKDLIINLQIN-------------NTTNIQTSL---------GQTKAINLRRGVMQGSPLSPTLYNL  198
Blag_4                        152  PTSLCTLIKTLTRG-------------NFTRIHNGK---------QISEPIELKRGVFQGSPLSPILFNL  199
Blag_3                        151  PNNLRDLVLNLLQG-------------NKIQIESGF---------QKTKPIQVQKGVAQGSPLSPGLFNL  198
Blag_6                        151  PSQLKSLVLSLVKN-------------NSVGIELDA--------TTRTKPINIEKGVAQGSPLSPLLFNL  199
R2_PS                         147  PERLTGYVESIYRS-------------VHLTLGDD-------------WFVQGRGVLQGDPISPFLFNL  189
RaR2                          151  PYGFITFIRRMYTG-------------LQTMMQFEG----------HCKMTQVNRGVYQGDPLSGPIFLL  197
R8HmA                         149  PSGVVKVVDSLYAG-------------AVTSISVGK---------TKTRSICINSGVKQGCPLSPLLFNL  196
R8HmB                         149  PRSVIKVVDSLYAG-------------AVTSITVGK---------TKTRPICINSGVKQGCPLSPLLFNL  196
R24_MR                        150  DQHTVKYIMSTYKD-------------VDTTIECHG---------ERSSPIRMCRGVRQGDTDSPMLFNI  197
R21_MR                        150  DEHTICYIEANYRN-------------ITTTLECHG---------KRSRPINVDRGVKQGDPISGFLFNV  197
R2SmA                         150  PELLCRYLNNYYRR-------------STSCVN-------------RTELHPTCGVKQGDPLSPLLFIM  192
R2NS1_CSi                     150  PSPLVRYIAQSYEN-------------AVAVFP-------------SSEVHCHRGVRQGDPLSPLLFIM  192
R2CiB                         150  PPPLITYISNLYPN-------------CSTKLKVNG---------KISKPIEVRRGVRQGDPLSPLLFNA  197
R2NvecA                       150  PGPLLTYINELYSR-------------SETVFEVGG---------ESSGSVKVSQGVKQGDPLSSTLFNC  197
```

```
R21_MDe                      150  PKMFIDYVTDLYLN------------ARTVFQYNG---------SSTFLDIKRGVLQGDPLSPLLFNL  196
R2A_NVi                      149  PLEMRNYIMWVYRN------------SKTRLEVVK---------TKGRWIRPARGVRQGDPLSPLLFNC  196
R21_TCas                     150  PSVIREYILATFGA-------------QTTIKCGS---------VTTRPIRMLRGVRQGDPLSPVLFNL  196
R2LcA                        150  PINFINFVRNSYDG------------CTTKLTCGG--------TSFPDSVRMNRGVKQGDPLSPVLFNL  198
R2Ll                         150  PVNLXNFVRNSYDG------------CTTKLTCGG--------TSSPDSVRMNRGVKQXXPLSPVLFNL  198
R2La                         150  PENLVNYIQNSYSG------------CTTQISVGG--------SISASKIPMNRGVKQGDPLSPVLFNL  198
R2LcB                        150  PENLVNYIQNSYSG------------CTTQISVGG--------SISTTKILMNRGVKQGDPLSPVLFNL  198
R2NS1_SMed                   150  PKLLLGYIKESYNG------------ADTEVEK-------------IKATFKRGVKQGDPLSPVLFNN  192
R21_PPap                     150  PQNLVEYLKGLYAN------------NQTTLEYGG---------SELYCRVKRGVRQGDPLSPLLFNL  196
R22_TCas                     152  --------------------------SAETVLEEG---------GRRHFVQVRXGVRQXDPLSPLLFNL  185
R21_PBa                      152  PREFRDYVRAIYAE------------ARTVLQPTG--------RQRMSPIHVGRGVRQGDPLSPLLFNL  200
R28_MR                       149  PCELVSYVTTMYAR------------SSTMFEVRG---------QRSELFPVGRGVRQGDPLSPILFCL  196
R27_MR                       148  PAGFVQYISRTYSD------------SSTRLEVGR---------NRSEPIKTNRGVRQGDPLSTLIFCL  195
R21_IS                       150  SGEFIGYLKELYTT------------SRTLLQFQG---------ESLLVEPTTGVRQGDPLSPILFNL  196
R21_RMi                      150  SEDFISYIEDFYQN------------AVTVLTFGG---------KSLVVHPTVGVRQGDPLSPLLFNL  196
R2_FA                        150  PKPMINYIMACYRR------------SKTRFTFNG--------WISDTVKPTCGVKQGDPLSPILFNL  197
R25_MR                       150  PAPLVEYVRDVYER------------STTRLTGSD-----------WQSDLIRCGVKQGDPLPPIIFNM  195
R2_Dan                       150  PKGFINYLRKAYEG------------GGTMLAGNG---------WVSEAFIPARGVKQGDPLSPILFNL  197
R2_DSi                       150  PKGFVDYVQNTYEG------------GGTSLNGDG---------WSSEEFVPARGVKQGDPLSPILFNL  197
R2B_DM                       150  PKSFVDYVRRWYSG------------GGTYFNGGD---------WRSEEFVPARGVKQGDPLSPVLFNL  197
R2_DPe                       150  PKEFVDYVQNSYEV------------CGTTLNGDG---------WRSEEFIPARGVRQGDPLSPIIFNL  197
R2_BM                        150  PEQFCGYIAHLYDT------------ASTTLAVNN---------EMSSPVKVGRGVRQGDPLSPILFNV  197
R2C_NGi                      149  PTPFIEYFMTHLED------------SFTVLQHGN---------WQSGKIHPTCGVKQGDPLSPPIFNF  196
R2_AM                        150  PDSFVTYVRNCYDG------------FASVVKLG---------RDTAQTTVRQGVLQGDPLSPILFNL  196
R2CiD                        151  PEKFVRIVQDLYDG------------AVTTVSVGG---------RATPQLVVKRGVKQGCPLSPLLFNL  198
R21_BTe                      151  PALIVDLIDEMYKN------------VKTTIKTKD---------GGVEIMIRRGVKQGDPLSPLLFNL  197
R22_MR                       151  PKVVVQLIMDSYSN------------ISTQIQYQS---------STFDISIKRGVKQGDPLSPLIFNI  197
R2_KF                        152  DEHTMAHIRDMYSG------------IRTRINGKG-----------SDIPLVRGVKQGDPLSPMLFNM  196
R2B_NVi                      150  PETVAAYISSMYRD------------CSTAIRTRS---------GDVKIGMKRGVKQGDPLSPLIFNL  196
R2Amel                       151  PSPICEYIQKMYIG------------CKTIIYCRD---------KKTLPVDILRGVKQGDPLSPLLFNL  198
R2_Rm_ribo                   151  PPFLANLVSRAYVG------------QRTHLNVRG---------EKVEVRNPLVIVKQGDPLSPMLFNL  198
R2_Bg_ribo                   151  PPYFADMVRRSYEG------------AYTSIRVRD---------HRIQLELKRGVKQGDPISPLLFNM  197
R2_RU                        151  PPLLRKIISNMYKD------------VYTVIEGQ-------------CIPIKRGVKQGDPLFPLLFNI  193
R2_RL                        151  PPLLRKIISNMYKD------------VYTVIEGQ-------------CIPIKRGVKQGDPLSPLLFNI  193
R2Dr                         155  DPHMVNLIREIYTN------------SCTSVELGR---------KEGPDIPVRVGVKQGDPLSPLLFNL  202
R21_GA                       155  DQHMVELIKDSYEN------------SVTKVHCQE---------GCSTDIAMKVGVKQGDSMSPLLFNL  202
R21_SSa                      155  DEHIIGIVGDSYTD------------VTTTITVSG---------EQSPPIDMRVGVKQGDPMSPLLFNL  202
R2OlA                        156  DRHVIGLIRNSYVD------------CVTRVGCVE---------GMTPPIQMKVGVKQGDPMSPLLFNL  203
R21_Gav                      155  DEHIVNLIEDSYQK------------VTTRVQVFN---------GVTPPISIKTGVKQGDPMSPLLFNI  202
R21_ZA                       155  DPHIVGLVNNMYKD------------ISTYVTTKK--------NTHTDKIQIRVGVKQGDPLSPLLFNL  203
R21_TG                       155  DPHIVGLVSNMYEN------------ISTYITTKR--------NTHTDKIQIRVGVKQGDPMSPLLFNL  203
R21_GFo                      155  DPHIIGLVKNMYKD------------ISTVITTKK--------NTYTDKIQIQVGVKQGDPLSPLLFNL  203
R2NS1_CGi                    153  PEHFIDIVADLYNG------------ASTVFQTDA---------GQTGEIPMTRGVKQGDPLSPLLFNI  200
NeSL1_TV                     153  PDSIINLLKDLYKG------------ARTAIYTRH---------AHSEIVPVRRGVIQGCPLSPILFNC  200
Utopia1_ACa                  155  PRYLRDAIGAIYRH------------SALFVQVGD--------QETTGVIPMRCGVKQGCPLSPLLFNL  203
Utopia1_LV                   156  PDSLVTLLMDAYNG------------ASTSFTLTG---------GQTDTVPIRSGVKQGCPMSPILFNL  203
Utopia1_CFl                  156  PGGLVNVWNSMYDG------------CSTRVRTAE---------GYTTPVLIRSGVRQGCPLSPIVFDL  203
Utopia1_AEc                  156  PRGLIAIWDSMYDG------------CTTRVRTAE---------GHTAPIPIRSGVRQGCPLSPIIFNL  203
Utopia1_CMy                  156  PENFLRVIREVYEG------------CSTTIRSVE---------GETAEIPIRSGVKQGCPLSPIIFNL  203
Utopia1B_CPB                 156  PETFIQILRDLYKD------------CTTTIRATD---------GETDAIPIRRGVKQGCPLSPIIFNL  203
Utopia1_ENe                  157  PKHAQEVIMDLYQG------------ASFCILTKE---------GCTGRIVNQRGVKQGCPLSPILFNL  204
R52_SM                       160  SEEFITIVKLLYTD------------TQSFYSCGP---------IVTPNLSIKKGVKQGCPLSMILFSI  207
R5                           167  GTSALTLIKNLYLD------------NYTNVTCGE---------SKIENVKLNKGVKQGCPLSMLLFNI  214
R51_SM                       159  KNKTINLIKDMYND------------CYTVYECEG---------KHTKTIKVNNGVKQGCPMSMTLFSL  206
LIN9_SM                      161  DEETINLLKEIYKD------------CSTKYKCGK---------NESEKIKITKGVRQGCPLSMTLFSL  208
R4_Hmel                      156  HPILITFLSSVMLS------------WKTRLKLINNN----ETLITDWIKIQRGIFQGDALSPLWFCL  207
R42_AS                       159  PKTITATFFKLMAG------------WETRYELKYKTKRGCMSIQRSQPVKIVNGIFQGDALSPLLFIL  215
R4_AL                        159  PRSVQLTLKRLMSD------------WETRFESTQCR-----PKLRSDKMKVLNGIFQGDSLSPTLFVL  210
R45_BX                       163  DRKIYYLLKSFTMN------------WTVRYELRRSG-----RVERSAPLRVKRGLLQGDSLSPTWFCL  214
HERO1_BF                     158  PETIRTMIQNYFQD------------FRVSVTTPQ---------FTTGWQQLEKGIAMGCTISPLLFVL  205
HEROTn                       158  PCSIKTLIMDYYDS------------FHLRFVTSG--------SVTSEWHRLEKGIITGCTISVIIFAL  206
HERO1_SP                     160  PESLVSLMNNYYVG------------LYARFSVKD---------WNSEWQKFSVGIFMGCTLSPILFVI  207
HERO1_HR                     159  PQLFRNLILSIYSS------------YAISVSIND---------QLTPLLPVRKGVLQGDPCSPLLFNL  206
HERO3_BF                     158  PPIITTYIKNLYSR------------LKGKVKGPG---------WESDPFPFGRGVFQGDNLSPIIFLT  205
R44_SRa                      151  IPKPIRNLLCKLPKMYKFSVVNCSPNRDENGKIIKE---------KKKSWITMKRGIYQGCSLAPWLFCL  211
Consensus_aa:                     s..h..hl..hh.s..............h.h...............s..h.hppGl.QGssLSPhLFsl
Consensus_ss:                        hhhhhhh                 eeeeee                ee              hhhhhh

Conservation:                     244 32  411   1            21  2112275993242414  131 12113    2 1 14513
6ar1_chainD_p001             202  LLDDLDKELEKRG-----------------LKFCRYADDCNIYVKSLRAGQRVKQSIQRFLEKTLKLKV  253
SLACS                        196  GTLATLRRLQQTFP----------------EAQFTAYLDDVTVAAP-PEELKNVCAATAEAM-EALGIVN  247
CRE1                         198  GMAAAIGPVRQRIP----------------GVPVTAYIDDITLAASGAEGA-RAAEAYADAL-ETVGVVT  249
CRE2                         200  AMSGIIRPLMEMHP----------------RVKVVAYLDDVTLIGP-HAAVQDFLAEAGPQL-SRVGFDI  251
GAU97528__Ramazzottius_      204  STDYVTRNMTEAPIAEEYGFPL---CEGEQPVTFLGFADDSTVVGKSREEAIQLTEMAIGLF-KEIGLEV  269
Blag_1                       199  STDHILDELSNQELQDEYGFSV---SPGLSNITALGFADDTLIAAKSMNSARILVELAMSRF-KEIGLDI  264
Blag_5                       199  STDHILNELSEDGVAKAYGYQL---VPGLKPVTVLGFADDTVIVANSTTSALELTKMAIERF-RSIGLEI  264
Blag_2                       199  ATDFIFDELTELSLLQQYGKSI---SPQLSSLSIMGFADDTLLMGKNSETTLELLHIALQRF-QEIGLQV  264
Blag_4                       200  SIDFIIKELSEKQITDKYGFKI---SNNLDHLSVAAFADDLVVIGQNMHAARELVMMTKYLL-QQIGLQL  265
Blag_3                       199  ALDDTLKELADPNVREVFGFSL---HPDLEKVSGLAFADNIALISKDEASLQTLINMTVFNL-DAIGLKV  264
Blag_6                       200  SIDFIYRNLNDFQNASQYGYRL---TEEVDNLIALSFADDIALISNNETSAVFMIERVNQCL-TEIGLNL  265
R2_PS                        190  MIDYILSGTQAGVGVG----------VGERLVSSLAYADDLALLASSRRGLNANLESVLARA-RSVNLAL  248
RaR2                         198  AIEKGLQALDKEVGYD----------IGDVRVNAGAYADDTDLVAGTRLGLQDNINRFSSTI-KQVGLEV  256
R8HmA                        197  ILDELAERIEATGCGLD---------LDGHVLSSMAFADDYVLLAKDSVEMNELIRVCSTFF-KEKGLSV  256
R8HmB                        197  VIDELAERLEATGCGLD---------LEGHVISSMAFADDYVLLAKDSVEMNVLMNVCNTFF-EEKGLAV  256
R24_MR                       198  AIDDLVTSINTTDGVQ----------LGNSKIGCLLFADDIIFVSNTVHGINEHLRRLRAFL-EGTHMEV  256
R21_MR                       198  VLDGLLQKLHDQTGIQ----------INGTNIAALAFADDIVLMAPTPMIMRSHIRIVESYF-RRHGFEV  256
R2SmA                        193  VLDELLEGLDPMTHLT----------VDGESLNYIAYADDLVVFAPNAELLQRKLDRISLLL-HEAGWSI  251
R2NS1_CSi                    193  AMDEVLGLSMPQLGYQ----------FHDTLVDGFAFADDWVVCAESQARLKEKLEAAAVEL-GRAGMKI  251
R2CiB                        198  VMDWALSELDPRVGVQ----------IGEQRINHLAFADDIILVSSTKIGMVSSINTLSRHL-AKSGLEI  256
R2NvecA                      198  VIDWAVSDLDPHIGVL----------LGESRVSFLAYADDLVLLSETEAALTSQLNSIEKSL-AHCGLKL  256
```

```
R21_MDe                     197  VMDRAIVKLSANIGYT----------YNGAIINCIAYTDDIILIAETKCGLQSLIDALTSEL-ASFGLQT  255
R2A_NVi                     197  VMDAVLRRLPENTGFL----------MGAEKIGALVFADDLVLLAETREGLQASLSRIEAGL-QEQGLEM  255
R21_TCas                    197  VMDELLEKVNEKYEGGSL--------QSGERCAIMAFADDLILIADRDQDVPAMFDDVSTFL-ERRGMSV  257
R2LcA                       199  IIDSAIRKLPDSIGYVI---------RDGLKINCLAYADDLILVASSRAGLKTLLNIVAEHL-SLRGLDL  258
R2Ll                        199  IIDSAIRKLPDSIXYLI---------RDGLKINCLAYADDLILVASTRAGLKTLLNIVAEHL-SLRGLDL  258
R2La                        199  VINEIIRKLPASIGYPI---------NSELSINCIAYADDLILVTNTREGLKLLLGLLNEEL-PKRGLEL  258
R2LcB                       199  VINEIIRKLPASIGYPI---------NSELSINCIAYADDLILVANTREGLKLLLNLLNEEL-PKRGLEL  258
R2NS1_SMed                  193  IVELAIKKAYESGIGYR---------MNGTVFPIMAYADDIILLSESKEGLQDLLDIVLTEL-RHNGLEA  252
R21_PPap                    197  VMESALVRLDKKLSFS----------LYGVSVNGLAYADDVILVASTSGGLQKNTESFLGAL-REIGLDL  255
R22_TCas                    186  VLDRALKRLSTDVGFRL---------TDATKVTALAFADDVVLCATTARGLQTNLDVLEAEL-RLAGLLL  245
R21_PBa                     201  VVDRALGILSEDVGYR----------LESRLINALGYADDIVLLSSTKIGLQENLTRLHAAF-LQNGLTI  259
R28_MR                      197  VVDSILRTVPPDVGYD----------LGGSRLNALAYADDFLLFAASVWGMQKTLGLVEQRA-GEYGLTF  255
R27_MR                      196  CFDRVARTLSPHIGYD----------LNNTRISTLLYADDAFLVSTTAPGMNILLRSVEESA-GEVGLSF  254
R21_IS                      197  VLDEYLSSLDPDISFV----------SGDLRLDAMAFADDLIVFASTPAGLQDRLDALVEFF-DPRGLRV  255
R21_RMi                     197  VIDEFLAELDPQLAFT----------SEGMKVSAMAFADDIILTTATHWGLKQQIDRLNSFL-GARGLKI  255
R2_FA                       198  VMDRMIRKLPKEVGVN----------VGSKHYNGLTFADDLLLFATTPEGLQSSIDIVHLFL-LECGLLI  256
R25_MR                      196  IIDRLLKLIPPEIGVD----------VGESHFNALAFADDLLFMASTTKGLQTCLDLASEYL-LKCGLRV  254
R2_Dan                      198  VIDRLLRSLPSEIGAK----------VGNAMTNAAAFADDIVLFAETPMGLQKLLDTTVCFL-SSVGLTL  256
R2_DSi                      198  VMDRLLRNLPSEIGAK----------VGNAITNAAAFADDLVLFAETRMGLQVLLDKTLDFL-SLVGLKL  256
R2B_DM                      198  IIDRLLRSLPKDIGVH----------VGNAKVNACAFADDLMLFASTPKGLQELLNTTVKFL-SSVGLTL  256
R2_DPe                      198  IIDQLLRSYPNEIGAT----------IGDHTTNAAAFADDIVLFAETRLGLQTMLDTTVDFL-SSVGLTL  256
R2_BM                       198  VMDLILASLPERVGYR----------LEMELVSALAYADDLVLLAGSKVGMQESISAVDCVG-RQMGLRL  256
R2C_NGi                     197  IMDEMLKRLPKEIGVN----------LDGLFVNAMAFADDLSLVANTEQGLQILIDEATSFL-GLCGLRA  255
R2_AM                       197  VIDQIIRSLPETVGVQL---------DANTKLNSMAFADDLILLSSSEAGMRRMLGVLAGVS-SKFGLIF  256
R2CiD                       199  VLDELIDELSSSFGYTL---------DDGSKLSVMAFADDLVLSSGSLAGLRKLIETTQQFL-LRCGMGL  258
R21_BTe                     198  CLEPLLEEIEEQASGINV--------SEHRKVSVLAFADDIVLLGADAREAQHQINVLTDYL-QSLMMNL  258
R22_MR                      198  VMESLILKLESMPGFKI---------NDRTNLSSLAFADDLMFLADNVEDARAQLNLLCQFL-DSVDMDI  257
R2_KF                       197  VMDPLIRDLQRKGFR-----------IGGHEIGALAFADDIVLLADSIDGAQDHVDQVGRYM-NKLGMTL  254
R2B_NVi                     197  VLEPLLERLQETSGVE----------IEGMNLSCAAFADDIVCFANTAPEAGRQLRMVADYL-GRLDMSL  255
R2Amel                      199  IIDPIIGTLDETTEGIK---------LENENISVLAFADDLVLLAKDKETADKQNRLINEYL-DDLKMKV  258
R2_Rm_ribo                  199  IMEPMINELESKPGYRI---------DEETQISSLVFADDLILLASDAPKAQVLLSTVHRYL-CNLGMSL  258
R2_Bg_ribo                  198  IMEPLLNKLESLGGVTL---------ADGSQLACLAFADDLILLADNRERARELLETVEEFL-DGLGMKI  257
R2_RU                       194  AIDPVLRSLEEFQGGLP---------LGNSAIKILAFDDDIILGASSAGQAQQMVDMLGIGL-TSCGLGV  253
R2_RL                       194  AIDPVLRSLEEFQGGLP---------LGNSAIKILAFADDIILGASSAGQAQQMVDMLGIGL-TSCGLGV  253
R2Dr                        203  ALDPLIQSLERTGKGCE---------AEGHKVTALAFADDLALVAGSWEGMAHNLALVDEFC-LTTGLTV  262
R21_GA                      203  ALDPLIQQLEREGRGFP---------VNGKSITAMAFADDLAIVSDSWEGMRANLDILVDFC-ELTGMRT  262
R21_SSa                     203  ALDPMIDTLERYGLGYR---------MGEQQITALAFADDLVLVSDSWEGMACNIRILEEFC-RLTGLRI  262
R2OlA                       204  AMDPLIHKLETAGTGLK---------WGDLSIATLAFADDLVLVSDSEEGMGRSLGILEKFC-QLTGLRV  263
R21_Gav                     203  AMDPLIAKLETDGQGVK---------VGSASLTTLAFADDLVLLSDSWEGMLKNISILEDFC-NLTGLRV  262
R21_ZA                      204  AMDPLLCKLEESGKGFH---------RGQSSITAMAFADDLVLLSDSWENMKENIKILETFC-NLTGLKT  263
R21_TG                      204  AMDPLLCKLEESGKGYH---------RGQSSITAMAFADDLVLLSDSWENMNTNISILETFC-NLTGLKT  263
R21_GFo                     204  AMDPLLCKLEEHGKGFH---------RGQSKITAMAFADDLVLLSDSWEDMNANIKILETFC-DLTGLKT  263
R2NS1_CGi                   201  AMDPLLEVIGRQNNGY-KFGP-----EESDRIESLCYADDNALMTESPDEMNENLALVEKFC-CETGMRL  263
NeSL1_TV                    201  CLDPLLYAVQRRHFEDGYRFQD---KAGQYSIAIQAYADDVLVISPTHEGMQRILNTVDEFQ-KIAKLKV  266
Utopia1_ACa                 204  CVEPALRCLRRTTGYKFY--------GTSITVEGQAYADDLLTAAPSAYHAARQVATIEEWA-NWAGVSF  264
Utopia1_LV                  204  AIELIIRAVKKNASDNHLGVT-----VQGKNLSILAYADDLVLLSRDTEGLQSLLQVAGSSA-STLQMQF  267
Utopia1_CFl                 204  AIDSVLRAVTAVDAGFD---------LLGSRHSVVAYADDIALVADSPEGMRRLLAVAEGAA-SSVGLRF  263
Utopia1_AEc                 204  AIDSVVRVAAEXNDGYS---------LHGNTWSALAYADDIALLAQTPEGMERMLASVEAEA-ASVGLRF  263
Utopia1_CMy                 204  AMEPLLRAISNGTDGFN---------LHGERVSVLAYADDLVLTADDPESLQGMLDATSRAA-DWMGLRF  263
Utopia1B_CPB                204  AMEPLIRAISSGPTGFD---------LHGKKISILAYADDLALVADSSESLQQMLDVTSQAA-EWMGLRF  263
Utopia1_ENe                 205  AIEPLLQRLAACNEGLELRTTD---GKPQVKVSHMAYADDLKTVAATRVGISKLHQVVEDFL-QWTGLKA  270
R52_SM                      208  AINPVLEAISRSCIEPFM--------IGDSPVQVLAYADDIALVANNVENLQKIVDVAVEAA-TEIGFEY  268
R5                          215  FINIIIRAIEAMPDVHGYP-------LGDMDIRILAYADDIALISDSHKDLQEMVYKAEYIG-RILGLLF  276
R51_SM                      207  TINFILQNILKEYPLI----------IHNHNISIMAYADDLVLIADTREKMRKMIKDITKYT-DSATLKF  265
LIN9_SM                     209  CIQYLIQGIAEKKKGAT---------IAGQEVCILAYADDLVIVANTAKDMQMLLTTIENLA-KQADLIF  268
R4_Hmel                     208  ALNPLSELLNNTNTGFKLKHN-----NTYHIISHLMYMDDIKLYASNNKELKILADLTQSFS-TDIRMEF  271
R42_AS                      216  SVSPISFALENKAKPYRSSVGQ--LSGKGFSLGHQFYVDDLKIYAPNRRELVNALVVAKNIS-TAIGLDL  282
R4_AL                       211  CIAPISYALNKGVGQCQSSSGW--SAGYGFEIGHQFYMDDLKLYARTPAMLDSQIQVVSEVS-EAMGLHL  277
R45_BX                      215  CIAPISHALRLRNPGPCIRQVAGHSDGGQVHVSHLFYMDDLKVYCTGEAAQVRAERLVPELF-SQIGLCV  283
HERO1_BF                    206  GFEIFLIGARQVAGGIKLP-------SGQRLPPLRAIMDDVTSILRSAPCTRRVLQRLEELT-DWARMRF  267
HEROTn                      207  AMNMLAKSAEPECRGPITK-------SGIRQPPIRAFMDDLTVTTTSVPGCRWILQGLERLM-TWARMRF  268
HERO1_SP                    208  TFNLLNDFLKSTPAKQYHLKK-----RDFNVPVMKEYMDDITIITASVESAGSILSKVNDFM-DWSKMRI  271
HERO1_HR                    207  CFNPLMKILEGDEFKQCGYLWG--PTSDVRARSWLQFADDTAIIAHNSESAQKLIDLNLAWC-RWSDMQM  273
HERO3_BF                    206  VFQPILQHLKGVEQQHGYN-------LNDKHYVTLPFADDFCLITTNKRQHQKLITQISSNT-KSMNLKL  267
R44_SRa                     212  CLAPLQWKMKQIIDPIDL--------YGQKKIDILLFMDDIKGFGKNKNELDKITNLIINYG-KEIGLTI  272
Consensus_aa:                    hhp.hh..l.......................lshhh@hDDlhlht.s...hp..hp.h...h.p.hshph
Consensus_ss:                    hhhhhhhhhhh                     eeeee           hhhhhhhhhhhhhh hhh


Conservation:                    221          86122 2    1 1           11   1 2   112      42 21 12 13599
6ar1_chainD_p001            254  NEE---------KSAVDRPW--------------------------------------------KRAFLG  270
SLACS                       248  NAD---------KTEVLELT---GDTGF--------------------------GTAV---KRVREFLE  275
CRE1                        250  NAR---------KSMVVGPE---GTRVG--------------------IGG---VDLPV---VAEARILG  281
CRE2                        252  NPA---------KSHHLAKL---EVPEA----------------LSV--SGR---TIPIA---QGVVRILG  286
GAU97528__Ramazzottius_     270  SPT---------KSKAIVVE---NG------------VMSEVPLYL--SSG---AVIEATKKGEKVRYLG  310
Blag_1                      265  NTD---------KCRCISII---KG------------CVRYNPLNL--TSG---QTIASITPDENIKYLG  305
Blag_5                      265  NFS---------KSVAINIS---CGK-----------LNPNYSLLV--AEN---ATIPCLGPNEFVRYLG  306
Blag_2                      265  NPQ---------KCTGISIK---RG------------KLVEEVLHI--NEK---FTIKTINNQEEVRYLG  305
Blag_4                      266  NIA---------KSVSINIE---HG------------KLQDKELLL--YSG---YAIRGITKEEKIKYLG  306
Blag_3                      265  NPK---------KCMLINIK----------------NGFLEQGQIL--VGG---QTLEFIKPGEIIKYLG  304
Blag_6                      266  NAS---------KSKIINIK---KG------------KLMNTTVTT--LND---IKINSIQAEETIKYLG  306
R2_PS                       249  GIN---------KCATIGKR---WLGREKK-------MILDREPFL--LEG---VAIPVYRWNNIYKYLG  294
RaR2                        257  NPR---------KSMTLSLV---PSGKEKK------MKVETGKPFR--AND---VPLKELSINDFWRYLG  303
R8HmA                       257  NPG---------KCQSLRVL---PVKEKKRSM-KVLVRPHRWWRIK--DQD---VDIPSMTYDSLGKYLG  308
R8HmB                       257  NPA---------KCQSLRVL---PVK-GKRSM-KVLTRTHRWWKIN--NQD---VEIPSMTYESVGKYLG  307
R24_MR                      257  NPS---------KCRALQLA---RVPGTKR------VVVDTKPKFS--IDG---ASVPTLRVLEQLKYLG  303
R21_MR                      257  NVE---------KCATFQYI---PVPGTKR------LVVETKPLFK--ING---TDVPTLNVSSQLKYLG  303
R2SmA                       252  NPE---------KSRTLDLI---SGGHSK-------ITALSQTEFT--IAG---MRIPPLSAADTFDYLG  297
R2NS1_CSi                   252  NAR---------KTKAMVIC---GDRKHR-------ATAVSVEPFC--FAE---ELITPLGPTDTVTYLG  297
R2CiB                       257  SAG---------KEGKSASMAIVVDGKKKM------WTVDPLPRFK--VNS---QKIPALSITQQYKYLG  306
R2NvecA                     257  STG---------DSGKSASLNIVIDGKAKR------WVVNPTPFLR--ASG---GEIRSLVANETYKYLG  306
```

```
R21_MDe                  256  NIG---------KSSTISLV---PSGRQKK------IKVITEPSFL--VSG---QFLSAIGVMDVWRYLG  302
R2A_NVi                  256  MPR---------KCHTLALV---PSGKEKK------IKVETHKPFT--VGN---QEITQLGHADQWKYLG  302
R21_TCas                 258  NPA---------KCRALIAG---AVSGR--------SVVRTGSSYK--IHN---TPIPNVDALDAFKYLG  302
R2LcA                    259  NAA---------KCHGLSII---ASGKAKT------TYVSAADSLD--LDG---QPIKNLGVLDTWTYLG  305
R2Ll                     259  NAA---------KCHGLSII---ASGKAKT------TYVXALESLD--LDG---QPIKNLRVLDTWTYLG  305
R2La                     259  NAS---------KCFGLSLT---ALGKLKK------THLCTSDQLD--LHG---TLIKNLTAEESWVYLG  305
R2LcB                    259  NAS---------KCFGLSLT---ALGKLKK------THLCTSDQLD--LHG---TLIKNLTAEESWVYLG  305
R2NS1_SMed               253  NAD---------KCASIIAK---KDGKQK-------KWVISSDSVL--AAG---KTMPCMNANSMYKYLG  298
R21_PPap                 256  NLA---------KCKSLSLK---PSGRDKR------CKVLSESQLS--IGG---TSVPQVDLVGFWRYLG  302
R22_TCas                 246  NPN---------KCQALSLV---ASGRDHK------VKLVTKPTFK--VGQ---NTIHQVDASSIWKYLG  292
R21_PBa                  260  NAN---------KTGVLSMV---ASGRDKK------VKIDMTPYFT--VGG---ALIPQRSPVDVWTYLG  306
R28_MR                   256  NAQ---------KCSVLSLV---PSGKDKK------MKVITTPLFH-LGRQ---AFIPQITTTAEWRYLG  303
R27_MR                   255  NTS---------KCSALSLI---PSGKEKK------MKVGTTPTFK--TSQ---GFITQITPSQEWRYLG  301
R21_IS                   256  NVK---------KSFTLSLQ---PGRDKK-------VKVVCDQIFT--IGG---TPLPASKVATPWRYLG  301
R21_RMi                  256  NAA---------KSTTLVIE---PSGWQKR------SKIRTDIDFF--VNG---ERLATTNCTSTWRYLG  302
R2_FA                    257  NKQ---------KSFVLTVK---AYPKLKK------TAVIVTEKYM--LDR---HILPAIDREKLFHYLG  303
R25_MR                   255  NAX---------KSFTVALR---NVPHVKK------SXVDGKVRFR--CLG---HEMPSLTREDQWRYLG  301
R2_Dan                   257  NTD---------KCFTVSIK---GQAKQK-------CTVVERRSFL--IGG---RECPSLKRTDEWKYLG  302
R2_DSi                   257  NAD---------KCFTVGIK---GQPKQK-------CTVLEAQSFY--VGS---REIPSLKRTDEWKYLG  302
R2B_DM                   257  NAD---------KCFTISIK---GQPKQK-------VTVVEQRTFC--IG----RARVQLKRSEEWKYLG  301
R2_DPe                   257  NSD---------KCFTVGIK---GQPKQK-------CTVVIPETFR--IGS---RSCPALKRTDEWKYLG  302
R2_BM                    257  NCR---------KSAVLSMI---PDGHRKK------HHYLTERTFN--IGG---KPLRQVSCVERWRYLG  303
R2C_NGi                  256  NPN---------KCVTLAIK---TIPKEKK------TAIDPSSHFR--IGN---AVIPSLKRTDEWVYLG  302
R2_AM                    257  HPG---------KCKYLAMI---WAGKQKK------MKIATDLSFE--IGG---GFMTPVGVTETWKYLG  303
R2CiD                    259  NVQ---------NCTSLGWK---SMVGGKQ------VRLRTEPFVR--VGD---SELACLGPNSTCRYLG  305
R21_BTe                  259  SIE---------KCQTFEVV---AKKD---------TWFIKEPGLK--IGN---QIMPTVDPDEAFKYLG  302
R22_MR                   258  SID---------KCAAFQII---TSKD---------SWYIIDPHLT--VAQ---GSIAFLGPDTPLSYLG  301
R2_KF                    255  NPR---------KSSSFLIT---AMRK---------TWICRDPGLS--IGE---TKVPGARPSSALKYLG  298
R2B_NVi                  256  SVS---------KCIAVEYV---PHRK---------TWYTKNPGLE--VNG---NAVPSISPSETFKYLG  299
R2Amel                   259  SAE---------KCTTFEIK---RQNK---------TWFLGDPQLT--LGQ---QRIPYADPEAAIKYLG  302
R2_Rm_ribo               259  AAS---------KSFAFQVT---RTQDSWCLVDPGLKLSKGDEERC--HNL---EGIPFAGPVDRLRYLG  311
R2_Bg_ribo               258  WAP---------KSVAFEIV---PTKDS--------WCVVDPGLRL--RQG---GAITYAGPDHSLKYLG  302
R2_RU                    254  SHR---------KCFGFQIV---NKNK---------TWAIVDPMIT--LNG---SSLPFSGPEDRLPYLG  297
R2_RL                    254  SHR---------KCFGFQIV---NKNK---------TWTIVDPMIT--LNG---SSLPFSGPEDRLPYLG  297
R2Dr                     263  QPK---------KCHSFMVR---PCRGA--------FTVNDCPPWV--LGG---KALQLTNIENSIKYLG  307
R21_GA                   263  QPS---------KCHGFLIE---KSGSRS-------YKVNRCEPWL--LND---TALHMVGPKESIKYLG  308
R21_SSa                  263  QPR---------KCHGFLIQ---KIQRA--------RSVNLCKPWI--VCG---EELHMVGPEESVSYLG  307
R2OlA                    264  QPR---------KCHGFFMD---KG------------VVNGCGTWE--ICG---SPIHMIPPGESVRYLG  304
R21_Gav                  263  QPK---------KCQGFFLN---PTCDS--------FTVNNCEAWK--IAG---REITMLGPGESTRYLG  307
R21_ZA                   264  QGQ---------KCHGFYIK---PTKDS--------YTINNCPAWT--ING---TPLNMINPGESEKYLG  308
R21_TG                   264  QGQ---------KCHGFYIK---PTKDS--------YTINDCAAWT--ING---TPLNMIDPGESEKYLG  308
R21_GFo                  264  QGQ---------KCHGFYIK---PTKDS--------YTVNNCAAWT--ING---TPLNMINPGESEKYLG  308
R2NS1_CGi                264  NIK---------KSATFCIT---PCGSRSY------TVNTTKTKVA--IKG---ERVPVIPPDGCMKYLG  310
NeSL1_TV                 267  APQ---------KCVTLAKT---STA---------------IQPFR--IGP---DEIPIKTSMDNITYLG  304
Utopia1_ACa              265  VVQALSLDAPAGKCAALAIN---FEGG---------LMHSIDPALK--VQG---AAIPAMSRNNVYRYLG  317
Utopia1_LV               268  KPQ---------KCATLTLD---CKR----------GTNVRQSAHH--IQG---AAIPSLTEEERYRYLG  310
Utopia1_CFl              264  NPA---------KCATLHLA---AGS----------GGRVLPTTFV--IQG---QPVHHLSSGEPYRHLG  306
Utopia1_AEc              264  NPA---------KCATLHVG---AGN----------GGRVLPTSFQ--IQG---ETINPLAQGESYTHLG  306
Utopia1_CMy              264  NAK---------KCATLHID---GSK----------RDSVQTTGFQ--IQG---EPVIPLAEGQAYQHLG  306
Utopia1B_CPB             264  NPK---------KCASLHVD---GGA----------RALVRPSRFL--IQG---EPMASLEEGEVYQHLG  306
Utopia1_ENe              271  NPS---------KCATLGLK---VQKA---------KQVPDPVKLT--LHY---EVLPVVKLGEAYKYLG  314
R52_SM                   269  RPE---------KCGYMQLP---RVN--------------INGEIL--INE---KEIKKLLSKEFYQYLG  307
R5                       277  NPS---------KCALMDIP---HDK--------------KRTPPIL--VNG---EMIKCVGKADPYKYLG  316
R51_SM                   266  RPS---------KCGYFQLK---RNH--------------NDPPIT--LYD---EQIPIIDENHLYKYLG  304
LIN9_SM                  269  KPA---------KCGYYRDP---RDK---------------KSMMK--IYG---KEISIVDEKNVYTYLG  306
R4_Hmel                  272  GIE---------KCKVHSIK---RGKS-------------QQNTYIL--NTG---EQIESMDENSTYKYLG  312
R42_AS                   283  NVE---------KCAQAHYT---PNVQ-------------QLEVETLADEEP--LMELPVLGLRQSYRYLG  326
R4_AL                    278  NLS---------KCAKAHYA---PHGA-------------GGAQEAVEGAEGSRKGEIPILGLRSTYKYLG  323
R45_BX                   284  NES---------KSAAAAGP---SR-----------------------TLR---SSLPLLGVKDQYKYLG  315
HERO1_BF                 268  KPS---------KSRSLSLR---KGK-------------SSNRVFS--INN---QDIPTIQ-QEPVKSLG  306
HEROTn                   269  KPG---------KSRSLVLK---AGK------------VTDRFRFY--LGG---TQIPSVS-EKPVKSLG  308
HERO1_SP                 272  KPV---------KSRSLVVH---GGK------------VKHIEPFA--VDG---QTIPGVH-NKPVKFLG  311
HERO1_HR                 274  RVD---------KCCVFGMM---KKDG---------SYDQFLPRLT--IDN---IPIPPVELGSGFKYLG  317
HERO3_BF                 268  KPR---------KCKSMSIV---SGK-------------PSDISFT--IDG---DPVKTT-KDAPEKFLG  306
R44_SRa                  273  NKD---------KTEYS--------------------------------------IPDEEKIIQYKYMG  294
Consensus_aa:                 p...........Kt.sh.h...................h.....h...h.s.....l..h...p.hcYLG
Consensus_ss:                               eeeee                          ee  e                eeeee


Conservation:                 2 22  11           1   2      3114 131 7221452 342 24252314 311
6ar1_chainD_p001         271  FSFTPERKARI---RLAPRSIQRL----KQRIRQLTNPN-ISMPERIHRVNQYVMGWIGYFRL-------  325
SLACS                    276  RTWP--DPMS----EEIREGVEKKAMETDRLFKAIVELP-LYNRTRWRILAMSAMPRITFLLRNH-----  333
CRE1                     282  AHFRARGTPEARTIEWLQAAVEKW----RPIHQKLRQDI-IPKNIAMMMTRISLGSKMTFLLQTH-----  341
CRE2                     287  AGFRGDTASVE---EWVWEKTKTH----DHYFEKLQSEW-LPRLARLQLLRGSTVPRLNHLLRTH-----  343
GAU97528__Ramazzottius_  311  ATVTDQLDF------DQGEVIKQL----TDQVDRLVHFAHLHADQKLNLLNQWLLPSIIYPLQTA----P  366
Blag_1                   306  VSYNATIL-------DAANSIEEL----KNKLDRLASTPLLQPHQKYTVLCSFICPTLIYKFQTT----P  360
Blag_5                   307  VNFADACLF------DSQVTLNDL----KNKLDLLISSPLLNPDQKFCILNSSICPSLIYTFQTT----P  362
Blag_2                   306  APFQDNALF------DKDSTIKQL----HKKLELLASSPLLKSDQKFLVLNTAICPTLIYRFQTT----D  361
Blag_4                   307  VSFNDEIIF------DRESFITSL----RNDLQILVSTPMLRPDQKLNIVNQYIWPKFIYALQMV----P  362
Blag_3                   305  VTFEDEIVL------DKSTLIKNF----ENDLNSLTVSNLLTPDQKLNIVNQYIWSMLIYPLQCT----P  360
Blag_6                   307  VTYNDEIILD-----KCSIITNLN----RSISKLITSSL-LKPDQKLNILNQYIWPTLIYPLQCA----P  362
R2_PS                    295  IETSPGAA--------ARWSVTGL----RNRLSKLESAC-LKPQQRMHLLRCYLLPGLYYGLIH------  345
RaR2                     304  ISYTNEG---------PERLSLTI----EQDLERLTKAP-LKPQQRIHMLNAYVIPKYQDKLVL------  353
R8HmA                    309  VSIDPTGK--------IALPIEEW----KNWMTKLKECK-LKPEQKVKILKEVVCSRVNYVLRM------  359
R8HmB                    308  VMIDPAGK--------IALPIEEW----KLWLTRLRECK-LKPDQKVKVLKEVVCARANYVLRM------  358
R24_MR                   304  HGYSQSGM--------LAPSPSNL----ESMLERLRRAA-LRPWQKLYILNRYLIPRLIHCCQS------  354
R21_MR                   304  LQYGFRGA--------TCPTPSKT----EEMLSRLGKSP-LKPWQKLNVLQRYLIPRLHHGMQM------  354
R2SmA                    298  IKSNFKGR-------CPVAHIDLL----NNYLTEISCAP-LKPQQRMKILKDNLLPRLLYPLTL------  349
R2NS1_CSi                298  IPFTFKGK-------GVFNHRQHL----LKLLDEVTRAP-LKPHQRMEITRNYLIPRLTYSLVL------  349
R2CiB                    307  INIDAQGA--------RNDAARIL----TEGLAELSRAP-LKPQQRLYLLRVHLLPKLQHGLVL------  357
R2NvecA                  307  INIGAQGV--------KAAEYNAF----KEALDNLSRAP-LKPQQRLFLLKTYLLPQLHHSLVL------  357
```

```
R21_MDe                     303  VDFRCNV---------LNGSEYGI----DANLRKLDRAP-LKPQQRLKMLCVAIIPRYLHSLVL------  352
R2A_NVi                     303  VVYNSYG---------PIQVKINI----AGDLQRVTAAP-LKPQQRMAILGMFLIPRFIHKLVL------  352
R21_TCas                    303  LEFGHKGV--------ERPTIHNL----SVWLNNLRRAP-LKPDQKCLFIRQYVIPRLLYGMQN------  353
R2LcA                       306  IPFSHLGR--------AEKVSPDL----TNLLNKLQKAP-LKLQQKLYAVRNFVIPRALHGLIL------  356
R2Ll                        306  IPFSHLGR--------AXKFSPDL----SNLLNKLQKAP-LKIQQKLYAVRNFIIPRALHGLIL------  356
R2La                        306  VPFSHIGR--------SKSFSPDL----EALLNKLQKSP-LKLQQKLFALRVYLIPRLLHGLVL------  356
R2LcB                       306  VPFSHIGR--------SKSFSPDL----EALLNKLQKSP-LKLQQKLFALRVYLIPRLLHGLVL------  356
R2NS1_SMed                  299  IYFGPLGI-------TTERLTKIY----SDQLYNIGIAP-LKPQQRLEMINDFLLPGILHEAVL------  350
R21_PPap                    303  IWFSGPRV--------VSPEQLSM----GVYLERISKAP-LKPQQRIRILVDYLLPKYTHGSVL------  353
R22_TCas                    293  IQFRGSGM--------CGCGSEGV----AAGLKRITCAP-LKPQQRMHLLRVFFLPKFYHAWTF------  343
R21_PBa                     307  GMYQGAR---------EYASVPPL----ANSIRHITKAP-LKPQQRLRLLRDCLLPRYYHRWII------  356
R28_MR                      304  VDFRSTG---------PKKSRIDL----TTYLDRIGKAP-LKPQQRLKLLRCFLLPRLYHVLVL------  353
R27_MR                      302  VDFQYSG---------PKKASRSL----KIELERISKAP-LKPQQRLLILRVYLLPRYYHHLVL------  351
R21_IS                      302  MTFTPQGS--------INKGTSEQL----DLLLTRTSKAP-LKPQQRLVVLRNYLLPRLYHRLVL------  353
R21_RMi                     303  VHFGVKGL-------EKGLVRRQL----AILLERVSKAP-LKPQQRLVVLRFYLLPRLYHRLVL------  354
R2_FA                       304  VPFTAEGR-------CRDDTIAHL----KRKIDVLTKAP-LKPQQRLFALRVVILPSCYHILTL------  355
R25_MR                      302  VPFTPEGR-------SSARADEQL----KQTLGKLTSAP-LKPQQKLFALRVVVLXGLHHLLSL------  353
R2_Dan                      303  IKFTAEGR-------ARYDPAEDL----GPKLLRLTRAP-LKPQQKLFALRTVLIPQLYHKLTL------  354
R2_DSi                      303  INFTATGR-------VRCNPAEDI----GPKLQRLTKAP-LKPQQRMFALRTVLIPQLYHKLAL------  354
R2B_DM                      302  IHFTADGR-------ARYNPSEDI----GPKLERLMQSP-LKPQQKLFALRTVLVPQLYHKLTL------  353
R2_DPe                      303  ITFTAQGR-------TRYSPADDL----GPKLLRLTRSP-LKPQQKLFALRTVLIPQLYHKLTL------  354
R2_BM                       304  VDFEASG---------CVTLEHSI----SSALNNISRAP-LKPQQRLEILRAHLIPRFQHGFVL------  353
R2C_NGi                     303  IKFNSNGR-------LISDAKPKL----IKDLELLTKAP-LKPQQRLWALKVIVIPGILYRGTL------  354
R2_AM                       304  AYLGQIG---------IQPARLSL----QTFLERIAKSP-LKPQQKLYLIRVHLLPKLIYPLVM------  353
R2CiD                       306  IHFGMVGS--------CRIRRREI----RESLLRLASSA-LKPEQKVFMLRSFLIPRWRYSVTL------  356
R21_BTe                     303  AKIGPWKGV------HCGVIVPEL----LSVVKRVRKLS-LKPGQKVELLTKYIFPRYIYHLLV------  355
R22_MR                      302  AKISPWVGV------KTDYSIQGL----KACVGRVQSLA-LKPHQKVELMARHIIPHYLYGLIL------  354
R2_KF                       299  VNYTLSEGL------ESGALIDKL----MQAVNRARGLA-LKPLQKVNLILERIIPKFLYGIIL------  351
R2B_NVi                     300  AKVSPWKGL------LEGFESDAF----REVISRVQRLP-LKPMQKVDLLQMYIFPRYTYGLIT------  352
R2Amel                      303  TNFNPWRG-------LCKTSIKEI----IDAARTVKQLK-LKPHQKINLIRTYLLPRYIHKLVA------  354
R2_Rm_ribo                  312  VSVSPWFGI------DIRETRANL----TEVLHKLCRLA-LKPWQKHHLLITHLVPHYLPELVA------  364
R2_Bg_ribo                  303  VKVSPWVGV------DVSDLRTHF----SKTLEDTRKLP-LKPHQKLHLLKTYLIPHFTHRLVS------  355
R2_RU                       298  VDTNPWDRK------SRYDAGQRL----ISAAKRGSQLS-LKPQQKINLITTFLLPKFLYILIE------  350
R2_RL                       298  VDINPWDRK------SRYDAGQRL----ISAAKRGSQLS-LKPQQKINLITAFLLPKFLYILIE------  350
R2Dr                        308  VKVNPWAGI------EKPDLTVAL----DRWCKRIGKSL-LKPSQKVYILNQFAIPRLFYLADH------  360
R21_GA                      309  VQVNPWTGI------FAEDTVAKL----RQWVVAISKTP-LRPLDKVSLLCQFAVPRVIFVADH------  361
R21_SSa                     308  MKVSPWHGI------MEPDPVERL----CNWISSIGRSP-LKPSQKVRMLNVYAAPRMTYQADH------  360
R2OlA                       305  VQVGPGRGV------MEPDLIPTV----HTWIERITEAP-LKPSQRMRVLNSFALPRIIYQADL------  357
R21_Gav                     308  LNVGPWVGI------DKPDLGTQL----SSWLERIGTAP-LKPMQKLSLLVQYAIPRLNYQADY------  360
R21_ZA                      309  LQIDPWTGV------AKYDLSTKL----KIWLESIDRAP-LKPLQKLDILKTYTIPRLTYLADH------  361
R21_TG                      309  LQFDPWIGI------ARSGLSTKL----DFWLQRIDQAP-LKPLQKTDILKTYTIPRLIYIADH------  361
R21_GFo                     309  LQFDPWVGI------AKTSLPEKL----DFWLERIDRAP-LKPFQKLDILKTYTIPRLTYVADH------  361
R2NS1_CGi                   311  SKMSPWVTK------IRKDIVAQL----EGMVESIGGAH-LKPRQKLVLLNHYALVRLTYALTQ------  363
NeSL1_TV                    305  IPISGTKTSRF---AAATGILEKV----KAQIRVVFASH-LALSQKIIALRVFILPQLDFYMFH------  360
Utopia1_ACa                 318  VHVGLTDALG-----QANELLEKA----SRDARTICASG-LEPWQKVVAIKTFILSRLPFFFHN------  371
Utopia1_LV                  311  VPIGLPRLT------SLQESSRKL----SSDIETISSSL-LAPWQKLDAIKTFVIPILQYTLRA------  363
Utopia1_CFl                 307  VPTGSSVDQ------TPFATIGGL----LEDLRAVDQSL-LAPWQKLEVVATNILPRLDFLLRG------  359
Utopia1_AEc                 307  VPTGFSVDQ------TPYAAVGDI----VSDLRAVDRSL-LAPWQKIEMLGTFILSRLDFLLRG------  359
Utopia1_CMy                 307  TPTGFRVRQ------TPEDTIQEI----LQDAAKIDASL-LAPWQKINALNTFLIPRISFVLRG------  359
Utopia1B_CPB                307  TPTGVRVRQ------TPEDTIAEI----LRDAAQIDSSL-LAPWQKINALNTFLIPRISFVLRG------  359
Utopia1_ENe                 315  IKDALESPVQQ---SQILRAMSRI----KKDLNKLLRSE-LLPWQKLDALRTFVMSRLDYYLRH------  370
R52_SM                      308  VPVGEDNDQ------SPYAILDKV----VSDTKKIADSG-LFGWQKLKAYKIFIHSRLTFAFRT------  360
R5                          317  TFRSWFRKL------DIKELLQMM----MDETKLITESN-LHPHQKIHAYETFIHSQLPFHLRH------  369
R51_SM                      305  VDFGQKGKH------NIDTILNTA----LDDTTKILSSD-LHPAQKLQAYKTFIHSRLIFPFRN------  357
LIN9_SM                     307  VRIGDTKKK------DLNVRFEEV----KKKTTAIFKSK-LRSDQKLEAYNIFCQSKFVYILQG------  359
R4_Hmel                     313  FQQAKQIQQK----QTKIELTNKF----KFRLNQILRSQ-LNSRNIIKAINTYAIPILTYSFAI------  367
R42_AS                      327  IDQKFLATKN-----ALQRFEESL----FNRASAIFRSD-LTWRQMSNAFQSIALGGLRYGFLN------  380
R4_AL                       324  VEQRLLPMEV----ALKEFEDKF----MDRAETIFASE-LTWGQMATAYNTIAIAGLRYVYSN------  377
R45_BX                      316  IESGFVANEA----SAYSRMQTTI----LAKVQSILDVEGHTVGQRRIAIRTVAFPGAAFILGNIILGDK  377
HERO1_BF                    307  RLYTSDIADT----KRGQELVKQA----VEGLRAIDKCE-LPGKLKVWCLQSVLIPRLKWPMKM------  361
HEROTn                      309  KMFDGSLKYA----FSIRETNDQL----GHWLTLVDKSG-LPGKFKAWVYQHGILPRILWPLLV------  363
HERO1_SP                    312  RLIDGNLKDN----VARQDMMVSL----GRWLHTIDKCV-LTGVMKCWIYQYLIVPRMQWKLMI------  366
HERO1_HR                    318  KIYDAELKDT----KAKEEMVSKL----TRLLKRTTDLK-VSTQMKLQVLRRFIPSQLKFELRS------  372
HERO3_BF                    307  GYITFLSKTK----ETYDILAKTI----ETTVENINKSA-IRNEYKLRVYMEYAFPSWRYMLMV------  361
R44_SRa                     295  VYENNKTACDE---INAEQLLMKL----DEKMDKIFNSK-LNVKNIIKAINGCVIPTCTYLFSH------  350
Consensus_aa:                    h.hs.................ph.......hp.l.ps.Lps.p+h.hlp.hhhsph.@.h........
Consensus_ss:                    eee                ehhhhhhh    hhhhhhhhh     hhhhhhhhhhhhhhhhhhhhhh

Conservation:                      11 1 31                141 421             3312213
6ar1_chainD_p001            326  --VETPSVLQT------------IEGWIRRR----------LRLCQWLQWKRVRTRIRELRALGLKETA  370
SLACS                       334  DMQHTHRVASW------------FDERTTQV----------MEHILGQ--------------------  359
CRE1                        342  SPQELETAAKT------------ADDEVEQT----------LQHLMGQ--------------------  367
CRE2                        344  KPEELNRSTTW------------FDERVMET----------ALNIANI--------------------  369
GAU97528__Ramazzottius_     367  TNTILKVFLQT------------VDKIVKST----------VREILQL--------------------  392
Blag_1                      361  LKKLPAKFLSD------------ADVLIKTT----------LKEVLQL--------------------  386
Blag_5                      363  LNKIPNKFIEA------------ADKIIKSG----------LKEILQL--------------------  388
Blag_2                      362  LQTIPLQFLHT------------ADKLVRST----------LKDILQL--------------------  387
Blag_4                      363  VHKLPLTFLED------------VDKLIRSA----------VKEMLLL--------------------  388
Blag_3                      361  LDKIPSSFLQD------------IDKLLRSS----------IKEIIGL--------------------  386
Blag_6                      363  LCKLQLGFLQD------------IDKIVKNA----------VKEIVGL--------------------  388
R2_PS                       346  -QGISVGLLRS------------ADKQVRAA----------ARKILHL--------------------  370
RaR2                        354  -SKTTAKGLKR------------TDRQIRQY----------VRRWLKL--------------------  378
R8HmA                       360  -SECGISELRS------------WTRFVRNW----------AKNIIHL--------------------  384
R8HmB                       359  -SGCGICELRK------------WSRFVRGW----------VKSIIHF--------------------  383
R24_MR                      355  -SSITAGRLEY------------MDRLIRKF----------IKKTLHL--------------------  379
R21_MR                      355  -MDVNKKKLTY------------LDGCIVKF----------VKETLHL--------------------  379
R2SmA                       350  -GIVHLKTLKS------------MDRNIHTA----------IRKWLRL--------------------  374
R2NS1_CSi                   350  -GQVHRNTLKR------------LDNYIRQS----------IRGWLRL--------------------  374
R2CiB                       358  -SSCAKRALTY------------LDKSVRSA----------IRRWLTL--------------------  382
R2NvecA                     358  -SRTTGKLLNS------------LDALVRKA----------VRGWLKL--------------------  382
```

```
R21_MDe           353  -GRVNKTKLAG-------------FDNLIRKY----------VRKWLYL---------------------  377
R2A_NVi           353  -GRTSNADVRK-------------GDKIIRKT----------VRGWLRL---------------------  377
R21_TCas          354  -PQVTSRVLRE-------------ADRLIRRH----------LKTYYHL---------------------  378
R2LcA             357  -SKTNLKELNT-------------LDRAIRVF----------LRTLLYL---------------------  381
R2Ll              357  -SRTNLKELNT-------------LDRAIRVF----------IRTLLHL---------------------  381
R2La              357  -SRVAIGELKI-------------MDKLILKH----------LRVWLRL---------------------  381
R2LcB             357  -SRVAIGELKI-------------MDKLILKH----------LRVWLRL---------------------  381
R2NS1_SMed        351  -GNTNVGELKD-------------LDKMTRVA----------IRKWLHL---------------------  375
R21_PPap          354  -GRYTRKTYKA-------------MDAQIRSY----------VRKWLHL---------------------  378
R22_TCas          344  -GRLNAGVLRR-------------LDVVVRTS----------VRTWLRL---------------------  368
R21_PBa           357  -GTITSKILKE-------------MDVLLRAA----------VRRWLRL---------------------  381
R28_MR            354  -GRTTLGALRA-------------LDVLTRAA----------VRKWLRL---------------------  378
R27_MR            352  -SRTTLGHLRG-------------LDLQVRAA----------VRRWLSL---------------------  376
R21_IS            354  -GPWSAALLLK-------------MDTTIRGA----------IRRWMDL---------------------  378
R21_RMi           355  -GPISAKTLLT-------------IDRVVRSA----------VRRWLAL---------------------  379
R2_FA             356  -GGSNLSLLKK-------------IDLMVRAA----------GRKWCCL---------------------  380
R25_MR            354  -GNVTLSLLRK-------------VDKLVRAA----------VRKWLDL---------------------  378
R2_Dan            355  -GSVTIGVLKK-------------FDKLVRYT----------ARKWLGL---------------------  379
R2_DSi            355  -GSVAIGVLRK-------------TDKLIRYY----------VRRWLNL---------------------  379
R2B_DM            354  -GSVALGVLRK-------------CDKLVRSF----------ARKLLGL---------------------  378
R2_DPe            355  -GSVMIGVLRK-------------CDILVRST----------VRKWLGL---------------------  379
R2_BM             354  -GNISDDRLRM-------------LDVQIRKA----------VGQWLRL---------------------  378
R2C_NGi           355  -GSSTAGYLRS-------------LDCVIRAY----------VRRWLRL---------------------  379
R2_AM             354  -APIRASMLNK-------------LDRMVRVA----------LTGKDGI---------------------  378
R2CiD             357  -TRVTVSFLSW-------------IDRVVRMR----------VRAILHA---------------------  381
R21_BTe           356  -SPPSDTVLKL-------------LDSEVRQE----------VKTILHL---------------------  380
R22_MR            355  -SIPSISVIRQ-------------LDQCVRVA----------VKEYLHL---------------------  379
R2_KF             352  -GGPSLTRLHA-------------ADKCVRMA----------VKEILHL---------------------  376
R2B_NVi           353  -SPPAKAVLKT-------------IDRIIRTR----------IKEILHL---------------------  377
R2Amel            355  -NPPPLGTLDL-------------IDKELKTI----------IKEILHL---------------------  379
R2_Rm_ribo        365  -AAPALTTLRE-------------LDQELRVV----------VKKIFHL---------------------  389
R2_Bg_ribo        356  -SLPSIAELRA-------------LDLEIRGC----------VKQILHL---------------------  380
R2_RU             351  -DPPSPAYLKS-------------IDHDLRQI----------YKNILHL---------------------  375
R2_RL             351  -DPPSPAYLKS-------------IDHDLRQI----------YKNILHL---------------------  375
R2Dr              361  -GGAGDVMLQN-------------LDGTIRKA----------VKKWLHL---------------------  385
R21_GA            362  -CMLSAKALTE-------------MDRSIRQA----------VKRWLHL---------------------  386
R21_SSa           361  -GGLGPIVLNV-------------LDGMIRKA----------VKVWLHL---------------------  385
R2OlA             358  -RKVTVTKLAQ-------------IDGIVRKA----------VKKWLHL---------------------  382
R21_Gav           361  -AGIGRVALEA-------------LDSMNRRK----------VKEWFHL---------------------  385
R21_ZA            362  -SEMKAGALEA-------------LDQQIRTA----------VKDWLHL---------------------  386
R21_TG            362  -SEVKTALLET-------------LDQKIRTA----------VKEWLHL---------------------  386
R21_GFo           362  -SEMKAGALEA-------------LDRTIRSA----------VKDWLHL---------------------  386
R2NS1_CGi         364  -DAYPXXVLGK-------------LDLIVRAA----------IRRWAKL---------------------  388
NeSL1_TV          361  -NVFRVNDLKA-------------TDQMIRGL----------IDKEAPT---------------------  385
Utopia1_ACa       372  -GKIQRGRCQQ-------------FDRELREN----------LRAALRL---------------------  396
Utopia1_LV        364  -TEYLKSDLKP-------------LRAAIIKH----------VKKICHL---------------------  388
Utopia1_CFl       360  -ATVGKKPLKA-------------ADLQVRRM----------AKSWLNL---------------------  384
Utopia1_AEc       360  -ARVFKGPLTA-------------VDLNIRRH----------VKSWLNL---------------------  384
Utopia1_CMy       360  -SAVAKVPLNK-------------ADKIVRQL----------VKKWLFL---------------------  384
Utopia1B_CPB      360  -SAVAKVPLNK-------------ADSTIRQL----------VKKWLYL---------------------  384
Utopia1_ENe       371  -CYPYKQQLVA-------------FDVYVRAA----------LKAAFKL---------------------  395
R52_SM            361  -REIKTMALSA-------------SQGNTNSCGNNSSKLRGHLRRILNL---------------------  395
R5                370  -SRIPFSDFITNRKTNKTTNNSNDSEKSIQKAYDPE--SGQLFLNTFAL---------------------  415
R51_SM            358  -CNINHMTLDT-------------NRNRTVQH----------REKQLGF---------------------  382
LIN9_SM           360  -EDIAKTKIET-------------YDEEIKKM----------IKEDILK---------------------  384
R4_Hmel           368  -INWSQTDLSN-------------LQRIINTH----------MTTHRKH---------------------  392
R42_AS            381  -TNGAGQKLSE-------------ALKHARDI----------DVRIRSL---------------------  405
R4_AL             378  -TNGASPKLLE-------------ALKRAATL----------DTRIRDL---------------------  402
R45_BX            378  QPNSAKADMLK-------------LDMVIREK----------IRRKGLL---------------------  403
HERO1_BF          362  -YDIPLSTADQ-------------VEGKANSF----------VRKWLGV---------------------  386
HEROTn            364  -YEFPISTVEG-------------LERRVSSC----------LRRWLGL---------------------  388
HERO1_SP          367  -YDVPVSQVEK-------------METLVSKK----------LRRWLGA---------------------  391
HERO1_HR          373  YNLSLTWIKNN-------------LDNMVTEA----------VRDWLTM---------------------  398
HERO3_BF          362  -HDLTDTQLQK-------------LDSIHTKA----------IKTWLRM---------------------  386
R44_SRa           351  -EFS-DEERIQ-------------LARNVDIR----------IRSYMNT---------------------  374
Consensus_aa:          ........lp..............h-..lp.h..........l+phh.h.....................
Consensus_ss:               hhhhhh             hhhhhhhh          hhhhh
```

Supplementary Figure 6

Alignment of amino acid sequences used to construct a phylogenetic tree. PROMALS3D (PROfile Multiple Alignment with predicted Local Structures and 3D constraints) software was used. The resulting alignments in FASTA format and with the predicted secondary structures are shown in Figures **A** and **B**, respectively.
