# Supplementary material for: R2 and Non-Site-Specific R2-Like Retrotransposons of the German Cockroach, Blattella germanica
Source: Genes (Basel). 2020 Oct 15;11(10):1202. doi: 10.3390/genes11101202 (PMC7650587; doi:10.3390/genes11101202)
Supplement: Supplementary file 1 [file genes-11-01202-s001.zip › Supplements/Supplementary Figure 7.pdf]

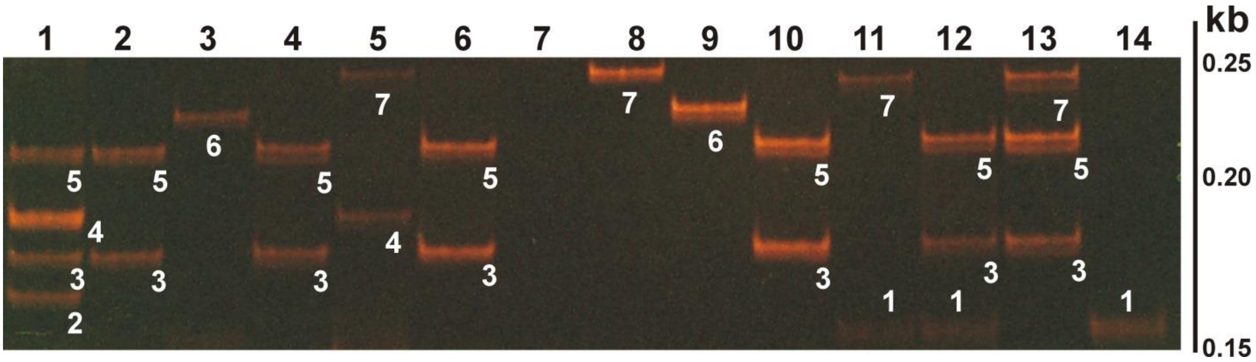

Supplementary Figure 7

Population analysis of the structural variants of R2 retrotransposon 5'-truncated copies.
The result of the electrophoretic separation of the amplification products in 10% acrylamide gels. In each lane (1 – 14), the product of the amplification of the genomic DNA of an individual male was applied. The numbers (white) indicate the seven different identified variants of 5'-truncated copies of the R2 retrotransposon.

Note, the resolution of the analysis method that we used did not allow us to determine the length of the amplified fragments with an accuracy of up to one nucleotide. During electrophoresis, slight distortions of the electrophoretic mobility of fragments located on different tracks can occur; for example, fragment #7 on tracks 5, 8, 11, and 13 shows slightly different electrophoretic mobility. All fragments designated as having a similar size were tested in repeated experiments with the placement of samples on adjacent tracks.
